# The [4+2]‐Cycloaddition of α‐Nitrosoalkenes with Thiochalcones as a Prototype of Periselective Hetero‐Diels–Alder Reactions—Experimental and Computational Studies

**DOI:** 10.1002/chem.201903385

**Published:** 2019-11-22

**Authors:** Grzegorz Mlostoń, Katarzyna Urbaniak, Marcin Jasiński, Ernst‐Ulrich Würthwein, Heinz Heimgartner, Reinhold Zimmer, Hans‐Ulrich Reissig

**Affiliations:** ^1^ Faculty of Chemistry University of Łódź Tamka 12 91403 Łódź Poland; ^2^ Organisch-Chemisches Institut and Center for Multiscale Theory and Computation (CMTC) Westfälische Wilhelms-Universität Münster Corrensstrasse 40 48149 Münster Germany; ^3^ Department of Chemistry University of Zurich Winterthurerstrasse 190 8057 Zurich Switzerland; ^4^ Institut für Chemie und Biochemie Freie Universität Berlin Takustrasse 3 14195 Berlin Germany

**Keywords:** DFT calculations, hetero-Diels–Alder reactions, organic synthesis, periselectivity, thiochalcone

## Abstract

The [4+2]‐cycloadditions of α‐nitrosoalkenes with thiochalcones occur with high selectivity at the thioketone moiety of the dienophile providing styryl‐substituted 4*H*‐1,5,2‐oxathiazines in moderate to good yields. Of the eight conceivable hetero‐Diels–Alder adducts only this isomer was observed, thus a prototype of a highly periselective and regioselective cycloaddition has been identified. Analysis of crude product mixtures revealed that the α‐nitrosoalkene also adds competitively to the thioketone moiety of the thiochalcone dimer affording bis‐heterocyclic [4+2]‐cycloadducts. The experiments are supported by high‐level DFT calculations that were also extended to related hetero‐Diels–Alder reactions of other nitroso compounds and thioketones. These calculations reveal that the title cycloadditions are kinetically controlled processes confirming the role of thioketones as superdienophiles. The computational study was also applied to the experimentally studied thiochalcone dimerization, and showed that the 1,2‐dithiin and 2*H*‐thiopyran isomers are in equilibrium with the monomer. Again, the DFT calculations indicate kinetic control of this process.

## Graphical abstract

**Superdienophiles select one of eight conceivable pathways**! The hetero‐Diels–Alder reactions of α‐nitrosoalkenes with thiochalcones are highly periselective and regioselective processes, providing only one of eight possible constitutional isomers. The thiochalcones undergo a regioselective, but reversible dimerization. High‐level DFT calculations reveal that these processes are kinetically controlled.

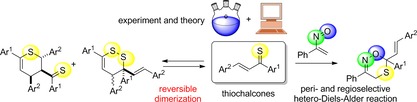

## Introduction

**Scheme 1:**
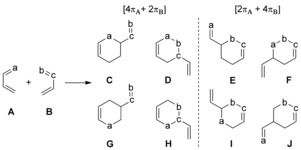
Hypothetical products **C**–**J** of Diels–Alder reactions of heterodiene **A** with heterodiene **B** (for clarity lone pairs at the centers a, b, and c are omitted); the conceivable four [3+2]‐cycloadditions are not depicted.

The employment of cycloaddition reactions^1^ belongs to the most important strategies for the preparation of functionalized carbocyclic and heterocyclic compounds.^2^ The many applications of the (hetero‐)Diels–Alder reaction^3^ and of the 1,3‐dipolar cycloaddition (Huisgen reaction)^4^ for the selective and efficient formation of six‐ and five‐membered ring systems prove the particular relevance of these cycloadditions. In general, they proceed as concerted reactions and are classified as periselective processes that can be treated by the Woodward–Hoffmann rules.^5^ Although countless examples of these cycloadditions have been reported, there are still puzzling selectivity issues and mechanistic problems.^6^, ^7^ One interesting selectivity challenge arises if two 4 π‐systems are involved in (hetero‐)‐ Diels–Alder reactions. In the case of the cycloadditions of a heterodiene **A** (one heteroatom a) with a second heterodiene of general structure **B** (two heteroatoms b and c) the hypothetical formation of eight constitutional isomers  **C**–**J** can be depicted (Scheme 1). Products **C** and **D** (and **G** and **H**) would be the result of [4 π_A_+2 π_B_] reactions, whereas the isomeric compounds **E** and **F** (and **I** and **J**) would arise from [2 π_A_+4 π_B_] processes. Given that different perimeters of the two π‐systems are involved, the term periselectivity as introduced by Houk^8^ is applicable for the relationship of **C**–**F**.^9^ For each of these cycloadducts the regioisomers **G**–**J** are conceivable. In addition to these hetero‐Diels–Alder reactions, [3+2]‐cycloadditions of the s‐*trans*‐conformer of heterodiene **B** with **A** can form four isomeric five‐membered heterocycles that incorporate a 1,3‐dipole moiety. Although all reactions are formally allowed by the Woodward–Hoffmann rules, it is evident that not all products are favored by kinetic and/or thermodynamic factors. To our best knowledge, this type of periselective and regioselective reactions has been rarely studied.^10^

**Scheme 2:**
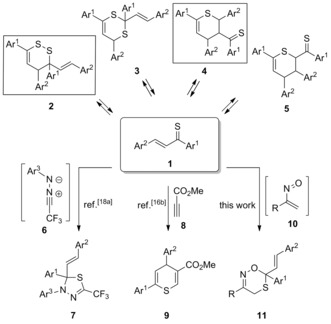
Monomeric thiochalcones **1** equilibrating with dimers **2** and **4**, and trapping of **1** through [3+2]‐ and [4+2]‐cycloadditions by using fluorinated 1,3‐dipoles or acetylenic dienophiles, respectively, and periselective hetero‐Diels–Alder reactions of **1** with α‐nitrosoalkenes **10** leading to cycloadducts **7**, **9**, and **11**.

In this report, we describe two types of hetero‐Diels–Alder reactions of 1,3‐diaryl‐substituted α,β‐unsaturated thioketones **1** (known as thiochalcones, a=S) that represent heterodiene **A**. Firstly, the reversible dimerization of these compounds is analyzed and secondly their hetero‐Diels–Alder reactions with α‐nitrosoalkenes **10** (b=O, c=N) as second heterodiene component **B**. Thiocarbonyl compounds and especially non‐enolizable thioketones are well known as versatile building blocks for the preparation of sulfur heterocycles with variable ring size through cycloaddition reactions.^11^ Based on kinetic studies, Huisgen et al. named these compounds superdipolarophiles12a–12c and superdienophiles12d, 12e to emphasize their high reactivity towards 1,3‐dipoles and dienes, respectively. In comparison with aryl‐, hetaryl‐, and ferrocenyl‐substituted thioketones, the related thiochalcones with the general structure **1** are much less explored in cycloaddition chemistry. Noteworthy, thiochalcones **1** exist in solutions as equilibrium mixtures of monomeric and dimeric forms, namely 3,4‐dihydro‐1,2‐dithiin **2** and 3,4‐dihydro‐2*H*‐thiopyran derivatives **4** (Scheme 2).^13^ The dimeric products are formed through thia‐Diels–Alder reactions with thiochalcones playing at the same time the role of the heterodiene and the C=S dienophile (→1,2‐dithiins **2** or 1,3‐dithiins **3**) or C=C dienophile (→thiopyrans **4** or **5**). Surprisingly, in contrast to several α,β‐unsaturated thioaldehydes such as thioacrolein, there is no indication that thiochalcones **1** provide the regioisomeric dimers **3** (1,3‐dithiins) or the regioisomeric 3,4‐dihydro‐2*H*‐thiopyran **5**.13d

In pioneering work, Lewis acid‐mediated hetero‐Diels–Alder reactions of several thiochalcones **1** with activated ethylenes including chiral fumarates and maleates have been described.^14^ In contrast, in our recent publication, organocatalytic asymmetric [4+2]‐cycloadditions of thiochalcones **1** as heterodienes with in situ generated enantiopure dienamines derived from O‐silylated l‐prolinols have also been demonstrated.^15^ In addition, we described thia‐Diels–Alder reactions of **1** acting as heterodienes with acetylenic dienophiles16a, 16b as well as with 1,4‐quinones.16c Notably, in the case of unsymmetrically activated acetylenes such as **8**, the [4+2]‐cycloadditions occurred with complete regioselectivity, and the sulfur atom attacked always the β‐position of the Michael‐type acceptor to give 4*H*‐thiopyrans **9** (Scheme 2).16b In contrast to the well‐established behavior of chalcones as reactive C=C dipolarophiles,^17^ [3+2]‐cycloadditions of thiochalcones **1** are almost unknown.^18^ Only in a recent publication,18a it was demonstrated that the 1,3‐dipolar cycloadditions of in situ generated electron‐deficient fluorinated nitrile imines **6**, derived from trifluoroacetonitrile, occur in a fully chemo‐ and regioselective fashion onto the C=S bond of **1**. The thioketone moiety played the role of a most suitable heterodipolarophile and 2,3‐dihydro‐1,3,4‐thiadiazoles **7** were obtained as products of these reactions (Scheme 2).

α‐Nitrosoalkenes constitute an exceptional class of highly reactive heterodienes, and their Diels–Alder reactions with C=C and C≡C dienophiles have been studied by experiments^19^ and with computational methods.^20^ Confirming an early study on α‐nitrosoalkene/thioketone cycloadditions,^21^ we recently described regioselective hetero‐Diels–Alder reactions of aryl‐, hetaryl‐, and ferrocenyl‐substituted thioketones with in situ generated electron‐deficient α‐nitrosoalkenes **10**. The corresponding 4*H*‐1,5,2‐oxathiazine derivatives were obtained as the only products in a regio‐ and chemoselective manner.^22^ For the first time, alkyl‐substituted thioketones were successfully reacted with α‐nitrosoalkenes **10** also giving the expected heterocycles in good yields. In a preliminary experiment with 1,3‐diphenylprop‐2‐ene‐1‐thione (**1 a**, Ar^1^, Ar^2^=Ph) and 1‐nitroso‐1‐phenylethylene (**10 a**, R=Ph) it was found that in this case the C=S bond was involved in the [4+2]‐cycloaddition reaction, yielding the corresponding 4‐styryl‐substituted 4*H*‐1,5,2‐oxathiazine of type **11** as major product.

The goal of the present work was the systematic examination of hetero‐Diels–Alder reactions of selected in situ generated α‐nitrosoalkenes **10** with a series of electron‐rich aryl‐, hetaryl‐, and ferrocenyl‐substituted thiochalcones **1** to establish scope and limitations of this periselective process. The obtained experimental results should be rationalized by theoretical studies that were also applied to the thiochalcone dimerization and the cycloadditions of related nitroso compounds and thioketones.

## Results and Discussion

### Synthesis of thiochalcones and dimerization

**Scheme 3:**
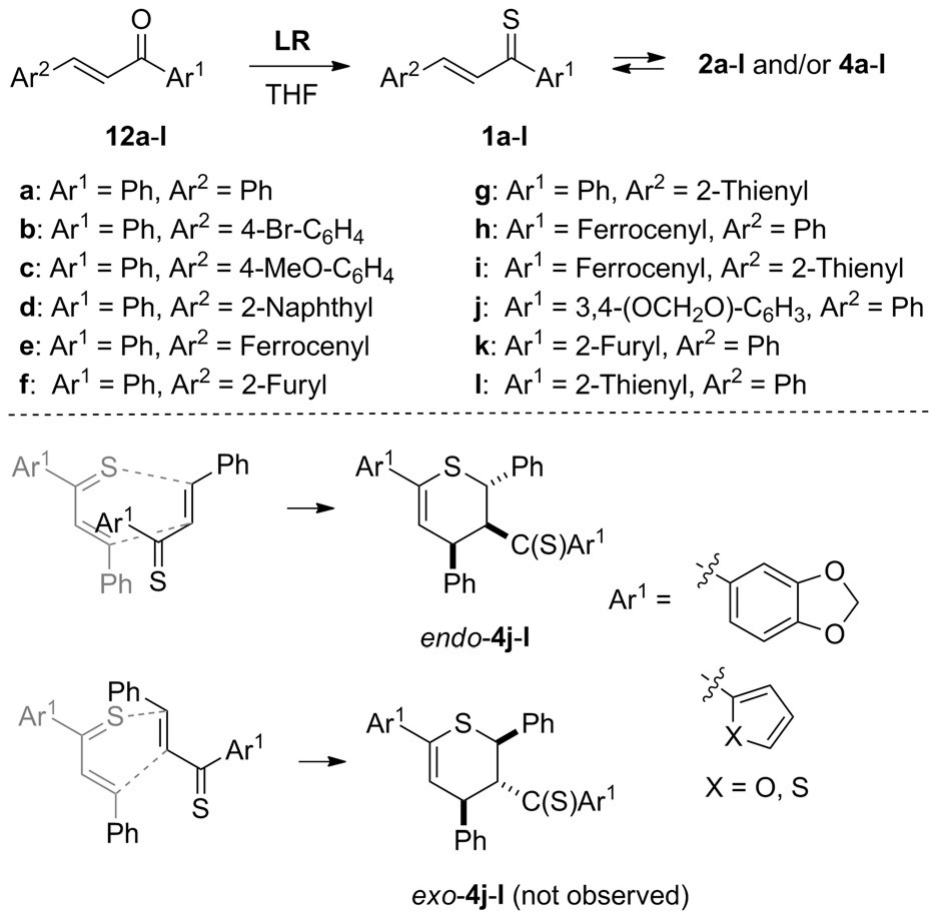
Synthesis of thiochalcones **1** **a**–**l** through thionation of chalcones **12** **a**–**l** with Lawesson's reagent (**LR**), and the structure of isolated 3,4‐dihydro‐2*H*‐thiopyran derivatives **4** **j**–**l**.

A series of thiochalcones **1 a**–**l** bearing diverse aryl, hetaryl, and ferrocenyl substituents was selected for the present study. All compounds were prepared according to a known procedure based on the treatment of the corresponding chalcones **12** with Lawesson's reagent (**LR**) (Scheme 3).^16^ The “thiochalcone fractions” containing predominantly mixtures of dimers **2** and **4** were isolated chromatographically and subsequently used for further studies without separation of the components. In analogy to earlier reports,13b, 13c compounds bearing phenyl (**1 a**–**g**) or ferrocenyl (**1 h**, **i**) groups located at the C=S group (Ar^1^) provided complex mixtures of dimers **2** and **4**, and depending on the type of substituents, the composition of the mixtures slightly differed in the studied cases. For example, the “thiochalcone fraction” of **1 a** was isolated chromatographically as a solid (**2 a**:**4 a** in approx. 1:1 ratio). When treated with petroleum ether at room temperature, a colorless solid precipitated, which was separated by filtration, recrystallized from benzene/diethyl ether and was studied by spectroscopy. The ^1^H NMR spectrum immediately recorded in CDCl_3_ solution at room temperature revealed the presence of two diastereomeric 1,2‐dithiins, *cis*‐ and *trans*‐**2 a**, as major components, and the structures of these isomers were confirmed by 2D NMR methods (for details, see the Supporting Information). The calculated NMR data also confirm the structure of these dimers **2 a** as well as their isomers **4 a**.

However, during the storage of the solution of 1,2‐dithiins *cis*‐ and *trans*‐**2 a** at room temperature overnight, the color turned blue and the ^1^H NMR spectrum evidenced that, again, an equilibrium mixture containing diastereomers **2 a** and 3,4‐dihydro‐2*H*‐thiopyran derivative **4 a** exists in a 1:1:5 ratio. In addition, another set of signals was found in this NMR spectrum which indicated the presence of trace amounts of another dimeric product, presumably one of the two possible diastereomeric 1,3‐dithiins **3 a**.^23^ These observations fit well with the literature report that two isomers of 1,2‐dithiins of type **2** form the dominant fraction of the thiochalcone dimers bearing Ph (for Ar^1^) and SPh (instead of Ar^2^) substituents. This constitution was confirmed by a desulfurization experiment with Raney nickel, which led to isolation of 1,4‐diphenylhexane as the major product.13b

A similar solvent‐dependent equilibrium shift was observed for other “thiochalcone fractions” of compounds bearing *para*‐substituted phenyl (**1 b** and **1 c**) and naphthyl (**1 d**) groups. However, in the case of analogs bearing the ferrocenyl group (**1 e**, **1 h**, and **1 i**) or with a hetaryl moiety located at the β‐position (**1 f**, **1 g**), the ^1^H NMR spectra of the purified material revealed either a very complex pattern or significant broadening of the signals was observed. Therefore, a reliable interpretation of the composition of the resulting mixtures was not possible. In contrast, more electron‐rich analogs bearing 3,4‐methylenedioxyphenyl (**1 j**), 2‐furyl (**1 k**), and 2‐thienyl (**1 l**) substituents as Ar^1^ groups, provided almost exclusively 3,4‐dihydro‐2*H*‐thiopyrans **4 j**–**l** as single diastereomers. These compounds were isolated and characterized spectroscopically. For example, the ^1^H NMR spectrum of **4 j** revealed the presence of a single set of diagnostic signals at 4.04 (dd, *J=*4.2, 6.5 Hz, HC(4)), 5.02 (d, *J=*11.1 Hz, HC(2)), 5.17 (dd, *J=*4.2, 11.1 Hz, HC(3)), and 6.24 ppm (d, *J=*6.5 Hz, HC(5)), attributed to hydrogen atoms of the central thiopyran ring. Moreover, the coupling constants *J=*4.2–4.4 Hz between the axial HC(3) and the equatorial HC(4) observed for the series **4 j**–**l** strongly support the structure of *endo*‐cycloadducts (Scheme 3).^13^ Notably, also in the case of the **a**–**d** series only *endo*‐dimers **4** could be identified in the mixtures.

### Hetero‐Diels–Alder reactions of thiochalcones with α‐nitrosoalkenes

**Scheme 4:**
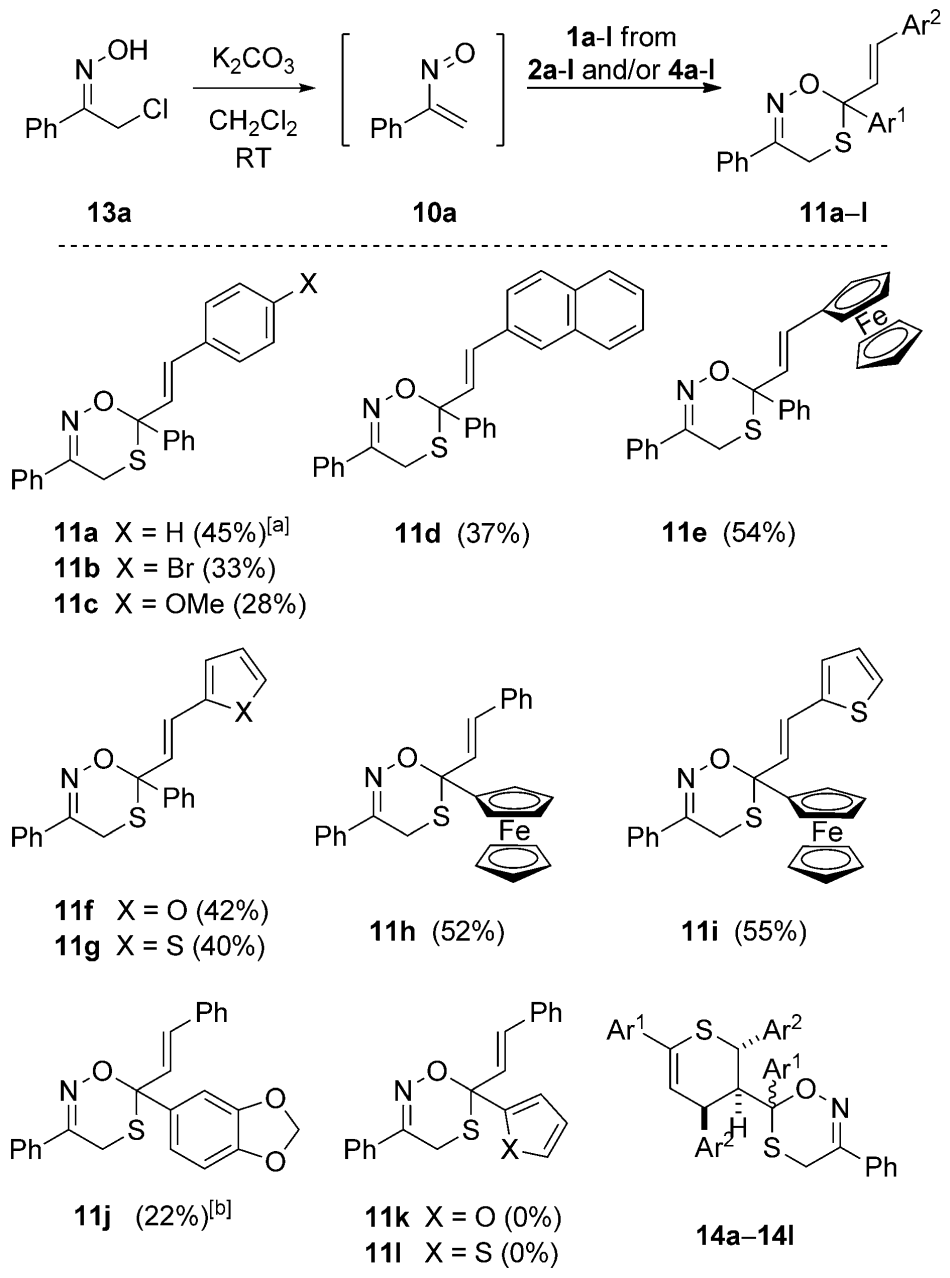
Cycloadditions of in situ generated 1‐nitroso‐1‐phenylethene (**10** **a**) with thiochalcones **1** **a**–**l** (in equilibria with their dimers **2** and/or **4**) leading to 4*H*‐1,5,2‐oxathiazines **11** **a**–**j**; (except from **14** **b**, side products of type **14** deriving from thiochalcone dimers *endo*‐**4** were not isolated). [a] Reaction performed at 0 °C; [b] Not isolated; yield estimated based on the ^1^H NMR of crude reaction mixture.

It was assumed that the [4+2]‐cycloaddition reactions with α‐nitrosoalkenes **10** occur with monomeric thiochalcones **1** that are present in mixture with their dimers **2**/**4** and therefore different rates depending on the monomer stationary concentration in the reaction solution can be expected. Employing an established method,^24^ α‐nitrosostyrene **10 a** was generated in situ by treatment of α‐chlorooxime **13 a** (two equivalents) with potassium carbonate in dry dichloromethane at room temperature. The heterogeneous conditions guarantee low concentrations of **10 a** and hence minimize the oligomerization of this reactive species. Due to the anticipated limited stability of the heterocyclic products, the reactions of **13 a** with the dimers of thiochalcones **1 a**–**l** were carried out at room temperature (or below), analogously to the already reported experiments with simple thioketones (Scheme 4).^22^ The conversions were monitored by TLC until the spots of the starting thiochalcone dimers completely disappeared. The ^1^H NMR analysis of the crude reaction mixtures confirmed the formation of the expected [4+2]‐cycloadducts of type **11**, contaminated in most of the cases by side products **14** (in general less than 10 %). The fairly stable major products were isolated by standard column chromatography in moderate to good yields up to 55 %, and the structure of the hitherto unknown 4*H*‐1,5,2‐oxathiazine derivatives **11 b**–**i** was confirmed by standard spectroscopic methods supplemented with mass spectrometry and combustion analysis. For example, in the case of the ferrocenyl‐substituted 4*H*‐1,5,2‐oxathiazine **11 e**, characteristic resonances attributed to C(3), C(4), and C(6) were found in the ^13^C NMR spectrum at 151.7, 23.5, and 87.0 ppm, respectively. In the constitutional isomer **11 h**, bearing the ferrocenyl group at C(6), the corresponding signals were found at similar regions (152.1, 23.8, and 88.6 ppm). The attempted synthesis of 4*H*‐1,5,2‐oxathiazine derivatives **11 j**–**l** performed under analogous reaction conditions (starting with dimers **4 j**–**l** bearing electron‐donating substituents) led to the expected product only in a very low yield (**11 j**) or failed completely (**11 k**, **11 l**). Instead, the formation of [4+2]‐cycloadducts **14 k** and **14 l** as mixtures of diastereomers was observed (Scheme 4). These results can be rationalized by the fact that the respective precursor dimers **4** exhibit enhanced stability and do not release sufficient amounts of the monomeric thiochalcone **1** at room temperature. Therefore, the reaction of heterodiene **10 a** occurs mainly or exclusively with the dimers **4** leading to the formation of cycloadducts **14**.

To gain more insight into this relationship of hetero‐Diels–Alder reactions of monomer **1** and/or dimer **4**, the reactions of the parent “thiochalcone fractions” **2**/**4** with **10 a** were studied in more detail and the crude product mixtures were analyzed by ^1^H NMR spectroscopy (Table 1). When the reaction of **2 a**/**4 a** with **10 a** was performed under standard conditions (CH_2_Cl_2_, RT), the products **11 a** and **14 a** were formed in an approximate 4:1 ratio in favor of (*E*)‐3,6‐diphenyl‐6‐styryl‐4*H*‐1,5,2‐oxathiazine (**11 a**), which was finally isolated in 39 % yield (Table 1, entry 1). To evaluate the influence of daylight, the same reaction was performed under light exclusion, but essentially the same mixture of **11 a** and **14 a** was obtained. Notably, decreasing the reaction temperature to 0 °C led to a similar mixture, in which the components **11 a** and **14 a** were found in an approximate 9:1 ratio, and the major product **11 a** was isolated in slightly higher yield of 45 % after a remarkably longer reaction time of 20 h (Table 1, entry 2). Finally, a “thiochalcone fraction” enriched in dimer *endo*‐**4 a** was prepared by equilibration of the dimer sample in CH_2_Cl_2_ overnight. By reaction with **10 a**, the respective products **11 a** and **14 a** were now formed in an approximate 1:2 ratio (Table 1, entry 3). Unfortunately, an attempted isolation of pure samples of diastereomers **14 a** by chromatography techniques failed due to similar polarity and limited stability of these cycloadducts. Noteworthy, the ^1^H NMR analysis of the fractions obtained by column chromatography revealed the presence of higher amounts of **11 a** and the dimer *endo*‐**4 a**, which indicates a possible decomposition of **14 a** by cycloreversion across the thiopyran ring.

**Table 1:** Ratio of products **11** and **14** formed in the reactions of model nitrosoalkene **10 a** with “thiochalcone fractions” **2**/**4** as determined by ^1^H NMR spectroscopy of the crude product mixture^[a]^

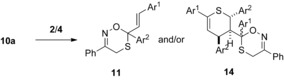		
Entry	Thiochalcone fraction **2**/**4**	Ratio of crude product mixture **11**:**14**:**14'**
1	**a**	78:20:2
2	**a** ^[b]^	89:10:1
3	**a** ^[c]^	29:65:6
4	**b**	67:29:4
5	**b** ^[c]^	31:59:10
6	**c**	48:35:17
7	**d**	70:29:1
8	**e**	100:0:0
9	**f**	87:11:2
10	**g**	65:28:7
11	**h**	100:0:0
12	**i**	100:0:0
13	**j**	22:53:25
14	**k**	0:68:32
15	**l**	0:79:21

[a] Reaction conditions: CH_2_Cl_2_, room temperature, K_2_CO_3_, **13 a** (2.0 equiv), thiochalcone fraction **2**/**4**. [b] Reaction performed at 0 °C. [c] Reaction carried out by using thiochalcone fraction **2**/**4** pre‐equilibrated in CH_2_Cl_2_ overnight at room temperature.

Similar results were noticed in the reaction of **10 a** with *p*‐bromophenyl‐substituted derivative **2 b**/**4 b**. The standard “thiochalcone fraction” mainly furnished **11 b** and minor amounts of **14 b**/**14 b'**, whereas a “thiochalcone fraction” enriched in **4 b** provided **14 b/14 b'** as major components (Table 1, entries 4 and 5). In this case, the subsequent purification by preparative TLC enabled isolation of small samples of both diastereomeric cycloadducts, **14 b** (9 %) and **14 b'** (2 %), obtained as a glassy solid and a thick colorless oil, respectively. The spectroscopic analysis of both products confirmed the anticipated bis‐heterocyclic structure of **14 b** and **14 b'** formed through [4+2]‐cycloaddition of **10 a** with the dimeric thiochalcone, *endo*‐**4 b**. For example, in the ^1^H NMR spectrum of **14 b**, the signals attributed to the hydrogen atoms of the thiopyran ring were found at 3.61 (dd, *J=*3.5, 9.1 Hz, HC(3′)), 4.40 (dd, *J=*3.5, 6.6 Hz, HC(4′)), 4.97 (d, *J=*9.1 Hz, HC(2′)), and 6.36 ppm (d, *J=*6.6 Hz, HC(5′)), whereas the diagnostic resonances of the CH_2_ group of the 1,5,2‐oxathiazine unit were located at 2.58 and 3.25 ppm (AB system, *J=*16.8 Hz). In the case of **14 b'**, a different pattern of signals was observed: the HC(3′) and HC(4′) resonances appeared as broadened *pseudo*‐triplets (for details, see the Supporting Information), whereas the highfield signals of the CH_2_ group were found at 2.97 and 3.17 ppm (AB system, *J=*17.3 Hz). Thus, based on the characteristic chemical shifts observed for the diastereomeric compounds of type **14**/**14'** in the ^1^H NMR spectra, all products formed as *major* diastereomers in the series are considered to have the analogous relative configurations at the newly generated stereogenic center at C(6) of the 1,5,2‐oxathiazine ring. Unfortunately, the relative configuration could not be determined by spectroscopic methods and the attempted isolation of pure crystalline compounds of type **14**/**14'** suitable for X‐ray diffraction was also unsuccessful.

The observed results of these reactions collected in Table 1 clearly demonstrate that the proportions of cycloadducts **11** and **14** formed under standard conditions reflect to some extent the composition of the “thiochalcone fraction”. Introduction of an electron‐donating group at the Ar^1^ substituent (**j**, **k**, and **l**) in thiochalcone **1** (entries 13–15) or previous equilibration of “thiochalcone fractions” in CH_2_Cl_2_ (entries 3 and 5) increases the content of the *endo*‐**4** dimer in the mixture, and hence, favors the formation of bis‐heterocyclic products of type **14**/**14'**. To the best of our knowledge, these are the first examples of [4+2]‐cycloadditions of an electron‐deficient heterodiene with dimeric thiochalcones of type **4** playing the role of the electron‐rich C=S heterodienophile. In contrast, thiochalcones bearing the bulky ferrocenyl group either as Ar^1^ (**h**, **i**) or Ar^2^ (**e**) substituents lead to the expected monocyclic products **11** exclusively (entries 8, 11, and 12), very likely due to higher stationary concentrations of the monomeric thiochalcones **1**.

**Scheme 5:**
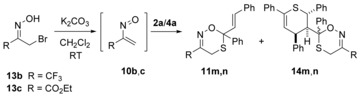
Reactions of very electron**‐**deficient **α‐**nitrosoalkenes **10** **b** and **10** **c** generated from oximes **13** **b** or **13** **c**, respectively, with the dimers **2** **a**/**4** **a** and its monomer **1** **a**.

In extension of the study, two particular electron‐deficient α‐nitrosoalkenes, **10 b** (R=CF_3_) and **10 c** (R=COOEt) derived from 3‐bromo‐1,1,1‐trifluoro‐2‐(hydroxyimino)propane (**13 b**) and ethyl 3‐bromo‐2‐(hydroxyimino)propionate (**13 c**), respectively, were also tested in cycloadditions with the parent system **2 a**/**4 a** (Scheme 5). By reacting of in situ generated **10 b** with **1 a**, the attempted preparation of the trifluoromethyl‐substituted analog of 4*H*‐1,5,2‐oxathiazine **11 a** gave a mixture of products identified as **11 m** and bis‐heterocyclic adducts **14 m** and **14 m'** formed in an approximate 6:3:1 ratio. However, the attempted isolation of these products by column chromatography resulted in complete decomposition. This observation is consistent with the previously described results of the reactions of the fluorinated α‐nitrosoalkene **10 b** with simple thioketones,^22^ which did not afford stable products in most of the performed hetero‐Diels–Alder reactions. Finally, reaction of **1 a** with in situ generated α‐nitrosoalkene **10 c**, bearing an ethoxycarbonyl group, led to a mixture containing **11 n**, **14 n** and **14 n'** in an approximate 2:5:2 ratio. The subsequent chromatographic purification followed by fractional crystallization enabled the isolation of a pure sample of the **14 n** in 26 % yield.

In summary, the presented preparative results show that the composition of the “thiochalcone fraction” considerably determines the type of products formed, and that the substituent present in the heterodiene is of importance. In the studied system of the two heterodienes **1** and **10**, the former component plays exclusively the role of the heterodienophile, reacting periselectively and regioselectively at the C=S bond, whereas the α‐nitrosoalkene acts as heterodiene to give styryl‐substituted 4*H*‐1,5,2‐oxathiazines **11**. The C=C bond of these cycloadducts offers many options for further functionalization and hence libraries of unique heterocycles should be available for examination of their properties, for example, as biologically active compounds.

## Computational Study

### Methods and procedure

To study the reaction mechanisms of the dimerization and the hetero‐Diels–Alder reactions in detail, high‐level DFT calculations (PBE1PBE/def2‐TZVP+PCM(dichloromethane)+GD3BJ dispersion correction^26^, ^27^, ^28^, ^29^ on the basis of preceding B3LYP/6‐31G(d)^30^+GD3BJ geometry optimizations) were performed. In the following part, we report relative Gibbs free enthalpies with respect to the sum of respective educts *s‐trans‐* **1 a** and **10 a** (Δ*G*_298_, kcal mol^−1^, see the Experimental Part and the Supporting Information for details). This study concentrates on classical [4+2]‐cycloadditions of closed‐shell species; open‐shell species (radicals, radical cations, for example, by influence of light) were not considered in this work. Possible catalytically active species like Brønsted or Lewis acids and bases present in the reaction mixtures were not considered in the calculations.10d NMR‐calculations for **2 a**, *endo*‐**4 a** and for **11 a** are in accord with the experimental findings and support the reported constitutions and configurations of the products. Only products derived from *E*‐thiochalcone **1 a** were considered.

### Dimerization

**Scheme 6:**
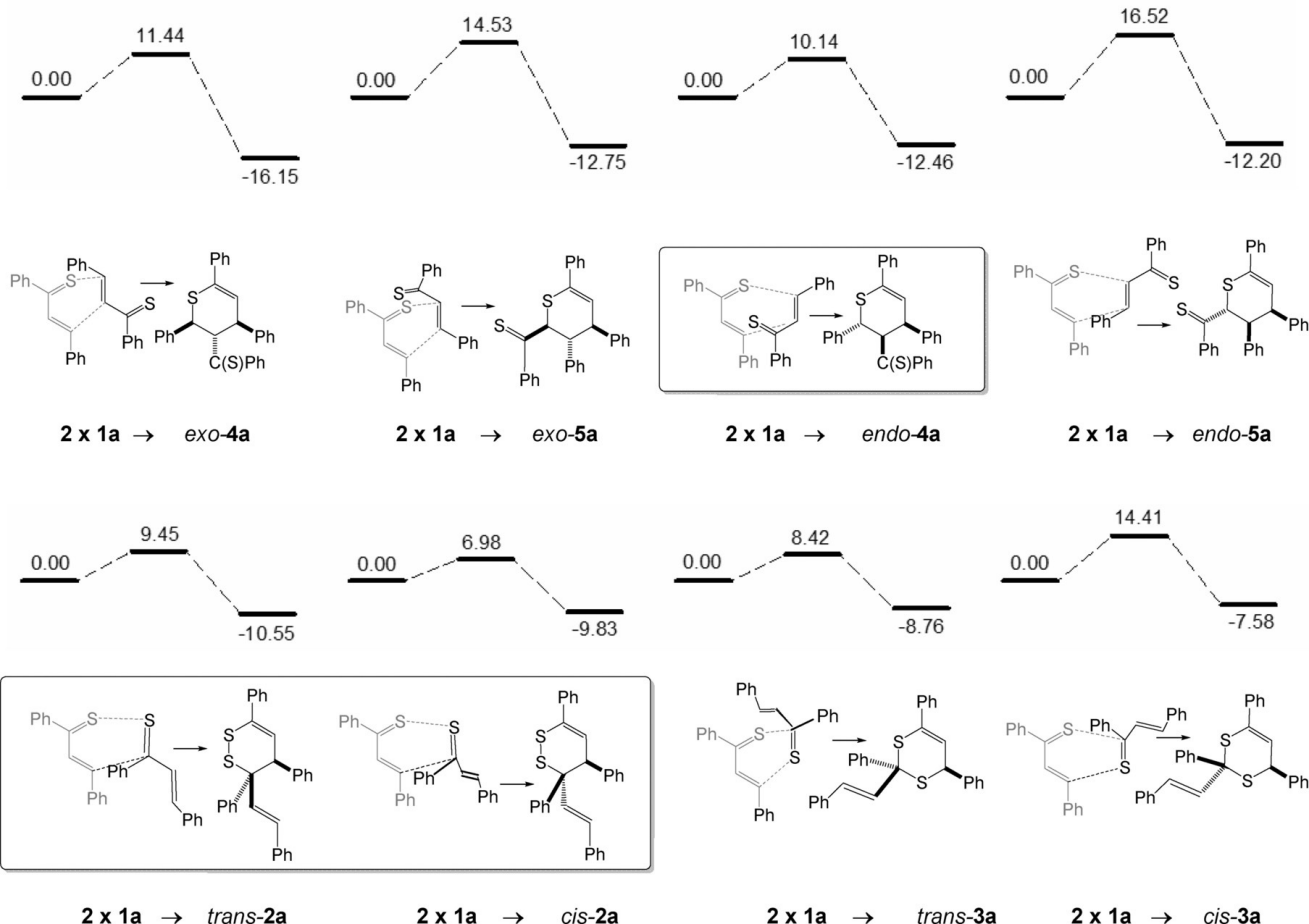
Results of the DFT‐calculations for the dimerization reaction of *E*‐thiochalcone **1 a** leading to (hypothetical) cycloadducts **2 a**–**5 a** (relative Gibbs free enthalpies Δ*G*_298_ are given in kcal mol^−1^). The experimentally observed dimers *endo*‐**4 a** and *cis*/*trans*‐**2 a** are depicted in frames.

In principle, the thiochalcone **1 a** may form the four isomeric products **2**–**5** of hetero‐Diels–Alder‐reactions (see Scheme 2 and Scheme 6) if only the *E*‐form of **1 a** is considered, and for each of the four products two diastereomers are possible due to the *exo*‐ or *endo*‐approaches of the components. The four principal products may be distinguished according to the number of bonds between the two sulfur atoms (1–4 bonds are possible). For all of the eight possible isomers, total and relative enthalpies were calculated and Scheme 6 summarizes the values with decreasing stability of the products. Among all isomers considered, compound *exo*‐**4 a** with a 1,5‐*S*,*S*‐arrangement came out to be by far the thermodynamically most stable isomer (−16.2 kcal mol^−1^) and second is dimer *exo*‐**5 a** (1,4‐*S*,*S*‐arrangement, −12.8 kcal mol^−1^). The diastereomers *endo*‐**4 a** and *endo*‐**5 a** are both slightly less stable compared with the respective *exo*‐isomers (−12.5 and −12.2 kcal mol^−1^). They are followed by dimer *trans*‐**2 a** with an S−S‐bond within the heterocycle (1,2‐*S*,*S*‐arrangement) and its diastereomer *cis*‐**2 a** (−10.6 and −9.8 kcal mol^−1^). The least stable dimers among these isomers are *trans*‐**3 a** and *cis*‐**3 a** (1,3‐*S*,*S*‐arrangement). In all cases, the diastereomers with a *trans*‐arrangement of the most spacious groups were calculated to be lower in enthalpy than the respective *cis*‐arrangements. Experimentally, the isomer *endo*‐**4 a** was found together with *cis*/*trans*‐**2 a** (see framed compounds in Scheme 6).

**Scheme 7:**
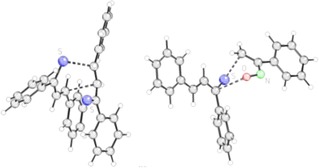
Calculated transition state structures of *endo*‐**4** **a**‐**TS** (left) and of **11** **a**‐**TS** (right). In both examples the transition state relevant C⋅⋅S distances are significantly shorter compared to the distances between the C⋅⋅C respectively C⋅⋅O atoms. *endo*‐**4** **a**‐**TS** (left; C⋅⋅S distance 2.307 Å; C⋅⋅C distance 2.726 Å; imaginary frequency −205.55 cm^−1^); **11** **a**‐**TS** (right; C⋅⋅S distance 2.483 Å; O⋅⋅C distance 2.953 Å; imaginary frequency −125.03 cm^−1^). Hydrogen: white, carbon: grey, nitrogen: green, oxygen: red, sulfur: blue.

Furthermore, the transition states for the formation of the products in the sense of a synchronous, but possibly asymmetric reaction pathway were elucidated. According to these calculations, kinetic stability is expected for the heterocycles *exo*‐**4 a**, *exo*‐**5 a**, *endo*‐**4 a** (for structure, see Scheme 7, left), *endo*‐**5 a** and *cis*‐**3 a** with barriers for a cycloreversion of 22 kcal mol^−1^ or more. Slightly smaller barriers were obtained for the cycloreversions of dimers *trans*‐**2 a**, *cis*‐**2 a**, and *trans*‐**3 a**, which indicate that these species may be subject of equilibration under the reaction conditions (room temperature, dichloromethane). These values are in good agreement with the NMR‐spectroscopic evidence for the equilibration of isomers *trans*‐ and *cis*‐**2 a** and *endo*‐**4 a** (see above).

For the dimerization, low barriers (<12 kcal mol^−1^) were calculated for the isomers *exo*‐**4 a**, *endo*‐**4 a** (Scheme 7, left), *trans*‐**2 a**, *cis*‐**2 a**, and *trans*‐**3 a**, whereas *exo*‐**5 a**, *endo*‐**5 a**, and *cis*‐**3 a** have barriers higher than 14 kcal mol^−1^. Among the thermodynamically most stable calculated isomers, the one with the lowest barrier of 10.1 kcal mol^−1^ is compound *endo*‐**4 a**, which was experimentally found and unambiguously characterized by NMR spectroscopy and calculation of the NMR data. Thus, the dimerization of thiochalcone **1 a** is considered to be an essentially kinetically controlled process, leading to isomers *trans*‐ and *cis*‐**2 a** and the third most stable isomer *endo*‐**4 a**, but not to the thermodynamically most stable form *exo*‐**4 a**. The fact, that dimers *trans*‐ and *cis*‐**3 a** are not observed (within the analytical limits) may be due to the reversibility of their formation.

### Hetero‐Diels–Alder reactions with nitroso compounds

**Scheme 8:**
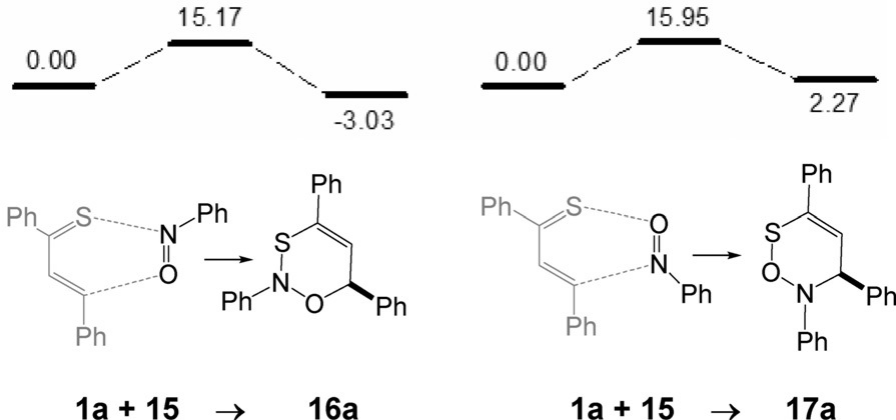
Results of the DFT**‐**calculations for the reaction of *E* **‐**thiochalcone **1** **a** with nitrosobenzene **15** to heterocycles **16** **a** and **17** **a** (relative Gibbs free enthalpies Δ*G*_298_ are given in kcal mol^−1^).

The cycloaddition of s‐*trans*‐*E*‐thiochalcone **1 a** with nitrosobenzene **15** was first computed as model reaction for the following hetero‐Diels–Alder reactions (Scheme 8). From the calculations, two products **16 a** and **17 a** generated by the [4+2]‐cycloadditions have to be expected, both of fairly low thermodynamic and kinetic stability. The three contiguous heteroatoms within the six‐membered ring may be the reason for the low stability of these cycloadducts. We are not aware of an experimental investigation of this reaction, but the calculations indicate that an equilibrium mixture of the precursors **1 a** and **15** and cycloadduct **16 a** can be expected.

**Scheme 9:**
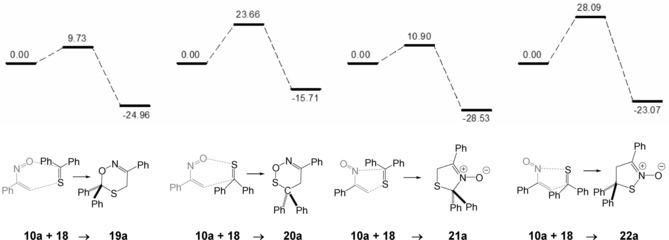
Results of the DFT**‐**calculations for the reaction of α‐nitrosostyrene **10** **a** with thiobenzophenone **18** leading to [4+2]**‐**cycloadducts **19** **a** and **20** **a** or [3+2]**‐**cycloadducts **21** **a** and **22** **a** (relative Gibbs free enthalpies Δ*G*_298_ are given in kcal mol^−1^).

In contrast, the calculations for the cycloaddition of α‐nitrosostyrene **10 a** with thiobenzophenone **18** reveal that in this case the reaction proceeds over a relatively small barrier (9.7 kcal mol^−1^) to a rather stable six‐membered ring product **19 a** (−26.0 kcal mol^−1^). The formation of regioisomer **20 a** requires a quite large activation barrier (Scheme 9). Remarkably, the formation of a five‐membered heterocycle **21 a** by a [3+2]‐cycloaddition requiring the s‐*trans*‐conformer of **10 a** seems also possible, since its barrier is calculated to be only slightly higher (10.9 kcal mol^−1^) and it shows pronounced thermodynamic stability (−28.5 kcal mol^−1^). The formation of the less stable regioisomer **22 a** requires a substantially higher activation enthalpy. Within the experimental limits, the formation of nitrones of type **21 a** was not observed.^22^, ^31^, ^32^ Again, these data reveal that kinetic control of the cycloaddition is responsible for the formation of the experimentally found cycloadduct **19 a**.

**Scheme 10:**
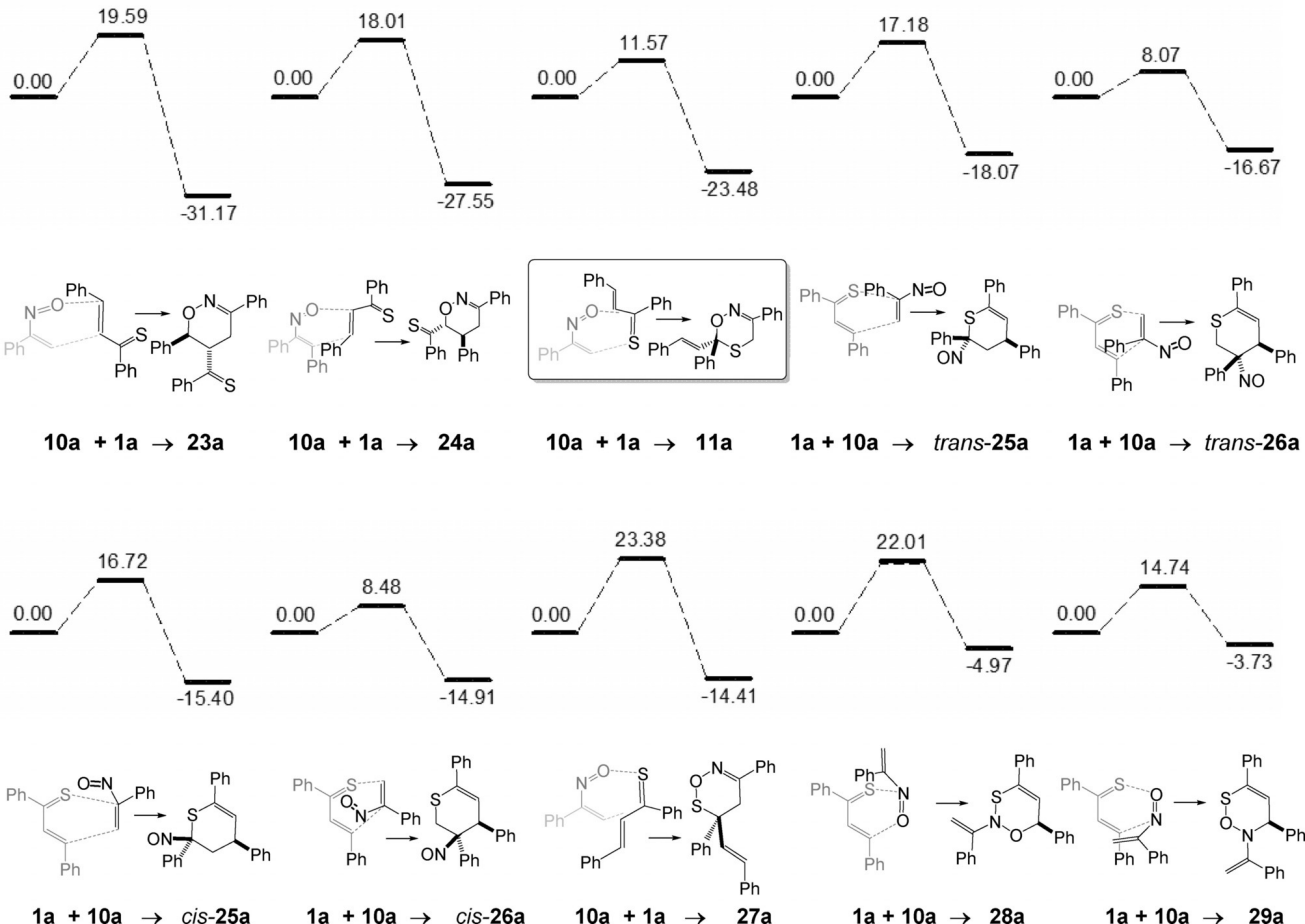
Results of the DFT**‐**calculations for the reaction of *E* **‐**thiochalcone **1** **a** with α‐nitrosostyrene **10** **a** (relative Gibbs free enthalpies Δ*G*_298_ are given in kcal mol^−1^).

Following the situation sketched in Scheme 1, the thiochalcone–nitrosoalkene cycloadditions of (*E*)‐**1 a** and **10 a** can afford eight different isomers (two of them with *exo*/*endo* diastereomers). Scheme 10 arranges the products following their calculated stability, among these isomers, two of them show a S−O bond (**27 a**, **29 a**; 1,2‐S,O‐arrangement), two of them 1,3‐S,O‐ (**11 a**, (Scheme 7, right), **28 a**), 1,4‐S,O‐ (**24 a**, *trans*‐**25 a**, *cis*‐**25 a**), and 1,5‐S,O‐arrangements (**23 a**, *trans*‐**26 a**, *cis*‐**26 a**). The most stable calculated isomer is isomer **23 a** (−31.2 kcal mol^−1^; 1,5‐S,O‐distance), followed by isomers **24 a** and the experimentally found **11 a** (−23.5 kcal mol^−1^, Scheme 7, right). As expected, isomers with three contiguous heteroatoms (**27 a**, **28 a**, **29 a**) are less stable compared to the others due to unfavorable lone pair interactions.

High barriers (>16 kcal mol^−1^) for the cycloaddition were found for the reactions leading to isomers **23 a**, **24 a**, *trans*‐**25 a**, *cis*‐**25 a**, **27 a**, and **28 a**, smaller barriers (<15 kcal mol^−1^) for **11 a** (for structure see Scheme 7, right), *trans*‐**26 a**, *cis*‐**26 a**, and **29 a**. Low barriers (<25 kcal mol^−1^) for cycloreversion were only obtained for isomers *cis*‐**26 a**, *trans*‐**26 a**, and **29 a**. Among the three thermodynamically most stable isomers (**11 a**, **23 a**, and **24 a**) the experimentally found and characterized isomer **11 a** shows by far the lowest barrier for the cycloaddition reaction. Consequently, we interpret its preferred formation as a kinetically controlled process, leading to the thermodynamically third‐best isomer and confirming the role of the thiocarbonyl moiety as superdienophile. Other isomers may possibly not be formed under the reaction conditions (0 °C) or are subject of rapid equilibration of less stable cycloadducts. The calculations also suggest that under thermodynamic control the formation of the most stable 1,2‐oxazine derivatives of type **23** should be possible. Experimentally, an equilibration of **11** and the precursors **1** and **10** could not be proved due its limited stability.

## Conclusions

A series of thiochalcones **1** was smoothly prepared by treatment of the corresponding chalcones **12** with Lawesson's reagent. As shown by NMR spectroscopy, the generated monomeric products **1** are in equilibrium with their dimers **2** (3,4‐dihydro‐1,2‐dithiins) and **4** (3,4‐dihydro‐2*H*‐thiopyrans), whereas the conceivable regioisomeric dimers **3** and **5** are not observed. Monomers such as **1 j**–**l** with electron‐rich aryl groups Ar^1^ give mainly or exclusively the dimers *endo*‐**4 j**–**l**. High‐level DFT calculations with the parent compound **1 a** indicate that the dimerization process is kinetically controlled. Low barriers allow for the formation of *endo*‐**4 a** and *cis*/*trans*‐**2 a**, whereas the most stable dimer *exo*‐**4 a** is not formed due to a slightly higher barrier.

The monomers **1** can be successfully trapped by hetero‐Diels–Alder reactions with in situ generated nitrosoalkenes such as α‐nitrosostyrene **10 a**. In these [4+2]‐cycloadditions the thioketone moieties of thiochalcones **1** function as superdienophiles with exclusive formation of styryl‐substituted 4*H*‐1,5,2‐oxathiazines **11**. The cycloaddition hence proceeds with high periselectivity and regioselectivity. Closer inspection of crude product mixtures of the reactions shows that, depending on the thiochalcone substitution pattern, small or considerable amounts of the bis‐heterocyclic [4+2]‐cycloadducts **14** are formed, which derive from *endo*‐**4** dimers. In particular, with electron‐rich groups Ar^1^, where the corresponding monomers **1** are less favored in the equilibria, compounds **14** are found to be the major products. Again, the hetero‐Diels–Alder reaction only involves the thioketone moiety of *endo*‐**4**.

The DFT calculations show that the cycloaddition of α‐nitrosostyrene **10 a** to the C=C double bond of **1 a** should lead to the most stable, but not formed products **23 a** and **24 a**, followed by experimentally observed **11 a**, and those arising from the cycloaddition of thiochalcone **1 a** and the C=C double bond of **10 a** (**25 a**, **26 a**). By far least stable are the cycloadducts resulting from **1 a** and the N=O double bond of **10 a** (**28 a**, **29 a**). Like the dimerization reactions of thiochalcone **1 a**, the hetero‐Diels–Alder reaction of **1 a** with **10 a** to **11 a** is also a kinetically controlled process. The thermodynamically most stable [4+2]‐cycloadduct **23 a** that would originate from a reaction of the thiochalcone C=C double bond with the nitrosoalkene is apparently not formed due the high barrier of this cycloaddition.

Overall, the so far unstudied thiochalcone/α‐nitrosoalkene cycloadditions are identified as unique prototypes of periselective and regioselective processes. Only one of the eight possible constitutional isomers is observed—if the conceivable four [3+2]‐cycloadditions are also considered the high periselectivity is even more intriguing.

## Experimental Section

For general information, all experimental and analytical details and computational details see the Supporting Information.

**General procedure for reactions of the in situ generated α‐nitrosoalkenes with “thiochalcone fractions”**: To a solution of the corresponding α‐halooxime **13** (2.00 mmol) in dry dichloromethane (6 mL), an excess of solid potassium carbonate (2.76 g, 20.0 mmol) was added. To the resulting suspension, a freshly prepared solution of the corresponding “thiochalcone fraction” **2**/**4** (1.00 mmol) in dry dichloromethane (2 mL) was added dropwise at room temperature, and stirring was continued until the characteristic color of the starting thiocarbonyl precursors faded. After completion of the reaction (confirmed by TLC) the precipitated inorganic materials were filtered off, washed with dichloromethane (2×4 mL), and the solvents were removed under reduced pressure. In all experiments the mass recovery was high (>90 %), and the ratio of products **11** and **14** identified in the crude mixtures and collected in Table 1 was established based on the registered ^1^H NMR spectra. The residue obtained thereafter was purified by column chromatography (SiO_2_, petroleum ether/dichloromethane 7:3, gradient 1:1) and the product was recrystallized from a petroleum ether/dichloromethane mixture to give the corresponding 4*H*‐1,5,2‐oxathiazine derivative **11** and/or product **14** as crystalline materials.

**(*E*)‐3,6‐Diphenyl‐6‐styryl‐4*H*‐1,5,2‐oxathiazine (11 a)**:^22^ the product was obtained in improved yield by a modified general protocol, running the reaction at 0 °C for 20 h; yield: 161 mg (45 %); colorless crystals, m.p. 139–140 °C (decomp.). ^1^H NMR (CDCl_3_, 600 MHz): *δ*=3.42, 3.62 (AB system, *J=*17.4 Hz, 2 H, H_2_C(4)), 6.52, 6.75 (AB system, *J=*15.9 Hz, 2 H, 2=CH), 7.24–7.42, 7.60–7.63, 7.72–7.75 ppm (3 m, 11 H, 2 H, 2 H, Ph). For further characterization see Ref. 22.

**(*E*)‐6‐(4‐Bromostyryl)‐3,6‐diphenyl‐4*H*‐1,5,2‐oxathiazine (11 b)**: reaction time: 90 min; yield: 145 mg (33 %); colorless crystals, m.p. 196–198 °C (EtOAc) (decomp.). ^1^H NMR (CDCl_3_, 600 MHz): *δ*=3.40, 3.60 (AB system, *J=*17.5 Hz, 2 H, H_2_C(4)), 6.50, 6.67 (AB system, *J=*15.9 Hz, 2 H, 2=CH), 7.24–7.46, 7.59–7.62, 7.70–7.73 ppm (3 m, 10 H, 2 H, 2 H, Ph, Ar). ^13^C NMR (CDCl_3_, 151 MHz): *δ*=23.3 (t, C(4)), 86.7 (s, C(6)), 122.3 (s, CBr), 125.7, 126.9, 128.4, 128.66, 128.71*, 129.8, 129.9, 131.6, 131.8 (9 d, Ph, Ar,=CH), 134.6, 135.9, 139.8 (3 s, Ph, Ar), 152.3 ppm (s, C(3)); * signal with higher intensity. IR: $\tilde{\nu }$ =3053m, 3026m, 2914m, 1524m, 1476m, 1444m, 1368m, 1248m, 1245m, 1220m, 1197m, 951s, 918s, 836m, 758s, 745s, 711s, 693vs cm^−1^. MS (ESI): *m*/*z* (%)=437 (6, [M+H]^+^), 223 (48), 149 (100). Anal. Calcd for C_23_H_18_BrNOS (436.37): C 63.31, H 4.16, N 3.21, S 7.35; found: C 63.11, H 4.20, N 3.17, S 7.23.

**(*E*)‐3,6‐Diphenyl‐6‐(4‐methoxystyryl)‐4*H*‐1,5,2‐oxathiazine (11 c)**: reaction time: 60 min; yield: 110 mg (28 %); pale orange crystals, m.p. 159–160 °C (decomp.). ^1^H NMR (CDCl_3_, 600 MHz): *δ*=3.40, 3.61 (AB system, *J=*17.5 Hz, 2 H, H_2_C(4)), 3.80 (s, 3 H, OMe), 6.37, 6.66 (AB system, *J=*15.9 Hz, 2 H, 2=CH), 6.82–6.86, 7.32–7.42, 7.59–7.62, 7.70–7.73 ppm (4 m, 2 H, 8 H, 2 H, 2 H, Ph, Ar). ^13^C NMR (CDCl_3_, 151 MHz): *δ*=23.5 (t, C(4)), 55.3 (q, OMe), 87.0 (s, C(6)), 114.0, 125.7, 126.6, 126.9, 128.2 (5 d, Ar), 128.3 (s, Ar), 128.56, 128.60*, 129.8, 132.5 (4 d, Ar,=CH), 136.0, 140.1 (2 s, Ar), 151.9 (s, C(3)), 159.8 ppm (s, *C*OMe); *signal with higher intensity. IR: $\tilde{\nu }$ =3068m, 2931m, 2855m, 1605m, 1515s, 1455m, 1367s, 1193m, 1053m, 933s, 858s, 793vs, 698s cm^−1^. HRMS (ESI‐TOF): *m*/*z* [M+H]^+^ calcd for C_24_H_22_NO_2_S: 388.1371; found: 388.1373.

**(*E*)‐3,6‐Diphenyl‐6‐[2‐(naphth‐2‐yl)vinyl]‐4*H*‐1,5,2‐oxathiazine (11 d)**: reaction time: 60 min; yield: 150 mg (37 %); colorless crystals, m.p. 171–172 °C (decomp.). ^1^H NMR (CDCl_3_, 600 MHz): *δ*=3.43, 3.65 (AB system, *J=*17.5 Hz, 2 H, H_2_C(4)), 6.64, 6.90 (AB system, *J=*15.9 Hz, 2 H, 2=CH), 7.34–7.47, 7.59–7.64, 7.75–7.80 ppm (3 m, 8 H, 3 H, 6 H, Ar). ^13^C NMR (CDCl_3_, 151 MHz): *δ*=23.4 (t, C(4)), 87.0 (s, C(6)), 123.6, 125.7, 126.3, 126.4, 127.0, 127.5, 127.7, 128.1, 128.3, 128.6, 128.67, 128.68, 129.3, 129.8, 133.0 (15 d, Ar, =CH), 133.1, 133.3, 133.4, 136.0, 140.0 (5 s, Ar), 152.2 ppm (s, C(3)). IR: $\tilde{\nu }$ =3054m, 2914m, 1582m, 1489m, 1443m, 1392m, 1222m, 1097m, 955s, 916s, 887m, 752s, 745s, 708s, 693vs, 689vs cm^−1^. HRMS (ESI‐TOF): *m*/*z* [M+H]^+^ calcd for C_27_H_22_NOS: 408.1422; found: 408.1426.

**(*E*)‐3,6‐Diphenyl‐6‐[2‐(ferrocenyl)vinyl]‐4*H*‐1,5,2‐oxathiazine (11 e)**: reaction time: 24 h; yield: 250 mg (54 %); pale orange crystals, m.p. 155–156 °C (decomp.). ^1^H NMR (CDCl_3_, 600 MHz): *δ*=3.44, 3.46 (AB system, *J=*17.4 Hz, 2 H, H_2_C(4)), 4.05 (s, 5 H, Fc), 4.23–4.25 (m, 2 H, Fc), 4.34–4.37 (m, 2 H, Fc), 6.11, 6.50 (AB system, *J=*15.7 Hz, 2 H, 2=CH), 7.33–7.42, 7.64–7.66, 7.70–7.74 ppm (3 m, 6 H, 2 H, 2 H, Ph). ^13^C NMR (CDCl_3_, 151 MHz): *δ*=23.5 (t, C(4)), 67.1, 67.5, 69.2*, 69.3* (4 d, Fc), 81.1 (s, Fc), 87.0 (s, C(6)), 125.6, 125.9, 126.9, 128.54, 128.55, 128.6, 129.8, 131.9 (8 d, Ph,=CH), 136.0, 140.3 (2 s, Ph), 151.7 ppm (s, C(3)); *signal with higher intensity. IR: $\tilde{\nu }$ =3086m, 2888m, 1654m, 1588m, 1446m, 1399m, 1224m, 969s, 887m, 754s, 693vs cm^−1^. HRMS (ESI‐TOF): *m*/*z* [M+H]^+^ calcd for C_27_H_24_FeNOS: 466.0928; found: 466.0929.

**(*E*)‐3,6‐Diphenyl‐6‐[2‐(furan‐2‐yl)vinyl]‐4*H*‐1,5,2‐oxathiazine (11 f)**: reaction time: 60 min; yield: 145 mg (42 %); pale yellow crystals, m.p. 131–133 °C (decomp.). ^1^H NMR (CDCl_3_, 600 MHz): *δ*=3.41, 3.62 (AB system, *J=*17.4 Hz, 2 H, H_2_C(4)), 6.29 (d_br_, *J*≈3.3 Hz, 1 H, Fur), 6.36 (dd, *J=*1.8, 3.3 Hz, 1 H, Fur), 6.47, 6.52 (AB system, *J=*15.8 Hz, 2 H,=CH), 7.32–7.41, 7.60–7.62, 7.70–7.73 ppm (3 m, 7 H, 2 H, 2 H, Ph, Fur). ^13^C NMR (CDCl_3_, 151 MHz): *δ*=23.4 (t, C(4)), 86.6 (s, C(6)), 110.1, 111.5, 120.9, 125.7, 126.8, 127.3, 128.62, 128.63, 128.64, 129.8 (10 d, Ph, Fur,=CH), 136.0, 139.9 (2 s, Ph), 142.7 (d, Fur), 151.4 (s, C(3)), 152.1 ppm (s, Fur). IR: $\tilde{\nu }$ =2905m, 2887m, 1573m, 1485m, 1444m, 1388m, 1246m, 1228m, 1088m, 989s, 954s, 822m, 733s, 688vs, 679vs cm^−1^. MS (ESI): *m*/*z* (%)=370 (18, [M+Na]^+^), 348 (100, [M+H]^+^). Anal. Calcd for C_21_H_17_NO_2_S (347.43): C 72.60, H 4.93, N 4.03, S 9.23; found: C 72.67, H 5.04, N 4.06, S 9.30.

**(*E*)‐3,6‐Diphenyl‐6‐[2‐(thien‐2‐yl)vinyl]‐4*H*‐1,5,2‐oxathiazine (11 g)**: reaction time: 30 min; yield: 145 mg (40 %); pale yellow crystals, m.p. 151–152 °C (decomp.). ^1^H NMR (CDCl_3_, 600 MHz): *δ*=3.41, 3.61 (AB system, *J=*17.4 Hz, 2 H, H_2_C(4)), 6.35, 6.84 (AB system, *J=*15.7 Hz, 2 H,=CH), 6.95 (dd, *J=*3.6, 5.1 Hz, 1 H, Thie), 6.99 (d_br_, *J*≈3.6 Hz, 1 H, Thie), 7.20 (d_br_, *J*≈5.1 Hz, 1 H, Thie), 7.32–7.42, 7.60–7.62, 7.70–7.73 ppm (3 m, 6 H, 2 H, 2 H, Ph). ^13^C NMR (CDCl_3_, 151 MHz): *δ*=23.4 (t, C(4)), 86.7 (s, C(6)), 125.4, 125.8, 126.3, 126.9, 127.3, 127.5, 128.3, 128.64, 128.66, 128.69, 129.9 (11 d, Ph, Thie,=CH), 136.0, 139.9, 140.6 (3 s, Ph, Thie), 152.3 ppm (s, C(3)). IR: $\tilde{\nu }$ =2955m, 2927m, 2901m, 2847m, 1591m, 1493m, 1485m, 1444s, 1389m, 1276m, 1223m, 1197m, 1078m, 954s, 946s, 923s, 873m, 850m, 753s, 709s, 698vs, 689vs cm^−1^. MS (ESI): *m*/*z* (%)=386 (12, [M+Na]^+^), 364 (100, [M+H]^+^). Anal. Calcd for C_21_H_17_NOS_2_ (363.49): C 69.39, H 4.71, N 3.85, S 17.64; found: C 69.41, H 4.73, N 3.86, S 17.53.

**(*E*)‐6‐Ferrocenyl‐3‐phenyl‐6‐styryl‐4*H*‐1,5,2‐oxathiazine (11 h)**: reaction time: 24 h; yield: 240 mg (52 %); beige crystals, m.p. 81–82 °C. ^1^H NMR (CDCl_3_, 600 MHz): *δ*=3.60, 3.67 (AB system, *J=*17.3 Hz, 2 H, H_2_C(4)), 4.27 (m_c_, 2 H, Fc), 4.31 (s, 5 H, Fc), 4.40–4.42, 4.46–4.48 (2 m, 1 H each, Fc), 6.51, 6.76 (AB system, *J=*15.9 Hz, 2 H, 2=CH), 7.27–7.30, 7.34–7.42, 7.44–7.47, 7.64–7.68 ppm (4 m, 1 H, 5 H, 2 H, 2 H, Ph). ^13^C NMR (CDCl_3_, 151 MHz): *δ*=23.8 (t, C(4)), 66.9, 67.3, 68.5, 68.8, 69.4* (5 d, Fc), 84.8 (s, Fc), 88.6 (s, C(6)), 125.8, 126.8, 128.2, 128.3, 128.6, 128.7, 129.7, 131.2 (8 d, Ph,=CH), 135.9, 136.0 (2 s, Ph), 152.1 ppm (s, C(3)); * signal with higher intensity. IR: $\tilde{\nu }$ =3058m, 3026m, 2926m, 1578m, 1456m, 1444m, 1313m, 1221m, 967s, 906s, 818m, 725s, 689vs cm^−1^. HRMS (ESI‐TOF): *m*/*z* [M+H]^+^ calcd for C_27_H_24_FeNOS: 466.0928; found: 466.0929.

**(*E*)‐6‐Ferrocenyl‐3‐phenyl‐6‐[2‐(thien‐2‐yl)vinyl]‐4*H*‐1,5,2‐oxathiazine (11 i)**: reaction time: 24 h; yield: 260 mg (55 %); beige crystals, m.p. 102–104 °C. ^1^H NMR (CDCl_3_, 600 MHz): *δ*=3.58, 3.68 (AB system, *J=*17.3 Hz, 2 H, H_2_C(4)), 4.26 (m_c_, 2 H, Fc), 4.30 (s, 5 H, Fc), 4.40, 4.44 (2 s_br_, 1 H each, Fc), 6.37, 6.88 (AB system, *J=*15.6 Hz, 2 H,=CH), 6.98 (dd, *J=*3.5, 4.9 Hz, 1 H, Thie), 7.01 (d_br_, *J*≈3.5 Hz, 1 H, Thie), 7.21 (d_br_, *J*≈4.9 Hz, 1 H, Thie), 7.39–7.42, 7.65–7.68 ppm (2 m, 3 H, 2 H, Ph). ^13^C NMR (CDCl_3_, 151 MHz): *δ*=23.8 (t, C(4)), 66.9, 67.3, 68.6, 68.8, 69.4* (5 d, Fc), 84.5 (s, Fc), 88.4 (s, C(6)), 124.5, 124.9, 125.8, 127.0, 127.6, 127.9, 128.6, 129.8 (8 d, Ph, Thie,=CH), 136.0, 140.8 (2 s, Ph, Thie), 152.3 ppm (s, C(3)); *signal with higher intensity. IR: $\tilde{\nu }$ =3039m, 2957m, 2894m, 1493m, 1442m, 1378w, 1226m, 998s, 956s, 823m, 733s, 695s, 689vs cm^−1^. MS (ESI): *m*/*z* (%)=472 (100, [M+H]^+^). Anal. Calcd for C_25_H_21_FeNOS_2_ (471.41): C 63.69, H 4.49, N 2.97, S 13.60; found: C 63.87, H 4.73, N 2.84, S 13.60.

**Quantum chemical calculations**: Quantum chemical calculations (PBE1PBE/def2‐TZVP+PCM(dichloromethane)+GD3BJ dispersion correction)^26^, ^27^, ^28^, ^29^ were performed on the basis of preceding B3LYP/6‐31G(d)^30^+GD3BJ‐geometry optimizations using the Gaussian 09, Revision D.01^33^ and the Gaussian 16, Revision B.01^34^, package of programs. To obtain a most reliable structural information, several conformers of each isomer were investigated, in many cases after MM2‐conformational analysis. The transition‐state localizations were started with reaction‐path calculations by stepwise, independent elongation of both relevant bonds beginning with the cycloadducts (“retro‐Diels–Alder”) with full optimization of all other parameters. Then transition‐state searches or QST2 calculations on the basis of the obtained 3D‐hyperfaces followed. The *s‐cis*‐ as well as the s*‐trans*‐conformers of the reacting ene‐components were considered in the transition‐state searches; the respective energy lower isomeric transition states are represented in Scheme 6 and Scheme 10 and in the Supporting Information. We cannot exclude that due to the steric complexity of the reacting systems further transition‐state conformations and configurations exist. The explicit localization of bifurcations on the potential‐energy surfaces was beyond the scope of this experimentally oriented work⋅^35^ In many cases, IRC‐calculations were subsequently performed in order to characterize the respective stationary points.

## Conflict of interest

The authors declare no conflict of interest.

## Supporting information

As a service to our authors and readers, this journal provides supporting information supplied by the authors. Such materials are peer reviewed and may be re‐organized for online delivery, but are not copy‐edited or typeset. Technical support issues arising from supporting information (other than missing files) should be addressed to the authors.

Supplementary

## References

[1] For definitions of cycloadditions, see: R. Huisgen , Angew. Chem. Int. Ed. Engl. 1968, 7, 321–328;

[2] Cycloadditions in synthesis:

[2a] W. Carruthers , Cycloaddition Reactions in Organic Synthesis Pergamon Press, Oxford, 1990;

[2b] E. J. Corey , X.-M. Cheng , The Logic of Chemical Synthesis Wiley, New York, 1995;

[2c] S. Warren , P. Wyatt , Organic Synthesis: The Disconnection Approach, 2. Ed., Wiley, New York, 2008;

[2d] G. S. Zweifel , M. H. Nantz , P. Somfai , Modern Organic Synthesis: An Introduction, 2. Ed., Wiley, New York, 2017.

[3] For selected reviews on hetero-Diels–Alder reactions, see:

[3a] L. F. Tietze , G. Kettschau , Top. Curr. Chem. 1997, 189, 1–120;

[3b] H. Waldmann , Synthesis 1994, 535–551;

[3c] D. L. Boger , S. Weinreb , Hetero Diels–Alder Methodology in Organic Synthesis 2. Ed. Academic Press, 2012;

[3d] G. Blond , M. Gulea , V. Mamane , Curr. Org. Chem. 2016, 20, 2161–2210.

[4a] R. Huisgen , Proc. Chem. Soc. London 1961, 357–369;

[4b] R. Huisgen , Angew. Chem. Int. Ed. Engl. 1963, 2, 565–598;

[4c] R. Huisgen , Angew. Chem. Int. Ed. Engl. 1963, 2, 633–645;

[4d] 1,3-Dipolar Cycloaddition Chemistry (A. Padwa Ed.), Vol. 1 and 2, John Wiley & Sons, New York 1984;

[4e] K. V. Gothelf , K. A. Jørgensen , Chem. Rev. 1998, 98, 863–909.11848917

[5] R. B. Woodward , R. Hoffmann , Angew. Chem. Int. Ed. Engl. 1969, 8, 781–853;

[6] For a long-lasting discussion about diradicals as intermediates in 1,3-dipolar cycloadditions and Diels–Alder reactions, see: R. A. Firestone , J. Org. Chem. 1968, 33, 2285–2290, and subsequent publications of this author. For the refutation of his arguments, see:

[7] For examples of stepwise cycloadditions, see:

[7a] R. Huisgen , G. Mlostoń , E. Langhals , J. Am. Chem. Soc. 1986, 108, 6401–6402;

[7b] R. Huisgen , G. Mlostoń , E. Langhals , J. Org. Chem. 1986, 51, 4085–4087;

[7c] G. Mlostoń , E. Langhals , R. Huisgen , Tetrahedron Lett. 1989, 30, 5373–5376;

[7d] G. Mlostoń , R. Huisgen , H. Giera , Tetrahedron 2002, 58, 4185–4193; for nonconcerted Diels–Alder reactions with C=S dienophiles, see:

[7e] S. Wilker , G. Erker , J. Am. Chem. Soc. 1995, 117, 10922–10930; for a review on recent theoretical treatment of stepwise mechanisms in the [3+2]-cycloadditions, see:

[8a] For the first use of the term “perispecific”, see: K. N. Houk , L. J. Luskus , N. S. Bhacca , J. Am. Chem. Soc. 1970, 92, 6392–6394;

[8b] the term was later replaced by “periselectivity”, see: K. N. Houk , J. Sims , C. R. Watts , L. J. Luskus , J. Am. Chem. Soc. 1973, 95, 7301–7315;

[8c] A. Z. Bimanand , Y. N. Gupta , M. J. Doa , T. A. Eaton , K. N. Houk , F. R. Fronczek , J. Org. Chem. 1983, 48, 403–405.

[9] In general, the term “periselectivity” is applied to concerted reactions of π-systems that selectively undergo one of the symmetry allowed pathways available to the component. In most cases the distinguished reactions differ in the overall number of π-electrons involved (e.g. 4 π+2 π versus 4 π+6 π cycloaddition). If the π-electrons of reactions originate from different components, even if the total number of involved π-electrons is constant (e.g. 4 π+2 π versus 2 π+4 π cycloaddition) the term periselectivity should also be appropriate. To our opinion, the alternative term regioselectivity is not correct in this case and it should only be used for the different orientations of the components.

[10] For selected experimental and theoretical studies on periselective reactions, see:

[10a] A. A. Tishkov , I. M. Lyapkalo , S. L. Ioffe , Y. A. Strelenko , V. A. Tartakovsky , Org. Lett. 2000, 2, 1323–1324; 10810738 10.1021/ol0057885

[10b] A. G. Leach , K. N. Houk , Chem. Commun. 2002, 1243–1255; 10.1039/b111251c 12109102

[10c] A. G. Leach , K. N. Houk , I. W. Davies , Synthesis 2005, 3463–3467;

[10d] N. Çelebi-Ölçüm , D. H. Ess , V. Aviyente , K. N. Houk , J. Am. Chem. Soc. 2007, 129, 4528–4529; 17385868 10.1021/ja070686w

[10e] A. Skryńska , A. Albrecht , Ł. Albrecht , Adv. Synth. Catal. 2016, 358, 2838–2844;

[10f] R. Mose , G. Preegel , J. Larsen , S. Jakobsen , E. H. Iversen , K. A. Jørgensen , Nat. Chem. 2017, 9, 487–492; 28430196 10.1038/nchem.2682

[10g] P. Yu , T. Q. Chen , Z. Yang , C. Q. He , A. Patel , Y. Lam , C.-Y. Liu , K. N. Houk , J. Am. Chem. Soc. 2017, 139, 8251–8258; 28535677 10.1021/jacs.7b02966

[10h] P. Yu , C. Q. He , A. Simon , W. Li , R. Mose , M. K. Thøgersen , K. A. Jørgensen , K. N. Houk , J. Am. Chem. Soc. 2018, 140, 13726–13735; 30251535 10.1021/jacs.8b07575

[10i] X.-S. Xue , C. S. Jamieson , M. Garcia-Borràs , X. Dong , Z. Yang , K. N. Houk , J. Am. Chem. Soc. 2019, 141, 1217–1221. 30623652 10.1021/jacs.8b12674

[11] G. Mlostoń , P. Grzelak , R. Hamera-Fałdyga , M. Jasiński , P. Pipiak , K. Urbaniak , Ł. Albrecht , J. Hejmanowska , H. Heimgartner , Phosphorus Sulfur Silicon Relat. Elem. 2017, 192, 204–211.

[12a] R. Huisgen , L. Fišera , H. Giera , R. Sustmann , J. Am. Chem. Soc. 1995, 117, 9671–9678;

[12b] R. Huisgen , X. Li , H. Giera , E. Langhals , Helv. Chim. Acta 2001, 84, 981–999;

[12c] R. Huisgen , E. Langhals , Heteroat. Chem. 2006, 17, 433–442;

[12d] J. Breu , P. Höcht , U. Rohr , J. Schatz , J. Sauer , Eur. J. Org. Chem. 1998, 2861–2873;

[12e] U. Rohr , J. Schatz , J. Sauer , Eur. J. Org. Chem. 1998, 2875–2883.

[13a] J.-P. Pradere , G. Bouet , H. Quiniou , Tetrahedron Lett. 1972, 13, 3471–3474;

[13b] P. Beslin , D. Lagain , J. Vialle , C. Minot , Tetrahedron 1981, 37, 3839–3845;

[13c] T. Karakasa , S. Satsumabayashi , S. Motoki , Bull. Chem. Soc. Jpn. 1986, 59, 335–337;

[13d] S. Motoki , T. Saito , T. Karakasa , T. Matsushita , E. Furuno , J. Chem. Soc. Perkin Trans. 1 1992, 2943–2948;

[13e] G. M. Li , S. Niu , M. Segi , K. Tanaka , T. Nakajima , R. A. Zingaro , J. H. Reibenspies , M. B. Hall , J. Org. Chem. 2000, 65, 6601–6612; 11052108 10.1021/jo000740q

[13f] A. Capperucci , A. Degl'Innocenti , S. Biondi , T. Nocentini , G. Rinaudo , Tetrahedron Lett. 2003, 44, 2831–2835.

[14] H. Tanaka , S. Motoki , Bull. Chem. Soc. Jpn. 1986, 59, 2047–2049.

[15] J. Hejmanowska , M. Jasiński , J. Wojciechowski , G. Mlostoń , Ł. Albrecht , Chem. Commun. 2017, 53, 11472–11475. 10.1039/c7cc06518c 28984320

[16a] G. Mlostoń , P. Grzelak , H. Heimgartner , J. Sulfur Chem. 2017, 38, 1–10;

[16b] G. Mlostoń , R. Hamera-Fałdyga , H. Heimgartner , J. Sulfur Chem. 2018, 39, 322–331; 10.1039/c8ob01022f 29850725

[16c] G. Mlostoń , K. Urbaniak , P. Urbaniak , A. Marko , A. Linden , H. Heimgartner , Beilstein J. Org. Chem. 2018, 14, 1834–1839. 30112087 10.3762/bjoc.14.156 PMC6071716

[17a] R. Atir , S. Mallouk , K. Bougrin , M. Saufiaoui , Synth. Commun. 2006, 36, 111–120;

[17b] D. Rane , M. Sibi , Curr. Org. Synth. 2011, 8, 616–627;

[17c] Y. Zhang , X. Li , J. Li , J. Chen , X. Meng , M. Zhao , B. Chen , Org. Lett. 2012, 14, 26–29; 22133007 10.1021/ol202718d

[17d] H. M. T. Albuquerque , C. M. M. Santos , J. A. S. Cavaleiro , A. M. S. Silva , Curr. Org. Chem. 2014, 18, 2750–2775;

[17e] W. Yang , T. Miao , P. Li , L. Wang , RSC Adv. 2015, 5, 95833–95839;

[17f] B. Gayen , A. Banerji , K. Dhara , Synth. Commun. 2016, 46, 293–308.

[18a] P. Grzelak , G. Utecht , M. Jasiński , G. Mlostoń , Synthesis 2017, 49, 2129–2137;

[18b] For a recent report on the [3+2]-annulation of azaoxyallyl cations to thiochalcones, see: V. Jaiswal , B. Mondal , K. Singh , D. Das , J. Saha , Org. Lett. 2019, 21, 5848–5852. 31294998 10.1021/acs.orglett.9b01933

[19] Reviews:

[19a] T. L. Gilchrist , Chem. Soc. Rev. 1983, 12, 53–73;

[19b] I. M. Lyapkalo , S. L. Ioffe , Russ. Chem. Rev. 1998, 67, 467–484;

[19c] H.-U. Reissig , R. Zimmer , Science of Synthesis 2006, 33, 371–389 and in

[19d] S. M. M. Lopes , A. L. Cardoso , A. Lemos , T. M. V. D. Pinho e Melo , Chem. Rev. 2018, 118, 11324–11352; 30495939 10.1021/acs.chemrev.8b00375

[19e] S. V. Weinreb , Synlett 2019, 30, 1855–1866.

[20] L. R. Domingo , M. T. Picher , P. Arroyo , Eur. J. Org. Chem. 2006, 2570–2580. 10.1021/jo0613986 17137358

[21] B. F. Bonini , E. Foresti , G. Maccagnani , G. Mazzanti , P. Sabatino , P. Zani , Tetrahedron Lett. 1985, 26, 2131–2134.

[22] G. Mlostoń , K. Urbaniak , R. Zimmer , H.-U. Reissig , H. Heimgartner , ChemistrySelect 2018, 3, 11724–11728.

[23] Diagnostic signals attributed to 1,3-dithiin **3 a**: ^1^H NMR (600 MHz, CDCl_3_): *δ*=4.73 (d, *J=*2.5 Hz, HC(4)), 6.24 (d, *J=*2.5 Hz, HC(5)), 6.61, 6.85 ppm (AB system, *J=*15.5 Hz, 2 HC=).

[24a] C. Hippeli , H.-U. Reissig , Liebigs Ann. Chem. 1990, 217–226;

[24b] H.-U. Reissig , C. Hippeli , T. Arnold , Chem. Ber. 1990, 123, 2403–2411;

[24c] R. Zimmer , M. Collas , R. Czerwonka , U. Hain , H.-U. Reissig , Synthesis 2008, 237–244.

[25] I. Fleming Molecular Orbitals and Organic Chemical Reactions, J. Wiley & Sons Ltd., Chichester, 2010, pp. 355–362.

[26a] J. P. Perdew , K. Burke , M. Ernzerhof , Phys. Rev. Lett. 1996, 77, 3865–3868; 10062328 10.1103/PhysRevLett.77.3865

[26b] J. P. Perdew , K. Burke , M. Ernzerhof , Phys. Rev. Lett. 1997, 78, 1396; 10.1103/PhysRevLett.77.3865 10062328

[26c] C. Adamo , V. Barone , J. Chem. Phys. 1999, 110, 6158–6169;

[26d] M. Ernzerhof , G. E. Scuseria , J. Chem. Phys. 1999, 110, 5029–5036.

[27] F. Weigend , R. Ahlrichs , Phys. Chem. Chem. Phys. 2005, 7, 3297–3305. 16240044 10.1039/b508541a

[28] J. Tomasi , B. Mennucci , R. Cammi , Chem. Rev. 2005, 105, 2999–3093. 16092826 10.1021/cr9904009

[29a] S. Grimme , S. Ehrlich , L. Goerigk , J. Comput. Chem. 2011, 32, 1456–1465; 21370243 10.1002/jcc.21759

[29b] S. Grimme , A. Hansen , J. G. Brandenburg , C. Bannwarth , Chem. Rev. 2016, 116, 5105–5154. 27077966 10.1021/acs.chemrev.5b00533

[30a] A. D. Becke , J. Chem. Phys. 1993, 98, 5648–5652;

[30b] C. Lee , W. Yang , R. G. Parr , Phys. Rev. B 1988, 37, 785–789. 10.1103/physrevb.37.785 9944570

[31] The NMR spectra of the crude products obtained by reaction of **10 a** with simple thioketones or thiochalcones are very complex and show signals of a variety of unidentified side products. Therefore, we cannot rigorously exclude the formation of [3+2] cycloadducts.

[32] For typical examples of the [3+2]-cycloadditions of α-nitrosoalkenes with alkenes leading to cyclic nitrones, see Ref. [24c] and:

[32a] T. L. Gilchrist , G. M. Iskander , A. K. Yagoub , J. Chem. Soc. Chem. Commun. 1981, 696–698;

[32b] R. Zimmer , M. Collas , M. Roth , H.-U. Reissig , Liebigs Ann. Chem. 1992, 709–714;

[32c] J. M. de los Santos , R. Ignacio , D. Aparicio , F. Palacios , J. M. Ezpeleta , J. Org. Chem. 2009, 74, 3444–3448; 19344164 10.1021/jo900489j

[32d] S. C. C. Nunes , S. M. M. Lopes , C. S. B. Gomes , A. Lemos , A. A. C. C. Pais , T. M. V. D. Pinho e Melo , J. Org. Chem. 2014, 79, 10456–10465. 25310009 10.1021/jo502095k

[33] Gaussian 09, Revision D.01, M. J. Frisch, G. W. Trucks, H. B. Schlegel, G. E. Scuseria, M. A. Robb, J. R. Cheeseman, G. Scalmani, V. Barone, B. Mennucci, G. A. Petersson, H. Nakatsuji, M. Caricato, X. Li, H. P. Hratchian, A. F. Izmaylov, J. Bloino, G. Zheng, J. L. Sonnenberg, M. Hada, M. Ehara, K. Toyota, R. Fukuda, J. Hasegawa, M. Ishida, T. Nakajima, Y. Honda, O. Kitao, H. Nakai, T. Vreven, J. A. Montgomery, Jr., J. E. Peralta, F. Ogliaro, M. Bearpark, J. J. Heyd, E. Brothers, K. N. Kudin, V. N. Staroverov, R. Kobayashi, J. Normand, K. Raghavachari, A. Rendell, J. C. Burant, S. S. Iyengar, J. Tomasi, M. Cossi, N. Rega, N. J. Millam, M. Klene, J. E. Knox, J. B. Cross, V. Bakken, C. Adamo, J. Jaramillo, R. Gomperts, R. E. Stratmann, O. Yazyev, A. J. Austin, R. Cammi, C. Pomelli, J. W. Ochterski, R. L. Martin, K. Morokuma, V. G. Zakrzewski, G. A. Voth, P. Salvador, J. J. Dannenberg, S. Dapprich, A. D. Daniels, Ö. Farkas, J. B. Foresman, J. V. Ortiz, J. Cioslowski, D. J. Fox, Gaussian, Inc., Wallingford CT, **2009**.

[34] Gaussian 16, Revision B.01, M. J. Frisch, G. W. Trucks, H. B. Schlegel, G. E. Scuseria, M. A. Robb, J. R. Cheeseman, G. Scalmani, V. Barone, G. A. Petersson, H. Nakatsuji, X. Li, M. Caricato, A. V. Marenich, J. Bloino, B. G. Janesko, R. Gomperts, B. Mennucci, H. P. Hratchian, J. V. Ortiz, A. F. Izmaylov, J. L. Sonnenberg, D. Williams-Young, F. Ding, F. Lipparini, F. Egidi, J. Goings, B. Peng, A. Petrone, T. Henderson, D. Ranasinghe, V. G. Zakrzewski, J. Gao, N. Rega, G. Zheng, W. Liang, M. Hada, M. Ehara, K. Toyota, R. Fukuda, J. Hasegawa, M. Ishida, T. Nakajima, Y. Honda, O. Kitao, H. Nakai, T. Vreven, K. Throssell, J. A. Montgomery, Jr., J. E. Peralta, F. Ogliaro, M. J. Bearpark, J. J. Heyd, E. N. Brothers, K. N. Kudin, V. N. Staroverov, T. A. Keith, R. Kobayashi, J. Normand, K. Raghavachari, A. P. Rendell, J. C. Burant, S. S. Iyengar, J. Tomasi, M. Cossi, J. M. Millam, M. Klene, C. Adamo, R. Cammi, J. W. Ochterski, R. L. Martin, K. Morokuma, O. Farkas, J. B. Foresman, and D. J. Fox, Gaussian, Inc., Wallingford CT, **2016**.

[35a] P. Caramella , P. Quadrelli , L. Toma , J. Am. Chem. Soc. 2002, 124, 1130–1131; 11841256 10.1021/ja016622h

[35b] D. H. Ess , S. E. Wheeler , R. G. Iafe , L. Xu , N. Çelebi-Ölçüm , K. N. Houk , Angew. Chem. Int. Ed. 2008, 47, 7592–7601; 10.1002/anie.200800918 PMC2790825 18767086

[35c] Z. Yang , L. Zou , Y. Yu , F. Liu , X. Dong , K. N. Houk , Chem. Phys. 2018, 514, 120–125.

