# Supplementary material for: The [4+2]‐Cycloaddition of α‐Nitrosoalkenes with Thiochalcones as a Prototype of Periselective Hetero‐Diels–Alder Reactions—Experimental and Computational Studies
Source: Chemistry. 2019 Nov 22;26(1):237–48. doi: 10.1002/chem.201903385 (PMC6973135; doi:10.1002/chem.201903385)
Supplement: Supplementary file 1 — Supplementary [file CHEM-26-237-s001.pdf]

CHEMISTRY
A European Journal

Supporting Information

The [4+2]-Cycloaddition of α-Nitrosoalkenes with Thiochalcones as a Prototype of Periselective Hetero-Diels–Alder Reactions—Experimental and Computational Studies

Grzegorz Mlostoń,*[a] Katarzyna Urbaniak,[a] Marcin Jasiński,*[a] Ernst-Ulrich Würthwein,*[b] Heinz Heimgartner,[c] Reinhold Zimmer,[d] and Hans-Ulrich Reissig*[d]

chem_201903385_sm_miscellaneous_information.pdf

Supporting Information

Table of Content:

1. Synthetic details 1

2. References synthetic part 13

3. Copies of NMR spectra 14

4. DFT Calculations 47

5. References theoretical part 91

1. Synthetic details

General remarks

Reagents were purchased (Sigma-Aldrich, Acros) and used as received without further purification. All solvents were dried over appropriate drying agents and distilled before use. Products were purified by flash chromatography on silica gel (230–400 mesh, Merck or Fluka). Reported yields refer to analytically pure samples. NMR spectra were recorded with a Bruker AVIII 600 instrument. Chemical shifts are reported relative to solvent residual peaks (^{1}H NMR: δ = 7.26 ppm [$CHCl_3$]; ^{13}C NMR: δ = 77.0 ppm [$CDCl_3$]). Integrals are in accordance with assignments and coupling constants *J* are given in Hz. All ^{13}C-NMR spectra are proton-decoupled. For detailed peak assignments 2D spectra were measured (COSY, HMQC, HMBC). IR spectra were measured with an Agilent Cary 630 FTIR spectrometer. Mass spectra were performed with a Varian 500-MS LC Ion Trap or with a Synapt G2-Si mass spectrometer (Waters). Elemental analyses were obtained with a Vario EL III (Elementar Analysensysteme GmbH) instrument. Melting points were determined in capillaries with a MEL-TEMP II apparatus (Aldrich) and are uncorrected. If not stated otherwise, reactions were carried out under argon in a flame-dried flask with addition of the reactants by using a syringe; subsequent manipulations were conducted in air.

Starting materials

Thionation of chalcones **12a**–**12l** was performed following literature procedures by using a slight excess of Lawesson's reagent (1.05 equiv.) to give '*thiochalcone fractions*' as solid materials.[1-5] In most cases, the ^{1}H-NMR spectra (in $CDCl_3$) of the pre-purified products (SiO_2, petroleum ether/dichloromethane mixtures) indicated the presence of a mixture of dimers **2** and **4** as major components of the resulting '*thiochalcone fractions*', which was used for the next step without additional purification.

In the case of chalcones **12j**–**12l**, the thionation reaction provided the corresponding 3,4-dihydro-2*H*-thiopyran derivatives **4j**–**4l** as major components. Spectroscopically pure samples of the latter products were obtained by additional chromatographic purification (SiO_2, petroleum ether/dichloromethane 2:1 gradient to 1:2).

α-Halooximes **13a**–**13c** were prepared according to the literature.[6-8]

Thiochalcone Dimers

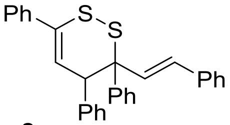

2a

3-Styryl-3,4,6-triphenyl-3,4-dihydro-1,2-dithiin (**2a**; ca. 1:1 mixture of *cis*- and *trans*-isomers contaminated with small amounts of *endo*-**4a**): In analogy to an earlier report,[9] a sample of **2a** (1:1 mixture) was obtained by treatment of the freshly prepared '*thiochalcone fraction*' with petroleum ether, followed by recrystallization of the resulting colorless precipitate from benzene/Et_2O mixture.

^{1}H NMR ($CDCl_3$, 600 MHz): δ = 4.10 (d, J = 5.4 Hz, 1 H, HC(4)), 4.57 (d, J = 4.4 Hz, 1 H, HC(4), *isomer*), 6.05, 6.37 (AB system, J = 16.2 Hz, 2 H, 2 =CH, *both isomers*), 6.49 (d, J = 5.4 Hz, 1 H, HC(5)), 6.52 (d, J = 4.4 Hz, 1 H, HC(5), *isomer*), 6.58, 6.79 (AB system, J = 16.3 Hz, 2 H, 2 =CH, *both isomers*), 7.00–7.41, 7.48–7.54 (2 m, 32 H, 4 H, Ph, *both isomers*), 7.55–7.59 (m, 2 H, Ph), 7.71–7.65 (m, 2 H, Ph, *isomer*) ppm. ^{13}C NMR ($CDCl_3$, 151 MHz): δ = 50.3 (d, C(4)), 51.0 (d, C(4), *isomer*), 57.3 (s, C(3), *isomer*), 58.8 (s, C(3)), 126.4, 126.5, 126.6, 126.9, 126.96, 127.03, 127.1*, 127.23, 127.25, 127.5, 127.56*, 127.71, 127.74, 127.87, 127.90, 128.47, 128.51, 128.55, 128.58*, 128.65, 128.9 (21 d, Ph, C(5), *both isomers*), 130.5 (d, C(2')), 131.0 (d, C(1'), *isomer*), 131.1, 131.3 (2 d, Ph, *both isomers*), 132.2 (d, C(1')), 133.0 (d, C(2'), *isomer*), 135.3, 135.9, 136.7, 136.9, 139.0, 139.2, 140.1, 140.3, 141.3, 141.8 (10 s, Ph, C(6), *both isomers*) ppm; *signal with higher intensity.

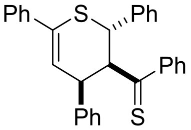

endo-**4a**

(*trans,cis*)-2,4,6-Triphenyl-3-(phenylthiocarbonyl)-3,4-dihydro-2*H*-thiopyran (*endo*-**4a**; as a mixture with **2a**): obtained by dissolving the above isolated sample of **2a** in $CDCl_3$ and storage overnight at room temperature (NMR tube experiment).

^{1}H NMR ($CDCl_3$, 600 MHz): δ = 4.08 (dd, J = 4.2, 6.6 Hz, 1 H, HC(4)), 5.07 (d, J = 11.2 Hz, 1 H, HC(2)), 5.30 (dd, J = 4.2, 11.2 Hz, 1 H, HC(3)), 6.37 (d, J = 6.6 Hz, 1 H, HC(5)), 7.06–7.63, 7.89–7.92 (2 m, 18 H, 2 H, Ph) ppm. ^{13}C NMR ($CDCl_3$, 151 MHz): δ = 45.9 (d, C(2)), 46.6 (d, C(4)), 59.3 (d, C(3)), 120.6 (d, C(5)), 126.3, 126.4, 127.34, 127.35, 127.6, 128.3, 128.4, 128.5, 128.7, 129.2, 130.2, 132.2 (12 d, Ph), 135.6, 138.8, 138.9, 139.5, 146.4 (5 s, Ph, C(6)), 239.4 (s, C=S) ppm.

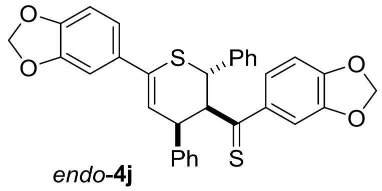

endo-**4j**

(*trans,cis*)-2,4-Diphenyl-6-(3,4-methylenedioxyphenyl)-3-[(3,4-methylenedioxyphenyl)thio-carbonyl]-3,4-dihydro-2*H*-thiopyran (*endo*-**4j**): violet solid, m. p. 83–84 °C; yield: 61%.

^{1}H NMR ($CDCl_3$, 600 MHz): δ = 4.04 (dd, *J* = 4.2, 6.5 Hz, 1 H, HC(4)), 5.02 (d, *J* = 11.1 Hz, 1 H, HC(2)), 5.17 (dd, *J* = 4.2, 11.1 Hz, 1 H, HC(3)), 5.96, 5.97 (AB system, *J* = 1.5 Hz, 2 H, OCH_2O), 6.04, 6.06 (AB system, *J* = 1.4 Hz, 2 H, OCH_2O), 6.24 (d, *J* = 6.5 Hz, 1 H, HC(5)), 6.79 (d, *J* = 8.1 Hz, 1 H, Ar), 6.86 (d, *J* = 8.3 Hz, 1 H, Ar), 7.01–7.05, 7.08–7.12, 7.14–7.18, 7.21–7.27 (4 m, 2 H, 3 H, 2 H, 5 H, Ar), 7.47 (d_{br}, *J* ≈ 1.8 Hz, 1 H, Ar), 7.66 (dd, *J* = 1.8, 8.3 Hz, 1 H, Ar) ppm. ^{13}C NMR ($CDCl_3$, 151 MHz): δ = 46.0 (d, C(2)), 47.1 (d, C(4)), 58.4 (d, C(3)), 101.2, 102.0 (2 t, 2 OCH_2O), 107.0, 107.8, 107.9, 108.2, 119.9 (5 d, Ar), 120.2 (d, C(5)), 121.5 (d, Ar), 127.3*, 127.5, 128.3, 129.1, 130.1 (5 d, 2 Ar), 133.1, 135.2, 139.1, 139.6, 141.2, 147.74, 147.75, 148.6, 151.8 (9 s, C(6), Ar), 235.0 (s, C=S) ppm; *signal with higher intensity. IR: ν = 1600*m*, 1482*s*, 1433*m*, 1241*s*, 1101*m*, 1036*s*, 932*m*, 807*m*, 695*s* cm^{-1}. MS (ESI): *m/z* (%) = 575 (17, [M + K]$^+$), 559 (10, [M + Na]$^+$), 537 (22, [M + H]$^+$), 415 (63), 307 (34), 269 (100, [$^1/_2$M + H]$^+$). Anal. Calcd for $C_{32}H_{24}O_4S_2$ (536.66): C 71.62, H 4.51, S 11.95; found: C 71.79, H 4.78, S 11.88.

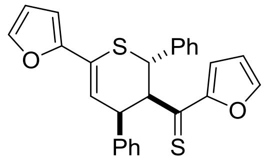

endo-**4k**

(*trans,cis*)-2,4-Diphenyl-6-(furan-2-yl)-3-[(furan-2-yl)thiocarbonyl]-3,4-dihydro-2*H*-thiopyran (*endo*-**4k**): green solid, m. p. 110–111 °C; yield: 68%.

^{1}H NMR ($CDCl_3$, 600 MHz): δ = 4.19 (dd, *J* = 4.4, 6.6 Hz, 1 H, H(C4)), 5.02 (d, *J* = 11.3 Hz, 1 H, HC(2)), 5.23 (dd, *J* = 4.4, 11.3 Hz, 1 H, HC(3)), 6.42 (dd, *J* = 1.9, 3.4 Hz, 1 H, Fur), 6.47 (d, *J* = 3.4 Hz, 1 H, Fur), 6.55 (s_{br}, 1 H, Fur), 6.65 (d, *J* = 6.6 Hz, 1 H, HC(5)), 7.04–7.07, 7.11–7.28 (2 m, 2 H, 9 H, Fur, Ph), 7.39 (d_{br}, *J* ≈ 1.9 Hz, 1 H, Fur), 7.69 (s_{br}, 1 H, Fur) ppm. ^{13}C NMR ($CDCl_3$, 151 MHz): δ = 44.1 (d, C(2)), 46.5 (d, C(4)), 56.8 (d, C(3)), 106.4, 111.3, 114.0, 115.6 (4 d, Fur), 118.5 (d, C(5)), 125.1 (s, C(6)), 127.3, 127.5, 127.6, 128.3, 129.1, 130.3 (6 d, Fur, Ph), 138.8, 139.0 (2 s, Ph), 142.1, 147.2 (2 d, Fur), 150.8, 160.9 (2 s, Fur), 217.3 (s, C=S) ppm. IR: ν = 1545*w*, 1448*m*, 1381*m*, 1146*m*, 1077*m*, 1015*s*, 962*m*, 837*s*, 714*s*, 694*s* cm^{-1}. MS (ESI): *m/z* (%) = 451 (8, [M + Na]$^+$), 307 (15), 215

(100,

[$^{1}/_{2}$M + H]$^+$), 181 (42). Anal. Calcd for $C_{26}H_{20}O_2S_2$ (428.56): C 72.87, H 4.70, S 14.96; found: C 72.77, H 4.98, S 14.68.

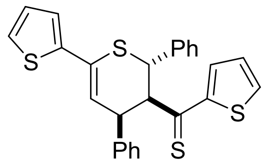

endo-**4l**

(*trans,cis*)-2,4-Diphenyl-6-(thien-2-yl)-3-[(thien-2-yl)thiocarbonyl]-3,4-dihydro-2*H*-thiopyran (*endo*-**4l**): green solid, m. p. 78–79 °C; yield: 71%.

^{1}H NMR ($CDCl_3$, 600 MHz): δ = 4.18 (dd, *J* = 4.4, 6.6 Hz, 1 H, HC(4)), 5.01 (d, *J* = 11.1 Hz, 1 H, HC(2)), 5.14 (dd, *J* = 4.4, 11.1 Hz, 1 H, HC(3)), 6.46 (d, *J* = 6.6 Hz, 1 H, HC(5)), 7.01 (dd, *J* = 3.7, 5.1 Hz, 1 H, Thie), 7.06–7.29 (m, 13 H, Thie, Ph), 7.70 (dd_{br}, *J* ≈ 1.0, 5.1 Hz, 1 H, Thie), 7.79 (s_{br}, 1 H, Thie) ppm. ^{13}C NMR ($CDCl_3$, 151 MHz): δ = 45.4 (d, C(2)), 47.8 (d, C(4)), 58.5 (d, C(3)), 119.8 (d, C(5)), 124.0, 124.7, 125.5, 127.4, 127.5, 127.6, 127.7, 128.4, 129.0 (9 d, Thie, Ph), 129.1 (s, C(6)), 129.2, 130.2, 137.3 (3 d, Thie, Ph), 138.8, 138.9, 141.6, 155.8 (4 s, Thie, Ph), 224.0 (s, C=S) ppm. IR: ν = 1491*m*, 1452*m*, 1405*s*, 1353*s*, 1264*m*, 1059*m*, 904*s*, 814*m*, 721*s*, 693*s* cm^{-1}. MS (ESI): *m*/*z* (%) = 461 (5, [M + H]$^+$), 339 (14), 293 (52), 231 (100, [$^{1}/_{2}$M + H]$^+$), 197 (43). Anal. Calcd for $C_{26}H_{20}S_4$ (460.68): C 67.79, H 4.38, S 27.84; found: C 67.53, H 4.58, S 27.71.

Reactions of the in situ-generated α-nitrosoalkenes 10 with ´*thiochalcone fractions*´ 2/4 – general procedure

To a solution of the corresponding α-halooxime **13** (2.00 mmol) in dry dichloromethane (2 mL), an excess of solid potassium carbonate (2.76 g, 20.0 mmol) was added. To the resulting suspension, a freshly prepared solution of the corresponding *´thiochalcone fraction´* **2**/**4** (1.00 mmol) in dry dichloromethane (2 mL) was added dropwise at room temperature, and stirring was continued until the characteristic color of the starting thiocarbonyl precursors faded. After completion of the reaction (confirmed by TLC) the precipitated inorganic materials were filtered off, washed with dichloromethane (2 × 4 mL), and the solvents were removed under reduced pressure. In all experiments the mass recovery was high (>90%), and the ratio of products **11** and **14** identified in the crude mixtures and collected in Table 1 was established based on the registered ^{1}H NMR spectra. The residue obtained thereafter was purified by column chromatography (SiO_2, petroleum ether/dichloromethane 7:3, gradient 1:1) and the product was recrystallized from a petroleum

ether/dichloromethane mixture to give the corresponding 4*H*-1,5,2-oxathiazine derivative **11** and/or product **14** as crystalline materials.

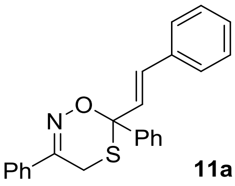

11a

(*E*)-3,6-Diphenyl-6-styryl-4*H*-1,5,2-oxathiazine (11a):[10] the product was obtained in improved yield by a modified general protocol, running the reaction at 0 °C for 20 h; yield: 161 mg (45%); colorless crystals, m. p. 139–140 °C (decomp.).

^{1}H NMR ($CDCl_3$, 600 MHz): δ = 3.42, 3.62 (AB system, *J* = 17.4 Hz, 2 H, $H_2C(4)$), 6.52, 6.75 (AB system, *J* = 15.9 Hz, 2 H, 2 =CH), 7.24–7.42, 7.60–7.63, 7.72–7.75 (3 m, 11 H, 2 H, 2 H, Ph) ppm. For further characterization see ref. 10.

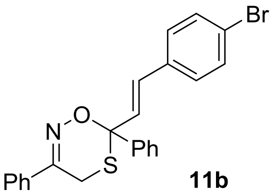

11b

(*E*)-6-(4-Bromostyryl)-3,6-diphenyl-4*H*-1,5,2-oxathiazine (11b): reaction time: 90 min; yield: 145 mg (33%); colorless crystals, m. p. 196–198 °C (EtOAc) (decomp.).

^{1}H NMR ($CDCl_3$, 600 MHz): δ = 3.40, 3.60 (AB system, *J* = 17.5 Hz, 2 H, $H_2C(4)$), 6.50, 6.67 (AB system, *J* = 15.9 Hz, 2 H, 2 =CH), 7.24–7.46, 7.59–7.62, 7.70–7.73 (3 m, 10 H, 2 H, 2 H, Ph, Ar) ppm. ^{13}C NMR ($CDCl_3$, 151 MHz): δ = 23.3 (t, C(4)), 86.7 (s, C(6)), 122.3 (s, CBr), 125.7, 126.9, 128.4, 128.66, 128.71*, 129.8, 129.9, 131.6, 131.8 (9 d, Ph, Ar, =CH), 134.6, 135.9, 139.8 (3 s, Ph, Ar), 152.3 (s, C(3)) ppm; * signal with higher intensity. IR: ν = 3053*m*, 3026*m*, 2914*m*, 1524*m*, 1476*m*, 1444*m*, 1368*m*, 1248*m*, 1245*m*, 1220*m*, 1197*m*, 951*s*, 918*s*, 836*m*, 758*s*, 745*s*, 711*s*, 693*vs* cm^{-1}. MS (ESI): *m*/*z* (%) = 437 (6, $[M + H]^+$), 223 (48), 149 (100). Anal. Calcd for $C_{23}H_{18}BrNOS$ (436.37): C 63.31, H 4.16, N 3.21, S 7.35; found: C 63.11, H 4.20, N 3.17, S 7.23.

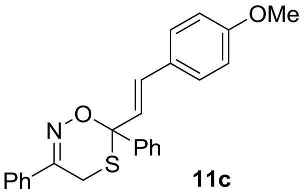

11c

(*E*)-3,6-Diphenyl-6-(4-methoxystyryl)-4*H*-1,5,2-oxathiazine (11c): reaction time: 60 min; yield: 110 mg (28%); pale orange crystals, m. p. 159–160 °C (decomp.). ^{1}H NMR ($CDCl_3$, 600 MHz): δ = 3.40, 3.61 (AB system, J = 17.5 Hz, 2 H, $H_2C(4)$), 3.80 (s, 3 H, OMe), 6.37, 6.66 (AB system, J = 15.9 Hz, 2 H, 2 =CH), 6.82–6.86, 7.32–7.42, 7.59–7.62, 7.70–7.73 (4 m, 2 H, 8 H, 2 H, 2 H, Ph, Ar) ppm. ^{13}C NMR ($CDCl_3$, 151 MHz): δ = 23.5 (t, C(4)), 55.3 (q, OMe), 87.0 (s, C(6)), 114.0, 125.7, 126.6, 126.9, 128.2 (5 d, Ar), 128.3 (s, Ar), 128.56, 128.60*, 129.8, 132.5 (4 d, Ar, =CH), 136.0, 140.1 (2 s, Ar), 151.9 (s, C(3)), 159.8 (s, *C*OMe) ppm; *signal with higher intensity. IR: ν = 3068*m*, 2931*m*, 2855*m*, 1605*m*, 1515*s*, 1455*m*, 1367*s*, 1193*m*, 1053*m*, 933*s*, 858*s*, 793*vs*, 698*s* cm^{-1}. HRMS (ESI-TOF): *m*/*z* $[M + H]^+$ calcd for $C_{24}H_{22}NO_2S$: 388.1371; found: 388.1373.

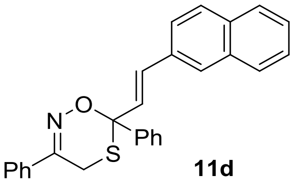

11d

(*E*)-3,6-Diphenyl-6-[2-(naphth-2-yl)vinyl]-4*H*-1,5,2-oxathiazine (11d): reaction time: 60 min; yield: 150 mg (37%); colorless crystals, m. p. 171–172 °C (decomp.).

^{1}H NMR ($CDCl_3$, 600 MHz): δ = 3.43, 3.65 (AB system, J = 17.5 Hz, 2 H, $H_2C(4)$), 6.64, 6.90 (AB system, J = 15.9 Hz, 2 H, 2 =CH), 7.34–7.47, 7.59–7.64, 7.75–7.80 (3 m, 8 H, 3 H, 6 H, Ar) ppm. ^{13}C NMR ($CDCl_3$, 151 MHz): δ = 23.4 (t, C(4)), 87.0 (s, C(6)), 123.6, 125.7, 126.3, 126.4, 127.0, 127.5, 127.7, 128.1, 128.3, 128.6, 128.67, 128.68, 129.3, 129.8, 133.0 (15 d, Ar, =CH), 133.1, 133.3, 133.4, 136.0, 140.0 (5 s, Ar), 152.2 (s, C(3)) ppm. IR: ν = 3054*m*, 2914*m*, 1582*m*, 1489*m*, 1443*m*, 1392*m*, 1222*m*, 1097*m*, 955*s*, 916*s*, 887*m*, 752*s*, 745*s*, 708*s*, 693*vs*, 689*vs* cm^{-1}. HRMS (ESI-TOF): *m*/*z* $[M + H]^+$ calcd for $C_{27}H_{22}NOS$: 408.1422; found: 408.1426.

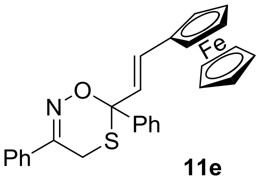

11e

(*E*)-3,6-Diphenyl-6-[2-(ferrocenyl)vinyl]-4*H*-1,5,2-oxathiazine (11e): reaction time: 24 h; yield: 250 mg (54%); pale orange crystals, m. p. 155–156 °C (decomp.).

^{1}H NMR ($CDCl_3$, 600 MHz): δ = 3.44, 3.46 (AB system, *J* = 17.4 Hz, 2 H, $H_2C(4)$), 4.05 (s, 5 H, Fc), 4.23–4.25 (m, 2 H, Fc), 4.34–4.37 (m, 2 H, Fc), 6.11, 6.50 (AB system, *J* = 15.7 Hz, 2 H, 2 =CH), 7.33–7.42, 7.64–7.66, 7.70–7.74 (3 m, 6 H, 2 H, 2 H, Ph) ppm. ^{13}C NMR ($CDCl_3$, 151 MHz): δ = 23.5 (t, C(4)), 67.1, 67.5, 69.2*, 69.3* (4 d, Fc), 81.1 (s, Fc), 87.0 (s, C(6)), 125.6, 125.9, 126.9, 128.54, 128.55, 128.6, 129.8, 131.9 (8 d, Ph, =CH), 136.0, 140.3 (2 s, Ph), 151.7 (s, C(3)) ppm; *signal with higher intensity. IR: ν = 3086*m*, 2888*m*, 1654*m*, 1588*m*, 1446*m*, 1399*m*, 1224*m*, 969*s*, 887*m*, 754*s*, 693*vs*
cm^{-1}. HRMS (ESI-TOF): *m*/*z* [M + H]$^+$ calcd for $C_{27}H_{24}FeNOS$: 466.0928; found: 466.0929.

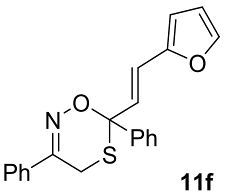

11f

(*E*)-3,6-Diphenyl-6-[2-(furan-2-yl)vinyl]-4*H*-1,5,2-oxathiazine (11f): reaction time: 60 min; yield: 145 mg (42%); pale yellow crystals, m. p. 131–133 °C (decomp.).

^{1}H NMR ($CDCl_3$, 600 MHz): δ = 3.41, 3.62 (AB system, *J* = 17.4 Hz, 2 H, $H_2C(4)$), 6.29 (d_{br}, *J* ≈ 3.3 Hz, 1 H, Fur), 6.36 (dd, *J* = 1.8, 3.3 Hz, 1 H, Fur), 6.47, 6.52 (AB system, *J* = 15.8 Hz, 2 H, =CH), 7.32–7.41, 7.60–7.62, 7.70–7.73 (3 m, 7 H, 2 H, 2 H, Ph, Fur) ppm. ^{13}C NMR ($CDCl_3$, 151 MHz): δ = 23.4 (t, C(4)), 86.6 (s, C(6)), 110.1, 111.5, 120.9, 125.7, 126.8, 127.3, 128.62, 128.63, 128.64, 129.8 (10 d, Ph, Fur, =CH), 136.0, 139.9 (2 s, Ph), 142.7 (d, Fur), 151.4 (s, C(3)), 152.1 (s, Fur) ppm. IR: ν = 2905*m*, 2887*m*, 1573*m*, 1485*m*, 1444*m*, 1388*m*, 1246*m*, 1228*m*, 1088*m*, 989*s*, 954*s*, 822*m*, 733*s*, 688*vs*, 679*vs* cm^{-1}. MS (ESI): *m*/*z* (%) = 370 (18, [M + Na]$^+$), 348 (100, [M + H]$^+$). Anal. Calcd for $C_{21}H_{17}NO_2S$ (347.43): C 72.60, H 4.93, N 4.03, S 9.23; found: C 72.67, H 5.04, N 4.06, S 9.30.

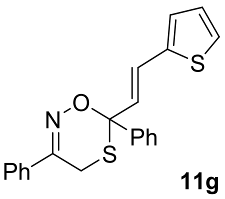

11g

(*E*)-3,6-Diphenyl-6-[2-(thien-2-yl)vinyl]-4*H*-1,5,2-oxathiazine (11g): reaction time: 30 min; yield: 145 mg (40%); pale yellow crystals, m. p. 151–152 °C (decomp.).

^1H NMR ($CDCl_3$, 600 MHz): $\delta = 3.41, 3.61$ (AB system, J = 17.4 Hz, 2 H, $H_2C(4)$), 6.35, 6.84 (AB system, J = 15.7 Hz, 2 H, =CH), 6.95 (dd, J = 3.6, 5.1 Hz, 1 H, Thie), 6.99 (d_{br}, $J \approx 3.6$ Hz, 1 H, Thie), 7.20 (d_{br}, $J \approx 5.1$ Hz, 1 H, Thie), 7.32–7.42, 7.60–7.62, 7.70–7.73 (3 m, 6 H, 2 H, 2 H, Ph) ppm. ^{13}C NMR ($CDCl_3$, 151 MHz): $\delta = 23.4$ (t, C(4)), 86.7 (s, C(6)), 125.4, 125.8, 126.3, 126.9, 127.3, 127.5, 128.3, 128.64, 128.66, 128.69, 129.9 (11 d, Ph, Thie, =CH), 136.0, 139.9, 140.6 (3 s, Ph, Thie), 152.3 (s, C(3)) ppm. IR: ν = 2955*m*, 2927*m*, 2901*m*, 2847*m*, 1591*m*, 1493*m*, 1485*m*, 1444*s*, 1389*m*, 1276*m*, 1223*m*, 1197*m*, 1078*m*, 954*s*, 946*s*, 923*s*, 873*m*, 850*m*, 753*s*, 709*s*, 698*vs*, 689*vs* cm^{-1}. MS (ESI): *m*/*z* (%) = 386 (12, $[M + Na]^+$), 364 (100, $[M + H]^+$). Anal. Calcd for $C_{21}H_{17}NOS_2$ (363.49): C 69.39, H 4.71, N 3.85, S 17.64; found: C 69.41, H 4.73, N 3.86, S 17.53.

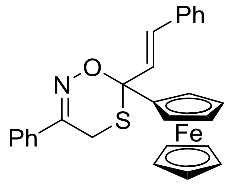

11h

(*E*)-6-Ferrocenyl-3-phenyl-6-styryl-4*H*-1,5,2-oxathiazine (11h): reaction time: 24 h; yield: 240 mg (52%); beige crystals, m. p. 81–82 °C.

^1H NMR ($CDCl_3$, 600 MHz): $\delta = 3.60, 3.67$ (AB system, J = 17.3 Hz, 2 H, $H_2C(4)$), 4.27 (m_c, 2 H, Fc), 4.31 (s, 5 H, Fc), 4.40–4.42, 4.46–4.48 (2 m, 1 H each, Fc), 6.51, 6.76 (AB system, J = 15.9 Hz, 2 H, 2 =CH), 7.27–7.30, 7.34–7.42, 7.44–7.47, 7.64–7.68 (4 m, 1 H, 5 H, 2 H, 2 H, Ph) ppm. ^{13}C NMR ($CDCl_3$, 151 MHz): $\delta = 23.8$ (t, C(4)), 66.9, 67.3, 68.5, 68.8, 69.4* (5 d, Fc), 84.8 (s, Fc), 88.6 (s, C(6)), 125.8, 126.8, 128.2, 128.3, 128.6, 128.7, 129.7, 131.2 (8 d, Ph, =CH), 135.9, 136.0 (2 s, Ph), 152.1 (s, C(3)) ppm; * signal with higher intensity. IR: ν = 3058*m*, 3026*m*, 2926*m*, 1578*m*, 1456*m*, 1444*m*, 1313*m*, 1221*m*, 967*s*, 906*s*, 818*m*, 725*s*, 689*vs* cm^{-1}. HRMS (ESI-TOF): *m*/*z* $[M + H]^+$ calcd for $C_{27}H_{24}FeNOS$: 466.0928; found: 466.0929.

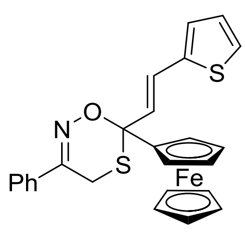

11i

(*E*)-6-Ferrocenyl-3-phenyl-6-[2-(thien-2-yl)vinyl]-4*H*-1,5,2-oxathiazine (11i): reaction time: 24 h; yield: 260 mg (55%); beige crystals, m. p. 102–104 °C.

^{1}H NMR ($CDCl_3$, 600 MHz): δ = 3.58, 3.68 (AB system, J = 17.3 Hz, 2 H, $H_2C(4)$), 4.26 (m_c, 2 H, Fc), 4.30 (s, 5 H, Fc), 4.40, 4.44 (2 s_{br}, 1 H each, Fc), 6.37, 6.88 (AB system, J = 15.6 Hz, 2 H, =CH), 6.98 (dd, J = 3.5, 4.9 Hz, 1 H, Thie), 7.01 (d_{br}, $J \approx$ 3.5 Hz, 1 H, Thie), 7.21 (d_{br}, $J \approx$ 4.9 Hz, 1 H, Thie), 7.39–7.42, 7.65–7.68 (2 m, 3 H, 2 H, Ph) ppm. ^{13}C NMR ($CDCl_3$, 151 MHz): δ = 23.8 (t, C(4)), 66.9, 67.3, 68.6, 68.8, 69.4* (5 d, Fc), 84.5 (s, Fc), 88.4 (s, C(6)), 124.5, 124.9, 125.8, 127.0, 127.6, 127.9, 128.6, 129.8 (8 d, Ph, Thie, =CH), 136.0, 140.8 (2 s, Ph, Thie), 152.3 (s, C(3)) ppm; *signal with higher intensity. IR: ν = 3039*m*, 2957*m*, 2894*m*, 1493*m*, 1442*m*, 1378*w*, 1226*m*, 998*s*, 956*s*, 823*m*, 733*s*, 695*s*, 689*vs* cm^{-1}. MS (ESI): *m*/*z* (%) = 472 (100, $[M + H]^+$). Anal. Calcd for $C_{25}H_{21}FeNOS_2$ (471.41): C 63.69, H 4.49, N 2.97, S 13.60; found: C 63.87, H 4.73, N 2.84, S 13.60.

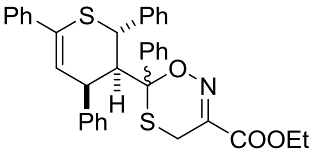

major-**14n**

Ethyl 6-(*trans*,*cis*-2',4',6'-Triphenyl-3',4'-dihydro-2'*H*-thiopyran-3'-yl)-6-phenyl-4*H*-1,5,2-oxathiazine-3-carboxylate (*major*-14n): reaction time: 35 min; yield: 75 mg (26%); colorless crystals, m. p. 175–176 °C (decomp.).

^{1}H NMR ($CDCl_3$, 600 MHz): δ = 1.30 (t, J = 7.1 Hz, 3 H, OCH_2CH_3), 2.31, 3.33 (AB system, J = 18.3 Hz, 2 H, $H_2C(4)$), 3.56 (dd, J = 4.1, 6.7 Hz, 1 H, HC(3')), 4.22–4.26 (m, 3 H, OCH_2CH_3, HC(4')), 4.75 (d_{br}, $J \approx$ 6.7 Hz, 1 H, HC(2')), 6.54 (d, J = 5.5 Hz, 1 H, HC(5')), 7.01–7.41, 7.63–7.66 (2 m, 18 H, 2 H, Ph) ppm. ^{13}C NMR ($CDCl_3$, 151 MHz): δ = 14.1 (q, OCH_2CH_3), 21.1 (t, C(4)), 42.2 (d, C(4')), 47.0 (d, C(2')), 55.9 (d, C(3')), 62.3 (t, OCH_2CH_3), 93.5 (s, C(6)), 121.9 (d, C(5')), 126.4, 126.8, 127.0, 127.2, 127.6, 127.9, 128.05, 128.07, 128.2, 128.4, 129.0, 129.8 (12 d, Ph), 135.6, 139.1, 139.6, 140.7, 140.9 (5 s, Ph, C(6')), 145.9 (s, C(3)), 162.5 (s, C=O) ppm. IR: ν = 3057*w*, 3029*w*, 1713*vs* (C=O), 1491*m*, 1445*m*, 1321*s*, 1249*m*, 760*s*, 706*s*, 696*s* cm^{-1}. HRMS (ESI-TOF): *m*/*z* $[M + Na]^+$ calcd for $C_{35}H_{31}NNaO_3S_2$: 600.1643; found: 600.1647.

Selected diagnostic signals of minor isomer (*minor*-**14n**): ^{1}H NMR ($CDCl_3$, 600 MHz): δ = 1.24 (t, J = 7.1 Hz, 3 H, OCH_2CH_3), 2.69, 3.35 (AB system, J = 18.7 Hz, 2 H, $H_2C(4)$), 3.36 (t_{br}, $J \approx$ 4.5 Hz, 1 H, HC(3')), 3.98 (t_{br}, $J \approx$ 4.2 Hz, 1 H, HC(4')), 4.02 (d, J = 4.1 Hz, 1 H, HC(2')), 4.30 (q, J = 7.1 Hz, 2 H, OCH_2CH_3), 6.58 (d, J = 3.6 Hz, 1 H, HC(5')) ppm.

Synthesis of thiopyranyl-substituted 1,5,2-oxathiazines 14b: Following the general protocol by using '*thiochalcone fraction*' **2b**/**4b** (455 mg, 1.5 mmol scale with respect to monomer **1b**)

equilibrated in dichloromethane overnight, the resulting mixture containing **11b**, *major*-**14b**, and *minor*-**14b** in a ca. 3:6:1 ratio was purified by column chromatography (SiO_2, petroleum ether/dichloromethane 4:1 gradient 1:1) to give a partially purified mixture of diastereoisomers **14b** (first eluted, 172 mg) and 1,5,2-oxathiazine **11b** (second eluted, 111 mg, 17%). Additional purification of the former fraction by column chromatography (SiO_2, petroleum ether/dichloromethane 2:1) followed by preparative TLC (SiO_2, pentane/dichloromethane 3:1) enabled isolation of small samples of pure *major*-**14b** (R_f = 0.42, 48 mg, 9%) and *minor*-**14b** (R_f = 0.38, 11 mg, 2%).

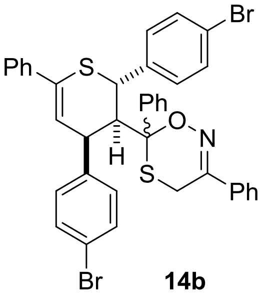

14b

Major-isomer: **6-[*trans,cis*-2',4'-Bis(4-bromophenyl)-6'-phenyl-3',4'-dihydro-2'*H*-thiopyran-3'-yl]-3,6-diphenyl-4*H*-1,5,2-oxathiazine (14b):** colorless glassy solid, m. p. 119–123 °C (decomp.).

^{1}H NMR ($CDCl_3$, 600 MHz): δ = 2.58, 3.25 (AB system, *J* = 16.8 Hz, 2 H, $H_2C(4)$), 3.61 (dd, *J* = 3.5, 9.1 Hz, 1 H, HC(3')), 4.40 (dd_{br}, $J \approx 3.5$, 6.6 Hz, 1 H, HC(4')), 4.97 (d, *J* = 9.1 Hz, 1 H, HC(2')), 6.36 (d, *J* = 6.6 Hz, 1 H, HC(5')), 6.99–7.10, 7.29–7.40, 7.42–7.45, 7.56–7.59 (4 m, 9 H, 10 H, 2 H, 2 H, Ph, Ar) ppm. ^{13}C NMR ($CDCl_3$, 151 MHz): δ = 22.2 (t, C(4)), 42.3 (d, C(4')), 45.6 (d, C(2')), 54.3 (d, C(3')), 92.2 (s, C(6)), 121.1, 121.2 (2 s, 2 C-Br), 121.7 (d, C(5')), 125.6, 126.3, 126.92, 127.04, 127.8, 128.4, 128.5, 128.6, 129.9, 130.76, 130.81, 131.4, 132.2 (13 d, Ph, Ar), 135.5, 135.7, 138.4, 138.5, 139.6, 141.1 (6 s, Ph, Ar, C(6')), 153.3 (s, C(3)) ppm. IR: ν = 3056–2853*w*, 1700*m*, 1593*w*, 1485*s*, 1444*m*, 1074*m*, 1010*m*, 906*s*, 729*s*, 690*s* cm^{-1}. MS (ESI): *m/z* (%) = 764 (59, $[M\{^{81}Br_2\} + Na]^+$), 762 (100, $[M\{^{79}Br^{81}Br\} + H]^+$), 760 (46, $[M\{^{79}Br_2\} + Na]^+$), 252 (87). HRMS (ESI-TOF): *m/z* $[M + Na]^+$ calcd for $C_{38}H_{29}Br_2NNaOS_2$: 759.9955; found: 759.9955.

minor-isomer of **14b**: thick colorless oil.

^{1}H NMR ($CDCl_3$, 600 MHz): δ = 2.97, 3.17 (AB system, *J* = 17.3 Hz, 2 H, $H_2C(4)$), 3.31 (t_{br}, $J \approx 5.0$ Hz, 1 H, HC(3')), 4.04 (t_{br}, $J \approx 4.3$ Hz, 1 H, HC(4')), 4.12 (d_{br}, $J \approx 5.2$ Hz, 1 H, HC(2')), 6.45 (d, *J* = 4.2 Hz, 1 H, HC(5')), 6.99–7.03, 7.19–7.39, 7.55–7.63 (3 m, 2 H, 17 H, 4 H, Ph, Ar) ppm. ^{13}C NMR ($CDCl_3$, 151 MHz): δ = 22.3 (t, C(4)), 40.4 (d, C(4')), 45.5 (d, C(2')), 55.4 (d, C(3')), 91.6 (s, C(6)), 120.3, 121.4 (2 s, 2 C-Br), 121.9 (d, C(5')), 125.5, 126.4, 127.7, 128.36, 128.45, 128.5, 128.6, 128.7, 129.7, 130.2, 130.8, 131.4, 131.8 (13 d, Ph, Ar), 134.4, 135.6, 139.1, 140.5, 140.8, 141.2 (6 s, Ph, Ar, C(6')), 153.2 (s, C(3)) ppm. MS (ESI): *m/z* (%) = 764 (31, $[M\{^{81}Br_2\} + Na]^+$), 762 (52, $[M\{^{79}Br^{81}Br\}$

+ H]$^{+}$), 760 (25, [M{^{79}Br$_2$} + Na]$^{+}$), 252 (100). HRMS (ESI-TOF): *m*/*z* [M + Na]$^{+}$ calcd for $C_{38}H_{29}Br_2NNaOS_2$: 759.9955; found: 759.9955.

Table S1. Selected diagnostic ^{1}H-NMR signals ($CDCl_3$, 600 MHz) of diastereomeric cycloadducts **14** derived from thiopyrans *endo*-**4** and nitrosoalkenes **10**.[a]

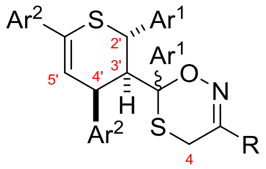

14

	**$H_2C(4)$** (AB system)	**HC(2')**	**HC(3')**	**HC(4')**	**HC(5')** (d)
major-**14a**	2.66, 3.09 (J = 16.5 Hz)	5.02 (d, J = 7.7 Hz)	3.69 (dd, J = 3.9, 7.7 Hz)	4.19 (dd_{br}, $J \approx 3.9$, 6.0 Hz)	6.46 (J = 6.0 Hz)
minor-**14a**	2.99, 3.20 (J = 17.3 Hz)	4.14 (d_{br}, $J \approx 4.6$ Hz)	3.40 (t_{br}, $J \approx 4.6$ Hz)	4.08 (t_{br}, $J \approx 4.3$ Hz)	6.60 (J = 3.8 Hz)
major-**14b** [b]	2.58, 3.25 (J = 16.8 Hz)	4.97 (d, J = 9.1 Hz)	3.61 (dd, J = 3.5, 9.1 Hz)	4.40 (dd_{br}, $J \approx 3.5$, 6.6 Hz)	6.36 (J = 6.6 Hz)
minor-**14b** [b]	2.97, 3.17 (J = 17.3 Hz)	4.12 (d_{br}, $J \approx 5.2$ Hz)	3.31 (t_{br}, $J \approx 5.0$ Hz)	4.04 (t_{br}, $J \approx 4.3$ Hz)	6.45 (J = 4.2 Hz)
major-**14c**	2.61, 3.13 (J = 16.4 Hz)	5.04 (d, J = 8.9 Hz)	3.65 (dd, J = 3.6, 8.9 Hz)	4.27 (dd_{br}, $J \approx 3.6$, 6.8 Hz)	6.37 (J = 6.8 Hz)
minor-**14c**	2.98, 3.19 (J = 17.2 Hz)	4.25 (d_{br}, $J \approx 5.2$ Hz)	3.39 (t_{br}, $J \approx 5.3$ Hz)	4.17 (t_{br}, $J \approx 4.3$ Hz)	6.52 (J = 4.4 Hz)
major-**14d**	2.59, 3.06 (J = 16.3 Hz)	5.38 (d, J = 8.6 Hz)	3.90 (dd, J = 3.6, 8.6 Hz)	4.48 (dd_{br}, $J \approx 3.6$, 6.6 Hz)	6.55 (J = 6.6 Hz)
minor-**14d**	2.93, 3.13 (J = 17.1 Hz)	[c]	[c]	[c]	6.72 (J = 4.3 Hz)
major-**14f**	2.86, 3.23 (J = 16.6 Hz)	5.35 (d, J = 7.8 Hz)	3.79 (dd, J = 3.9, 7.8 Hz)	3.96 (dd_{br}, $J \approx 3.8$, 6.2 Hz)	6.17 (J = 6.2 Hz)
minor-**14f**	2.91, 3.23 (J = 17.2 Hz)	4.70 (d, J = 5.7 Hz)	4.76 (dd_{br}, $J \approx 4.4$, 5.7 Hz)	4.10 (t_{br}, $J \approx 4.6$ Hz)	6.27 (J = 4.8 Hz)
major-**14g**	2.72, 3.25 (J = 16.9 Hz)	5.41 (d, J = 7.2 Hz)	3.67 (dd, J = 3.6, 7.2 Hz)	3.52 (dd, J = 3.6, 6.0 Hz)	6.45 (J = 6.1 Hz)
minor-**14g**	3.12, 3.41 (J = 13.5 Hz)	4.30 (d, J = 6.2 Hz)	3.55 (t_{br}, $J \approx 5.1$ Hz)	[c]	[c]
major-**14j**	2.83, 3.13 (J = 16.7 Hz)	5.06 (d, J = 7.2 Hz)	3.63 (dd, J = 3.8, 7.2 Hz)	~ 4.19 (m) [d]	6.40 (J = 6.1 Hz)

minor-**14j**	3.08, 3.24 (J = 17.4 Hz)	~ 4.19 (m) [d]	3.33 (t_{br}, $J \approx 4.5$ Hz)	4.03 (t_{br}, $J \approx 4.1$ Hz)	[c]
major-**14k**	2.70, 2.96 (J = 13.9 Hz)	5.41 (d, J = 9.6 Hz)	4.16 (dd, J = 4.0, 9.6 Hz)	3.74 (dd, J = 4.0, 6.6 Hz)	6.54 (J = 6.6 Hz)
minor-**14k**	2.98, 3.04 (J = 14.6 Hz)	4.92 (d, J = 9.2 Hz)	4.12 (dd, J = 4.5, 9.2 Hz)	4.60 (t_{br}, $J \approx 5.4$ Hz)	6.72 (J = 6.1 Hz)
major-**14l**	2.86, 2.98 (J = 15.1 Hz)	5.25 (d, J = 8.3 Hz)	3.84 (dd, J = 3.9, 8.3 Hz)	4.03 (dd, J = 3.9, 6.2 Hz)	6.41 (J = 6.2 Hz)
minor-**14l**	3.19, 3.24 (J = 16.5 Hz)	4.33 (d, J = 5.5 Hz)	3.59 (t_{br}, $J \approx 5.1$ Hz)	4.19 (t_{br}, $J \approx 4.7$ Hz)	6.64 (J = 4.3 Hz)
major-**14m**	2.32, 2.90 (J = 17.9 Hz)	4.67 (d, J = 6.6 Hz)	3.52 (dd, J = 4.3, 6.6 Hz)	4.21 (dd_{br}, $J \approx 4.3$, 5.4 Hz)	6.52 (J = 5.4 Hz)
minor-**14m**	2.70, 2.92 (J = 18.2 Hz)	4.07 (d, J = 4.2 Hz)	3.30 (t_{br}, $J \approx 4.4$ Hz)	3.93 (t_{br}, $J \approx 4.0$ Hz)	6.59 (J = 3.4 Hz)
major-**14n** [b]	2.31, 3.33 (J = 18.3 Hz)	4.75 (d_{br}, $J \approx 6.7$ Hz)	3.56 (dd, J = 4.1, 6.7 Hz)	[c]	6.54 (J = 5.5 Hz)
minor-**14n**	2.69, 3.35 (J = 18.7 Hz)	4.02 (d, J = 4.1 Hz)	3.36 (t_{br}, $J \approx 4.5$ Hz)	3.98 (t_{br}, $J \approx 4.2$ Hz)	6.58 (J = 3.6 Hz)

[a] From ^{1}H-NMR spectra of partially purified mixtures containing second diastereoisomer of **14** and/or 1,5,2-oxathiazine derivative **11**.
[b] From ^{1}H-NMR spectra of pure compound.
[c] Overlapping signal with other signals.
[d] Overlapping signals attributed to HC(4') of *major*-**14j** and HC(2') of *minor*-**14j**.

3. References synthetic part

[1] T. Karakasa, S. Motoki, *J. Org. Chem.* **1978**, *43*, 4147-4150.

[2] G. Mlostoń, P. Grzelak, H. Heimgartner, *J. Sulfur Chem.* **2017**, *38*, 1-10.

[3] P. Grzelak, G. Utecht, M. Jasiński, G. Mlostoń, *Synthesis* **2017**, *49*, 2129-2137.

[4] J. Hejmanowska, M. Jasiński, J. Wojciechowski, G. Mlostoń, Ł. Albrecht, *Chem. Commun.* **2017**, *53*, 11472-11475.

[5] G. Mlostoń, R. Hamera-Fałdyga, H. Heimgartner, *J. Sulfur Chem.* **2018**, *39*, 322-331.

[6] H. Korten, R. Scholl, *Ber. Dtsch. Chem. Ges.* **1901**, *34*, 1901-1910.

[7] R. Zimmer, H.-U. Reissig, *J. Org. Chem.* **1992**, *57*, 339-347.

[8] T. L. Gilchrist, T. G. Roberts, *J. Chem. Soc., Perkin Trans. 1* **1983**, 1283-1292.

[9] S. Motoki, T. Saito, T. Karakasa, T. Matsushita, E. Furuno, *J. Chem. Soc., Perkin Trans. 1*, **1992**, 2943-2948.

[10] G. Mlostoń, K. Urbaniak, R. Zimmer, H.-U. Reissig, H. Heimgartner, *ChemistrySelect* **2018**, *3*, 11724-11728.

3. NMR spectra

Copies of ^{1}H NMR and ^{13}C NMR spectra for selected compounds are shown in Figures 1-33.

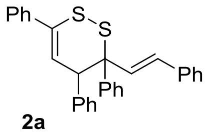

2a

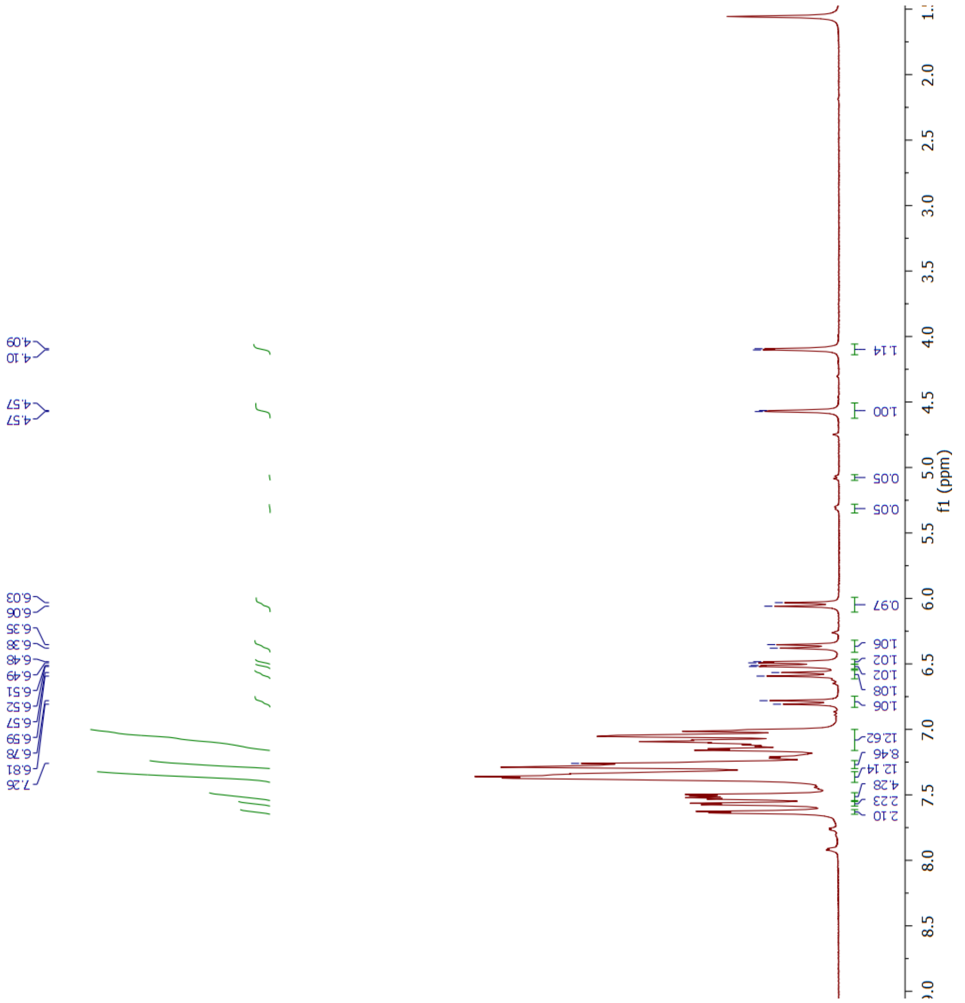

Figure 1. ^{1}H NMR of **2a** (1:1 mixture of *cis* and *trans* isomers) contaminated with *endo*-**4a** ($CDCl_3$, 600 MHz).

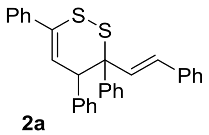

2a

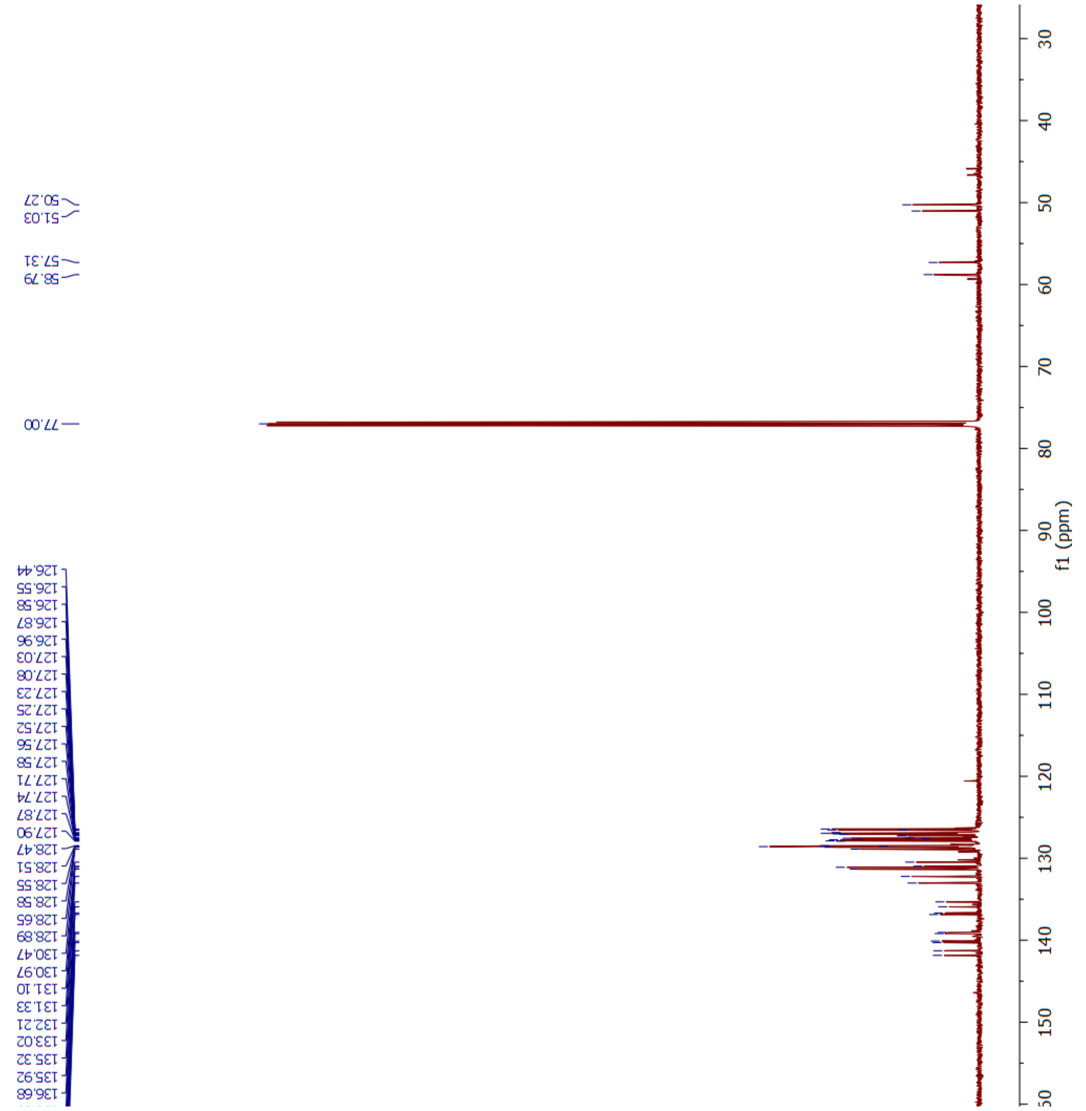

Figure 2. ^{13}C NMR of **2a** (1:1 mixture of *cis* and *trans* isomers) contaminated with *endo*-**4a** ($CDCl_3$, 151 MHz).

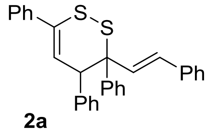

2a

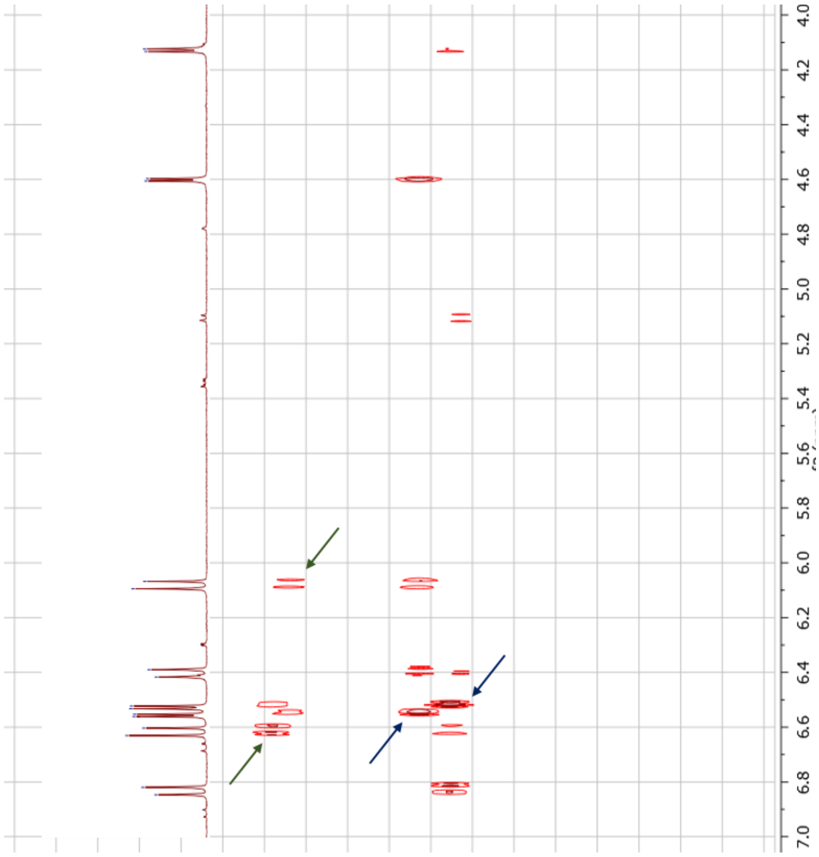

Figure 3. Diagnostic part of HMBC spectrum of diastereomers **2a** indicating three-bond-distance correlations between C(3) and *H*C(5) (marked with *blue* arrows) and between C(4) and *H*C(1') (*green* arrows).

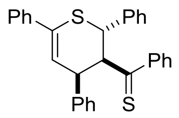

endo-**4a**

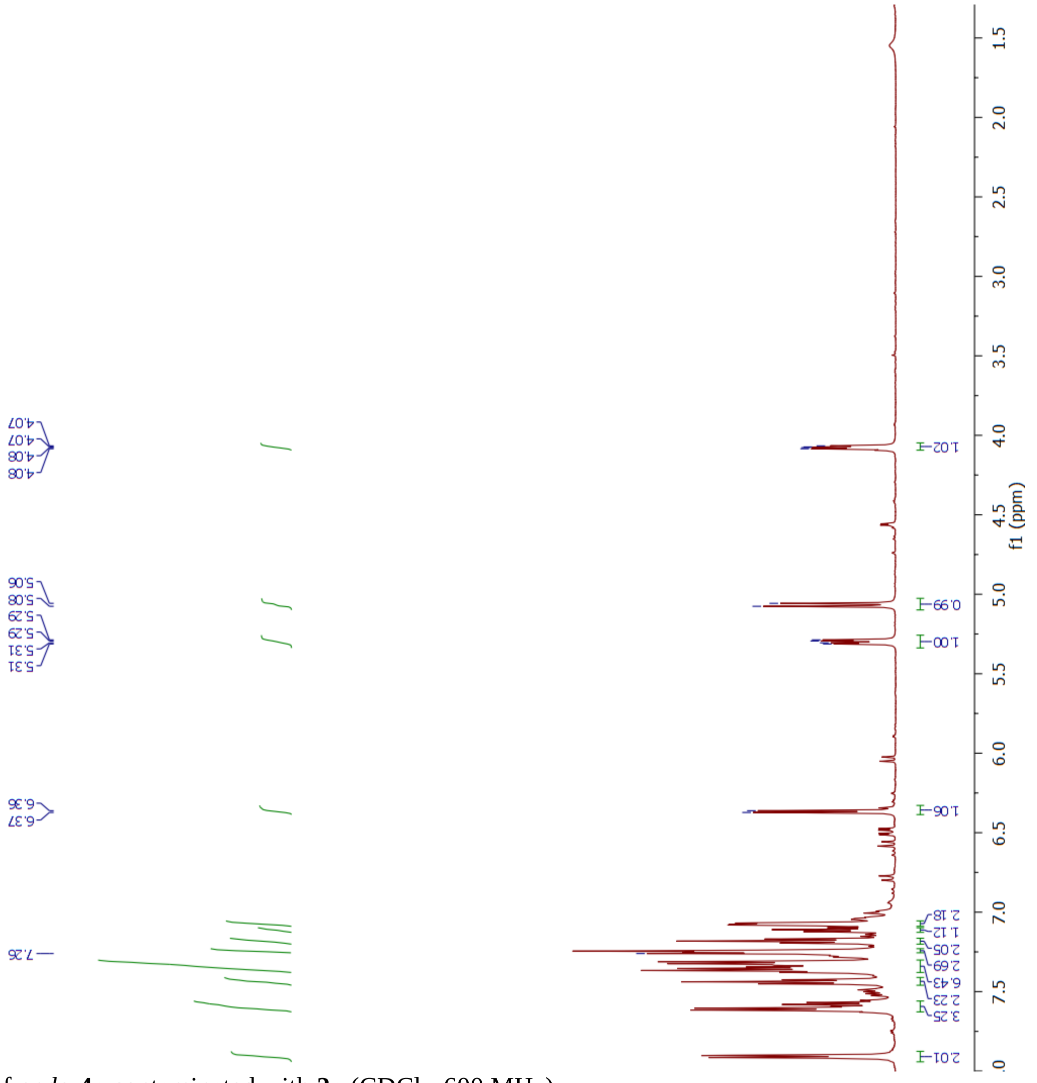

Figure 4. ^{1}H NMR of *endo*-**4a** contaminated with **2a** ($CDCl_3$, 600 MHz).

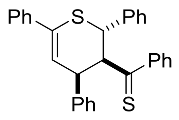

endo-**4a**

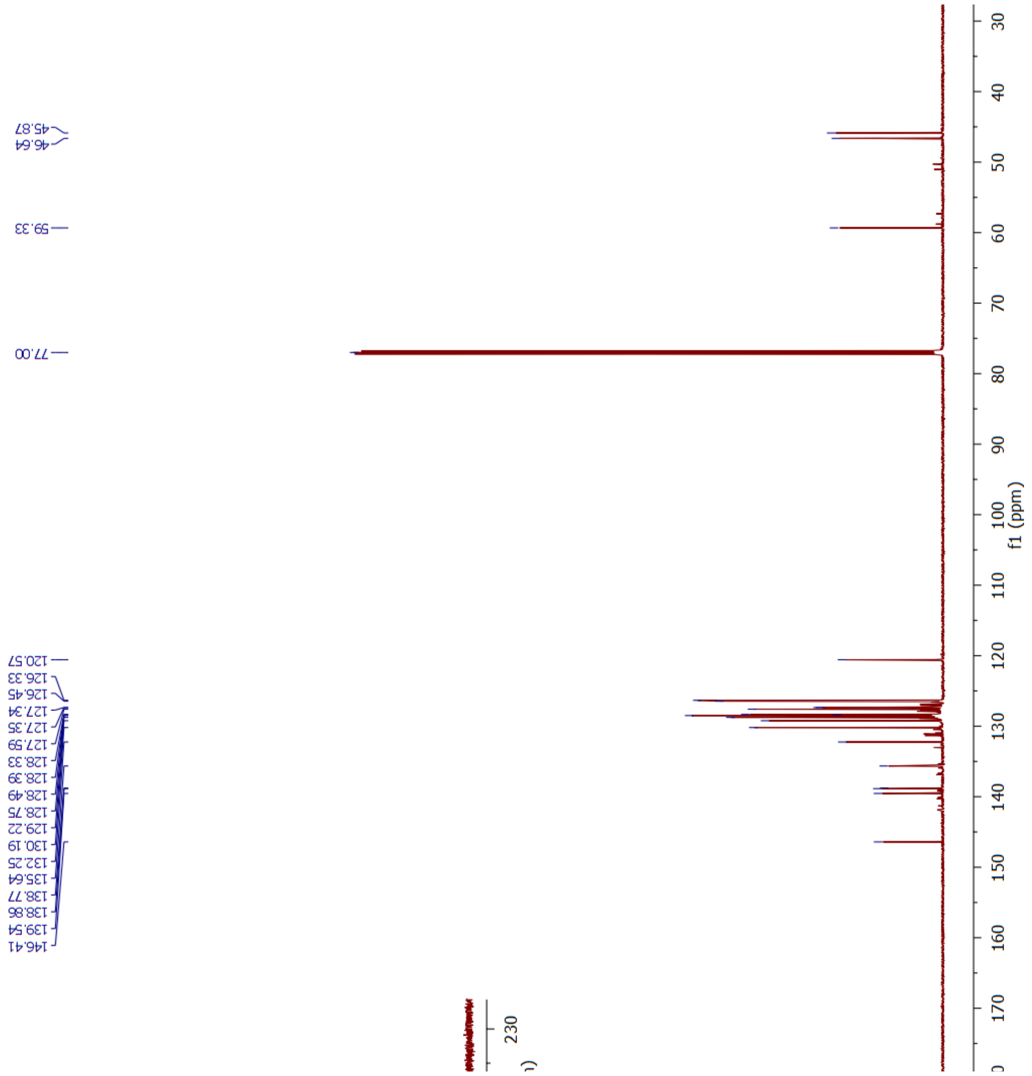

Figure 5. ^{13}C NMR of *endo*-**4a** contaminated with **2a** ($CDCl_3$, 151 MHz).

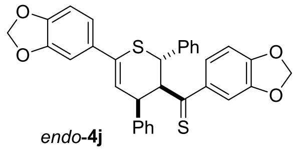

endo-**4j**

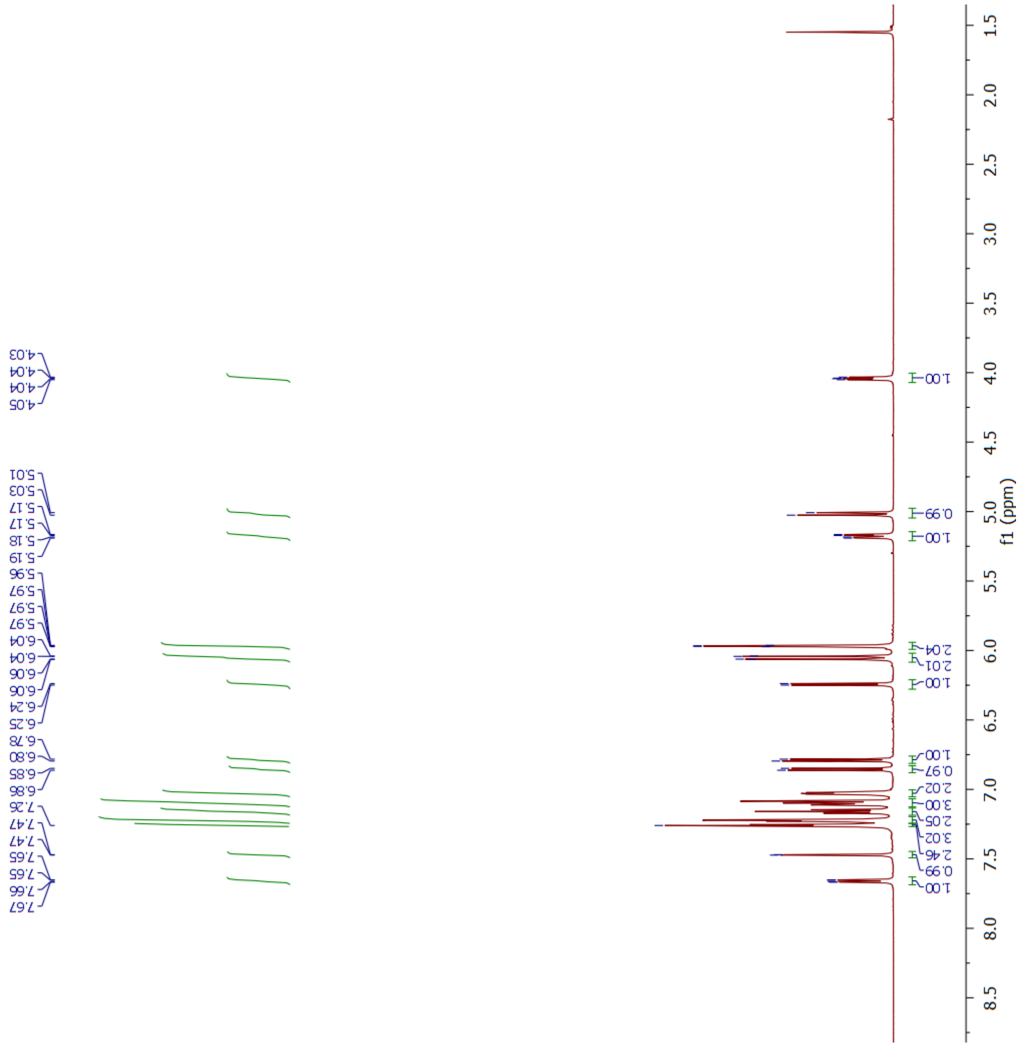

Figure 6. ^{1}H NMR of *endo*-**4j** ($CDCl_3$, 600 MHz).

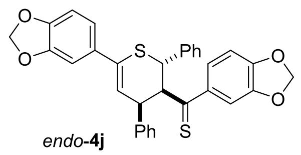

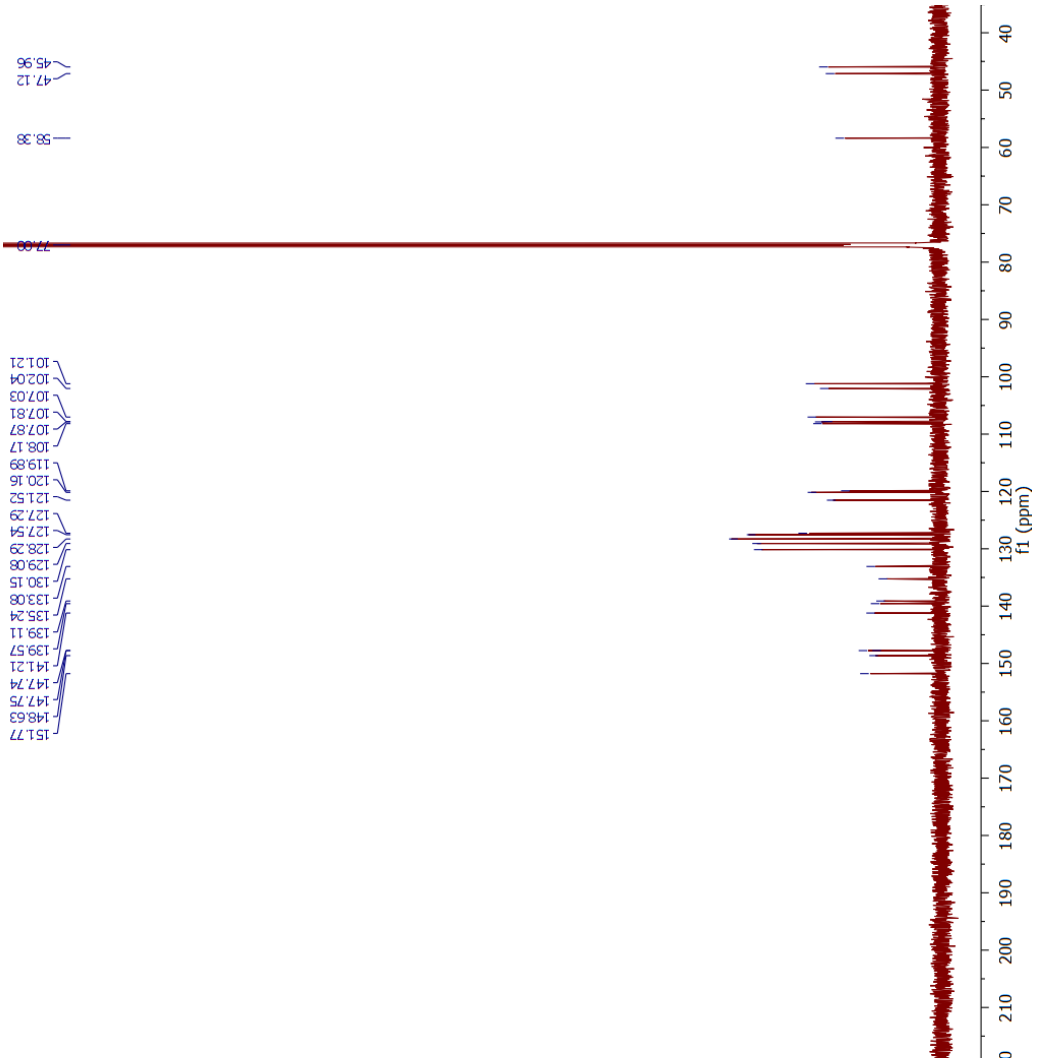

Figure 7. ^{13}C NMR of *endo*-**4j** ($CDCl_3$, 151 MHz).

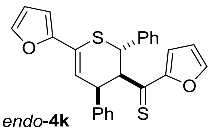

endo-**4k**

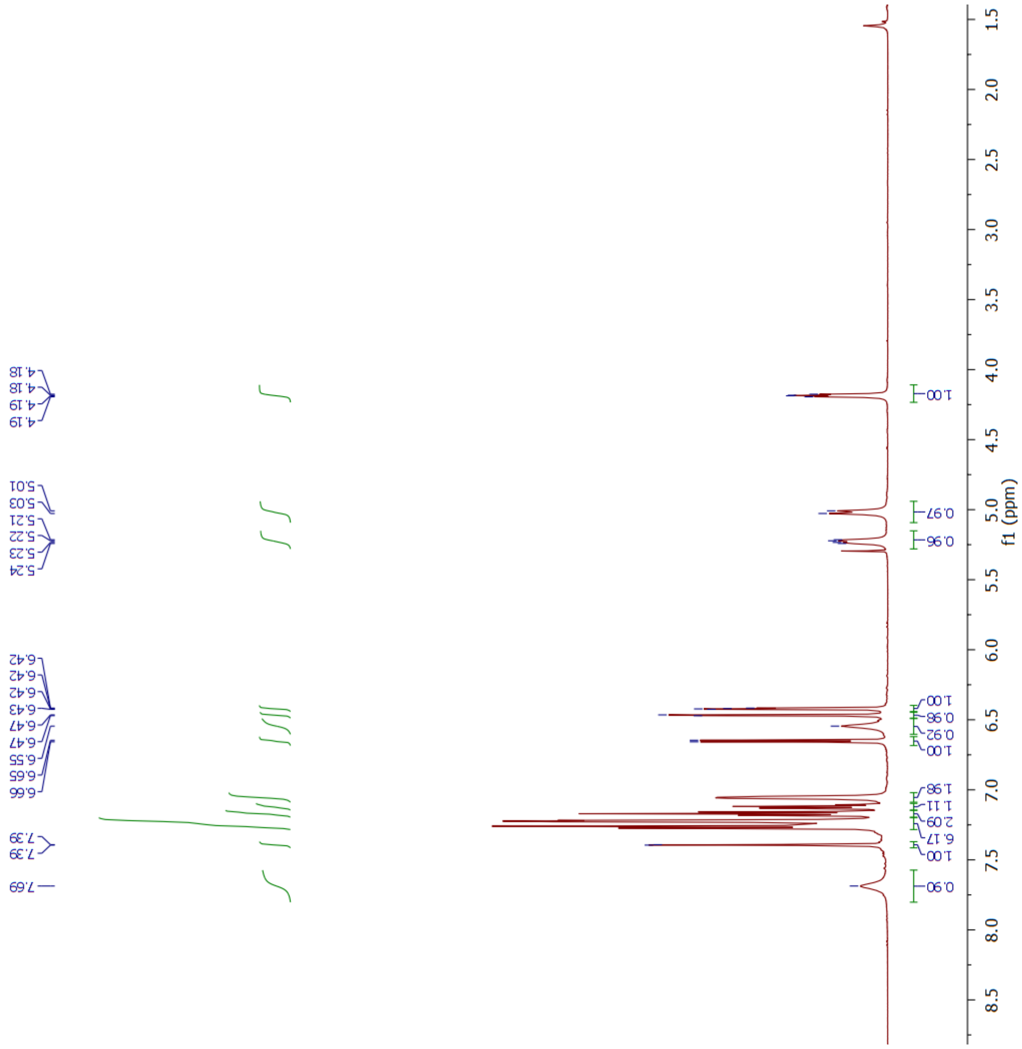

Figure 8. ^{1}H NMR of *endo*-**4k** ($CDCl_3$, 600 MHz).

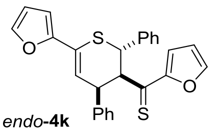

endo-**4k**

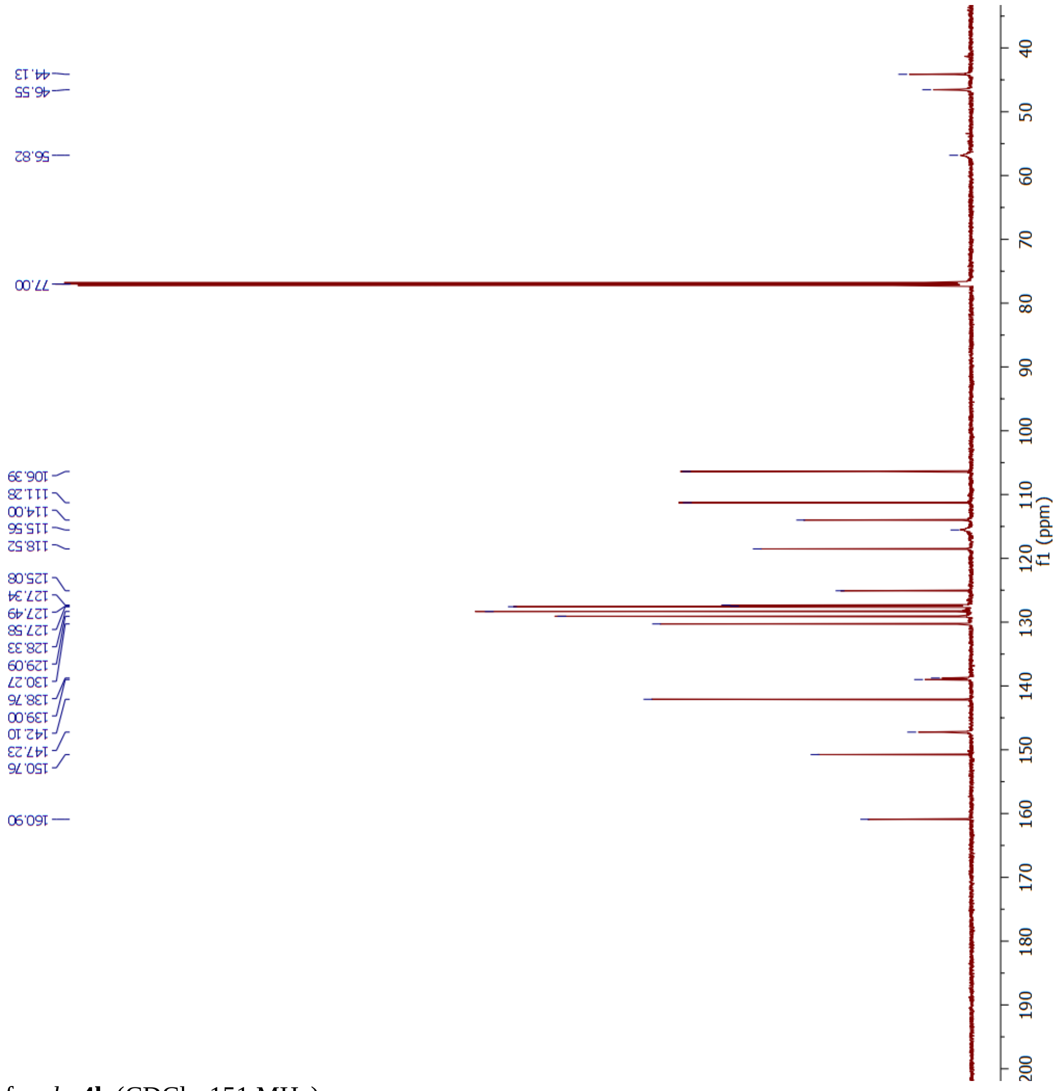

Figure 9. ^{13}C NMR of *endo*-**4k** ($CDCl_3$, 151 MHz).

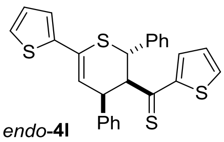

endo-**4l**

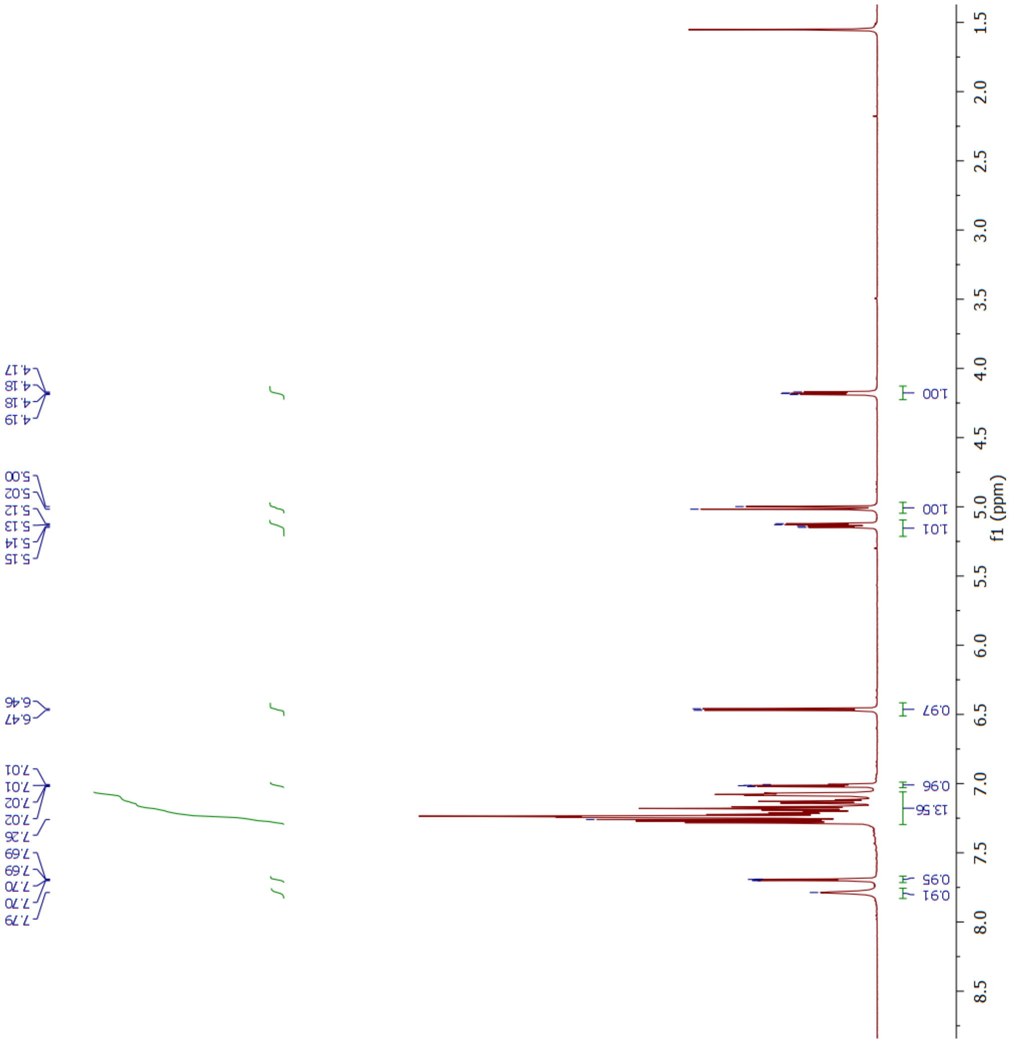

Figure 10. ^{1}H NMR of *endo*-**4l** ($CDCl_3$, 600 MHz).

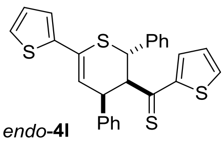

endo-**4l**

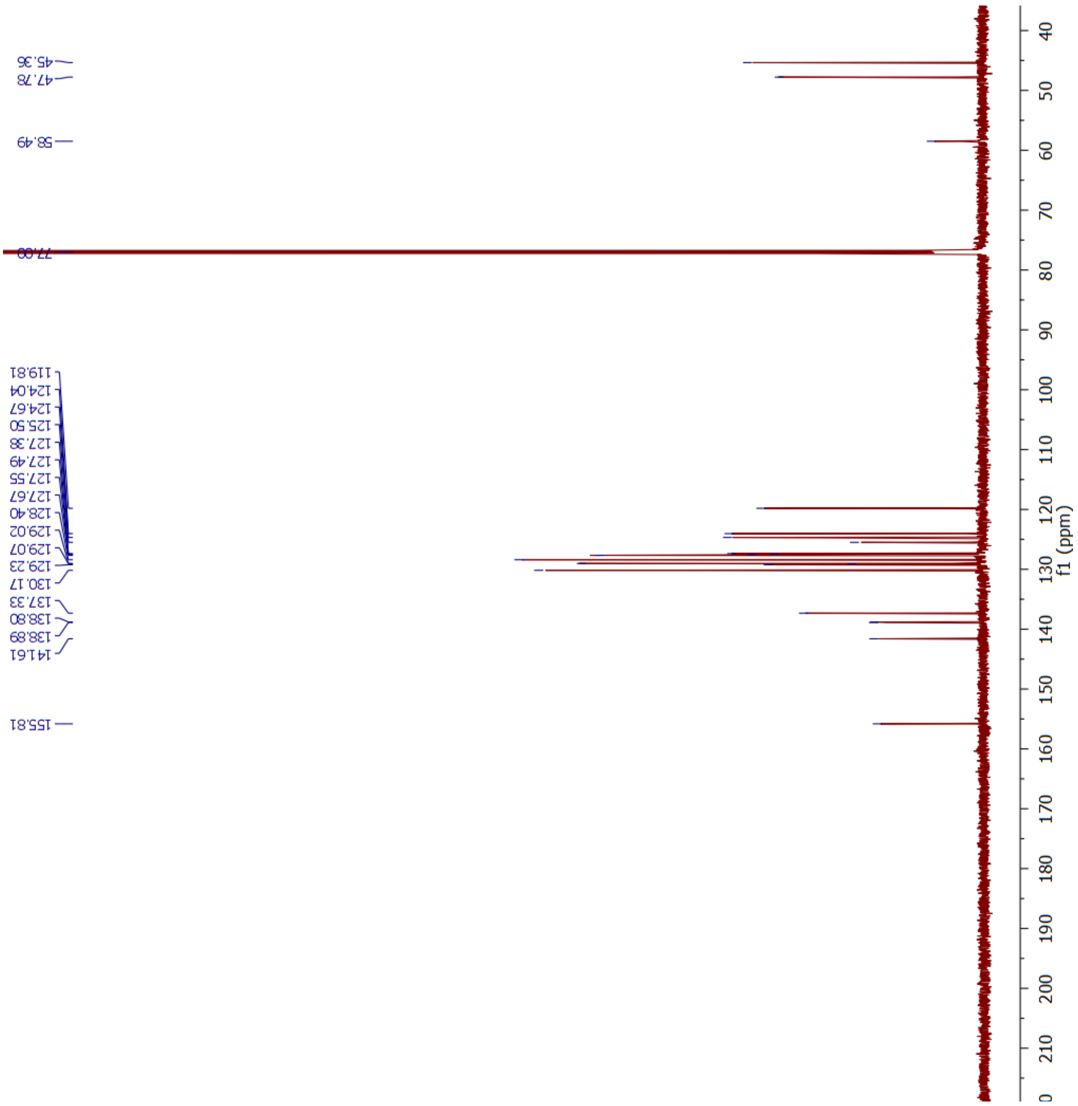

Figure 11. ^{13}C NMR of *endo*-**4l** ($CDCl_3$, 151 MHz).

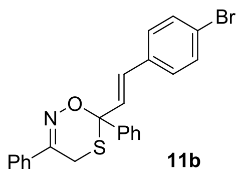

11b

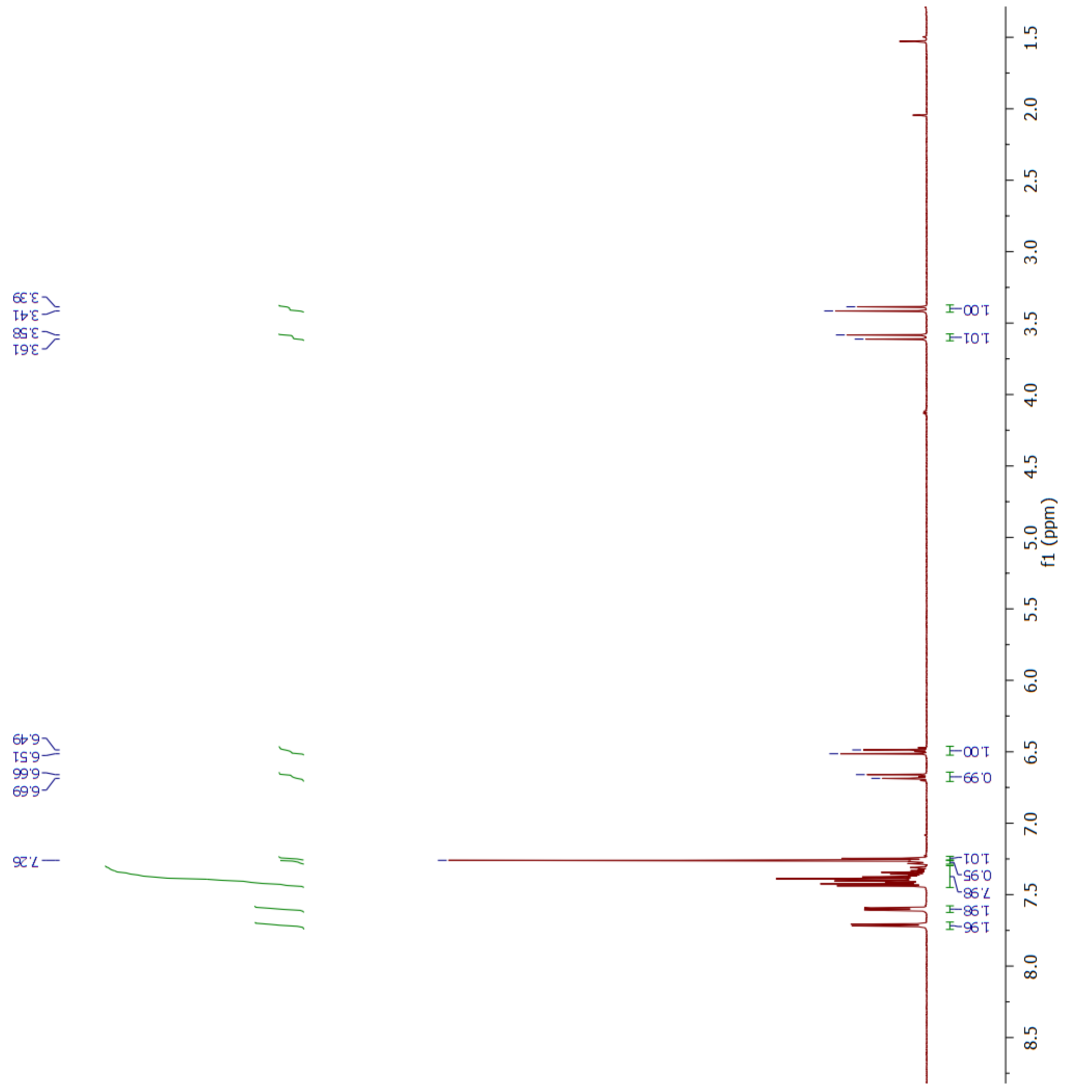

Figure 12. ^{1}H NMR of **11b** ($CDCl_3$, 600 MHz).

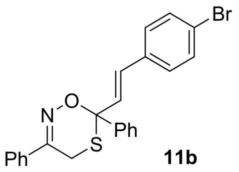

11b

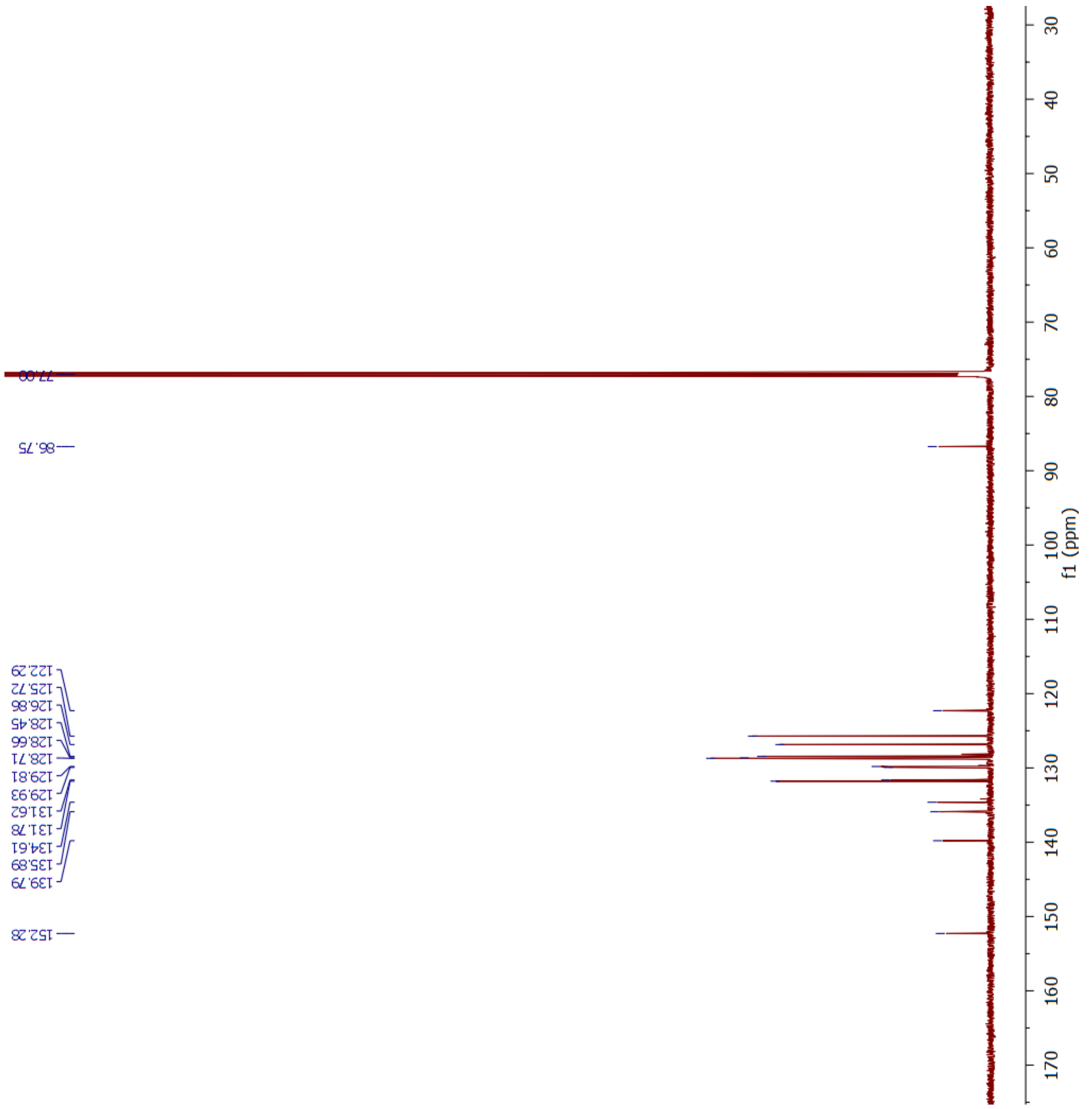

Figure 13. ^{13}C NMR of **11b** ($CDCl_3$, 151 MHz).

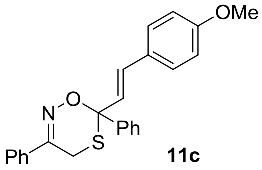

11c

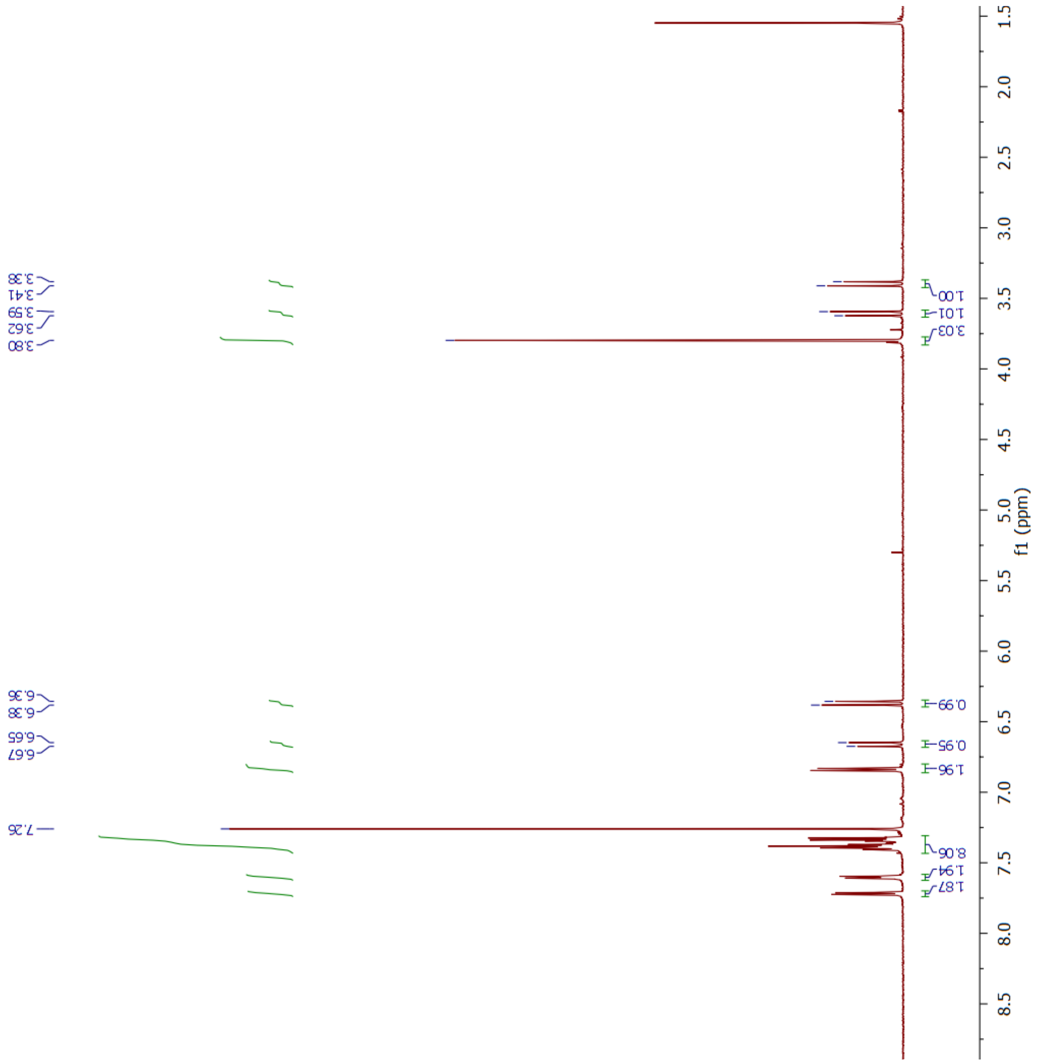

Figure 14. ^{1}H NMR of **11c** ($CDCl_3$, 600 MHz).

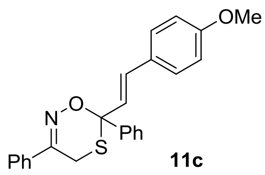

11c

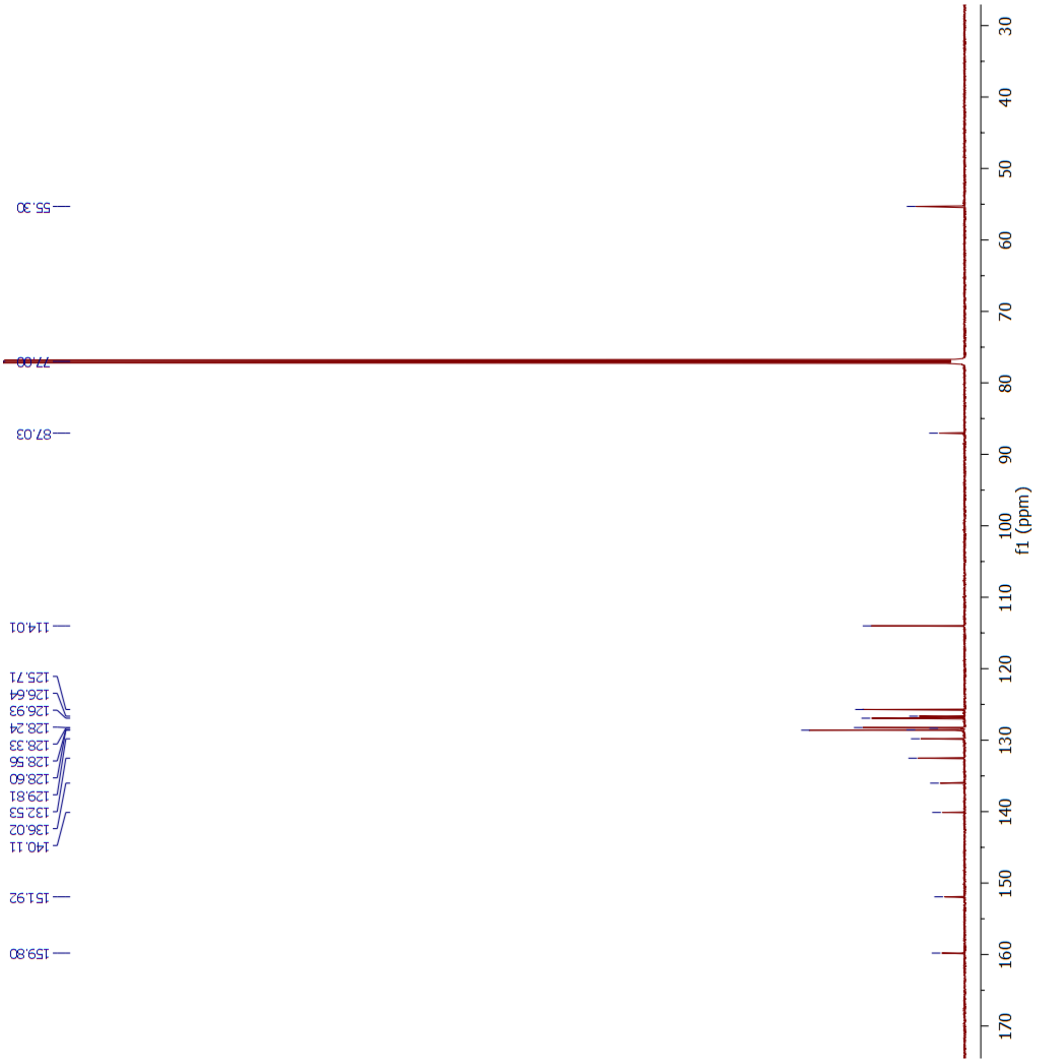

Figure 15. ^{13}C NMR of **11c** ($CDCl_3$, 151 MHz).

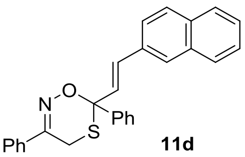

11d

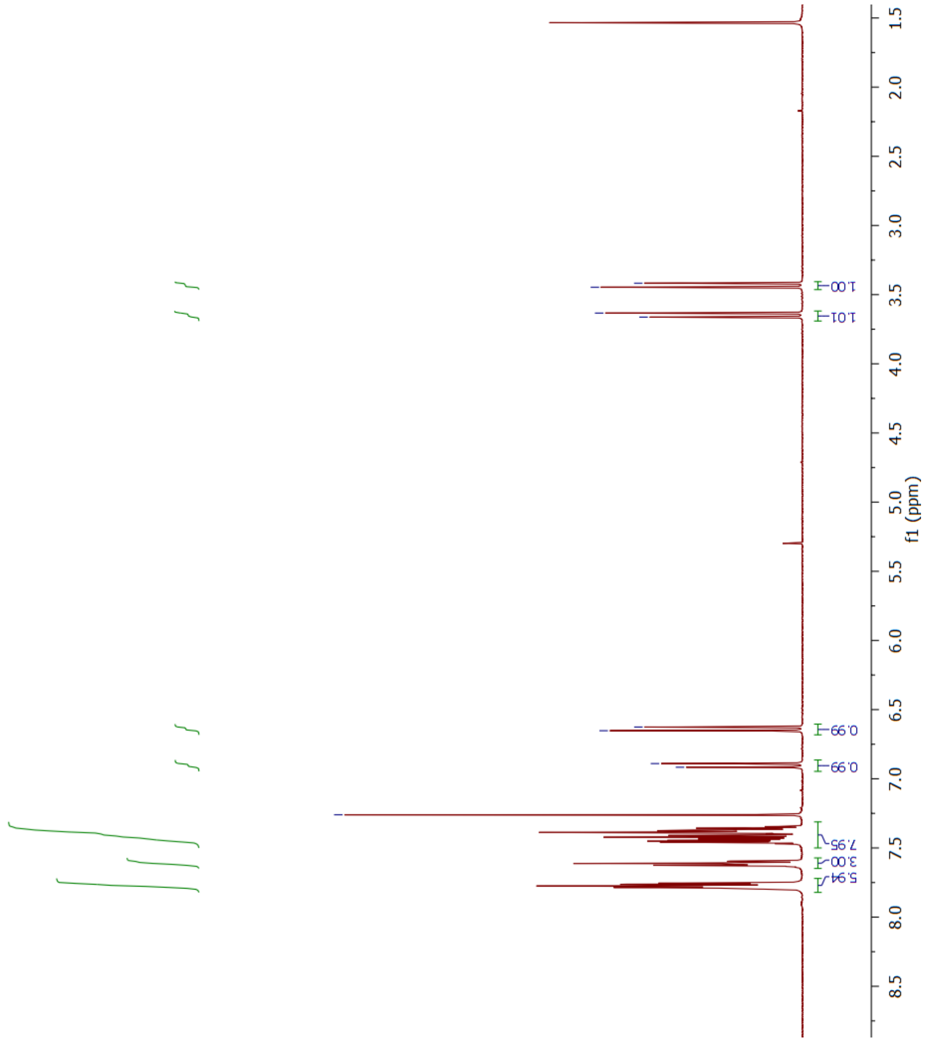

Figure 16. ^{1}H NMR of **11d** ($CDCl_3$, 600 MHz).

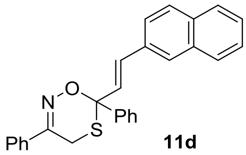

11d

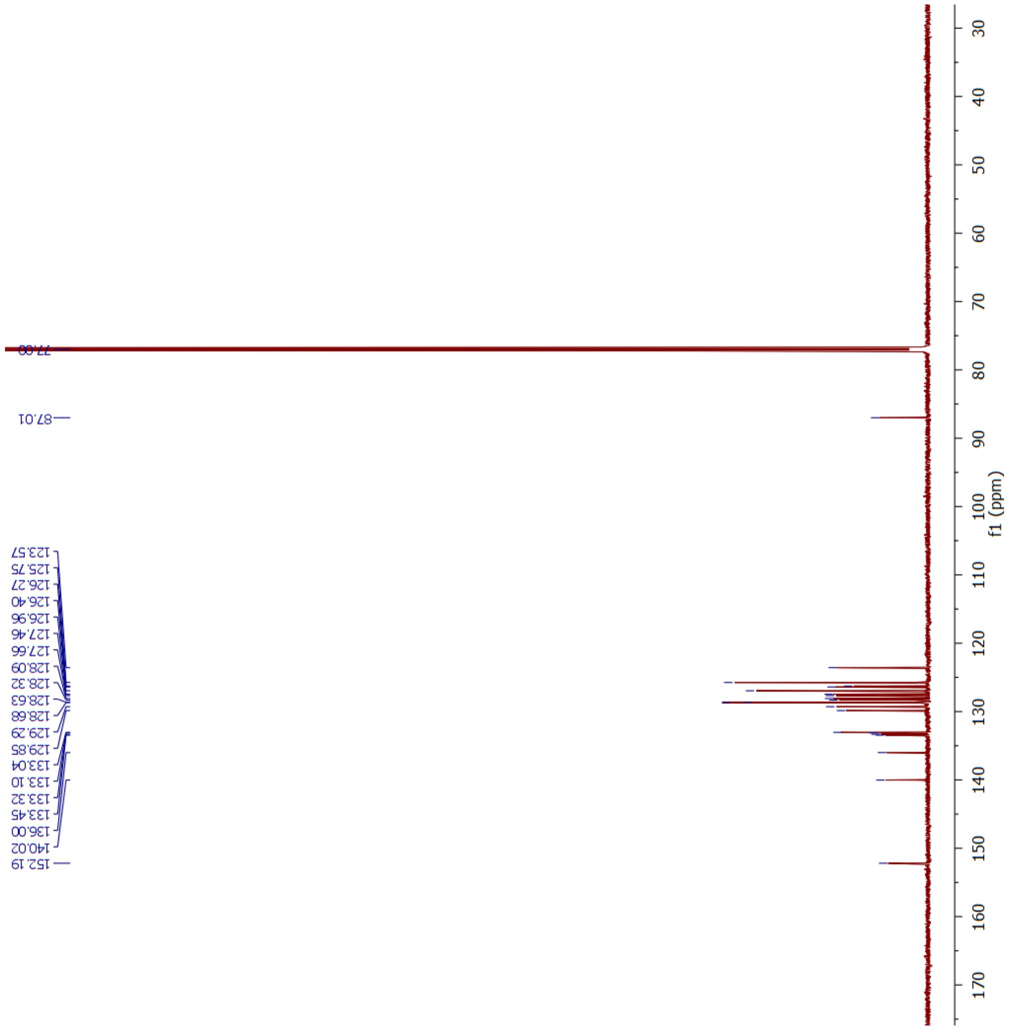

Figure 17. ^{13}C NMR of **11d** ($CDCl_3$, 151 MHz).

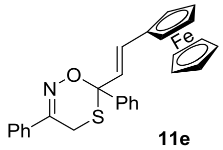

11e

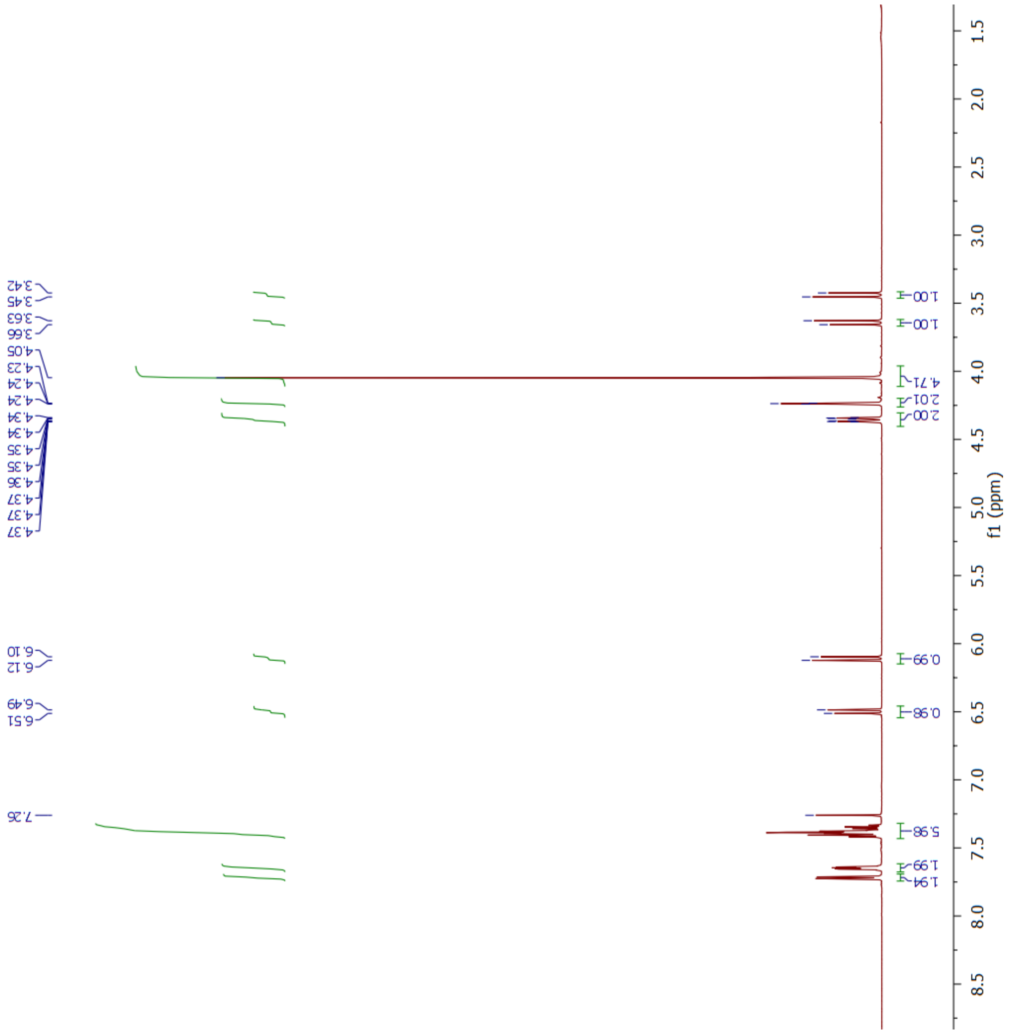

Figure 18. ^{1}H NMR of **11e** ($CDCl_3$, 600 MHz).

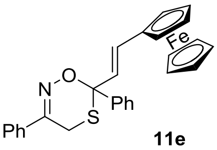

11e

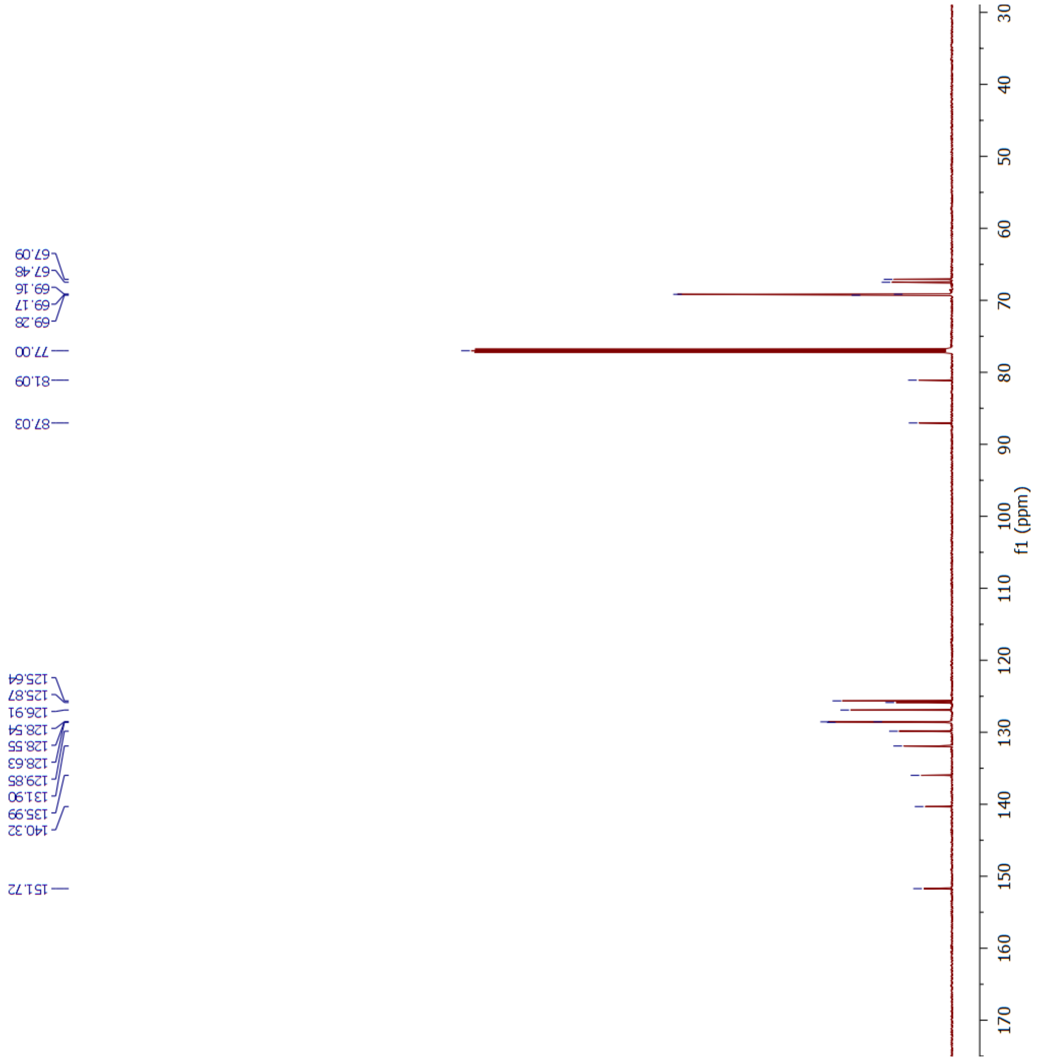

Figure 19. ^{13}C NMR of **11e** ($CDCl_3$, 151 MHz).

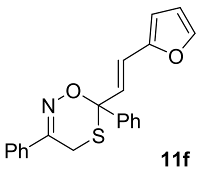

11f

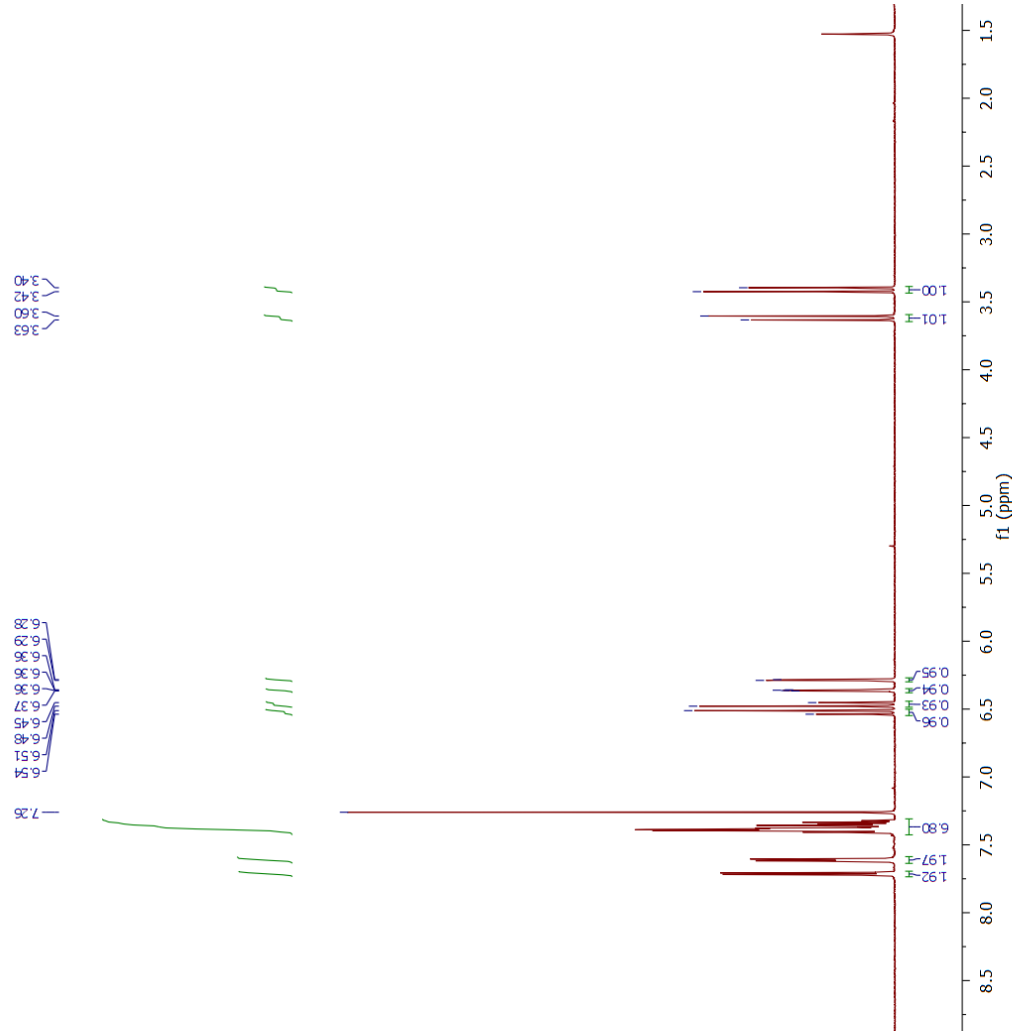

Figure 20. ^{1}H NMR of **11f** ($CDCl_3$, 600 MHz).

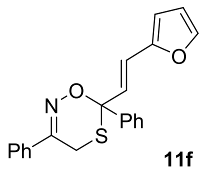

11f

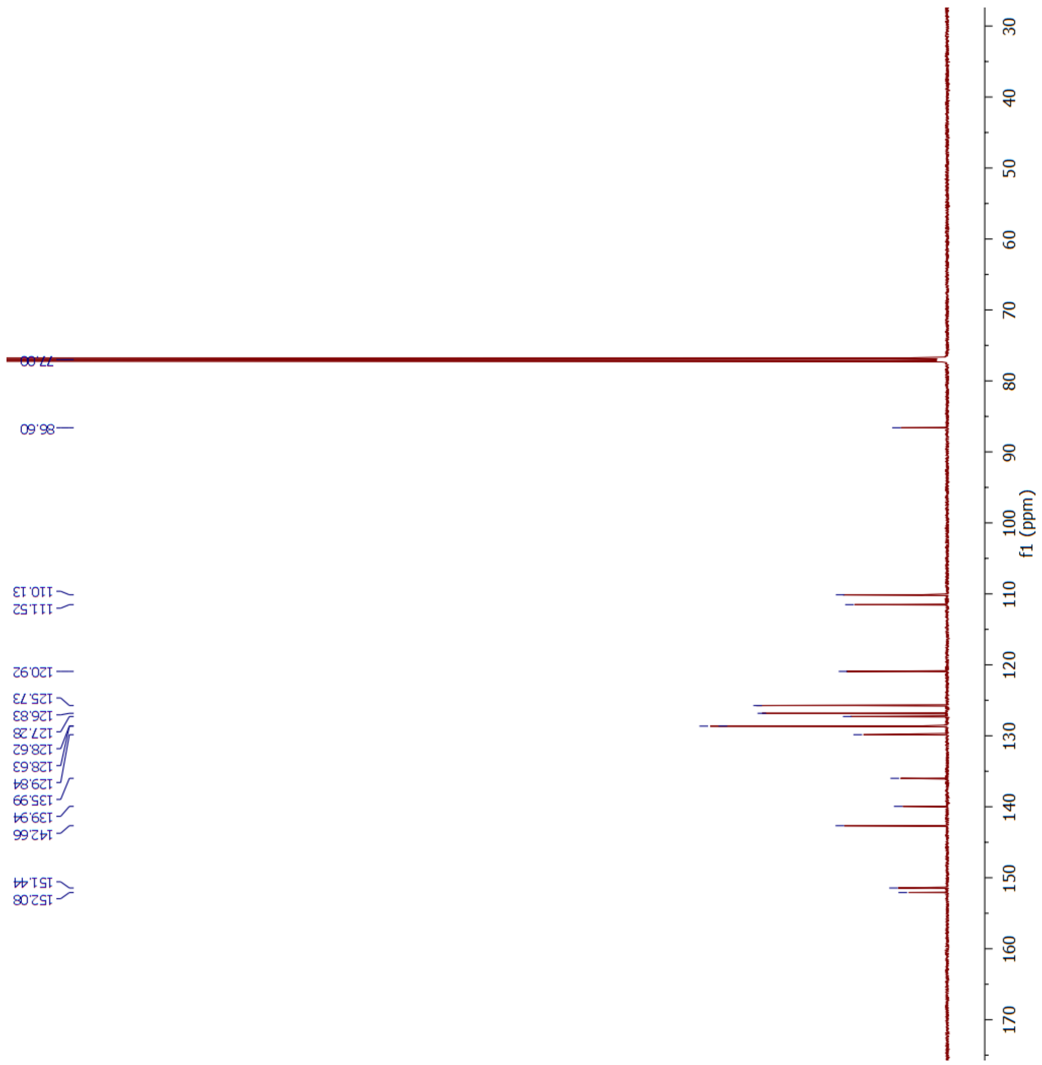

Figure 21. ^{13}C NMR of **11f** ($CDCl_3$, 151 MHz).

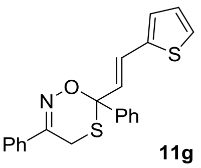

11g

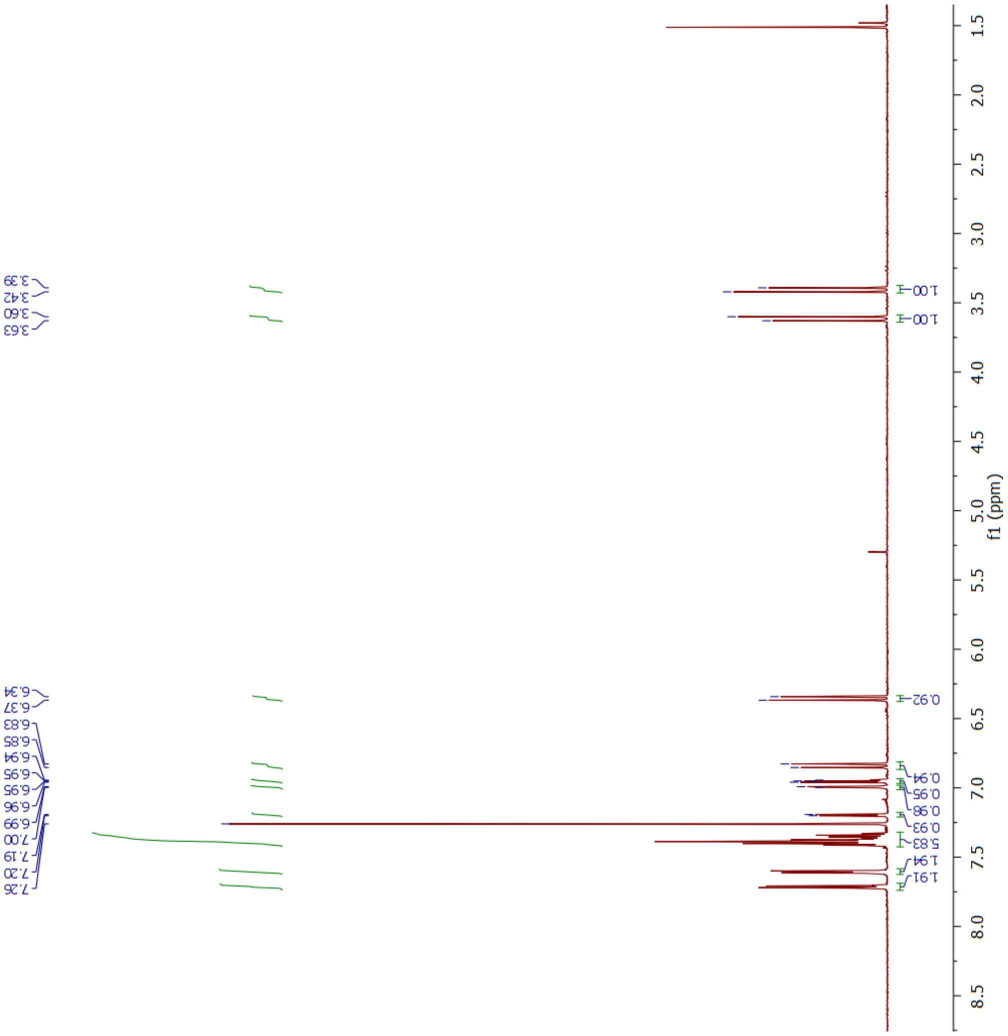

Figure 22. ^{1}H NMR of **11g** ($CDCl_3$, 600 MHz).

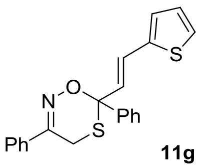

11g

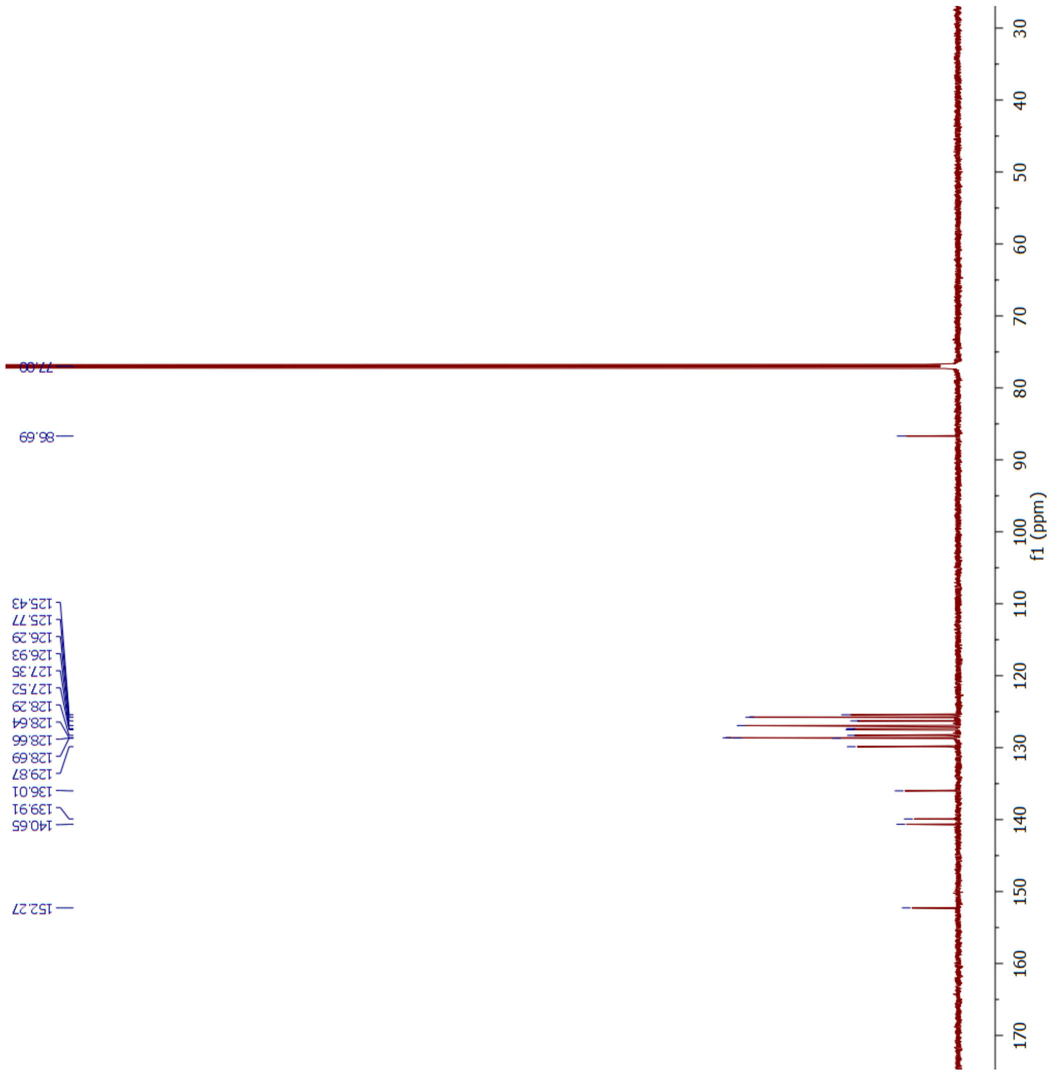

Figure 23. ^{13}C NMR of **11g** ($CDCl_3$, 151 MHz).

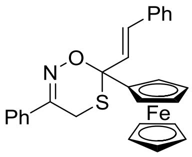

11h

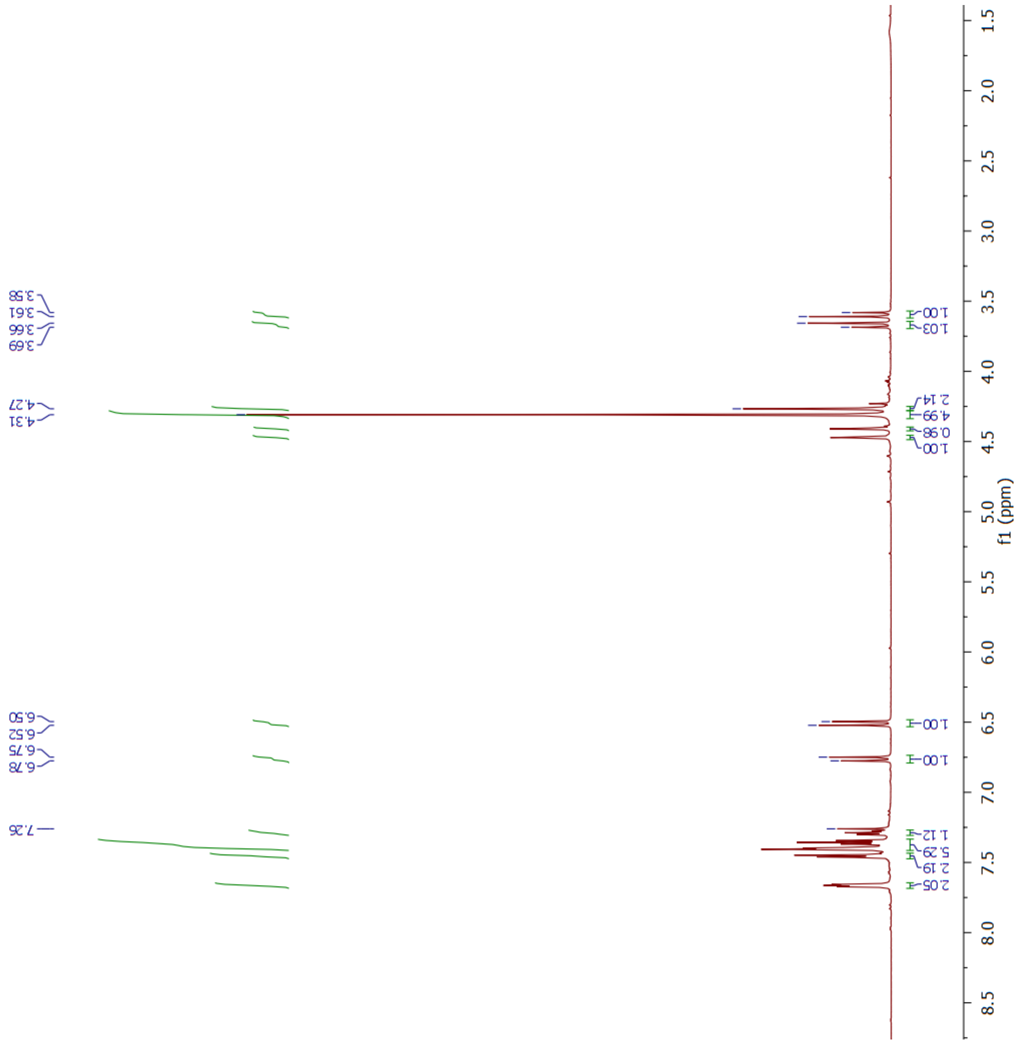

Figure 24. ^{1}H NMR of **11h** ($CDCl_3$, 600 MHz).

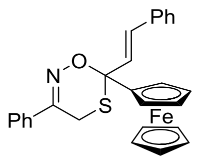

11h

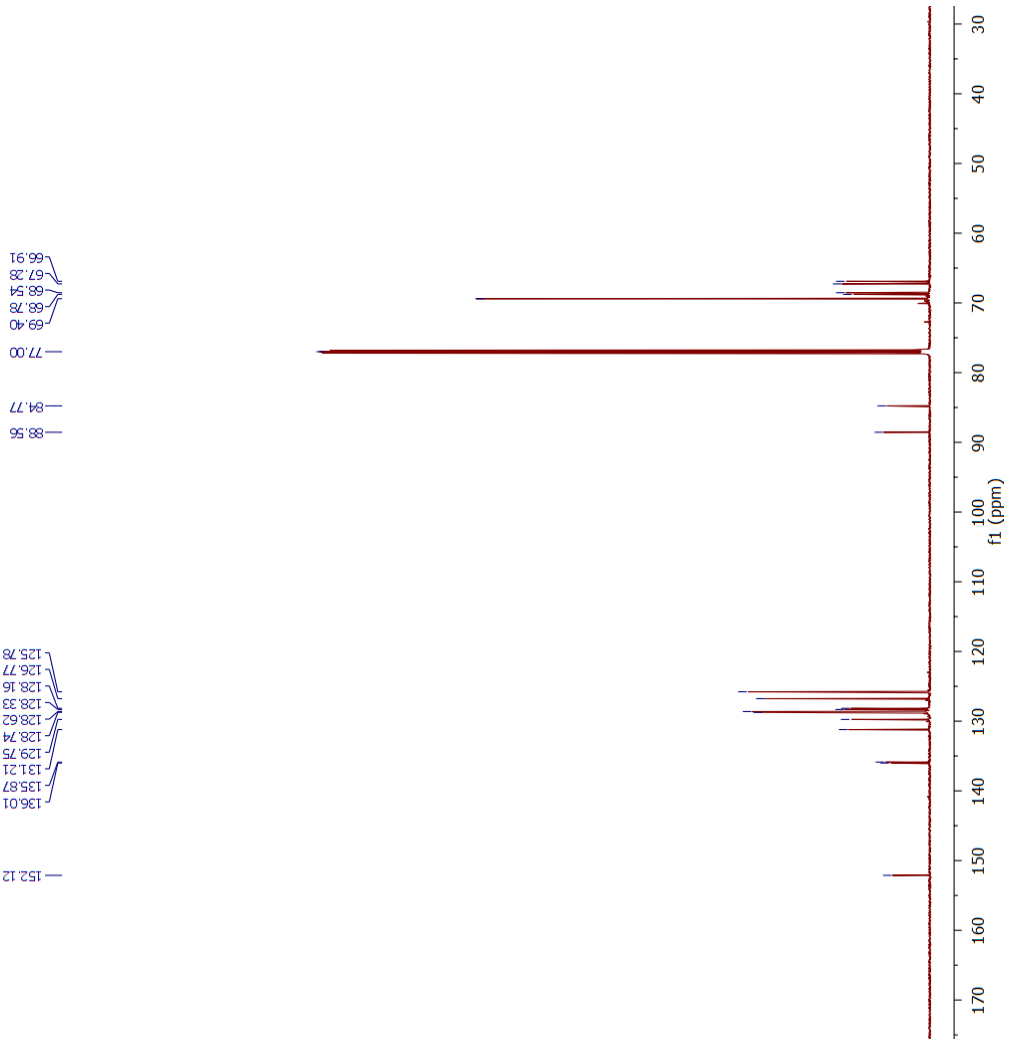

Figure 25. ^{13}C NMR of **11h** ($CDCl_3$, 151 MHz).

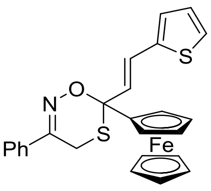

11i

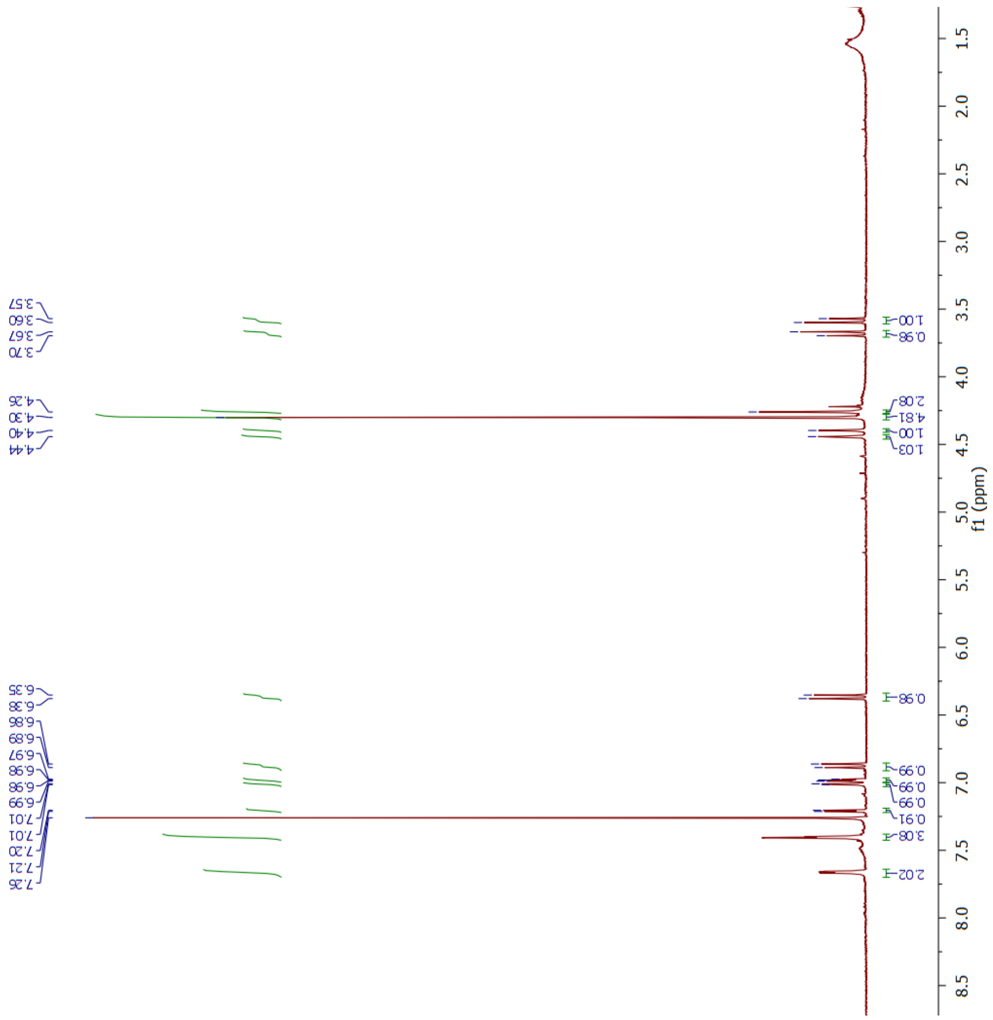

Figure 26. ^{1}H NMR of **11i** ($CDCl_3$, 600 MHz).

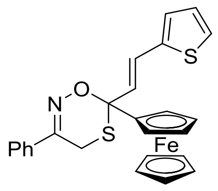

11i

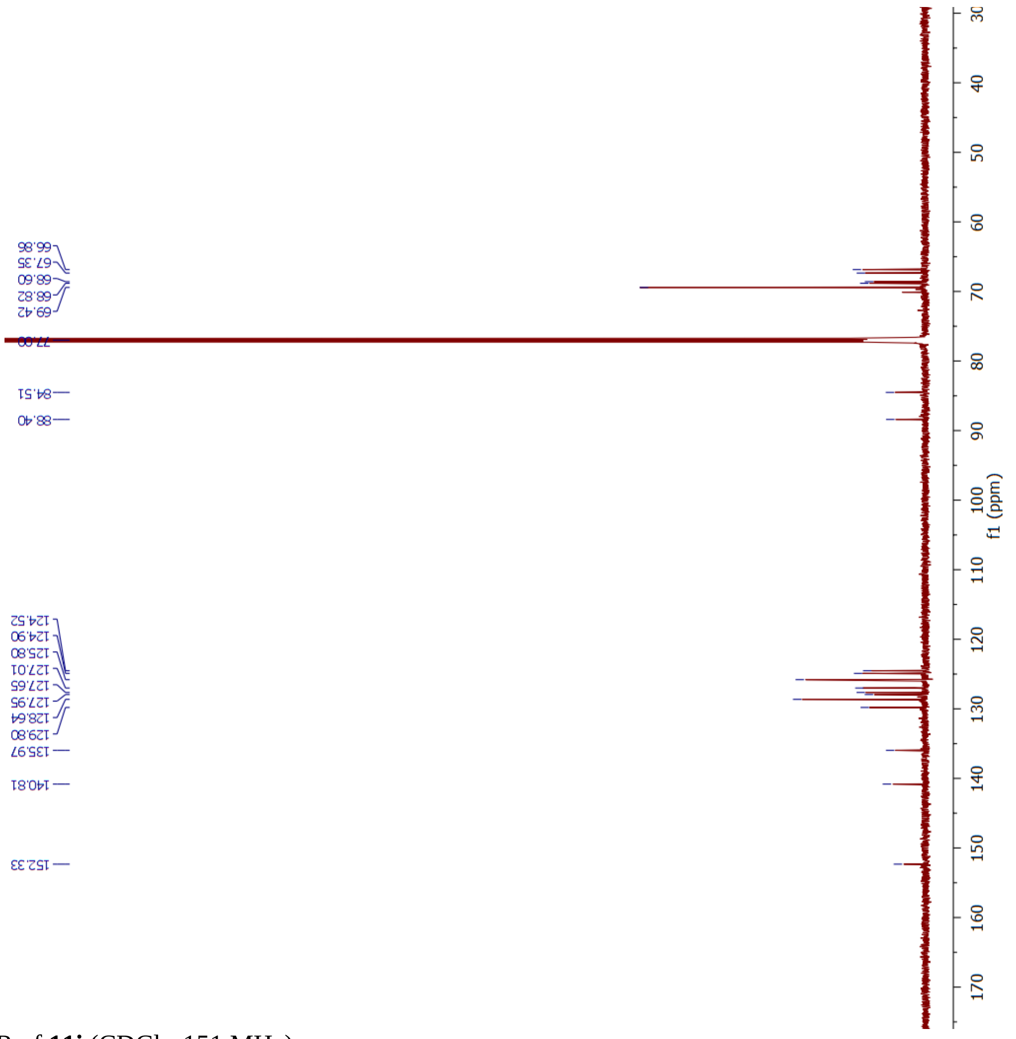

Figure 27. ^{13}C NMR of **11i** ($CDCl_3$, 151 MHz).

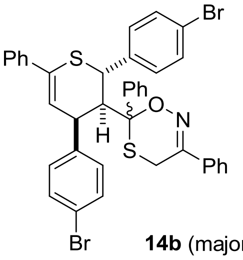

14b (major)

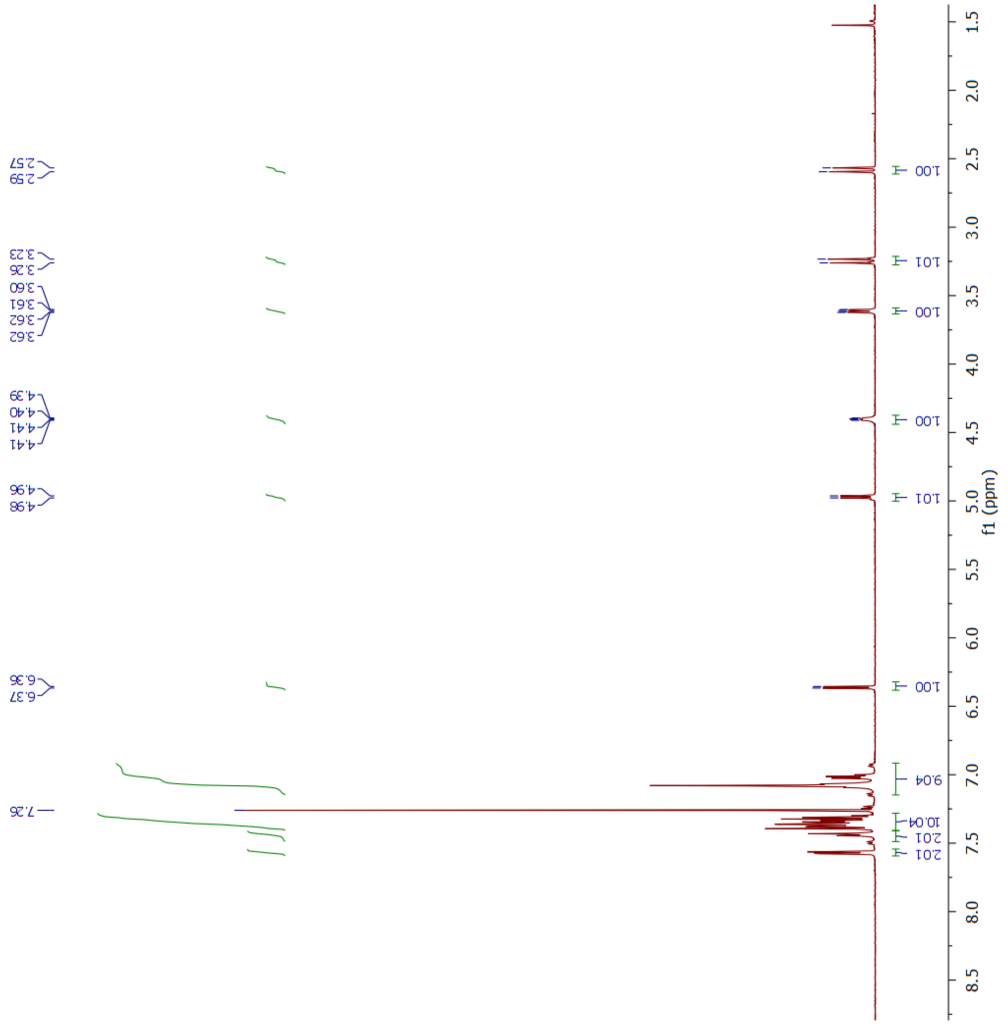

Figure 28. ^{1}H NMR of **14b** (major isomer) ($CDCl_3$, 600 MHz).

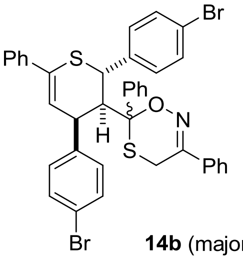

14b (major)

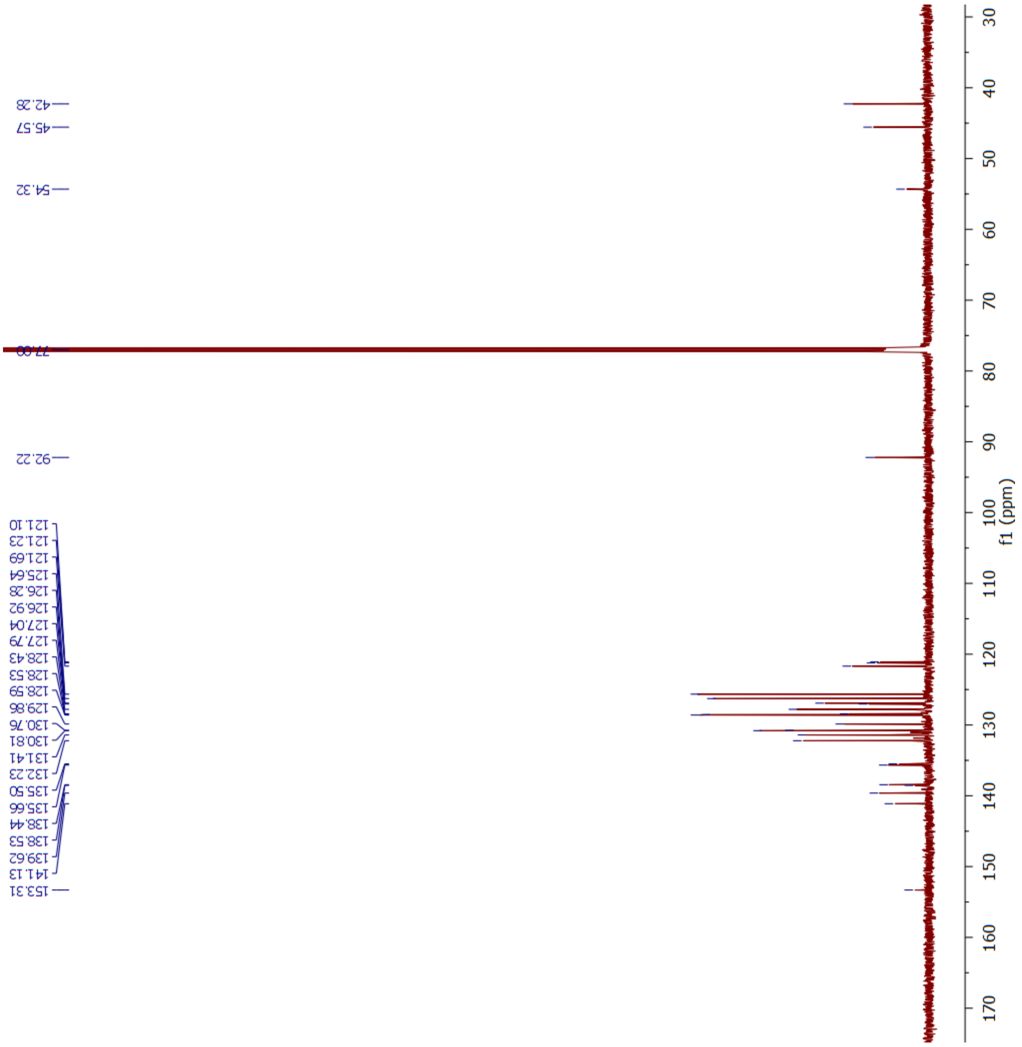

Figure 29. ^{13}C NMR of **11b** (major isomer) ($CDCl_3$, 151 MHz).

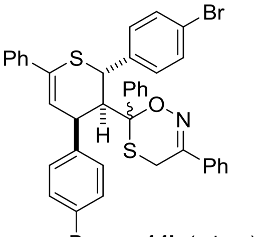

14b (minor)

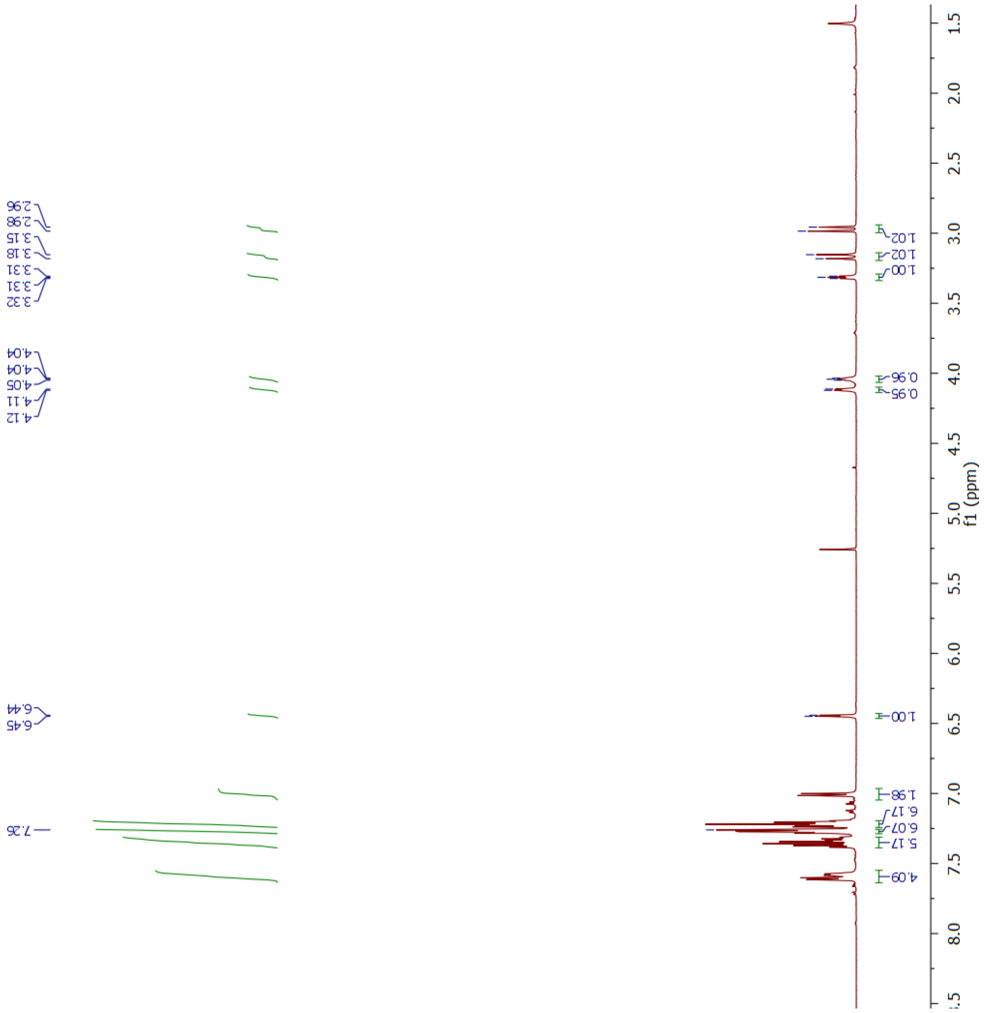

Figure 30. ^{1}H NMR of **11b** (minor isomer) ($CDCl_3$, 600 MHz).

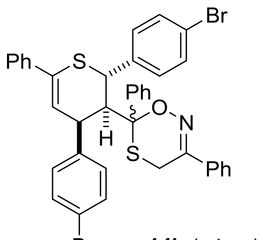

14b (minor)

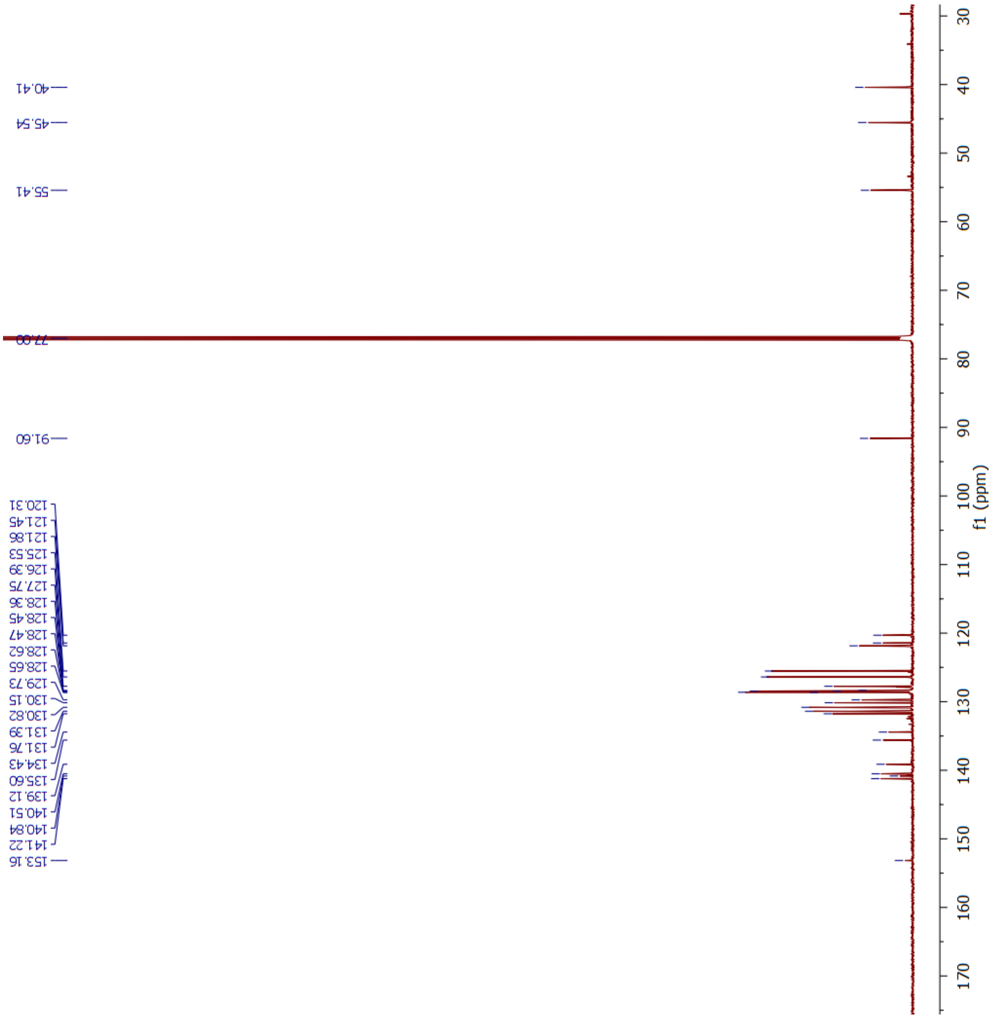

Figure 31. ^{13}C NMR of **14b** (minor isomer) ($CDCl_3$, 151 MHz).

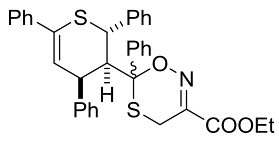

14n (major)

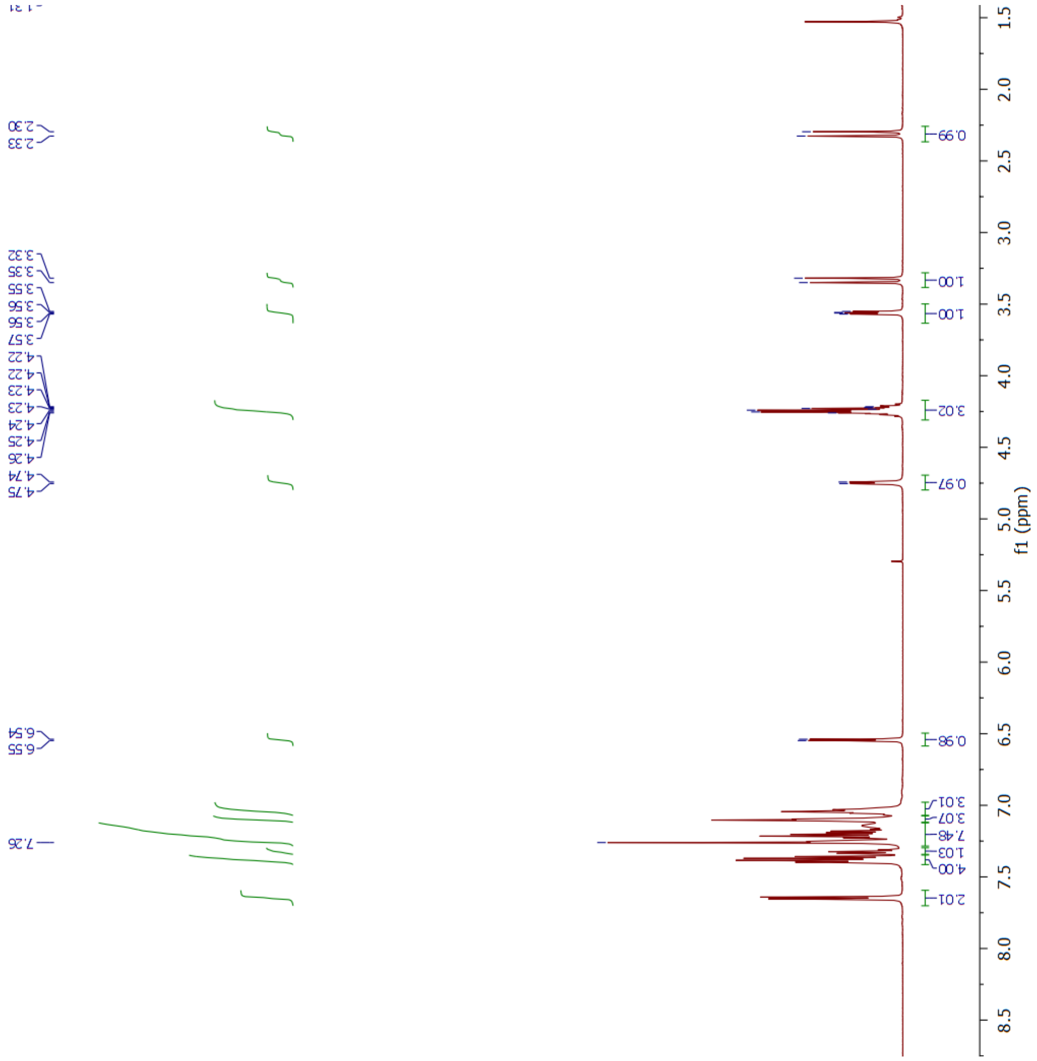

Figure 32. ^{1}H NMR of **11n** (major isomer) ($CDCl_3$, 600 MHz).

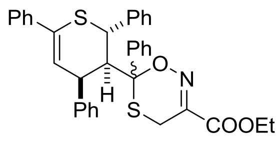

14n (major)

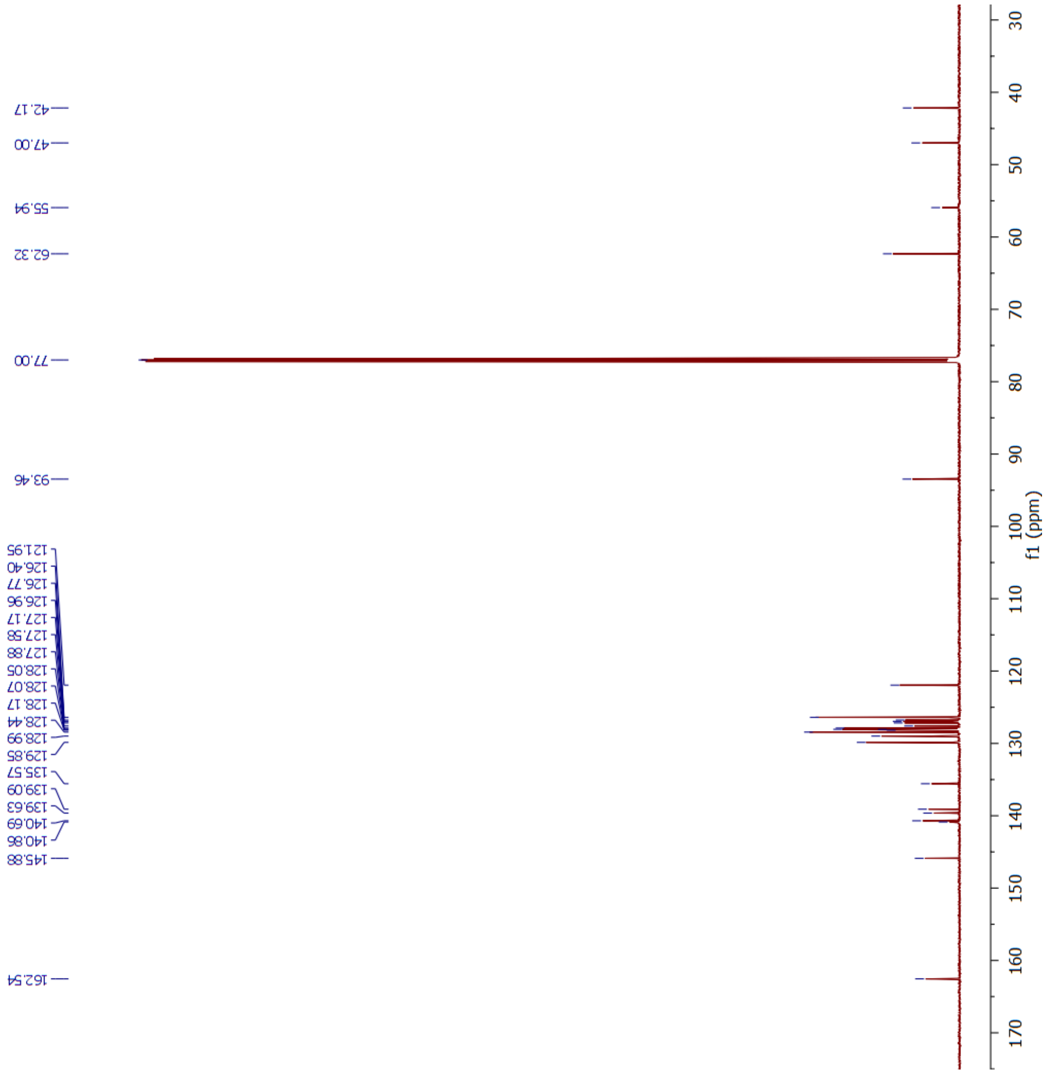

Figure 33. ^{13}C NMR of **14n** (major isomer) ($CDCl_3$, 151 MHz).

4. DFT Calculations

All calculations were performed using the GAUSSIAN 09 D.01 [1] and GAUSSIAN 16, B.01 packages of programs.[2] The structures were fully optimized first using the B3LYP/6-31G(d)[3] + GD3BJ [4] method, then the PBE1PBE/def2tzvp [5] + GD3BJ [4] functional including the PCM-solvent sphere for dichloromethane.[6] Zero point vibrational energies and free enthalpy contributions were determined analytically.[7]

1. Dimers of s-trans-*(E)*-1,3-diphenylprop-2-ene-1-thione (1a)

PBE1PBE/def2TZVP + GD3BJ + PCM (toluene)–Geometry Optimizations

Table S1: Total energies (E_{tot}), energies at 0K E_0), and Gibbs free energies (G_{298})[a.u.], relative energies of the thiochalcone dimers with respect to 2 moles of **1a** and transition state energies also with respect to the relevant heterocyclic ring (last column)[kcal/mol]

Species	E_{tot} [a.u.]	E_{rel} [kcal/mol]	E_0 [a.u.]	E_{rel} [kcal/mol]	G_{298} [a.u.]	To **2*1a** E_{rel} [kcal/mol]	To Ring E_{rel} [kcal/mol]
s-*trans*-thio-chalcone **1a**	-976,40820	-	-976,18464	-	-976,22691	-	
***exo*-4a**	- 1.952,87148	-34,57	-1.952,41969	-31,63	- 1.952,47956	-16,15	
***exo*-4a-TS**	- 1.952,82411	-4,84	-1.952,37587	- 4,13	-1.952,43559	11,44	27,59
***exo*-5a**	- 1.952,86548	-30,80	-1.952,41371	-27,88	-1.952,47414	-12,75	
***exo*-5a-TS**	- 1.952,81924	- 1,79	-1.952,37097	- 1,06	-1.952,43067	14,53	27,28
***endo*-4a**	- 1.952,86533	-30,71	-1.952,41340	-27,68	-1.952,47367	-12,46	
***endo*-4a-TS**	- 1.952,82617	-6,13	-1.952,37814	- 5,55	-1.952,43766	10,14	22,60
***endo*-5a**	- 1.952,86442	-30,14	-1.952,41231	-27,00	-1.952,47327	-12,20	
***endo*-5a-TS**	- 1.952,81551	0,55	-1.952,36740	1,19	-1.952,42749	16,52	28,72
***trans*-2a**	- 1.952,86044	-27,64	-1.952,40994	-25,51	-1.952,47063	-10,55	
***trans*-2a-TS**	-1.952,82724	-6,81	-1.952,37924	- 6,25	-1.952,43876	9,45	20,00
***cis*-2a**	- 1.952,85785	-26,02	-1.952,40785	-24,20	-1.952,46949	-9,83	
***cis*-2a-TS**	- 1.952,82777	-7,14	-1.952,38025	- 6,88	-1.952,44269	6,98	16,81
***trans*-3a**	- 1.952,85513	-24,31	-1.952,40575	-22,88	-1.952,46778	-8,76	-

***trans*-3a-TS**	- 1.952,83090	-9,10	-1.952,38213	- 8,06	-1.952,44040	8,42	17,18
***cis*-3a**	- 1.952,85324	-23,12	-1.952,40383	-21,68	-1.952,46590	-7,58	
***cis*-3a-TS**	-1.952,81538	0,63	-1.952,36819	0,69	-1.952,43086	14,41	21,99

Gaussian Archive Entries (Total energies (a.u.), number of imaginary frequencies (for transition states: imaginary frequencies), coordinates)

s-*trans-(E)*-1,3-diphenylprop-2-ene-1-thione (**1a**)

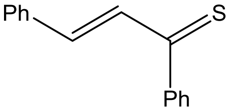

HF=-976.4082058 a.u. (0)

1\1\GINC-R08N35\FOpt\RPBE1PBE\def2TZVP\C15H12S1\WURTHWE\07-Feb-2019\0\\# pbe1pbe/def2tzvp emp=gd3bj Opt=readfc geom=check guess=read Pop=NBO Freq nosym scrf=(solvent=dichloromethane)\\Thiochalcone-s-trans\\0,1\C,0.1631871124,0.332999459,-0.0947356767\C,0.1636457386,0.5134345533,1.2770015296\C,1.2275748333,0.0495247043,2.0521801752\C,2.2808408255,-0.618961281,1.4233732856\C,2.2637969717,-0.8235573223,0.0527990645\C,1.2109289323,-0.3405813882,-0.7105980537\C,1.215042108,0.2250215932,3.5167961881\C,2.4024067428,0.6821994457,4.1920568892\C,3.4422724666,1.2819473099,3.5761314129\C,4.6305789771,1.8169403258,4.2097928336\C,5.5791320149,2.4580093963,3.4053341122\C,6.7357541655,2.9869215909,3.9521533719\C,6.9652546515,2.8824840364,5.31647298\C,6.032399888,2.2461683459,6.1294954541\C,4.8776431488,1.7186961871,5.5851839073\S,-0.1516045997,-0.0936760909,4.3835306489\H,2.3808911831,0.5906470183,5.2720796814\H,3.3978996876,1.410237106,2.4988862406\H,5.3972559496,2.5373415487,2.3388823761\H,7.4590172389,3.4806029113,3.3140187984\H,7.8693051113,3.2947103453,5.7491437161\H,6.2111660689,2.1627503434,7.1950249172\H,4.1632300161,1.2248967891,6.2324391346\H,-0.6557064983,1.0259972083,1.7664210092\H,3.0989384361,-1.0073541497,2.0177176907\H,3.0764940635,-1.3630729908,-0.4192559108\H,1.2038258004,-0.4912417528,-1.7838258856\H,-0.6596841833,0.7156742243,-0.6870899751\\Version=ES64L-G16RevB.01\HF=-976.4082058\RMSD=9.259e-09\RMSF=1.466e-06\Dipole=2.1900988,0.7282603,-0.8869615\Quadrupole=15.4581635,-7.4340797,-8.0240838,7.206405,17.0784344,5.8151068\PG=C01 [X(C15H12S1)]\\@

exo-**4a**

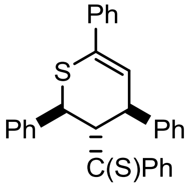

c

HF=-1952.8714807 a.u. (0)

1\1\GINC-R09N03\FOpt\RPBE1PBE\def2TZVP\C30H24S2\WURTHWE\28-Mar-2019\0\\# pbe1pbe/def2tzvp emp=gd3bj Opt=readfc geom=check guess=read nosym Pop=NBO Freq scrf=(solvent=dichloromethane) int=(Acc2e=11,grid=ultrafine)\\Thiochalcone-Dimer1\\0,1\S,-1.2817950338,1.5521932195,-1.4829291505\C,0.3063576932,2.2921820723,-1.5284684339\C,1.0881317193,2.4304221837,-0.4531729239\C,0.8376050243,1.9461588357,0.936589167\C,-0.3647722313,0.9987568127,1.0604786615\C,-1.599346179,1.5225278071,

0.2959581937\C,2.0486465458,2.5708211052,-3.2822163916\C,2.4632775137,3.0092548431,-4.5289380454\C,1.5607500333,3.6167671362,-5.39191017\C,0.2404023858,3.780095624,-4.9980265864\C,-0.1754339055,3.3444616129,-3.7492729862\C,0.7259846234,2.7436327286,-2.8698097849\C,-2.8258059019,0.6810601537,0.5265162113\C,-3.9399104229,1.2311487759,1.1493450738\C,-5.0532830924,0.4493172666,1.4228914038\C,-5.0639885234,-0.892243182,1.0706744126\C,-3.9588119624,-1.4466655873,0.4386244266\C,-2.8479463879,-0.6646358638,0.1676387664\C,2.0662981162,1.2561868942,1.4892106638\C,2.7113290822,0.2719735967,0.743088803\C,3.7982354437,-0.413188073,1.2631022911\C,4.2592439302,-0.1203745487,2.5402249181\C,3.6299288076,0.8648461119,3.2864052093\C,2.5412543053,1.5490400541,2.7623859627\C,-0.7728021273,0.7922580192,2.5013915992\S,-1.131675627,2.0725519121,3.4488114986\C,-0.9011391467,-0.5861507801,2.979313219\C,-0.0201353827,-1.5801756065,2.541570465\C,-0.1340315383,-2.8757370899,3.0158456224\C,-1.15073905,-3.2070675431,3.9005396433\C,-2.0413281031,-2.2300317031,4.3300853065\C,-1.9083124178,-0.928239675,3.8865425229\H,2.0321976305,2.9494477106,-0.592403978\H,0.623775984,2.8100840174,1.578226646\H,-0.1028602135,0.0357837786,0.6193827626\H,-1.7931390711,2.5444137266,0.6297133548\H,2.7491000948,2.0727917199,-2.6217923431\H,3.4934741231,2.8643070072,-4.8334611284\H,1.884163308,3.9546765195,-6.3695816069\H,-0.471243324,4.2533508784,-5.6647449495\H,-1.205344657,3.4852515922,-3.4424849145\H,-3.9273293846,2.2773302228,1.4346048178\H,-5.9126221233,0.8903388238,1.9146145239\H,-5.9315687949,-1.5049680883,1.2864116238\H,-3.9601450727,-2.4939813353,0.1597802035\H,-1.9872058402,-1.104790857,-0.323714281\H,2.3546333954,0.0424425384,-0.2563011725\H,4.2880444829,-1.1771774762,0.6700397334\H,5.1086025622,-0.6552926511,2.9490593191\H,3.9862572893,1.1036295915,4.2819901733\H,2.0488537295,2.3155559076,3.3505119733\H,0.7853135291,-1.3323144802,1.860991294\H,0.5711408641,-3.6306838329,2.6889255805\H,-1.2516971817,-4.2266569245,4.2539848755\H,-2.8436302187,-2.4876200873,5.0113476865\H,-2.599179302,-0.1597224146,4.2103502377\\Version=ES64L-G16RevB.01\HF=-1952.8714807\RMSD=4.165e-09\RMSF=5.467e-07\Dipole=0.3397585,-1.3028212,-0.0156809\Quadrupole=5.7320428,-0.8220641,-4.9099787,1.2315921,-5.5474828,-10.1197685\PG=C01 [X(C30H24S2)]\\@

exo-**4a-TS**

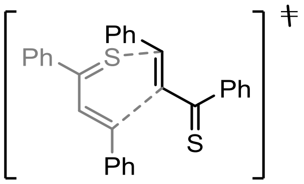

HF=-1952.8241101 a.u. (1, -219.1621cm^{-1})

thiochalcon-dimer1-path-ts_pbe-def2tzvp-gd3bj-ch2cl2-g16.out

1\1\GINC-R01N23\FTS\RPBE1PBE\def2TZVP\C30H24S2\WURTHWE\03-Jan-2019\0\\# pbe1pbe/def2tzvp emp=gd3bj Opt=(ts,noeigentest,readfc) geom=check guess=read nosym Pop=NBO Freq scrf=(solvent=dichloromethane)\\Thiochalcone-Dimer-TS\\0,1\C,-1.3359634724,-0.1755892282,-0.9143709657\C,-0.7256586186,0.0792401214,0.3161991125\C,0.6703971908,0.036663955,0.3788711628\C,1.4245872542,-0.2626999973,-0.7434401963\C,0.8012693992,-0.5293978678,-1.9535213339\C,-0.5844624189,-0.4813060437,-2.0340562997\C,-1.5380434135,0.3918979969,1.5137803\S,-3.1122739834,0.9462297553,1.3721612899\C,-0.9670517592,0.1519913896,2.7810677413\C,-1.5527357397,-2.4887741933,2.7743410906\C,-1.6256389436,-2.7288692241,4.1193462034\C,-2.5599869557,-2.0973510634,4.9683604935\S,-3.3606748159,-0.7303237368,4.4251323719\C,-1.5855883211,0.5543888247,3.9749708997\C,-2.7312425651,-2.5870295069,6.3394159282\C,-2.5624243082,-3.9456649071,6.6272678532\C,-2.7073368001,-4.4141200968,7.9214111019\C,-3.0102111799,-3.5329204356,8.9513484279\C,-3.1776418125,-2.1813890192,8.677183457\C,-3.0482173414,-1.7129922025,7.3822657073\C,-0.5602164919,-3.057247282,1.8898416145\C,-0.8522637761,-3.175125296,0.5271646915\C,0.0713206755,-

3.7143061457,-0.3496411048\C,1.3095953632,-4.1310096252,0.1180374291\C,1.6193026216,-4.009502773,1.4686007921\C,0.6938151016,-3.4806578154,2.3478187616\C,-0.8655518511,0.5209252208,5.2558121718\C,0.1896019316,-0.3594886848,5.4967491634\C,0.8182589794,-0.3860671129,6.7318672594\C,0.4094915465,0.4710119046,7.7433231987\C,-0.6338521818,1.3587518731,7.5124252549\C,-1.2673267985,1.3781976454,6.2823743152\H,-0.9227639602,-3.4186361648,4.574523794\H,-2.384000488,-1.9586499719,2.316960395\H,0.0157773033,-0.2947893277,2.8333810233\H,-2.292347367,1.3741988663,3.8619256279\H,-2.3434220147,-4.6416983132,5.825922643\H,-2.5876925147,-5.471268436,8.1275075981\H,-3.1170018863,-3.8995026651,9.9656693714\H,-3.4059680674,-1.4886925045,9.4787383274\H,-3.164830658,-0.6591605809,7.1632833001\H,0.5236513913,-1.032676506,4.717406265\H,1.633122106,-1.0796351996,6.9041578748\H,0.9027278459,0.4490793476,8.708017495\H,-0.9573193576,2.033829888,8.2961518929\H,-2.093031393,2.0596908262,6.1076171132\H,-1.8160968717,-2.8394634084,0.1640898002\H,-0.1694632971,-3.7970080771,-1.4024052506\H,2.0391541813,-4.5428003829,-0.569505633\H,2.590576034,-4.323310143,1.8325566048\H,0.9508419537,-3.3738835925,3.3953805817\H,1.1812685035,0.2646395129,1.3057018912\H,2.5058097875,-0.2846965408,-0.6715240336\H,1.3918535761,-0.7693262164,-2.8301767433\H,-1.0816392166,-0.6881565228,-2.974879456\H,-2.4171033141,-0.137027681,-0.9686275033\\Version=ES64L-G16RevB.01\HF=-1952.8241101\RMSD=4.380e-09\RMSF=1.309e-06\Dipole=2.535043,-2.3145845,1.3841469\Quadrupole=-25.9008273,-3.843094,29.7439213,-0.7890403,3.9058176,-8.5747925\PG=C01 [X(C30H24S2)]\\@

exo-**5a**

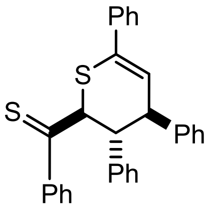

HF=-1952.8654778 a.u. (0)

1\1\GINC-R01N07\FOpt\RPBE1PBE\def2TZVP\C30H24S2\WURTHWE\04-Dec-2018\0\\# pbe1pbe/def2tzvp emp=gd3bj Opt=readfc geom=check guess=read Pop=NBO Freq nosym scrf=(solvent=dichloromethane)\\Thiochalcone-dimer\\0,1\S,0.2798830472,2.8331849171,-0.8007990049\C,-1.1691088919,1.893455285,-1.3750406387\C,-1.0452760887,0.4063099929,-1.0168539011\C,0.0732332946,-0.2404597836,-1.8529848797\C,1.351553096,0.538175908,-1.8408847803\C,1.554586686,1.7783798976,-1.3863852174\C,-2.4130090449,2.581123181,-0.8558392584\S,-3.1830061387,3.6377881032,-1.8290678344\C,-4.2895940972,2.1884067239,0.7078379922\C,-4.7876198528,1.8460796824,1.9503760955\C,-3.9177257878,1.5886309087,3.0026651145\C,-2.5471380455,1.6710606557,2.8008449062\C,-2.0445074081,1.98879181,1.5509074738\C,-2.9107308223,2.256473511,0.4858280714\C,-3.0450071063,-0.2576438573,-2.4089609548\C,-4.2401365009,-0.9418068388,-2.5734357736\C,-4.7525910294,-1.7090110092,-1.5365953387\C,-4.061462115,-1.7873083192,-0.3356344427\C,-2.8694804324,-1.0984721992,-0.1744143203\C,-2.3479395826,-0.3259384017,-1.206044506\C,2.8974444289,2.389967563,-1.334757379\C,3.9957875714,1.621869875,-0.9441277379\C,5.2662928,2.1724885038,-0.9111591992\C,5.459834148,3.5040095101,-1.2545125554\C,4.372702182,4.280067135,-1.63008167\C,3.1008136396,3.729761879,-1.6672445246\C,0.3087576708,-1.6556262709,-1.3761781431\C,-0.1760882407,-2.740079075,-2.097163793\C,0.0013562396,-4.0360760495,-1.6329079744\C,0.6682270077,-4.2617872201,-0.4376900492\C,1.1593338668,-3.1842528304,0.2881227986\C,0.9810879594,-1.8917593401,-0.1800819793\H,-1.1813639495,2.0086807996,-2.4599388848\H,-0.7622586447,0.3225261791,0.0341105146\H,-0.283113866,-0.3059171105,-2.8891068578\H,2.2153987885,0.0158642595,-2.2419756163\H,-4.9600247629,2.3766047824,-0.1214441403\H,-5.8584446563,1.7737756749,2.0991180654\H,-4.3082115344,1.3238188383,

3.9783589832\H,-1.8639102109,1.4851792127,3.621057421\H,-0.9741252979,2.0660996092,
1.4069844496\H,-2.6586596915,0.3390723385,-3.2286699569\H,-4.7734696873,-0.8738975517,-
3.5147359699\H,-5.6872492605,-2.2427575755,-1.6642744221\H,-4.4534682265,-
2.3835501265,0.4803857287\H,-2.3352061577,-1.1535598324,0.7679064811\ H,3.8428897181,
0.589886557,-0.6501498363\H,6.1077951437,1.562432143,-0.6034750381\H,6.4532544127,
3.9360746225,-1.2228464538\H,4.5145859854,5.3200239543,-1.9005995149\H,2.2574039793,
4.337415702,-1.973975827\H,-0.7065595553,-2.5659791891,-3.0273393284\H,-0.3846986903,-
4.8710950059,-2.2064606581\H,0.8074487024,-5.2730668741,-0.0732854929\H,1.6844186212,-
3.3520713679,1.221700343\H,1.3695333876,-1.0523458869,0.3881484149\\Version=ES64L-
G16RevB.01\HF=-1952.8654778\RMSD=5.165e-09\RMSF=8.194e-07\Dipole=0.2909664,-
1.4926144,0.8470989\Quadrupole=8.5601121,-11.2495476,2.6894355,11.0316735,-
3.3392308,2.7193482\PG=C01 [X(C30H24S2)]\\@

exo-**5a-TS**

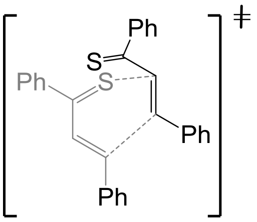

HF=-1952.8192426 a.u. (1, -364.0294 cm^{-1})

\1\GINC-R09N13\FTS\RPBE1PBE\def2TZVP\C30H24S2\WURTHWE\10-Aug-2019\0\\ #
pbe1pbe/def2tzvp emp=gd3bj Opt=(ts,noeigentest,readfc) geom=check guess=read
scrf=(solvent=dichloromethane) nosym freq pop=nbo\\Thiochalcone-Dimer:
TS\\0,1\C,0.2690304783,0.4570204634,0.6509701214\C,-0.3025656084,0.0138813438,
1.8455192203\C,0.262331839,-1.0895029212,2.4851998222\C,1.3441281623,-
1.7509433243,1.9311358207\C,1.8976742554,-1.3094920593,0.735956003\C,1.3599479231,-
0.1981614007,0.102533562\C,-1.4685775628,0.7167730284,2.4181662645\S,-1.8317485096,
0.6335006201,4.0485379574\C,-2.3771976902,1.3661949181,1.5720596334\C,-3.4387105478,
2.1423384534,2.0542993424\C,-4.2544884219,2.9440194691,1.139067043\C,-4.6055287029,
2.4756408978,-0.1292830895\C,-5.3963617561,3.2423573506,-0.9672611476\C,-5.8467848919,
4.491348822,-0.5548164823\C,-5.5093547038,4.9629902096,0.7052119117\C,-4.7274745617,
4.1902177882,1.5493647263\S,-3.5643774623,-1.3111839006,1.2622894683\C,-3.6988146173,-
1.3288444199,2.9124424417\C,-4.1872554029,-0.1683091393,3.597606591\C,-4.8675057941,
0.8540933283,2.9126917327\C,-5.7022255718,1.8077626195,3.6450331759\C,-5.3513268387,
2.2664317309,4.917611931\C,-6.1472372027,3.186678687,5.5778648202\C,-7.3093871034,
3.6622366114,4.981793669\C,-7.664568781,3.2175170872,3.7164678137\C,-6.8623669706,
2.3051404847,3.0501328582\C,-3.268085065,-2.4929418027,3.7143149648\C,-2.2861562068,-
3.3623469794,3.2368429251\C,-1.877826784,-4.4493043196,3.9880545179\C,-2.4604616905,-
4.7037638211,5.2235361031\C,-3.4539267115,-3.8605946623,5.6995111788\C,-3.8503421493,-
2.7609371532,4.9553390233\H,-2.246450892,1.2660696484,0.5019355636\H,-3.2874885658,
2.5469673727,3.0517595744\H,-5.1987418992,0.5817475031,1.9151449051\H,-4.2672511485,-
0.210866557,4.6755202366\H,-0.1689127178,-1.4287581822,3.4187861236\H,1.7583834246,-
2.6167804588,2.4350204658\ H,2.7486620083,-1.8241952811,0.3052049292\H,1.7949354151,
0.1655307931,-0.8212209982\H,-0.1264095269,1.3357158957,0.1551094032\H,-4.2725653012,
1.4940449776,-0.4490285457\H,-5.6665458499,2.8645271764,-1.9466170898\H,-6.4638188326,
5.0911113723,-1.2137619019\H,-5.8636586034,5.9320943978,1.0368073263\H,-4.4764851284,
4.5517013973,2.5405133142\H,-4.6406092433,-2.1253664003,5.3359811083\H,-3.9263057404,-
4.0599238008,6.654292128\H,-2.1448953424,-5.5589850791,5.8097508687\H,-1.1008053023,-
5.1027573992,3.6084872938\H,-1.8401146475,-3.1612607563,2.2709212025\H,-7.1323802587,
1.9688662098,2.0551238003\H,-8.5656468487,3.588552084,3.242015266\H,-7.9327907558,

4.3800618802,5.5020936924\H,-5.8600961585,3.5372761307,6.5625483214\H,-4.4379902436,
1.9124648445,5.3831901071\\ Version=ES64L-G16RevB.01\HF=-1952.8192426\RMSD=5.588e-
09\RMSF=1.967e-06\Dipole=-0.6241381,0.6358813,0.1553623\Quadrupole=1.8483427,-1.2901895,-
0.5581532,-19.025404,-9.1031325,-3.761435\PG=C01[X(C30H24S2)]\\@

endo-**4a**

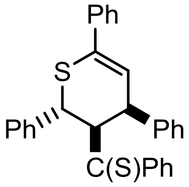

HF=-1952.8653281a.u. (0)

1\1\GINC-R02N03\FOpt\RPBE1PBE\def2TZVP\C30H24S2\WURTHWE\01-Mar-2019\0\\#
pbe1pbe/def2tzvp emp=gd3bj Opt=readfc geom=check guess=read scrf=(solvent=dichloromethane)
nosym Pop=NBO Freq\\Thiochalcone-Dimer1a\\0,1\C,-0.1669256134,0.1854436063,0.0734510749\
C,-0.1016491936,-0.2980373724,1.3846944893\C,0.9184644227,-1.1969869842,1.7124293805\
C,1.8505511402,-1.5842139199,0.7637181155\C,1.76487353,-1.1052776929,-0.5351762157\
C,0.7487825122,-0.2214932876,-0.8782073682\C,-1.093079934,0.1440295506,2.3790852543\S,-
1.7504114437,1.633114178,2.3250976767\C,-1.5419318152,-0.8856522557,3.3780052821\C,-
2.6259035552,-1.7860915938,2.7048328099\C,-2.9753680376,-2.9406205035,3.5850600226\C,-
2.9074452761,-2.9620108001,4.9182775134\S,-2.3657359471,-1.6195674529,5.9080572384\C,-
2.0107375621,-0.2976008504,4.7026549384\C,-3.299069648,-4.1481588823,5.7068311884\C,-
4.4366260117,-4.8748806916,5.3537256723\C,-4.8044012028,-6.002453756,6.0701059644\C,-
4.0482050701,-6.4166688775,7.1581545405\C,-2.9225687535,-5.6929462831,7.5254715344\C,-
2.551985278,-4.5656927628,6.8087446439\C,-3.8575167951,-1.0379111807,2.2474829154\C,-
4.9006897513,-0.751253229,3.12451129\C,-6.0118176964,-0.0414350858,2.6941882037\C,-
6.1050247276,0.3780171199,1.3744825189\C,-5.0811643103,0.0789292076,0.4870602921\C,-
3.9674692495,-0.6224903715,0.9232338612\C,-1.0113647361,0.6091119322,5.3709225574\
C,0.315795555, 0.2248916053,5.5396955817\C,1.2232966304,1.0744888111,6.1522500575\
C,0.8109989535,2.3159190599,6.6185437597\C,-0.5137593251,2.6993771763,6.4696746553\C,-
1.4175416612,1.8494230886,5.8485410386\H,-3.3154720212,-3.8398119234,3.0811582962\H,-
2.1499904312,-2.1937088437,1.8076676793\H,-0.7129679002,-1.5672042866,3.5775726138\H,-
2.9308025097,0.2633241908,4.5369234776\H,-5.0427948686,-4.5392388482,4.5203451988\H,-
5.6928180518,-6.5536266968,5.7838310227\H,-4.3388970922,-7.295868584,7.7212466417\H,-
2.3258937075,-6.0087903058,8.3734803788\H,-1.6651119596,-4.0119220708,7.0951391662\
H,0.6449672857,-0.7505069182,5.1989340975\H,2.2553328596,0.7647337529,6.269910635\
H,1.5203806782,2.9801998328,7.0983197983\H,-0.8451688296,3.6654805714,6.8325590419\H,-
2.4495646083,2.1555905868,5.7172320845\H,-4.8499123716,-1.0977056848,4.1507307486\H,-
6.8122513963,0.1774791652,3.3917577643\H,-6.9761848267,0.9276386258,1.0371396722\H,-
5.1482973202,0.393566907,-0.5481490342\H,-3.1682173719,-0.8517842283,0.2258755983\
H,1.0189296768,-1.571260473,2.7230890895\H,2.6474431351,-2.263405437,1.0423772803\
H,2.487265217,-1.4196216086,-1.279336012\H,0.6705329397,0.1498220128,-1.8932373928\H,-
0.9607646782,0.8755970157,-0.1846846541\\Version=ES64L-G16RevB.01\HF=-1952.8653281\
RMSD=4.558e-09\RMSF=9.233e-07\Dipole=0.7073489,-1.0392765,-0.8705558\Quadrupole=
4.4799136,2.0741636,-6.5540772,0.2379312,6.3896982,-4.7043372\PG=C01 [X(C30H24S2)]\\@

endo-**4a-TS**

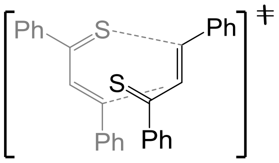

HF=-1952.8261657 a.u. (1, -205,5507cm^{-1})

1\1\GINC-R01N09\FTS\RPBE1PBE\def2TZVP\C30H24S2\WURTHWE\22-Dec-2018\0\\# pbe1pbe/def2tzvp emp=gd3bj Opt=(ts,noeigentest,calcfc) nosym pop=nbo freq scrf=(solvent=ch2cl2)\\Thiochalcone-Dimer TS\\0,1\C,0.0824532228,0.0233593041,0.0334599475\C,0.0597658175,0.071260407,1.4285324369\C,1.2790662161,0.1367748866,2.1080557855\C,2.4775586293,0.1611749353,1.4146037386\C,2.4828983863,0.1270090255,0.0277077394\C,1.2789923178,0.0561944194,-0.660236241\C,-1.225984349,0.0651638826,2.1611848711\S,-2.6070379716,-0.5738336739,1.4708477605\C,-1.2639812938,0.6936348138,3.4235129952\C,-2.3898361306,0.6722187247,4.2478429793\C,-2.3457905616,1.1058238983,5.6499964482\C,-3.2867249867,0.5905988941,6.5420962689\C,-3.2830959066,0.9697094,7.8748567161\C,-2.3402104299,1.8772945474,8.3357452318\C,-1.401489999,2.4014272151,7.4554873634\C,-1.4043652781,2.0199709573,6.125153726\S,-4.0160841685,2.1105568046,3.4677673053\C,-3.6911261376,2.3043355574,1.8333004851\C,-4.7556362926,2.0878248885,0.8360082895\C,-5.7690593161,1.1504010206,1.0441000169\C,-6.7677569247,0.9742047513,0.1054987712\C,-6.7842095752,1.7431612992,-1.053491392\C,-5.7905557632,2.6866301617,-1.2647327091\C,-4.7806983406,2.8552835648,-0.3296083857\C,-2.4161130166,2.7521068618,1.4011720443\C,-1.4420618352,3.1532653173,2.2620133585\C,-0.0861474092,3.5247377192,1.9242988807\C,0.4479727571,3.382151419,0.6381368544\C,1.7613459143,3.7231309218,0.3819415858\C,2.5705225318,4.2148730305,1.4002094363\C,2.0553858639,4.3671311562,2.6802376331\C,0.7398029975,4.0250369294,2.9376183974\H,-3.1067013613,-0.1125039286,4.0168158984\H,-0.3649408386,1.177558149,3.7812056266\H,1.2980019424,0.1433233068,3.190847408\H,3.4121862076,0.2050962123,1.9615566411\H,3.4210972982,0.1535196698,-0.5144184105\H,1.2726619196,0.0319740246,-1.7439486485\H,-0.8613660193,-0.0297044094,-0.4954336416\H,-1.7352391804,3.2928636398,3.2990903093\H,-0.6673944236,2.4413127094,5.4521665685\H,-0.6639097577,3.1125917023,7.8088670293\H,-2.3359294181,2.1772264469,9.3771058324\H,-4.0184529132,0.5558657476,8.554895315\H,-4.0261473263,-0.1162801749,6.1810059425\H,-5.7437447428,0.5482824578,1.9439482823\H,-7.5376235997,0.2300477461,0.2736220927\H,-7.5710134114,1.6068884563,-1.7862838514\H,-5.8015770445,3.2992633193,-2.158684181\H,-4.0212542422,3.6102595022,-0.4951004945\H,-2.2076444668,2.684642846,0.3396447619\H,0.3346721643,4.1455843683,3.9367732825\H,2.6806061044,4.7513139526,3.4774990005\H,3.6021698507,4.4758065617,1.1949574326\H,2.1637129474,3.5961079098,-0.6159104445\H,-0.1624346564,2.9842897439,-0.1619600903\\Version=ES64L-G16RevB.01\HF=-1952.8261657\RMSD=5.322e-09\RMSF=1.330e-06\Dipole=1.801466,1.5530695,0.1647912\Quadrupole=-3.9917589,-9.5125236, 13.5042824, 8.4215742,8.3116733,3.5546263\PG=C01 [X(C30H24S2)]\\@

endo-**5a**

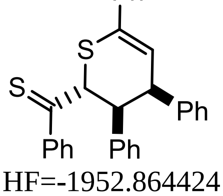

HF=-1952.8644241 a.u. (0)

1\1\GINC-R07N19\FOpt\RPBE1PBE\def2TZVP\C30H24S2\WURTHWE\02-Apr-2019\0\\# pbe1pbe/def2tzvp emp=gd3bj Opt=readfc geom=check guess=read scrf=(solvent=dichloromethane)

nosym Pop=NBO Freq int=(Acc2e=11,grid=ultrafine)\\Thiochalcone-Dimer 4f-pcm2\\0,1\C,0.1051688402,1.2535186399,0.7059768591\C,-0.0678841195,0.1184734892,1.504533676\C,1.0501928821,-0.6661939323,1.8105961636\C,2.298986947,-0.333663188,1.3143507982\C,2.4579742905,0.8030523341,0.5345207455\C,1.3574371437,1.5985443218,0.2361501632\C,-1.3979179859,-0.2434028538,2.0023617514\S,-2.7679597476,0.1283495789,1.2039520278\C,-1.4486858512,-0.9940773984,3.3175445735\C,-2.4065957018,-0.3653489181,4.3290205777\C,-2.3290175449,-1.0892924777,5.6952575841\C,-2.6289004604,-2.5474077295,5.5752069143\C,-2.4833670628,-3.2994572082,4.48207306\S,-1.9016766596,-2.7122273311,2.9330112009\C,-2.1822335125,1.1182427727,4.4747510542\C,-0.9103110283,1.6844236975,4.4895050247\C,-0.7451590004,3.0526274447,4.6452904804\C,-1.8499294249,3.8775813742,4.7977655898\C,-3.1225462092,3.3235623024,4.7892694906\C,-3.2828413065,1.9557375203,4.6256442716\C,-1.0298487362,-0.8463803188,6.4329316385\C,-0.932600931,0.1981971151,7.3482914974\C,0.2633170598,0.4696186814,7.9944052426\C,1.3869158025,-0.3024557094,7.7326539535\C,1.2992817424,-1.3550490038,6.8329879006\C,0.0975211478,-1.6289421929,6.1950538014\C,-2.7970130955,-4.7428986522,4.4659361495\C,-3.4578996361,-5.3266937685,3.3846678624\C,-3.7611597452,-6.679362255,3.3908360049\C,-3.4041883668,-7.4710203449,4.4731585792\C,-2.7374962627,-6.9009786112,5.549245065\C,-2.4311233067,-5.5496530755,5.5437873351\H,-0.4507685428,-1.0135192865,3.7537973248\H,-3.4214732986,-0.5107881144,3.9507011031\H,-3.12327031,-0.6421986494,6.3020572173\H,-2.9988716564,-3.036174054,6.4713168539\H,-3.7528134872,-4.712461304,2.5412688931\H,-4.2831673642,-7.1152229742,2.5468240055\H,-3.6392531421,-8.5289763308,4.4758671582\H,-2.44384542,-7.5140155736,6.3936269507\H,-1.8889440479,-5.1121455795,6.3740095798\H,0.0317428013,-2.4712088952,5.514766775\H,2.167422525,-1.9724209116,6.6317037276\H,2.323797229,-0.0903981001,8.2347707657\H,0.3190487154,1.2890154137,8.7020164645\H,-1.8044148354,0.8126750126,7.5461833968\H,-4.280041177,1.5278722283,4.605926786\H,-3.9939727984,3.9585507594,4.9015839771\H,-1.7205312723,4.946833087,4.9191857425\H,0.2536230362,3.4739227482,4.6507482613\H,-0.0318748685,1.0585827244,4.3856041217\H,0.9416959748,-1.565526445,2.4050830438\H,3.1513233746,-0.9630831079,1.5404294158\H,3.4392770637,1.071869976,0.1612827334\H,1.4802191131,2.4931094283,-0.3627657894\H,-0.7582908532,1.8679489371,0.4830473108\\Version=ES64L-G16RevB.01\HF=-1952.8644241\RMSD=2.065e-09\RMSF=5.857e-07\Dipole=1.3581275,0.3235828,0.9439029\Quadrupole=-5.4333589,2.6018941,2.8314647,1.0216133,0.8205324,-0.7340864\PG=C01 [X(C30H24S2)]\\@

endo-**5a-TS**

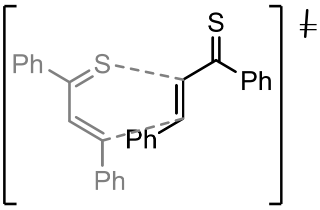

HF=-1952.8155133 a.u. (1, -363.5566 cm^{-1})

1\1\GINC-R07N17\FTS\RPBE1PBE\def2TZVP\C30H24S2\WURTHWE\02-Apr-2019\0\\# pbe1pbe/def2tzvp emp=gd3bj Opt=(ts,noeigentest,readfc) geom=check guess=read scrf=(solvent=dichloromethane) pop=nbo freq nosym\\Thiochalcone-Dimer: TS\\0,1\C,-0.0188831991,-0.0703527676,-0.0537447679\C,0.000899178,-0.0173471424,1.3441981231\C,1.2405013094,-0.0383367801,1.9939375857\C,2.4168468583,-0.1014647507,1.2685817461\C,2.3822754832,-0.152814836,-0.1199466205\C,1.1594382264,-0.1424330398,-0.7774936247\C,-1.2492078613,0.1054788486,2.0717125449\C,-1.3735492672,-0.294236378,3.4093354298\C,-2.6804324243,-0.5548027928,4.0126569856\C,-2.6714865894,-1.2775352578,5.2972053589\C,-1.7392308888,-2.2887779216,5.5459523249\C,-1.7540094275,-2.9787955461,6.7467087209\C,-2.677866286,-2.6492780064,7.7280234958\C,-3.6005278656,-1.6365040934,7.4965683286\C,-3.6057963536,-0.9656874917,6.287595703\S,-4.0862636423,-0.0453219701,3.3330369509\S,-

0.8858285099,1.4305316944,4.9215739391\C,0.0943524339,2.3777961475,3.9369933979\C,1.46845
53247,2.7299957202,4.3525199385\C,2.0467476292,3.9216091528,3.9111368047\C,3.3412480943,4
.2571439737,4.2747679284\C,4.0836199924,3.4058252628,5.0807166889\C,3.5149931571,2.221136
8452,5.5306016859\C,2.2174771339,1.8912389595,5.1782264407\C,-0.3465913585,2.7195722929,
2.6625174026\C,-1.5707736449,2.312553328,2.1486186405\C,-2.0104915254,2.6326874853,
0.7962410346\C,-3.3752665969,2.5692596457,0.4965842607\C,-3.8360663182,2.8483024565,-
0.7779108834\C,-2.9391641555,3.1884991122,-1.7830211079\C,-1.5806133985,3.2450254841,-
1.5025596676\C,-1.1182998433,2.9676354589,-0.2270385533\H,-2.1643096827,0.0925661406,
1.4904520515\H,-2.3560581766,2.0696008888,2.8628821089\H,0.3700136075, 3.1901512975,
1.9990620362\H,-0.0536612835,2.9994695072,-0.032017715\H,-0.8751842992,3.501368787,-
2.2845029796\H,-3.2978781342,3.4056486035,-2.7824488704\H,-4.8977881556,2.7991607096,-
0.9902021186\H,-4.0703439148,2.2916133731,1.2810800442\H,-0.5381740878,-0.8220074064,
3.8529235709\H,-4.3224765916,-0.1765916323,6.0961526944\H,-4.3180272353,-1.3691770705,
8.2633264624\H,-2.6790134318,-3.1801509785,8.6728649514\H,-1.0400185243,-3.7760054693,
6.9162001908\H,-1.0220325273,-2.569415086,4.7842790567\H,1.7703189874,0.9714508398,
5.5365610343\H,4.0861001802,1.5498016806,6.1616188893\H,5.0968795296,3.6668696523,5.3629
342347\H,3.7692961446,5.191550485,3.9304281299\H,1.4707217107,4.6032814976,3.2960085401\
H,1.2870657494,0.0108712977,3.0749346128\H,3.3676504687,-0.1076103817,1.7891973537\
H,3.3051119286,-0.2041336825,-0.6859069376\H,1.1237033993,-0.1862635224,-1.8599725122\H,-
0.9729717827,-0.0559210181,-0.5684380053\\Version=ES64L-G16RevB.01\HF=-1952.8155133\
RMSD=4.483e-09\RMSF=1.404e-06\Dipole=1.4980027,-0.2056451,-0.5873971\Quadrupole=-
3.4506335,-1.9534716,5.4041051,2.5248719,9.7081533,-14.7866736\PG=C01 [X(C30H24S2)]\\@

trans-**2a**

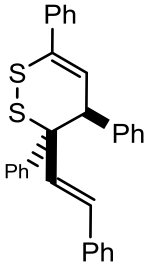

HF=-1952.8604356 a.u. (0)

1\1\GINC-R10N04\FOpt\RPBE1PBE\def2TZVP\C30H24S2\WURTHWE\28-Mar-2019\0\\#
pbe1pbe/def2tzvp emp=gd3bj Opt=readfc geom=check guess=read nosym Pop=NBO Freq
scrf=(solvent=dichloromethane) int=(Acc2e=11,grid=ultrafine)\\Thiochalcone-Dimer\\0,1\C,-
1.3293539287,4.473190343,2.0434466179\C,-0.5128261377,3.8465638885,1.0995869479\
C,0.5223231583,4.5886190636,0.522048778\C,0.7146502027,5.9158629492,0.8624713395\C,-
0.1164955623,6.5313512579,1.7916075674\C,-1.1381254298,5.8033429893,2.3836300496\C,-
0.7642339116,2.4453777868,0.7688200077\C,-0.2556111235,1.7952275521,-0.2762761132\C,-
0.4353751472,0.3343672459,-0.5734821778\C,0.9035641221,-0.4112978493,-0.3273898879\
C,0.9817200216,-1.800131754,-0.8830552417\C,0.2335266107,-2.4136289941,-1.8050268592\S,-
1.131221543,-1.6818691738,-2.6438981078\S,-0.7473599978,0.3014691151,-2.4008403088\
C,0.4542409363,-3.8244925342,-2.1794921996\C,1.7474869387,-4.304002005,-2.387576668\
C,1.9607542515,-5.6307867188,-2.7259931576\C,0.8858718397,-6.4971610786,-2.8717145419\C,-
0.4049770907,-6.0267407509,-2.6765413423\C,-0.6206352849,-4.7008036371,-2.3346707818\
C,2.1270168401,0.3917700943,-0.7221714781\C,2.7503484974,1.2069040324,0.2200205785\
C,3.8476232423,1.9808515749,-0.1235706379\C,4.3449340464,1.9438073432,-1.4195627253\
C,3.743166851,1.1211237996,-2.3604714278\C,2.6443455118,0.3470889829,-2.0126812048\C,-
1.6124770239,-0.2811098479,0.1575362641\C,-2.8900035192,0.2318524823,-0.0727120559\C,-
3.9955432975,-0.2811336652,0.5802215425\C,-3.8497121613,-1.330446933,1.4799734363\C,-
2.5891102741,-1.851311419,1.7142231633\C,-1.4792216954,-1.3298470038,1.0590100276\

H,1.7810544851,-2.3957815474,-0.4496939763\H,0.9675212932,-0.5080448439,0.7630646007\
H,2.587067331,-3.6243505007,-2.2982280899\H,2.9718099129,-5.9868084221,-2.8873633497\
H,1.053548761,-7.53356982,-3.140362973\H,-1.2503567141,-6.6963273948,-2.7849440723\H,-
1.6323449438,-4.3479447135,-2.1659920712\H,2.3604851274,1.2438247171,1.2317155794\
H,4.3157807779,2.6129341725,0.6223936697\H,5.2035217546,2.5469740576,-1.6915215985
\H,4.132160368,1.0768678437,-3.3714209688\H,2.1824579473,-0.2985274863,-2.7491240199\H,-
3.0121881141,1.0429261744,-0.7808000445\H,-4.9764727488,0.1361325569,0.3836074694\H,-
4.7147298797,-1.7388002912,1.9895829927\H,-2.4588132131,-2.6727895204,2.4092824064\H,-
0.5116378167,-1.7699585857,1.2582104906\H,0.393062802,2.3194866556,-0.9717597652\H,-
1.4249392997,1.9155938003,1.4490947805\H,1.1953630075,4.1185040656,-0.1857389963\
H,1.5246166591,6.4741375699,0.4068904814\H,0.0390412707,7.5704016423,2.0576549337\H,-
1.7871908506,6.2713957064,3.1148438747\H,-2.1280278594,3.9050550275, 2.5089872434\\
Version=ES64L-G16RevB.01\HF=-1952.8604356\RMSD=8.742e-09\RMSF=3.592e-
07\Dipole=0.5073052,-0.0730609,0.9743853\Quadrupole=2.3616351,8.1786801,-
10.5403152,2.3043715,-7.4333223,3.1021368\PG=C01 [X(C30H24S2)]\\@

trans-**2a-TS**

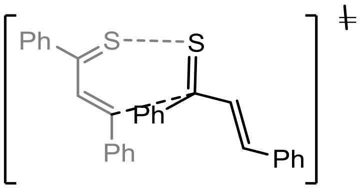

HF=-1952.8272419 a.u. (1, -168.8208cm^{-1})

1\1\GINC-R08N21\FTS\RPBE1PBE\def2TZVP\C30H24S2\WURTHWE\30-Mar-2019\0\\#
pbe1pbe/def2tzvp emp=gd3bj Opt=(ts,noeigentest,readfc) geom=check guess=read
scrf=(solvent=dichloromethane) freq pop=nbo nosym\\Thiochalcone-Dimer-TS\\0,1\C,-
0.0024812827,-0.0835802031,-0.0030279592\C,-0.0101171853,-0.0450246541,1.3994732721\
C,1.1713260214,-0.0731706741,2.1167814038\C,2.3948452266,-0.1399961911,1.4597944782\
C,2.4192296789,-0.1809160338,0.0747447759\C,1.2365377563,-0.1531841834,-0.645807783\C,-
1.2433465682,0.0617998968,-0.7696487848\C,-2.5292659836,-0.4268968923,-0.315630764\C,-
2.7537812675,-1.3013739092,0.6814940529\C,-4.056275007,-1.7539385149,1.140816612\C,-
5.265831023,-1.2063710722,0.6948702418\C,-6.4752409078,-1.6810539321,1.1670985546\C,-
6.5128920762,-2.7095995791,2.1027627776\C,-5.3238807706,-3.2568910885,2.5625982494\C,-
4.1119071685,-2.782245658,2.0880838117\S,-1.1751482274,0.1455996351,-2.4881367457\C,-
1.6298546483,2.363882563,-0.3953866342\C,-2.4142028824,2.4245319474,0.8204116535\C,-
1.8449365151,2.5670878561,2.0922193157\C,-2.638292942,2.5676154644,3.2262503566\C,-
4.0165889812,2.4279586214,3.1212449298\C,-4.598535257,2.2966366484,1.8672984506\C,-
3.8062483165,2.2949746262,0.7340230695\C,-0.3020552806,2.7714689592,-0.4887155549\
C,0.4519865281,2.6961709419,-1.6435185938\C,1.8474549436,3.1559673906,-1.685720634\
C,2.2512375394,4.2185314398,-0.8716744963\C,3.5675402064,4.6495005917,-0.8695202623\
C,4.5065678385,4.0361943591,-1.687243797\C,4.1136322708,2.9894906651,-2.5113017415\
C,2.7991112395,2.5563361244,-2.5138713119\S,-0.2244152364,2.0105362433,-3.0558787635\
H,2.5001253016,1.7322445249,-3.1503317794\H,4.8383668869,2.5024659647,-3.1536836958\
H,5.5356291654,4.3759260401,-1.6879277986\H,3.8582767948,5.4776359894,-0.2333550659\
H,1.5211011815,4.7253345946,-0.2515525911\H,3.3645783761,-0.2261710409,-0.4535771716\
H,1.2655799384,-0.1926569308,-1.7276157799\H,3.3191393792,-0.1575829716,2.0253880452\H,-
0.9481926695,0.0440333043,1.9278482532\H,0.2258781152,3.0504422598,0.4167082937\H,-
0.7721095786,2.6673955776,2.1987280301\H,-2.1961966737,2.3164795819,-1.3199632696\H,-
2.1776247857,2.675195052,4.2015615729\H,-4.6334701884,2.4246946131,4.0121722498\H,-
5.6730322856,2.1903612541,1.7743880829\H,-4.2636293288,2.1981676254,-0.2448257418\H,-
5.2606759064,-0.3926516419,-0.0198605895\H,-7.399422268,-1.2416859899,0.8089524035\H,-

7.4633078263,-3.0758348878,2.4729531068\H,-5.3396405303,-4.0558715004,3.2950773833\H,-3.1840632876,-3.2126204464,2.4504074661\H,-1.9066331968,-1.7444103892,1.1942779872\H,-3.3741399487,-0.053707098,-0.8848997979\H,1.1377467162,-0.0324356421,3.1996866405\\Version=ES64L-G16RevB.01\HF=-1952.8272419\RMSD=5.180e-09\RMSF=1.441e-06\Dipole=0.0829974,0.2584596,1.4617928\Quadrupole=13.2055797,-9.1448808,-4.0606989,3.3398483,-5.0892844,2.1042246\PG=C01 [X(C30H24S2)]\\@

cis-**2a**

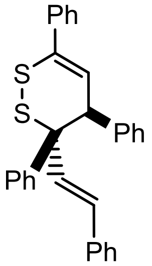

HF=-1952.8578525 a.u. (0)

1\1\GINC-R08N23\FOpt\RPBE1PBE\def2TZVP\C30H24S2\WURTHWE\28-Mar-2019\0\\# pbe1pbe/def2tzvp emp=gd3bj Opt=readfc geom=check guess=read nosym Pop=NBO Freq scrf=(solvent=dichloromethane) int=(Acc2e=11,grid=ultrafine)\\Thiochalcone-Dimer\\0,1\S,-0.9592592851,0.94614264,1.882121179\C,-1.5132654221,-0.5682857457,1.1764215366\C,-0.7189173765,-1.5107152494,0.6571043652\C,0.774831624,-1.5771470904,0.5494918679\C,1.4981920348,-0.2065420902,0.6849691297\S,0.9892244329,0.5350802312,2.3087594132\C,-2.9852693048,-0.6863428825,1.1526362164\C,-3.7871146916,0.4019544334,0.8062083984\C,-5.1675627476,0.2791122265,0.7771298025\C,-5.7683028436,-0.9305087203,1.0958347049\C,-4.9786540575,-2.0165516357,1.4481673354\C,-3.598625801,-1.8951708645,1.481487013\C,0.8839116862,-2.8229730271,2.7526725664\C,1.4412224829,-3.7909813414,3.5765207862\C,2.4483688003,-4.6177548667,3.1006783003\C,2.8884230717,-4.4772632756,1.7914179488\C,2.3317243882,-3.5077274941,0.9727285654\C,1.3340915151,-2.6594894974,1.4460516691\C,3.0010681258,-0.4152810719,0.7167982189\C,3.6811065588,-0.6257841946,-0.4813725024\C,5.0367189256,-0.9110422224,-0.4887820949\C,5.7420764509,-0.9865975634,0.7043912483\C,5.0759530301,-0.779002608,1.9015281651\C,3.7162294013,-0.5016969757,1.9060336749\C,1.0406696115,0.6956744795,-0.4256900685\C,1.6695302453,1.8039818676,-0.8107656893\C,1.2319951063,2.7257186821,-1.8545971667\C,2.1085075488,3.727059834,-2.2784718909\C,1.7455663512,4.6230969377,-3.2722623328\C,0.4921237117,4.5379334497,-3.8594038608\C,-0.3953381713,3.5523704126,-3.4405466587\C,-0.0323994213,2.6588537398,-2.4492748299\H,-1.2245521526,-2.377564393,0.2404499567\H,0.9883290622,-1.8907538709,-0.4813478757\H,-3.3270292392,1.3469107617,0.5378056145\H,-5.7755839967,1.1318379393,0.4980222621\H,-6.8476011046,-1.0255506826,1.073877577\H,-5.439803903,-2.9621563924,1.7092468298\H,-2.9859155955,-2.73795728,1.7799043205\H,0.0909731385,-2.1873296878,3.1263177822\H,1.0818713012,-3.9019467203,4.5933534813\H,2.8825329871,-5.37477148,3.7437140559\H,3.6686031645,-5.1240203057,1.4064258961\H,2.6860697402,-3.3956131113,-0.0461184851\H,3.1427837346,-0.56365751,-1.4192721687\H,5.5441953182,-1.0729341966,-1.4328785884\H,6.803373406,-1.2061094225,0.6996820104\H,5.6113338324,-0.8393890029,2.8418910792\H,3.213949419,-0.3752853572,2.8585778615\H,0.1122689674,0.3978878724,-0.9026761091\H,2.6050159932,2.0675946811,-0.3224412766\H,3.0892310647,3.7967734454,-1.8194679089\H,2.4432464789,5.390883266,-3.58656913\H,0.2025909738,5.2382677164,-4.6341698539\H,-1.3810053312,3.4865329901,-3.8870892573\H,-0.74438424,1.9091742243,-2.1240120843\\Version=ES64L-G16RevB.01\HF=-1952.8578525\RMSD=4.494e-09\RMSF=1.922e-06\Dipole=0.158887,-0.6638859,-0.8542851\Quadrupole=10.5572331,-3.2379809,-7.3192523,-2.0712917,0.758792,-7.7829851\PG=C01 [X(C30H24S2)]\\@

cis-**2a-TS**

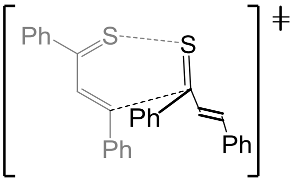

HF=-1952.8277716 a.u. (1, -85.8325 cm^{-1})

1\1\GINC-R02N02\FTS\RPBE1PBE\def2TZVP\C30H24S2\WURTHWE\21-Dec-2018\0\\# pbe1pbe/def2tzvp emp=gd3bj Opt=(ts,noeigentest,calcfc) nosym freq pop=nbo scrf=(solvent=dichloromethane)\\Thiochalcone-Dimer TS\\0,1\C,-0.5515144579,-0.5165744562,-0.0586421193\C,-0.4568603387,-0.0177467586,1.2454776274\C,0.8044341256,-0.0037936225,1.8571905194\C,1.919200973,-0.4703187115,1.1874013873\C,1.8068546737,-0.9639994782,-0.1086201994\C,0.5661972635,-0.9848639722,-0.7291409191\C,-1.6654119929,0.4470737268,1.8981526778\C,-1.7565671593,0.9372048754,3.1487026469\C,-2.9846774126,1.3016991692,3.8122904087\C,-2.7540383699,-1.3610263733,5.066039431\C,-4.0337382217,-1.475906502,5.5026390831\C,-5.1664208117,-1.5062266034,4.6394847633\S,-4.9675393291,-1.3351539811,2.9869847658\S,-4.4720653624,1.1158002728,3.0858572508\C,-6.5128348929,-1.5453130808,5.2307960159\C,-7.5842742683,-2.0984257238,4.5225771964\C,-8.8543288304,-2.1348989919,5.0683245538\C,-9.0879925749,-1.6069942975,6.3321585978\C,-8.037660178,-1.046195779,7.0446174136\C,-6.7625418953,-1.0215615246,6.5044668829\C,-1.5842215454,-1.1673965687,5.8868048244\C,-1.6560053481,-0.7474523829,7.2210784506\C,-0.5034962481,-0.5164095519,7.9477831025\C,0.7441549321,-0.69490917,7.3606581747\C,0.8327185138,-1.1027686644,6.035793059\C,-0.3200361617,-1.3301710673,5.3056088298\C,-2.901887637,1.9032296286,5.1579518468\C,-3.9902717197,1.863062586,6.0368299809\C,-3.9073455906,2.4126802511,7.3009514156\C,-2.7371869782,3.0377970514,7.720086944\C,-1.6554472214,3.1007793198,6.8572095845\C,-1.7329481057,2.5351037722,5.5931976099\H,-4.2244406729,-1.5098007199,6.5695102995\H,-2.5858025509,-1.4326241579,3.9965585372\H,-7.401180037,-2.5098897598,3.5372998711\H,-9.6674540027,-2.5798498676,4.5066212502\H,-10.0842514319,-1.6312625399,6.7580082791\H,-8.2121351854,-0.6184099651,8.025031663\H,-5.9631843218,-0.5569655631,7.0685731475\H,-2.6208545863,-0.5690124484,7.6794129624\H,-0.5747190195,-0.1802690287,8.9755855655\H,1.6450705319,-0.5076217087,7.9332483567\H,1.8023960747,-1.2383412619,5.5709256025\H,-0.2550871847,-1.6426423289,4.2688512034\H,-4.9027502703,1.3804133465,5.709671987\H,-4.7609719662,2.3543015087,7.9668591508\H,-2.6735286748,3.4722763659,8.7108798051\H,-0.7400997892,3.5923963451,7.1657308226\H,-0.8772748595,2.6142346217,4.9349892765\H,-0.8574624288,1.0082927106,3.7486883742\H,-2.5793382161,0.3759544955,1.3139467776\H,0.9158805847,0.3756747895,2.8659767989\H,2.8861393949,-0.4510157323,1.6769142383\H,2.6840215718,-1.3299528955,-0.629248612\H,0.4685983936,-1.3676740326,-1.7383783314\H,-1.5216901832,-0.5351775643,-0.5435878324\\Version=ES64L-G16RevB.01\HF=-1952.8277716\RMSD=4.858e-09\RMSF=9.793e-07\Dipole=1.5162386,-0.1358754,1.5465808\Quadrupole=-7.8483555,-21.6746129,29.5229685,4.0507565,-4.3911379,7.4504607\PG=C01 [X(C30H24S2)]\\@

trans-**3a**

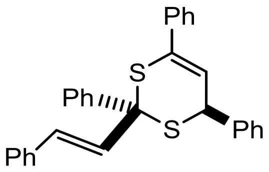

HF=-1952.8551268 a.u. (0)

1\1\GINC-R08N01\FOpt\RPBE1PBE\def2TZVP\C30H24S2\WURTHWE\28-Mar-2019\0\\# pbe1pbe/def2tzvp emp=gd3bj Opt=readfc geom=check guess=read nosym Pop=NBO Freq scrf=(solvent=dichloromethane) int=(Acc2e=11,grid=ultrafine)\\ Thiochalcone-Dimer\\0,1\ C,2.7655168598,1.7863783863,2.880047546\C,2.9301744513,1.6806268797,1.5034431549\C,3.6531 890353,2.6611254392,0.8297029531\C,4.2070667071,3.7260981512,1.5230850038\C,4.0423259212, 3.8231483555,2.8985240473\C,3.3199290965,2.8509253748,3.575769182\C,2.3083291239,0.531634 4721,0.75311422\S,0.7714894982,1.1713662045,0.0259275978\C,0.2017410539,-0.3665632043,- 0.7899278636\S,1.3440222702,-0.761086483,-2.1582632994\C,2.9407957437,-0.5944805815,- 1.4492435027\C,3.2306314149,-0.0670991689,-0.2551459211\C,-1.0677941618,-0.0000689182,- 1.5034031623\C,-2.1661838224,-0.7547225328,-1.5286870425\C,-3.407499643,-0.4605067636,- 2.2393546441\C,4.0060889387,-1.096530099,-2.3429227899\C,3.8594557592,-2.2930229107,- 3.0455860602\C,4.8775064326,-2.7606757878,-3.862299566\C,6.0541706608,-2.0378829285,- 3.9958691176\C,6.2053661671,-0.840218492,-3.3096543496\C,5.1883952667,-0.3703527629,- 2.4950365866\H,2.0110181251,-0.2315996899,1.4815011492\C,0.049510006,-1.476008985, 0.2342498873\H,4.2753780243,-0.0858294374,0.0432331313\H,2.9470069198,-2.86781875,- 2.9381350812\H,4.7511368319,-3.6971945483,-4.3930993809\H,6.8482061994,-2.4025274309,- 4.6370442159\H,7.1156440508,-0.2619932591,-3.4190531114\H,5.2981790335,0.5770937699,- 1.9803742223\H,-2.1650507161,-1.6874966239,-0.971853257\H,3.7790941035,2.5857708976,- 0.2451360347\H,4.7694106219,4.4829893708,0.988416799\H,4.4774170722,4.6544235765,3.44112 46894\H,3.188874101,2.9195984366,4.6495447758\H,2.2003333513,1.0258739397,3.4088122311\C, -0.7765164627,-1.2507924898,1.3374042716\C,-0.9512612971,-2.2265000143,2.3021860274\C,- 0.2940368151,-3.4464571189,2.1899840114\C,0.5311350403,-3.6764432911,1.1027666015\ C,0.6994804006,-2.6977745276,0.1297089826\H,-1.2808218287,-0.2970787407,1.4364940889\H,- 1.6007190927,-2.0332036543,3.1482016885\H,-0.4254230909,-4.2091604438,2.9487084071\ H,1.052388537,-4.621539302,1.0034888836\H,1.3424120353,-2.8901033834,-0.7192939622\H,- 1.0127581033,0.9329327739,-2.0565198787\C,-4.448503808,-1.3889430473,-2.1701002554\C,- 5.6505140279,-1.1671936104,-2.8241742832\C,-5.8347779936,-0.0070303124,-3.560886352\C,- 4.8077606531,0.9276665756,-3.6373002135\C,-3.6086082975,0.7051492466,-2.985459164\H,- 4.306256422,-2.2970657256,-1.5936321649\H,-6.444308106,-1.9022096244,-2.7572288425\H,- 6.7727258241,0.1716193285,-4.0735979627\H,-4.9454339796,1.8373220051,-4.210548095\H,- 2.8225857095,1.4475494594,-3.0572179471\\Version=ES64L-G16RevB.01\HF=-1952.8551268 \RMSD=3.619e-09\RMSF=5.382e-07\Dipole=0.161366,-0.5167063,0.4731387 \Quadrupole=6.3394368,-1.4312295,-4.9082073,3.5975176,-0.036698,-0.3013789\PG=C01 [X(C30H24S2)]\\@

trans-**3a-TS**

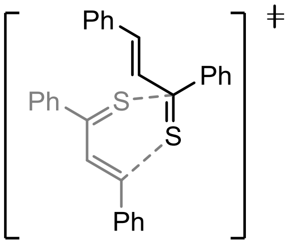

HF=-1952.830899 a.u. (1, -86.5812cm^{-1})

1\1\GINC-R10N38\FTS\RPBE1PBE\def2TZVP\C30H24S2\WURTHWE\03-Jan-2019\0\\#
pbe1pbe/def2tzvp emp=gd3bj Opt=(ts,noeigentest,readfc,tight) geom=check guess=read freq pop=nbo
nosym scrf=(solvent=dichloromethane)\\Thiochalcone-Dimer: TS\\0,1\ C, -0.1601028584, -
0.0335617084,0.0584548147\C,-0.0644502587,-0.1952216407,1.4411104442\C,1.2053781599,-
0.3645488351,1.9987035645\C,2.3389486413,-0.3475799403,1.2035326948\C,2.2289920512,-
0.1654495235,-0.1678469849\C,0.9723952723,-0.0141201679,-0.7372744478\C,-1.2757240246,-
0.1673237931,2.2918593642\S,-2.8130592308,-0.4266482362,1.6502093594\C,-
1.164295271,0.23705442,3.6152036546\C,-1.2688049101,2.6501237698,3.043514637\C,-
2.3987251142,2.5630213126,2.2665648902\C,-3.5927493632,2.027505216,2.7525780526\S,-
3.6714302866,1.460175052,4.3779432673\C,-2.2961932331,0.1345760479,4.5343843202\
C,0.050077658,3.0394715554,2.5881029041\C,0.3927075139,3.1073552843,1.2333365064\C,1.6737
804288,3.4542145769,0.8483960154\C,2.64029215,3.7406225689,1.8050879904\C,2.315554829,3.6
809387235,3.1537762126\C,1.0333367182,3.3328168317,3.5393767759\C,-4.8610124235,
2.1768690707,2.0222090223\C,-5.9067309319,1.2661017592,2.1849463886\C,-7.0915907386,
1.4247170923,1.4900050489\C,-7.2586949216,2.5015508957,0.6269342448\C,-6.2307356299,
3.4191836514,0.4670091246\C,-5.041457206,3.260556461,1.1605795532\C,-1.9667552248,
0.0881791641,5.9940710399\C,-1.0132835968,0.9282818872,6.5635238401\C,-0.7413589312,
0.8696409874,7.9207124984\C,-1.4238365344,-0.0284137016,8.7308864811\C,-2.3780627366,-
0.8667299583,8.1741052897\C,-2.6468882438,-0.80686135,6.8142404228\H,-2.8931359738,-
0.7320394572,4.2340391924\H,-0.1902451606,0.4524838175,4.0345150748\H,1.3136400361,-
0.5295599821,3.0636049056\H,3.3137859259,-0.481644759,1.6581126719\H,3.1170022536,-
0.1491833094,-0.789142525\H,0.8737920872,0.1247843235,-1.8079207737\H,-1.1432138331,
0.0883776158,-0.3797394896\H,-1.4201086983,2.6361356239,4.1185359926\H,-3.3919690829,-
1.4648810737,6.379759842\H,-2.9141710214,-1.5723202921,8.7981020455\H,-1.210374019,-
0.0743397003,9.7923923908\H,0.0060511568,1.5280225473,8.3480557789\H,-0.4728425759,
1.6355124586,5.9456057611\H,-5.7660280893,0.4204389672,2.8468626324\H,-7.8892266891
,0.702046124,1.6170761168\H,-8.1892098485,2.6261877007,0.085491582\H,-6.3573278582,
4.2693715009,-0.1931077812\H,-4.2538805176,3.9967897157,1.0519671158\H,-2.3518060788,
2.7868429799,1.2069522483\H,0.7800510982,3.2887876068,4.5933845301\H,3.0640723853,3.9041
776132,3.9048928459\H,3.6454783726,4.0060787487,1.4990592691\H,1.9258327588,3.4907787841
,-0.2047351605\H,-0.3418023809,2.8651059726,0.4764057438\\Version=ES64L-G16RevB.01\HF=-
1952.830899\RMSD=6.556e-09\RMSF=1.303e-06\Dipole=1.4260242,1.2265798,
0.4035573\Quadrupole=-0.6168219,-8.7220479,9.3388698,3.2156456,8.9957114,-2.902726\PG=C01
[X(C30H24S2)]\\@

cis-**3a**

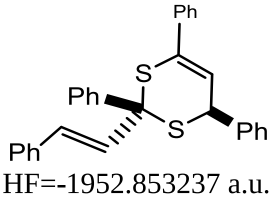

HF=-1952.853237 a.u. (0)

1\1\GINC-R10N01\FOpt\RPBE1PBE\def2TZVP\C30H24S2\WURTHWE\28-Mar-2019\0\\#
pbe1pbe/def2tzvp emp=gd3bj Opt=readfc geom=check guess=read nosym Pop=NBO Freq
scrf=(solvent=dichloromethane) int=(Acc2e=11,grid=ultrafine)\\Thiochalcone-Dimer\\0,1\C,-
0.3419921496,-1.905486447,1.3554179344\C,0.1379513637,-1.2535507491,0.2990155832\
H,1.1727213545,-1.3783400513,-0.0047789875\C,-0.6499911517,-0.337842001,-0.5930071223\S,-
2.1511710929,0.3407089542,0.1857794987\C,-1.4455995889,1.1417960194,1.6567371598\C,-
0.2155085475,1.947487609,1.4089658621\C,0.5931876325,1.9909925722,0.3458584553\S,0.444549
9326,1.0371879215,-1.1180873306\C,-2.5626527326,1.9729474622,2.2413127653\C,-
3.2948672218,1.4969208342,3.3226015688\C,-4.3323296367,2.2489175291,3.8563052133\C,-
4.6463752965,3.4848147054,3.3103828973\C,-3.9197216323,3.9653182647,2.2280543775\C,-
2.8852708481,3.2126945561,1.6959307281\C,-1.1163176574,-1.004575508,-1.8789531034\
C,1.7316361341,2.9337828641,0.2968740886\C,1.5674349627,4.2469027306,0.7403664037\C,2.630
552079,5.1350344034,0.7255883389\C,3.8729202047,4.729124694,0.2570833432\C,4.0430806278,3
.429376858,-0.1984286102\C,2.9805150388,2.5392025398,-0.1838600968\H,0.591963384,
4.5718361977,1.0834674484\H,2.4850363793,6.1523743001,1.0702416051\H,4.7032486642,5.4254
921975,0.2409390116\H,5.0099957622,3.1038915111,-0.564513426\H,3.1193601248,
1.5226773646,-0.5324779107\H,0.0584348241,2.5824693446,2.2478706917\H,-1.2046811929,
0.3548920943,2.3780982503\H,-3.047360489,0.531101259,3.7508471614\H,-4.8940438494,
1.8685090886,4.7016731661\H,-5.4546848692,4.0742590816,3.7274341791\H,-4.160774303,
4.9304909805,1.7975433425\H,-2.3179258222,3.5843025598,0.8493182439\C,-0.9677088963,-
2.3728965798,-2.0740914873\C,-1.4175323191,-2.9683871165,-3.2448696684\C,-2.0252368564,-
2.2046752028,-4.2288642879\C,-2.1822751875,-0.8379667565,-4.0375542243\C,-1.7295118582,-
0.241396941,-2.8727554891\H,-0.5038335721,-2.9776782889,-1.3052760818\H,-1.2917755849,-
4.0359288302,-3.3835608115\H,-2.3769162829,-2.6708063234,-5.141925931\H,-2.6590317893,-
0.2323734009,-4.7994935486\H,-1.8517621642,0.8253693911,-2.7265529192\C,0.3952252047,-
2.820903158,2.2226647193\C,-0.3060475145,-3.503667555,3.2188032338\C,0.3375046826,-
4.3894450504,4.0693167193\C,1.7011707451,-4.6060844161,3.9418852069\C,2.4140129176,-
3.9282468373,2.958797458\C,1.7707671668,-3.0461116001,2.1096972952\H,-1.3729950697,-
3.3345288229,3.3209317553\H,-0.2278500658,-4.9096815909,4.8339346454\H,2.2095525441,-
5.2957468909,4.6055193612\H,3.4813717106,-4.0880592262,2.8570957776\H,2.3468284505,-
2.5215163053,1.3565568939\H,-1.3916856486,-1.7707952389,1.6061886526\\Version=ES64L-
G16RevB.01\HF=-1952.853237\RMSD=8.286e-09\RMSF=1.037e-06\Dipole=0.3420334,-
0.2153351,0.6831343\Quadrupole=-2.9235176,4.4461594,-1.5226418,-1.929519,-
2.767171,0.2734489\PG=C01 [X(C30H24S2)]\\@

cis-**3a-TS**

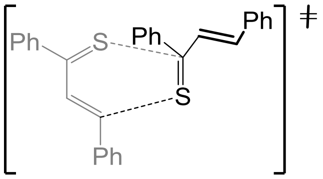

HF=-1952.81538 a.u. (1, -229.9646 cm^{-1})

1\1\GINC-R08N02\FTS\RPBE1PBE\def2TZVP\C30H24S2\WURTHWE\27-Mar-2019\0\\#
pbe1pbe/def2tzvp emp=gd3bj Opt=(ts,noeigentest,readfc) geom=check guess=read freq pop=nbo
scrf=(solvent=dichloromethane) nosym int=(Acc2e=11,grid=ultrafine)\\Thiochalcone-dimer:
TS\\0,1\C,-0.1267242711,-0.1571422326,-0.0200797738\C,-0.1154829352,-0.1179370735,
1.3747954003\C,1.11139351,-0.0651518352,2.0353306696\C,2.3002443692,-0.0685706307,
1.3226421526\C,2.2775493706,-0.114885974,-0.0636441824\C,1.0598851107,-0.1569795859,-
0.7323343038\C,-1.3519218914,-0.1166510903,2.1706799655\C,-2.513179506,-0.7605645265,
1.7270691294\C,-3.6357613013,-0.9411403847,2.5409756209\S,-3.7245103514,-0.2069213888,

4.0496243029\C,-4.7732459467,-1.7172066479,1.9989943212\C,-4.5619611643,-2.7325502766,
1.0625766809\C,-5.6256682028,-3.4544932017,0.5459426046\C,-6.9228990341,-3.1702109149,
0.9493916349\C,-7.1448178794,-2.168002741,1.8845330201\C,-6.0802801409,-1.4559186298,
2.4089882732\C,-3.2805408715,2.3404220983,2.7381437155\C,-4.2708367625,
1.9933077631,1.7151578317\C,-5.6513427535,2.0388561128,1.9706833729\C,-6.5632676669,
1.6916184505,0.997923742\C,-6.1297732744,1.2745899006,-0.2584748501\C,-4.7746175219,
1.2142861433,-0.528013811\C,-3.8553212649,1.5732468113,0.4467200305\S,-1.6429268979,
2.1025800386,2.4165304547\C,-3.6957034871,3.1137728753,3.8899376309\C,-2.8839592936,
3.5330996807,4.876133398\C,-3.2654843625,4.3204890195,6.0314804784\C,-4.5719750437,
4.7716530541,6.2630956704\C,-4.8705824213,5.5190743718,7.3859054074\C,-3.876082839,
5.8348532039,8.3064650637\C,-2.5775520714,5.3958386662,8.0929050995\C,-2.2773148531,
4.6474824655,6.9671430112\H,-4.7430546807,3.3839646795,3.9260135825\H,-6.245548852,-
0.673835207,3.1388901082\H,-8.1545785482,-1.9387161122,2.2055529793\H,-7.7558881037,-
3.7317935792,0.5423269092\H,-5.4393577556,-4.2466699468,-0.1700919481\H,-3.5533993076,-
2.9812152032,0.7544461865\H,-2.5574583155,-1.1078804376,0.7027526239\H,-1.2176491393,-
0.1344337501,3.2509595591\H,1.1277842132,-0.0252982049,3.1192370303\H,3.2457720582,-
0.0337686337,1.8510895565\H,3.2052963272,-0.115366508,-0.6238019215\H,1.0368358837,-
0.1869380802,-1.8154816141\H,-1.069106653,-0.1790709738,-0.5549946195\H,-
6.018683121,2.3076907496,2.951878552\H,-7.6229769841,1.7251537825,1.2214215259\H,-
6.8515687399,0.9956609114,-1.017154593\H,-4.4245391843,0.8929219226,-1.5018697884\H,-
2.7957723112,1.578139057,0.2240040127\H,-1.2621822736,4.3037534685,6.7997990997\H,-
1.7969176504,5.6368851756,8.8049252075\H,-4.1161999005,6.4208248674,9.1859032128\H,-
5.8863982155,5.859912702,7.5492188323\H,-5.3617693345,4.5368233148,5.5596948977\H,-
1.8351621785,3.2540177801,4.8210715782\\Version=ES64L-G16RevB.01\HF=-1952.8153841\
RMSD=8.863e-09\RMSF=1.278e-06\Dipole=-0.3629149,0.5752962,-1.0673165\Quadrupole
=15.5966413,-9.6203484,-5.9762929,-8.2266132,6.1206318,20.3432039\PG=C01
[X(C30H24S2)]\\@

2. s-*trans*-*E*-1,3-diphenyl-prop-2-ene-1-thione (1a) - Nitrosobenzene (15) Cycloadditions and Thiobenzophenone/ α-Phenylnitrosoethene-Cycloadditions

Table S2: Total energies (E_{tot}), energies at 0K E_0), and Gibbs free energies (G_{298})[a.u.], relative energies of the nitrosobenzene (**15**)-thiochalcone (**1a**) cycloadducts and transition state energies also with respect to the respective heterocyclic ring (last column)[kcal/mol]

Species	E_{tot} [a.u.]	E_{rel} [kcal/mol]	E_0 [a.u.]	E_{rel} [kcal/mol]	G_{298} [a.u.]	To **1a+15** E_{rel} [kcal/mol]	To Ring E_{rel} [kcal/mol]
**1a**	-976,40820	-	-976,18464	-	-976,22691	-	
**15**	-361,27241		-361,17457		-361,20502		
**16a**	-1.337,71150	- 19,38	-1.337,38590	-16,74	-1.337,43677	-3,03	
**16a-TS**	-1337,68017	0,28	-1.337,35744	1,12	-1.337,40777	15,17	18,19
**17a**	-1.337,70423	-14,82	-1.337,37856	-12,14	-1.337,42833	2,27	
**17a-TS**	-1.337,67943	0,74	-1.337,35615	1,92	-1.337,40652	15,95	13,68

Gaussian Archive Entries (Total energies (a.u.), number of imaginary frequencies (for transition states: imaginary frequencies), coordinates)

Nitrosobenzene 15

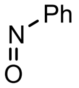

HF=- 361.2724082 a.u. (0)

1\1\GINC-R01N02\FOpt\RPBE1PBE\def2TZVP\C6H5N1O1\WURTHWE\28-Feb-2019\0\\# pbe1pbe/def2tzvp Opt=readfc emp=gd3bj geom=check guess=read scrf=(solvent=dichloromethane) Pop=NBO Freq\\Nitrosobenzene\\0,1\O,-0.3383023395,0.6918294525,0.5076127478\N, 0.1678346906,-0.2952576627,0.9827787525\C,1.4602457702,-0.1177298195,1.5523070354\C, 2.1304799672,1.104940673,1.577817134\C,3.3815075553,1.1668594481,2.1553493718\C,3.9567022136,0.0193976404,2.7015545061\C,3.2847297236,-1.1946554258,2.6731616355\C,2.0281497918,-1.2649468141,2.094989743\H,1.6577639377,1.9780788057,1.145604233\H,3.9203792226,2.1062792013,2.1862628202\H,4.9402476744,0.0782591592,3.1531774414\H,3.7400398608,-2.0797599331, 3.099985024\H,1.470228095,-2.193294725,2.0523995553\\Version=ES64L-G16RevB.01\State=1-A\HF=-361.2724082\RMSD=7.629e-09\RMSF=1.083e-05\Dipole=1.7159019,-0.094848, 0.8350229\Quadrupole=-2.4104411,3.9450155,-1.5345744,0.8065986,-0.6508209,-0.9040137\PG=C01 [X(C6H5N1O1)]\\@

16a

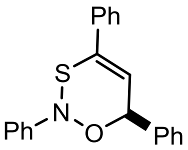

HF=-1337.7114962 a.u. (0)

1\1\GINC-R01N07\FOpt\RPBE1PBE\def2TZVP\C21H17N1O1S1\WURTHWE\28-Feb-2019\0\\#
pbe1pbe/def2tzvp emp=gd3bj Opt=readfc geom=check guess=read nosym Pop=NBO Freq
scrf=(solvent=dichloromethane)\\Nitrosobenzene+Thiochalcone\\0,1\C,-0.3868012427,-
0.0443462415,0.11310456\C,-0.2286707245,-0.1013524542,1.4983844054\C,1.0569119609,-
0.2428338931,2.0239094464\C,2.1525219465,-0.3514083181,1.1841606183\C,1.984207521,-
0.3033950195,-0.1936634589\C,0.7122750407,-0.1464797317,-0.7253442843\C,-1.3864051218,-
0.0126548533,2.4026609355\S,-2.6362370474,1.1087046259,1.9061537264\N,-
3.8449128397,0.4649562592,2.9555071423\O,-3.409387871,0.5914303622,4.2853695236\C,-
2.5732201888,-0.5276854312,4.5731023029\C,-1.501821324,-0.7031499334,3.5408166212\C,-
1.9871638165,-0.2648433133,5.9360997118\C,-1.1235737703,0.8100624033,6.1280429477\C,-
0.5847815135,1.0570162318,7.3795628449\C,-0.8956247882,0.2240930433,8.4483126459\C,-
1.7511508265,-0.8506108572,8.260636733\C,-2.2987029266,-1.0908274211,7.0069223888\C,-
5.1428589217,0.9877581853,2.782485022\C,-5.6483001512,1.1438954284,1.4902518617\C,-
6.9479231844,1.5814855664,1.3065024381\C,-7.7612349057,1.8692598444,2.3953084492\C,-
7.2530030732,1.7126677479,3.675097113\C,-5.9507788777,1.2785405886,3.8784889477\
H,1.1923056571,-0.2497398741,3.0992728256\H,-1.380483788,0.060185048,-0.3073949566\
H,0.5726733995,-0.1088157337,-1.799453962\H,2.8431098469,-0.3812452593,-0.8499550527\
H,3.1445456186,-0.4599677423,1.6070765743\H,-3.1931834832,-1.4319237147,4.622124904\H,-
0.7502739887,-1.4502947757,3.7738122262\H,-0.8787279937,1.4527115499,5.2894895722\
H,0.0832730996,1.8983342498,7.5236636208\H,-0.469554059,0.4148581006,9.4265530273\H,-
1.997337964,-1.502271195,9.0909570434\H,-2.9737567093,-1.9271896532,6.8593117123\H,-
5.0258251056,0.9131568318,0.63438045\H,-7.3274495047,1.696314438,0.2976880329\H,-
8.7777316826,2.2121385083,2.2451941479\H,-7.8724633811,1.9350517387,4.5365871894\H,-
5.5598925218,1.1618985583,4.8786266485\\Version=ES64L-G16RevB.01\HF=-1337.7114962\
RMSD=6.045e-09\RMSF=1.697e-06\Dipole=0.7535031,-0.5724515,0.1941024\Quadrupole=-
1.9410225,-9.6817302,11.6227527,2.0344078,3.6797886,-5.9634481\PG=C01
[X(C21H17N1O1S1)]\\@

16a-TS

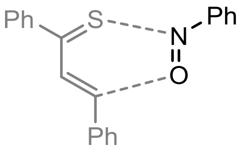

HF=-1337.6801676 a.u. (1, -186.7247cm^{-1})

1\1\GINC-R02N29\FTS\RPBE1PBE\def2TZVP\C21H17N1O1S1\WURTHWE\05-Mar-2019\0\\#
pbe1pbe/def2tzvp emp=gd3bj Opt=(ts,noeigentest,readfc) geom=check guess=read nosym Pop=NBO
Freq scrf=(solvent=dichloromethane)\\ Retro-Diels-Alder: TS\\0,1\C,0.1805172267,0.0030940347,
0.1311589895\C,0.1203315651, 0.0571478262,1.5172030911\C,1.2818801186, 0.1091976188,

2.2800437606\C,2.5107386886,0.1156270711,1.6476375031\C,2.582364413,0.0690324674,0.258871
721\C,1.4187165865,0.0113463345,-0.4943198192\N,-1.1411737348,-0.0482438318,2.2074763065\
O,-2.1252599563,-0.2201950385, 1.478509797\C,-2.8330745157,1.854246447,0.7332595849\C,-
3.5375868548,1.6425939189,-0.5141232757\C,-2.9495760888,1.8663826436,-1.7650964564\C,-
3.664027837,1.6450651464,-2.9274524386\C,-4.9771806605,1.1908968846,-2.867669346\C,-
5.57006563,0.9546410357,-1.6350494124\C,-4.8549638364,1.1744909323,-0.4704071324\C,-
1.6728595585,2.5743291042, 0.8776121629\C,-0.9419087013,2.6473061864,2.0745570634\S,-
1.3865144781,1.6702160244, 3.3777153654\C,0.3391792537,3.3711852365,2.1122871131\
C,0.8155726189,3.899042569,3.3147870063\C,2.0255208199,4.5675660577,3.3669623457\C,2.7951
171647,4.7069149205,2.2187536953\C,2.3407528879,4.1759718135,1.0201863827\C,1.1222569665,
3.5198734047,0.9646594351\H,0.8045862473,3.0773660798,0.0293430399\H,0.2199796723,3.7824
24122,4.2127392995\H,2.3718741497,4.9802043223,4.3073710483\H,3.7476469956,5.2222649141,
2.2604159241\H,2.943687242,4.2622627137,0.1237080533\H,-3.3595414781,1.5732427642,
1.6385163504\H,-1.2510786107,3.0652573725 ,0.0075937138\H,-1.9213862445,2.2024271846,-
1.8285668235\H,-3.1951889335,1.8207473291,-3.8886527593\H,-5.5331600302,1.016522197,-
3.7814253779\H,-6.5911825558,0.5956501078,-1.5822869779\H,-5.3145661435,0.9868529916,
0.4938322935\H,1.2061172061,0.1580186711, 3.359825367\H,3.4185862496,0.1600789278,
2.2373518251\H,3.5477060609,0.0740721923,-0.2334905925\H,1.4736706471,-0.0303427734,-
1.5760948739\H,-0.7405457445,-0.0603366149,-0.4334494353\\Version=ES64L-G16RevB.01\HF=-
1337.6801676\RMSD=5.580e-09\RMSF=2.320e-06\Dipole=0.9614452,0.8911456,-
1.4173973\Quadrupole=7.5892529,-6.8022165,-0.7870365,5.10182,9.2504567,0.3949854\PG=C01
[X(C21H17N1O1S1)]\\@

17a

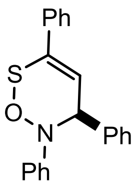

HF=-1337.7042299 a.u. (0)

1\1\GINC-R01N24\FOpt\RPBE1PBE\def2TZVP\C21H17N1O1S1\WURTHWE\28-Feb-2019\0\\#
pbe1pbe/def2tzvp emp=gd3bj Opt=readfc geom=check guess=read nosym Pop=NBO Freq
scrf=(solvent=dichloromethane)\\Nitrosobenzene+Thiochalcone 2.Isomer\\0,1\C,1.8184237715,-
0.7994160381,4.5069088048\N,1.6589769269,-1.9814928082,5.2676406249\O,0.8006000909,-
1.7653254155,6.3752236124\S,-0.7402853547,-1.2447071107,5.9391715395\C,-1.1202094846,-
2.4412438786,4.7290508224\C,-0.185852486,-3.254026508,4.2249369486\C,1.2536795287,-
3.2383156863,4.6424690608\C,2.2671618509,-3.6473766179,3.5919186478\C,1.9118695654,-
4.0444503256,2.3094370576\C,2.8824584846,-4.4389409754,1.3974403642\C,4.2205576565,-
4.4385210547,1.7567160781\C,4.5834405455,-4.047071407,3.0392325257\C,3.613865458,-
3.6604600166,3.9488186477\C,-2.5229518606,-2.4202247865,4.283270945\C,-2.8244768907,-
2.5472690728,2.926491666\C,-4.1407678987,-2.5462954456,2.4964888381\C,-5.174552121,-
2.4008158379,3.4126277296\C,-4.8837010501,-2.2576032546,4.7616592585\C,-3.5671957451,-
2.265495829,5.1959702751\H,-2.0153393742,-2.6302027027,2.2101848173\H,-3.3450536758,-
2.1743828228,6.2532420118\H,-5.6855679756,-2.1455657415,5.4820541379\H,-6.2040505436,-
2.3933173627,3.0741518002\H,-4.360982147,-2.645808716,1.4399192902\H,1.3650807797,-

3.973446602,5.451144242\H,-0.5099257278,-4.0256584102,3.5364735992\H,0.874064023,-4.0379025942,1.9995833054\H,2.5863501827,-4.7434511997,0.400188929\H,4.9778792453,-4.7425455647,1.0435648084\H,5.6268697998,-4.0457564734,3.3328918345\H,3.8982899556,-3.353674302,4.9488624971\C,1.5256594838,-0.7083364844,3.1509368625\C,1.7485118972,0.4826709129,2.4731562988\C,2.2357574236,1.594524801,3.1406665568\C,2.5099773114,1.5084086973,4.5009343191\C,2.3129962431,0.3195539336,5.1778615851\H,1.1180568459,-1.5556690072,2.6198907253\H,1.5223266184,0.5392892213,1.4145849366\H,2.4009720222,2.5233576592,2.6077180324\H,2.8925773989,2.3705417798,5.0348979803\H,2.5378449146,0.2409241239,6.2345120416\\Version=ES64L-G16RevB.01\HF=-1337.7042299\RMSD=5.047e-09\RMSF=1.439e-06\Dipole=-0.4129608,-0.3449513,-1.1435775\Quadrupole=10.4079697,2.5862195,-12.9941892,2.3429328,-3.2159278,4.3551533\PG=C01 [X(C21H17N1O1S1)]\\@

17a-TS

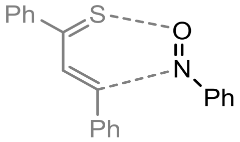

HF=-1337.6794312 a.u. (1, -306.9007cm^{-1})

1\1\GINC-R08N01\FTS\RPBE1PBE\def2TZVP\C21H17N1O1S1\WURTHWE\05-Mar-2019\0\\# pbe1pbe/def2tzvp emp=gd3bj Opt=(ts,noeigentest,readfc) geom=check guess=read nosym Pop=NBO Freq scrf=(solvent=dichloromethane)\\ Retro-Diels-Alder: TS\\0,1\C,0.0574565321,0.0434525566,0.033578899\C,0.0487165509,-0.0033840464,1.4212375608\C,1.2308058375,0.0396443622,2.1521180152\C,2.4378483581,0.1070154351,1.4792765691\C,2.4599900726,0.1372106168,0.0890703501\C,1.2714234174,0.110129726,-0.6291551525\N,-1.1811989163,-0.0472549251,2.1586511645\O,-2.204255974,0.1511451396,1.5229864221\C,-1.4654533105,-1.6636771608,3.2292821147\C,-0.8712923667,-1.4932371406,4.5570148678\C,0.3808877555,-2.0043964328, 4.9032299469\C,0.8985407589,-1.797095694,6.1707151805\C,0.1745167709,-1.0789920715,7.1132763791\C,-1.074309564,-0.5699464011,6.7827287598\C,-1.5906394802,-0.7765291769,5.5153618247\C,-0.9648884251,-2.5514546673,2.2693741128\C,-1.5541133362,-2.7644762096,1.0219308098\C,-0.8127773431,-3.574640166,0.0310109642\C,0.5792778342,-3.5041083979,-0.0563945999\C,1.2642818086,-4.2472302694,-1.0042117809\C,0.572486937,-5.0846385808,-1.8680993696\C,-0.8114732737,-5.1665785488,-1.7857659831\C,-1.4979993872,-4.4116388304,-0.8498895301\S,-2.9770147678,-2.0011014264,0.571674328\H,1.1281803763,-2.8321355479,0.5926839658\H,-2.5783087921,-4.4692965998,-0.7846186998\H,-1.3580267586,-5.8221953606,-2.4537109058\H,1.1096826228,-5.6701553265,-2.6051962358\H,2.3429282012,-4.1652582926,-1.0726562389\H,-2.5280219552,-1.4439889429,3.1670134332\H,0.0111485548,-2.9930304697,2.4307211739\H,0.9621517644,-2.5668368659,4.183301891\H,1.8715915797,-2.2002231869,6.4255634619\H,0.5818802695,-0.9197316338,8.1048191085\H,-1.6461399451,-0.011938763,7.5147105254\H,-2.5645171751,-0.3771016218,5.2536658745\H,1.1922801434,0.022989915,3.2341905743\H,3.3650060401,0.1420357439,2.0387327597\H,3.4071321996,0.1898198138,-0.4347510512\H,1.2913912088,0.1356786095,-1.7122600626\H,-0.8818800678,0.0138236031,-0.5034134928\\Version=ES64L-G16RevB.01\HF=-1337.6794312\RMSD=9.424e-09\RMSF=1.868e-06\Dipole=2.4429295,-0.2228836,0.9783549\Quadrupole=-7.6008624,-7.7975417,15.3984041,-6.95305,3.2700274,2.3518511\PG=C01 [X(C21H17N1O1S1)]\\@

3. Thiobenzophenone (18) - α-Phenylnitrosoethene(10a) Cycloadditions

Table S3: Total energies (E_{tot}), energies at 0K E_0), and Gibbs free energies (G_{298})[a.u.], relative energies of the thiobenzophenone (**18**) - α-phenylnitrosoethene (**10a**) cycloadducts and transition state energies also with respect to the respective heterocyclic ring (last column)[kcal/mol]

Species	E_{tot} [a.u.]	E_{rel} [kcal/mol]	E_0 [a.u.]	E_{rel} [kcal/mol]	G_{298} [a.u.]	To **10a+18** E_{rel} [kcal/mol]	To Ring E_{rel} [kcal/mol]
**Thiobenzo-phenone 18**	-899,06557		-898,87582		-898,91357		
**α-Phenyl-nitroso-ethene 10a**	-438,60583	-	- 438,47480	-	-438,50857		
**19a**	-1.337,73888	-42,35	-1.337,41308	-39,19	-1.337,46350	-25,96	
**19a-TS**	-1.337,67487	-2,18	-1.337,35363	-1,89	-1.337,40664	9,73	35,68
**20a**	-1.337,72366	-32,80	-1.337,39766	-29,51	-1.337,44717	-15,71	
**20a-TS**	-1.337,65691	9,09	-1.337,33494	9,85	-1.337,38443	23,66	39,37
**21a**	-1.337,74489	-46,12	-1.337,41829	-42,46	-1.337,46761	-28,53	
**21a-TS**	-1.337,67315	-1,10	-1.337,35127	-0,41	-1.337,40477	10,90	39,43
**22a**	-1.337,73509	-39,97	-1.337,40876	-36,48	-1.337,45890	-23,07	
**22a-TS**	-1.337,64893	14,09	-1.337,32693	14,87	-1.337,37738	28,09	51,16

Gaussian Archive Entries (Total energies (a.u.), number of imaginary frequencies (for transition states: imaginary frequencies), coordinates)

Thiobenzophenone 18

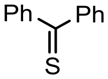

HF=- 899.0655657 a.u. (0)

1\1\GINC-LUPUS04\FOpt\RPBE1PBE\def2TZVP\C13H10S1\WURTHWE\28-Oct-2018\0\\# pbe1pbe/def2tzvp emp=gd3bj opt=readfc geom=check guess=read nosym pop=NBO Freq

scrf=(solvent=dichloromethane)\\thiobenzophenone\\0,1\C,-0.0000000002,0.0000000016,
0.0422474032\S,-0.0000000008,0.0000000033,1.6831651189\C,1.2556596977,0.0886599162,-
0.7181438092\C,-1.2556596979,-0.0886599146,-0.7181438094\C,1.4067663686,-0.6141018958,-
1.9180115238\C,2.609379483,-0.5829227194,-2.6040845442\C,3.6669131108,0.1737100634,-
2.1197528057\C,3.5226707253,0.8887860721,-0.9364461997\C,2.3325667892,0.8368352312,-
0.2351928676\H,0.5857657484,-1.210624967,-2.2960575492\H,2.7201721437,-1.1504730516,-
3.5204305514\H,4.6035139857,0.2086328711,-2.6640199204\H,4.3432301358,1.4887484931,-
0.5610063183\H,2.2122405282,1.3855087271,0.6909398312\C,-1.4067663696,0.6141018974,-
1.9180115239\C,-2.6093794838,0.5829227193,-2.6040845445\C,-3.6669131105,-0.1737100653,-
2.1197528065\C,-3.5226707241,-0.8887860741,-0.9364462006\C,-2.3325667883,-0.8368352314,-
0.2351928682\H,-0.5857657502,1.21062497,-2.296057549\H,-2.7201721452,1.1504730516,-
3.5204305515\H,-4.6035139852,-0.2086328744,-2.6640199213\H,-4.3432301338,-1.4887484965,-
0.5610063196\H,-2.2122405267,-1.3855087274,0.6909398305\\Version=AS64L-G09RevD.01\HF=-
899.0655657\RMSD=3.671e-09\RMSF=8.835e-07\Dipole=0.,0.,-1.8027329\Quadrupole=6.8705209,-
5.2595661,-1.6109547,3.8639341,0.,0.\PG=C02 [C2(C1S1),X(C12H10)]\\@

19a

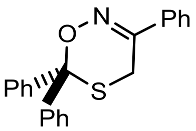

HF=-1337.7388793 a.u. (0)

1\1\GINC-LUPUS08\FOpt\RPBE1PBE\def2TZVP\C21H17N1O1S1\WURTHWE\27-Oct-2018\0\\#
pbe1pbe/def2tzvp emp=gd3bj opt=readfc nosym geom=check guess=read pop=NBO Freq
scrf(solvent=dichloromethane)\\4+2-product\\0,1\C,-0.2507820391,-0.6061099668,0.0010869813\
C,0.0479222593, -0.0315351264,1.23284348\C,1.3665750225,0.3035490899,1.5188569407\
C,2.3696758946,0.061722787,0.5909970279\C,2.0656095364,-0.5086907659,-0.635646504\
C,0.7499017225,-0.839340648,-0.9290248116\C,-1.0503957145,0.2862214562,2.2330554615\O,-
0.4934600336,0.4643846639, 3.5356365606\N,0.0513810244,-0.6028451711,4.2027696324\C,-
0.2047312775,-1.8119200619, 3.8732434551\C,-1.1153430292,-2.2902597021,2.7859647525\S,-
2.3038572725,-1.0363762595, 2.3058195779\C,-1.781820925,1.5812620868,1.9467570572\
C,0.4723009416,-2.8398784449, 4.6975810443\C,0.6431523735,-4.1337551196,4.2071013234\
C,1.292162793,-5.0970471665, 4.9655936081\C,1.7703308385,-4.7841279349,6.2282107345\
C,1.5971493862,-3.4988423393, 6.7284121847\C,0.9560736424,-2.5349216267,5.9714129407\
H,3.3939043006, 0.3221348229, 0.8315760067\H,-1.6859534673,-3.145923456,3.1498508168\H,-
0.5422140381,-2.6199486891, 1.9145300282\H,1.4229472614,-6.0954765688,4.5649218921\
H,0.2816341413,-4.3976743635, 3.2207161559\H,0.8159917933,-1.5345623252,6.359977938\
H,1.9597517488,-3.24894997, 7.7188684806\H,2.2721338044,-5.5378942767,6.8236859194\
H,2.8499698205,-0.6972855493,-1.3593772114\H,0.499887034,-1.2849739492,-
1.884821363\H,1.6099084661,0.7529230726, 2.4727042074\H,-1.2760406115,-0.8742852598,-
0.225995178\C,-2.7174109286 2.0405352195,2.8725965246\C,-3.4124600572,3.2158143935,
2.6423243085\C,-3.1827078417,3.9462503842,1.4833637025\C,-2.2506610592,3.4956673236,
0.562187861\C,-1.5501495018,2.31931042,0.7931195298\H,-2.892723591,1.4722675269,
3.7779222329\H,-4.1359981114, 3.563259164,3.3706224565\H,-3.727869188,4.8653574948,
1.3023315271\H,-2.0609070895,4.0617192563,-0.3423189836\H,-0.8197783036,

1.9805102878,0.0699413905\\ Version=AS64L-G09RevD.01\HF=-1337.7388793\RMSD=8.330e-09\RMSF=3.179e-06\Dipole=0.2410738,-0.9032348,-0.8630217\Quadrupole=-3.2299502,9.1864939,-5.9565436,-5.1718742,0.7689145,-7.2843689\PG=C01 [X(C21H17N1O1S1)]\\@

19a-TS

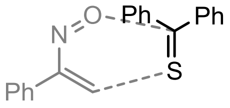

HF=-1337.6748697 a.u. (1, -63,8714cm^{-1})

1\1\GINC-PALMA305\FTS\RPBE1PBE\def2TZVP\C21H17N1O1S1\WURTHWE\30-Oct-2018\0\\# pbe1pbe/def2tzvp emp=gd3bj opt=(ts,noeigentest,readfc) geom=check guess=read freq pop=nbo nosym scrf=(solvent=dichloromethane)\\TS ring-Opening\\0,1\C,0.0230044813, -0.6112891311,0.2010764974\C,-0.0725902247,-0.3328868662,1.5664163903\C,1.0656752836, 0.1183721209,2.2369588463\C,2.261084623,0.2968072633,1.5611651542\C,2.344384379,0.0193537725,0.2030366295\C,1.2213692145,-0.4372930596,-0.472452123\C,-1.3502942424,-0.4970166418,2.2686815419\C,-1.7414120948,0.2057385725,3.3576694921\N,-2.2221211647,-1.4580620175,1.6997199961\O,-3.3526192616,-1.4800021196,2.1452486415\C,-4.6304025926, 1.3259108662,2.0711453882\C,-5.2894762741,0.9366164741,3.3226152322\C,-5.1153195497, 1.6883919839,4.4880847306\C,-5.7076148458,1.294857252,5.6741480643\C,-6.466087814 ,0.1314931246,5.7214590421\C,-6.6356513054,-0.6312314212,4.5739686959\C,-6.0620194489,-0.2282413176,3.3809264822\C,-5.3182713368,1.0666504954,0.7995453478\C,-6.7109148955, 1.1664122108,0.7178435778\C,-7.3593914525,0.976736809,-0.4911599202\C,-6.6316769289, 0.6602945638,-1.6292252162\C,-5.2477140035,0.5475337016,-1.556961288\C,-4.5959235062, 0.7603471557,-0.3575012167\S,-3.1536849529,2.0494435019,2.0869456696\H,-7.2140338055,-1.5467989939,4.6095533651\H,-1.0954805437,0.933383725,3.8286040705\H,-2.6588956411,-0.0648252884,3.8642345606\H,3.1351830636,0.6444067066,2.0996770125\H,1.0208128511,0.3137410193,3.3022155446\H,-0.8528021904,-0.9597703866,-0.3320942286\H,1.2762961141,-0.6538347959,-1.5331716818\H,3.2810468182,0.1556072952,-0.3248482241\H,-6.9239696376,-0.1802168936,6.6530501548\H,-5.5794859518,1.8971155189,6.5658005398\H,-6.1761873052,-0.8364471809,2.4929012045\H,-4.5213795874,2.5932081097,4.4425760454\H,-3.5179175393, 0.6763589286,-0.2941424047\H,-4.6767784035,0.2902243194,-2.4412974887\H,-7.1409627376,0.4998877238,-2.572358045\H,-8.4371687743,1.0755466505,-0.5436584801\H,-7.282300816,1.4201582487,1.6020873995\\Version=EM64L-G09RevD.01\HF=-1337.6748697\RMSD=8.041e-09\RMSF=2.725e-06\Dipole=-0.7641651,0.5665462,0.5870427\Quadrupole=14.0382148,-22.5814293,8.5432145,-0.4705867,-6.9261461,5.038736\PG=C01 [X(C21H17N1O1S1)]\\@

20a

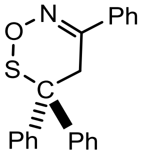

HF=-1337.7236583 a.u. (0)

1\1\GINC-LUPUS05\FOpt\RPBE1PBE\def2TZVP\C21H17N1O1S1\WURTHWE\29-Nov-2018\0\\# pbe1pbe/def2tzvp emp=gd3bj nosym Opt=readfc geom=check guess=read Pop=NBO Freq scrf=(solvent=dichloromethane)\\6-memb.ring\\0,1\O,-0.9942621261,2.4839234852,-0.2791401574\N,-2.1607062024,1.8249134676,-0.6031884368\C,-2.1202009328,0.7415532799,-1.2786865976\C,-0.9152954387,0.0335003595,-1.8312125119\C,0.3970303259,0.3913672574,-1.1450476865\S,0.4067751042,2.2031228951,-1.1623550181\C,1.5773113439,-0.6111031795,-3.1851478386\C,2.744719797,-0.9209031044,-3.8705155296\C,3.9801339335,-0.589443878,-3.3371723697\C,4.045629897,0.0466305638,-2.1044621968\C,2.8826912901,0.3441001405,-1.4151276265\C,1.6335560422,0.0247176582,-1.9497388563\C,0.6438254342,0.5216779853,1.4056413504\C,0.7236032659,-0.1022545027,2.6442798499\C,0.6322946072,-1.4809247098,2.7416780227\C,0.4668224148,-2.2366449936,1.5882006914\C,0.3878704534,-1.6129379556,0.3546817705\C,0.4704478255,-0.2246215176,0.2482542414\C,-4.5229488633,0.9913890064,-1.8778322066\C,-5.7748768878,0.4596780093,-2.136942172\C,-5.9735558648,-0.9137227592,-2.0883616272\C,-4.9118799914,-1.750887702,-1.7782694644\C,-3.6563400453,-1.2209303893,-1.5238430072\C,-3.449173748,0.1569705046,-1.5664465612\H,-0.8622182957,0.2816123533,-2.8938660564\H,-1.0943190454,-1.0413937478,-1.7768455068\H,0.6271869821,-0.8846524954,-3.625099223\H,2.6827273586,-1.4240561333,-4.8284318678\H,4.8889675129,-0.8290984224,-3.8765897148\H,5.0061827972,0.3055963805,-1.6746655645\H,2.9383872477,0.8204786003,-0.4426365459\H,0.7186060835,1.5996751476,1.34332519\H,0.8573650542,0.4989304642,3.5362356582\H,0.6933689963,-1.9666600275,3.7085543196\H,0.4047902492,-3.3171340304,1.6481121293\H,0.2839698834,-2.2198799244,-0.5382144755\H,-4.3599140942,2.060952494,-1.9230986657\H,-6.5979413952,1.1197628605,-2.3849475895\H,-6.9527953604,-1.3301686375,-2.2938209837\H,-5.0602851043,-2.8232811459,-1.7298077123\H,-2.8458915044,-1.8904836567,-1.2622304528\\Version=AS64L-G09RevD.01\HF=-1337.7236583\RMSD=2.951e-09\RMSF=1.794e-06\Dipole=0.3812201,-1.8521726,-0.8393562\Quadrupole=4.4592795,-6.8053159,2.3460364,5.1066365,3.4043184,3.6928583\PG=C01 [X(C21H17N1O1S1)]\\@

20a-TS

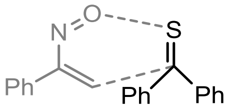

HF=-1337.6569093 a.u. (1, -354.2853cm^{-1})

1\1\GINC-LUPUS04\FTS\RPBE1PBE\def2TZVP\C21H17N1O1S1\WURTHWE\30-Nov-2018\0\\# pbe1pbe/def2tzvp emp=gd3bj nosym Opt=(ts,noeigentest,readfc) geom=check guess=read freq pop=nbo scrf=(solvent=dichloromethane)\\TS\\0,1\C,-0.0779433213,-0.1404371788,0.071883427\C,-0.0495074816,-0.1132413607,1.4700504413\C,1.1785425914,-0.279377367,2.1076973311\C,2.3432807327,-0.4480130653,1.3741387651\C,2.3004515305,-0.4674648241,-

0.0106942099\C,1.0813534581,-0.3174387844,-0.6593073358\C,-1.3041150256,0.0300939122,
2.2534417554\C,-2.3534115076,0.9723482406,1.7691382032\C,-3.6781959828,0.8750912971,
2.2025049483\C,-4.6284972165,1.7911068368,1.7941129117\C,-4.2746035,2.844369883,
0.9581126648\C,-2.9593247605,2.9690459347,0.5428029742\C,-2.0075235236,2.0405806466,
0.9396909595\S,-1.3346526228,-0.3973885567,3.8752200276\C,-2.3553724704,-1.9296514004,
1.5491669306\C,-1.3470779518,-2.8555524179,1.6783496948\N,-0.8764461373,-3.1670035932,
2.9225834966\O,-1.4547238489,-2.5827711561,3.8724346103\C,-0.5941271612,-3.4055404338,
0.5472622912\C,0.7488319616,-3.7575911136,0.6878267926\C,1.4597238349,-4.2531240919,-
0.3919273367\C,0.844670519,-4.3995302233,-1.6278255923\C,-0.492407304,-4.0556004084,-
1.7764790785\C,-1.207019158,-3.5660216044,-0.6966028044\H,-3.0442600363,-
1.7942209926,2.3678219927\H,-2.6434135644,-1.5577668396,0.5758873373\H,-
3.9595839234,0.0773567338,2.8785875305\H,-5.6523398689,1.6867629682,2.1338934118\H,-
5.0195750967,3.5650543815,0.6420576891\H,-2.6632736924,3.7957774957,-0.092434588\H,-
0.9812456829,2.1634747399,0.620481412\H,1.212406009,-0.2698926035,3.1899433467\
H,3.2876611154,-0.5689476326,1.8919326296\H,3.2088612059,-0.6098317151,-0.5841256348\
H,1.0317394038,-0.3534122436,-1.7411697217\H,-1.0230554247,-0.0462389609,-0.4490046257\
H,1.2344984255,-3.611883126,1.6434446787\H,2.5053281352,-4.5121728576,-0.2722245261\
H,1.4052394867,-4.7810284684,-2.4733794223\H,-0.9822736948,-4.1767041044,-2.7355979647\H,-
2.2564483876,-3.3215585487,-0.8153619742\\Version=AS64L-G09RevD.01\HF=-1337.6569093\
RMSD=4.259e-09\RMSF=3.832e-06\Dipole=-0.1091173,0.8452241,-2.4180904\Quadrupole=
16.3492447,-4.0364808,-12.3127639,-5.9312151,2.9799621,10.7725583\PG=C01
[X(C21H17N1O1S1)]\\@

21a

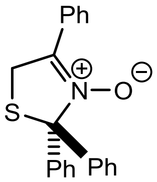

HF=-1337.7448922 a.u. (0)

1\1\GINC-R01N07\FOpt\RPBE1PBE\def2TZVP\C21H17N1O1S1\WURTHWE\28-Feb-2019\0\\#
pbe1pbe/def2tzvp emp=gd3bj Opt=readfc geom=check guess=read nosym Pop=NBO Freq
scrf=(solvent=dichloromethane)\\ 3+2: Nitrosoethene to diphenylthione\\0,1\S,-0.1035455837,-
0.7428133582,-0.1027711181\C,-0.2574379377,-0.4060927076,1.6584487707\C,1.0406229089,
0.1953698052,2.0778412867\N,1.7834803809,0.625160805,1.0886406392\C,1.169696155,0.5339953
385,-0.3006134488\H,-1.0941858134,0.2668691936,1.8716880771\H,-0.4422496847,-
1.3536700423,2.1650040662\C,1.3915538327,0.3257183033,3.4813551798\O,2.8995993121,1.20315
4741,1.1631177539\C,2.1802927947,0.018845396,-1.2981721696\C,0.6502141635,1.9286080457,-
0.6172706047\C,2.6417099445,0.7757034417,3.9321851801\C,2.9048910646,0.867102172,5.289255
8458\C,1.9403667176,0.5256574365,6.2253051245\C,0.697622439,0.0819346081,5.7928056894\C,0
.4278055697,-0.0211536534,4.4412905502\H,3.3939087907,1.0471942255,3.2088541757\
H,3.8788355401,1.2124891073,5.6165691418\H,2.1536133288,0.603893947,7.2849867419\H,-
0.0666737898,-0.186870099,6.5124681799\H,-0.5496894116,-0.3678457934,4.1311562697\C,-
0.7057973831,2.2258761757,-0.6385628488\C,-1.1405853166,3.521403369,-0.8846941983\C,-
0.2218549087,4.5339420219,-1.1095020888\C,1.1361994556,4.2433902153,-1.0912575814\

C,1.570577702,2.9506950309,-0.8503482936\H,-1.427508081,1.4342993475,-0.4778045495\H,-2.2030981855,3.7345490124,-0.9026172087\H,-0.5604898368,5.5450879448,-1.3037871828\H,1.8628022497,5.0274403315,-1.2705225424\H,2.6287198036,2.7261941863,-0.8378443707\C,2.0776212141,0.3803043802,-2.6356396648\C,2.9438945683,-0.1576396204,-3.5782120178\C,3.9257185251,-1.0540702329,-3.1885677163\C,4.0295160714,-1.4212235167,-1.8523250062\C,3.1571571002,-0.8961622493,-0.9149206843\H,1.3170418049,1.0864433618,-2.9456973306\H,2.8495742315,0.1310638215,-4.6185257814\H,4.6084340972,-1.4681895021,-3.9214193929\H,4.7937644541,-2.1229541867,-1.5390400911\H,3.2393294834,-1.1954231774,0.1224751866\\Version=ES64L-G16RevB.01\HF=-1337.7448922\RMSD=6.508e-09\RMSF=1.241e-06\Dipole=-1.1105165,-0.2069688,0.5356401\Quadrupole=-5.2525328,-3.8708334,9.1233662,-4.6962183,-4.8721867,-4.4716897\PG=C01 [X(C21H17N1O1S1)]\\@

21a-TS

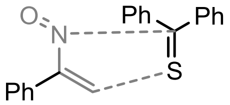

HF=-1337.6731507 a.u. (1, -198.9060 cm^{-1})

1\1\GINC-R02N12\FTS\RPBE1PBE\def2TZVP\C21H17N1O1S1\WURTHWE\06-Mar-2019\0\\# pbe1pbe/def2tzvp emp=gd3bj Opt=(ts,noeigentest,readfc) geom=check guess=read nosym Pop=NBO Freq scrf=(solvent=dichloromethane)\\Nitrosoethene to diphenylthione: 3+2-cycloaddition TS\\0,1\C,0.3496597567,-0.5531649223,0.2751498188\C,0.163206397,0.0451985108,1.5262653303\C,1.1825537588,0.8481575533,2.0532939629\C,2.3486684394,1.0563678508,1.3391233222\C,2.5258216118,0.4496293537,0.1025563259\C,1.5261404213,-0.3597281865,-0.4238110406\C,-1.0841276906,-0.1336598094, 2.266108818\C,-1.0523214887,-0.240982319,3.7218922524\C,-2.1240590919,0.2135211938, 4.4995137913\C,-2.1083802737,0.0615672258,5.8725355325\C,-1.0325892105,-0.5645806309,6.4914808454\C,0.0314544623,-1.030677797,5.7313608121\C,0.0296019067,-0.8626638221,4.3580813397\S,-2.5085798508,-0.2636223542,1.4465275894\N,-2.5779993563,-3.7371536171,1.1365672168\O,-2.5617918994,-4.5251581764,0.1912058805\C,-3.6966258111,-2.9463829271,1.3092492852\C,-4.8731962212,-2.9165618093,0.4322631778\C,-5.332668531,-4.0465533249,-0.253908532\C,-6.4660302431,-3.976429502,-1.0488730455\C,-7.1620352444,-2.7838785223,-1.1870872926\C,-6.7186660847,-1.6556897336,-0.511218948\C,-5.5913390511,-1.7237412171,0.2898533844\C,-3.5482372465,-2.0824915447,2.3868212462\H,-4.3994566514,-1.5562266012,2.796011683\H,-2.7297878623,-2.2777947436,3.0684469928\H,-4.8027562345,-4.9818903045,-0.1564682222\H,-6.80865107,-4.8661752669,-1.5651279514\H,-8.0444504417,-2.7342457784,-1.8145083872\H,-7.2515202688,-0.716660043,-0.6084689185\H,-5.2605207057,-0.8290856985,0.8050509713\H,-2.9551824114,0.7073816707,4.0103116764\H,-2.9348389409, 0.4344374427,6.4654878971\H,-1.0246408653,-0.6886103046,7.5679930556\H,0.8647027548,-1.5305935046,6.2102606635\H,0.8521187168,-1.2415949034,3.7647177183\H,1.0414551261, 1.3299107447,3.0129802831\H,3.1227037881, 1.6946248109,1.7481626162\H,3.4448648895,0.6046506806,-0.4503724586\H,1.6679477347,-0.8452494021,-1.3818990478\H,-0.429701428,-1.1906267093,-0.1240864565\\Version=ES64L-G16RevB.01\HF=-1337.6731507\RMSD=3.608e-09\RMSF=2.487e-06\Dipole=1.1094713, 2.5994936,2.164621\Quadrupole=5.0351179,-27.9675719,22.932454,-10.1718052,-3.9427517,1.9828987\PG=C01 [X(C21H17N1O1S1)]\\@

22a

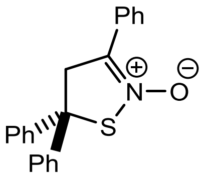

HF=-1337.7350918 a.u. (0)

1\1\GINC-R10N44\FOpt\RPBE1PBE\def2TZVP\C21H17N1O1S1\WURTHWE\23-Apr-2019\0\\#
pbe1pbe/def2tzvp emp=gd3bj Opt=readfc geom=check guess=read scrf=(solvent=dichloromethane)
nosym Pop=NBO Freq\\3+2: Thiobenzophenone + Nitrosoethylene\\0,1\S,-0.9821073765,-
0.1011940823,2.5988256444\C,-1.9838171777,1.383937883,2.9170336806\C,-
3.3992090962,0.8131213794,2.7492203303\C,-3.3674339812,-0.144320996,1.6018337633\N,-
2.1926593659,-0.7079771822,1.4517007681\C,-4.5285952001,-0.4752874221,0.7979145471\C,-
4.4619871077,-1.2553195615,-0.3662987986\C,-5.6067138547,-1.5184116679,-1.0994877911\C,-
6.8397248657,-1.0299830469,-0.6917755219\C,-6.9206265332,-0.2636199576,0.4629316581\C,-
5.7816434373,0.0151372986,1.1955738601\O,-1.8267171288,-1.6085545779,0.6642241524\H,-
3.707455701,0.2746416016,3.6523439432\H,-4.1045646167,1.6268368304,2.5808613239\H,-
3.5084579978,-1.6469336394,-0.6853943759\H,-5.5321909877,-2.1169268066,-2.0001510457\H,-
7.7317927506,-1.2453895898,-1.2681440112\H,-7.8770908978,0.1223169446,0.7956684337\H,-
5.8715987539,0.6134501603,2.0930253644\C,-1.6400935339,1.8750772829,4.3077833245\C,-
1.7615860213,2.488934216,1.8855244815\C,-1.0981898253,2.2921210266,0.6826191705\C,-
0.9868847081,3.3255474209,-0.2393347344\C,-1.5377132284,4.5674842126,0.0309117734\C,-
2.2044283576,4.7721512638,1.232108186\C,-2.3148581959,3.7411699364,2.1496775616\H,-
0.654430514,1.3292700487,0.4603396092\H,-0.4636058819,3.1528935038,-1.1726901943\H,-
1.4468923741,5.3739671454,-0.68730925\H,-2.6362808849,5.7402794148,1.4581449691\H,-
2.8290320905,3.9139376151,3.0889186339\C,-0.3309765339,2.2896959051,4.5521308167\
C,0.044438295,2.7498916381,5.8021885954\C,-0.8910498493,2.820862582,6.8266859607\C,-
2.1975522875,2.4271227149,6.5874053647\C,-2.5705425426,1.9528232043,5.3358092897\
H,0.397724815,2.2611858786,3.74813533\H,1.0680121033,3.0606417267,5.9757859749\H,-
0.6019504611,3.1869016757,7.80479007\H,-2.9375604089,2.4876846196,7.376920577\H,-
3.5990916393,1.6566151385,5.1738724875\\Version=ES64L-G16RevB.01\HF=-1337.7350918\
RMSD=5.561e-09\RMSF=9.593e-07\Dipole=-1.1609929,1.8624366,1.1209789\Quadrupole=
6.7182025,-11.8316884,5.1134859,-6.3348927,-8.0832044,10.8242313\PG=C01
[X(C21H17N1O1S1)]\\@

22a-TS

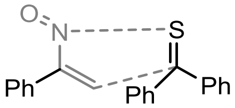

HF=-1337.648934 a.u. (1, -164,3099cm^{-1})

1\1\GINC-R08N34\FTS\RPBE1PBE\def2TZVP\C21H17N1O1S1\WURTHWE\24-Apr-2019\0\\#
pbe1pbe/def2tzvp emp=gd3bj Opt=(ts,noeigentest,readfc) nosym Pop=NBO Freq geom=check
guess=read scrf=(solvent=dichloromethane)\\3+2: Thiobenzophenone + Nitrosoethylene:
TS\\0,1\C,0.0928684491,-1.0608965939,0.4673037158\C,-0.2170215821,-0.3186454782,

1.6150264918\C,0.5306421096,0.8356994718,1.8664544331\C,1.5503609514,1.2263644201,1.0133903709\C,1.856112858,0.4707188472,-0.1090846831\C,1.1190781572,-0.6765411089,-0.3764964895\C,-1.3186876945,-0.7389933894,2.4891779884\C,-1.5584278928,-2.1513604475,2.7939376441\C,-2.8202133061,-2.5823722689,3.2272541722\C,-3.046644919,-3.9052808612,3.5633805938\C,-2.0199583914,-4.8357762318,3.4737258828\C,-0.762448038,-4.4256222755,3.0468155333\C,-0.5316193006,-3.1039090807,2.7115744775\S,-1.9723362381,0.3145622971,3.6393329292\C,-2.9913350056,0.3649444707,0.7443332195\C,-3.7073761446,1.0529545628,1.6784370942\C,-5.1504472291,1.3297026133,1.5459916186\C,-6.0208388683,1.2361414553,2.6332586855\C,-7.3801570435,1.4223132104,2.4577682771\C,-7.8922057153,1.7110616036,1.198110014\C,-7.0338022349,1.8136613057,0.1144850049\C,-5.6704944593,1.6276623723,0.2876688477\N,-3.1393622041,1.3932013161,2.8829948906\O,-3.4635329292,2.3746987937,3.5664056299\H,-0.4962504223,-1.9391158006,0.2300412478\H,1.3368998534,-1.2697041234,-1.257324696\H,2.6578072848,0.7735804406,-0.7721185001\H,2.1172208622,2.1230322457,1.2363440738\H,0.316543859,1.4244233204,2.7508404976\H,0.459352337,-2.7943361872,2.4041755939\H,0.0495458004,-5.1412322047,2.9856025685\H,-2.1974640579,-5.8728812216,3.7328771065\H,-4.0342897728,-4.2142218159,3.8859532665\H,-3.6327455049,-1.8664350915,3.2701760128\H,-3.5340810871,-0.149807395,-0.0399619424\H,-1.9426939492,0.528520334,0.5842876289\H,-5.6331404731,1.0125422538,3.6196218753\H,-8.0463903422,1.3385346427,3.3084349311\H,-8.9573130517,1.8610766755,1.0657480593\H,-7.4227415093,2.048814948,-0.8693176727\H,-4.9973147574,1.7268401938,-0.5556089071\\Version=ES64L-G16RevB.01\HF=-1337.648934\RMSD=5.189e-09\RMSF=3.023e-06\Dipole=-0.0106861,-1.1338866,-1.2447984\Quadrupole=12.5429507,-2.6979678,-9.8449829,6.2485122,3.8542689,-9.5751496\PG=C01 [X(C21H17N1O1S1)]\\@

4. Cycloadducts of s-*trans*-Thiochalcone *(E)*-1,3-diphenylprop-2-ene-1-thione (1a) and α-Phenyl-nitrosoethene (10)

Table S4: Total energies (E_{tot}), energies at 0K E_0), and Gibbs free energies (G_{298})[a.u.], relative energies of the α-phenylnitrosoethylene (**10**)-thiochalcone (**1a)** cycloadducts and transition state energies also with respect to the respective heterocyclic ring (last column)[kcal/mol]

Species	E_{tot} [a.u.]	E_{rel} [kcal/ mol]	E_0 [a.u.]	E_{rel} [kcal/ mol]	G_{298} [a.u.]	To **10** + **1a** E_{rel} [kcal/ mol]	To Ring E_{rel} [kcal/ mol]
**10**	-438,60583	-	- 438,47480	-	-438,50857	-	
**1a**	-976,40819	-	-976,18464	-	-976,22693		
**23a**	-1.415,09400	-50,18	-1.414,73238	- 45,77	-1.414,78517	-31,17	
**23a-TS**	-1.415,00706	4,38	-1.414,65059	5,56	- 1.414,70427	19,59	50,76
**24a**	-1.415,08737	-46,02	-1.414,72556	- 41,49	-1.414,77939	-27,55	
**24a-TS**	-1.415,00968	2,73	-1.414,65341	3,79	- 1.414,70679	18,01	45,56
**11a**	-1.415,07783	-40,03	-1.414,71856	- 37,10	- 1.414,77291	-23,48	
**11a-TS**	-1.415,01449	-0,28	-1.414,65947	-0,01	-1.414,71707	11,57	34,92
***trans*-25a**	-1.415,07004	- 35,15	-1.414,71081	- 32,23	-1.414,76429	-18,07	
***trans*-25a-TS**	-1.415,01160	1,53	-1.414,65558	2,42	- 1.414,70812	17,18	5,25
***trans*-26a**	-1.415,07027	-35,29	-1.414,71035	- 31,95	-1.414,76206	-16,67	
***trans*-26a-TS**	-1.415,02274	-5,46	-1.414,66742	- 5,00	-1.414,72264	8,07	24,74
***cis*-25a**	-1.415,06709	-33,29	-1.414,70757	- 30,20	-1.414,76004	-15,40	
***cis*-25a-TS**	-1.415,01125	1,75	-1.414,65542	2,52	- 1.414,70886	16,72	32,12
***cis*-26a**	-1.415,06429	-31,54	-1.414,70497	- 28,57	-1.414,75926	-14,91	
***cis*-18a-TS**	-1.415,02307	-5,67	-1.414,66756	- 5,09	-1.414,72199	8,48	23,39
**27a**	-1.415,06341	-30,99	-1.414,70400	- 27,96	-1.414,75847	-14,41	
**27a-TS**	-1.415,00008	8,76	-1.414,64440	9,44	-1.414,69824	23,38	37,79
**28a**	-1.415,04837	-21,54	-1.414,68954	- 18,88	-1.414,74342	-4,97	
**28a –TS**	-1.415,00144	7,91	-1.414,64569	8,63	- .414,70042	22,01	26,98

**29a**	-1.415,04723	-20,83	-1.414,68801	- 17,93	-1.414,74144	-3,73	
**29a-TS**	-1.415,01633	-1,44	-1.414,65972	- 0,18	-1.414,71200	14,74	18,47

Gaussian Archive Entries (Total energies (a.u.), number of imaginary frequencies (for transition states: imaginary frequencies), coordinates)

α-Phenylnitrosoethene (**10a**)

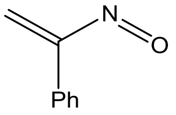

HF=-438.6058288 a.u. (0)

1\1\GINC-LUPUS06\FOpt\RPBE1PBE\def2TZVP\C8H7N1O1\WURTHWE\26-Oct-2018\0\\# pbe1pbe/def2tzvp emp=gd3bj opt=readfc nosym geom=check guess=read pop=NBO Freq scrf(solvent=dichloromethane)\\alpha-Phenyl-nitrosoethene\\0,1\C,0.9775497674,2.1631120294,-0.2765053162\C,0.9149842835,0.8600768206,0.0184567994\N,2.2136369708,0.3035716436,0.2692609276\O,2.2396357589,-0.8572483365,0.586942056\C,-0.447742969,-1.1661477612,-0.5166328454\C,-0.3192313373,0.0668916,0.1227589174\C,-1.3936943969,0.5644081695,0.8582690754\C,-2.577768868,-0.150439903,0.9473596853\C,-2.700078326,-1.3734048556,0.3036722844\C,-1.6336338379,-1.8771875398,-0.4287959985\H,1.9435071136,2.6539411551,-0.2947346042\H,0.0924850237,2.7448632327,-0.5000006881\H,0.3753337681,-1.5627734021,-1.0970544264\H,-1.2926950905,1.5136227633,1.3725202753\H,-3.4043891983,0.2470773458,1.5245745243\H,-3.6245197484,-1.9348027816,0.3736862563\H,-1.7256239138,-2.8301701802,-0.9366599227\\Version=AS64L-G09RevD.01\HF=-438.6058288\RMSD=9.177e-09\RMSF=2.764e-06\Dipole=-1.217586,0.8735551,-0.3362295\Quadrupole=-3.1189947,4.7818753,-1.6628806,3.337494,-3.3084714,2.355975\PG=C01 [X(C8H7N1O1)]\\@

s-trans-(E)-1,3-diphenylprop-2-ene-1-thione (**1a**)

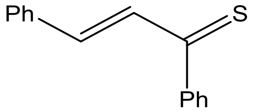

HF=-976.4081868 a.u. (0)

1\1\GINC-R07N17\FOpt\RPBE1PBE\def2TZVP\C15H12S1\WURTHWE\12-Jun-2019\0\\# pbe1pbe/def2tzvp emp=gd3bj opt nosym pop=NBO Freq scrf(solvent=dichloromethane)\\2,4-diphenyl-thioacroleine\\0,1\S,-0.3937724133,0.2694783584,0.143589311\C,-0.0677606607,0.3842485643,1.7563231692\C,1.2972453331,0.3706496826,2.2164513503\C,1.6648227728,0.1204948715,3.490256746\C,3.0187974141,0.0377253092,3.9998794943\C,-1.1667374635,0.4992026438,2.7336488988\C,-1.1159430539,1.4542640328,3.7519075148\C,-2.173034252,1.5938886395,4.637115317\C,-3.2808587884,0.7651898587,4.5351444964\C,-3.3361477832,-0.1936466059,3.5309428977\C,-2.2948910543,-0.3157823118,2.6280247734\H,-0.2577877068,2.1109801724,3.8272064952\H,-2.130136982,2.3537384663,5.408591673\H,-4.1023993147,0.8672405695,5.2346063613\H,-4.1974590898,-0.8463364491,3.4496002608\H,-2.3364964105,-1.0515891705,1.8339877247\C,3.2003081113,-

0.3039649464,5.3445556173\C,4.4688955611,-0.3941567816,5.8912831943\C,5.5810486892,-
0.1415235066,5.1012806215\C,5.4169874331,0.201541967,3.7627873826\C,4.1517124042,0.290573
1681,3.2157641083\H,2.3288146962,-0.500244507,5.9598670994\H,4.5905988333,-
0.6609391408,6.9343931873\H,6.5761284247,-0.2097758084,5.5250741843\H,6.28475705,
0.4013602407,3.1452803187\H,4.0422866244,0.5624962283,2.1729765034\H,0.8855007793,-
0.0641216195,4.2234173979\H,2.0454155196,0.4960209565,1.4419823676\\Version=ES64L-
G09RevD.01\HF=-976.4081868\RMSD=9.644e-09\RMSF=5.044e-06\Dipole=0.9394053,
0.005507,2.2891927\Quadrupole=4.4055356,-14.7576568,10.3521212,1.6533474,
4.8626325,1.1653597\PG=C01 [X(C15H12S1)]\\@

11a

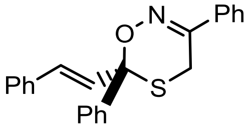

HF=-1415.0778312 a.u. (0)

1\1\GINC-LUPUS09\FOpt\RPBE1PBE\def2TZVP\C23H19N1O1S1\WURTHWE\27-Oct-2018\0\\#
pbe1pbe/def2tzvp emp=gd3bj opt=readfc nosym geom=check guess=read pop=NBO Freq
scrf(solvent=dichloromethane)\\observed product (other configuration)\\0,1\C,-0.6633743538,-
0.3656227087,-0.0081768396\C,-0.4898356099,0.1136562841,1.28099015\H,0.4791885304,
0.4797226708,1.5999477432\C,-1.5544101547,0.1295671207,2.171561431\C,-2.8021000011,-
0.3445884274,1.7835497307\C,-3.9768024077,-0.2789581354,2.7395641231\O,-3.5234357276,-
0.0820095819,4.0822316011\N,-2.864660069,-1.0823079898,4.7496414702\C,-2.8923868004,-
2.2974257362,4.3523912618\C,-3.6250289617,-2.8540192435,3.1727413244\S,-4.9885929741,-
1.7974507396,2.6768102221\C,-4.9062165397,0.8698248223,2.4999769236\C,-4.7636827214,
1.7695055646,1.5286253479\C,-2.1221389757,-3.2455620622,5.1895068803\C,-1.6791305953,-
4.4552004822,4.6560800917\C,-0.9412260936,-5.3396900821,5.429134463\C,-0.6445266623,-
5.0328967496,6.7478893324\C,-1.0892902703,-3.8328768842,7.2899548362\C,-1.8197254382,-
2.9464180871,6.5192537669\H,-4.0509604906,-3.8222514821,3.4385620853\H,-2.9475378962,-
3.0070855593,2.327717914\H,-0.598289595,-6.2719648201,4.9957557067\H,-1.894741678,-
4.7113769115,3.6256944727\H,-2.1710228798,-2.0130411764,6.9398461112\H,-0.8697595263,-
3.5901339036,8.3233159477\H,-0.0733533971,-5.7265804109,7.3537881854\C,-5.6395535194,
2.9049472469,1.2523158122\C,-5.2786145838,3.7829257452,0.2282679896\C,-6.0616429655,
4.8843335022,-0.0797038661\C,-7.2261807077,5.1265119381,0.6329427084\C,-7.6006215171,
4.2584962225,1.6530164274\C,-6.8183787191,3.1598987288,1.9598112664\H,-4.3672859272,3
.5948385219,-0.3295966908\H,-5.7615310832,5.5537835562,-0.8775061424\H,-7.8429724536,
5.9856545745,0.3962609447\H,-8.5116064935,4.4407257161,2.2114897342\H,-7.1303336898,
2.4923901362,2.7544406753\C,-1.9098168611,-0.8324476345,-0.4035047152\C,-2.9703214957,-
0.8256531102,0.488042228\H,0.1685960577,-0.3773480871,-0.7026767637\H,-2.0574390005,-
1.207313964,-1.4096646688\H,-1.414894283,0.510865878,3.1745689429\H,-3.9404935859,-
1.1994856172,0.1824142616\H,-5.7258135462,0.9220628767,3.2101932727\H,-3.9140146294,
1.6744545368,0.8584964835\\Version=AS64L-G09RevD.01\HF=-1415.0778312\RMSD=9.270e-
09\RMSF=4.145e-06\Dipole=0.3998584,-0.6333685,-1.031003\Quadrupole=-3.2623156,11.895825,-
8.6335094,-5.0689817,7.5828014,-8.5540023\PG=C01 [X(C23H19N1O1S1)]\\@

11a-TS

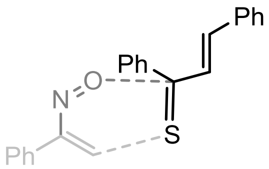

HF=-1415.014488 a.u. (1, -125,0284cm^{-1})

1\1\GINC-PALMA337\FTS\RPBE1PBE\def2TZVP\C23H19N1O1S1\WURTHWE\27-Oct-2018\0\\# pbe1pbe/def2tzvp emp=gd3bj opt=(ts,noeigentest,readfc) geom=check guess=read pop=nbo freq nosym scrf=(solvent=dichloromethane)\\observed product: TS-Search Diels-Alder\\0,1\C,-0.0080312767,-0.1745678123,0.0700627954\C,-0.0292537483,-0.1750050086,1.4671335887\C,1.1735973087,0.0128906264,2.1516590375\C,2.3591047288,0.2044256094,1.4620223275\C,2.3681981124,0.2026696014,0.0735186379\C,1.1801894822,0.0095679494,-0.6179645044\C,-1.2903372346,-0.357906494,2.1919862579\C,-1.5570755968,0.1429344305,3.4343066711\N,-2.2908950132,-1.0521049979,1.4988070404\O,-3.3992273257,-1.0467913331,2.0174971679\C,-4.3254178389,1.6992128511,2.5822353701\C,-5.0378496838,1.9079619031,1.3081223816\C,-4.9653737426,3.1391861398,0.6554899461\C,-5.6188919733,3.3353482393,-0.5489773477\C,-6.3360360537,2.2976415335,-1.1298160047\C,-6.4015751293,1.0649191603,-0.4957409211\C,-5.7664313716,0.8714028884,0.7202216934\C,-4.9926297588,1.1091979878,3.7048697998\C,-6.3283592294,0.9325067958,3.7883876722\C,-7.0559788575,0.3825122203,4.9130365072\C,-6.4296920616,-0.1339912717,6.0550708997\C,-7.1798760236,-0.6449533938,7.0960986221\C,-8.5693868208,-0.6538495055,7.0225445567\C,-9.2047459837,-0.149388063,5.8969425307\C,-8.4540315958,0.3621549149,4.8525302088\S,-2.7495249998,2.1985692637,2.7158351193\H,-0.8045847769,0.6650230549,4.0084678641\H,-2.4388995758,-0.2038333842,3.9549821105\H,3.282682744,0.3453463956,2.0116604889\H,1.1863310643,-0.0105934951,3.23528529\H,-0.9346742933,-0.3111662479,-0.4728657221\H,1.1766443552,0.0112611344,-1.7019826873\H,3.2966556272,0.3492166836,-0.4660093858\H,-4.4009894537,3.9449763629,1.1098308678\H,-5.5668808545,4.3012999912,-1.0374518823\H,-6.8396509667,2.4487856568,-2.077513444\H,-6.9454129459,0.2461111317,-0.9521242013\H,-5.7927452495,-0.0978229353,1.2021166471\H,-4.3599143384,0.864925317,4.5505982966\H,-6.9420898984,1.2501899626,2.9511196025\H,-8.9480275486,0.7562363038,3.9709252145\H,-10.2863794534,-0.1558338552,5.8329176529\H,-9.1528911932,-1.0564928298,7.842172415\H,-6.6821153659,-1.0424463682,7.9727575298\H,-5.3489553349,-0.1409758747,6.1271019913\\Version=EM64L-G09RevD.01\HF=-1415.014488\RMSD=6.069e-09\RMSF=1.466e-06\Dipole=-1.2684839,0.5199985,1.1907444\Quadrupole=19.649427,-26.6262849,6.9768578,-2.7473794,-14.7081313,-5.3010742\PG=C01 [X(C23H19N1O1S1)]\\@

23a

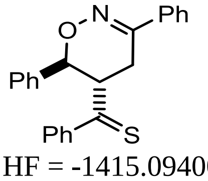

HF = -1415.0940042 a.u. (0)

1\1\GINC-R02N21\FOpt\RPBE1PBE\def2TZVP\C23H19N1O1S1\WURTHWE\09-Jun-2019\0\\# pbe1pbe/def2tzvp emp=gd3bj opt freq pop=nbo scrf=(solvent=dichloromethane) nosym int=(Acc2e=11,grid=ultrafine)\\Cycloadduct after PCM\\0,1\N,-0.3787374983,-1.7348185468,-2.0283747329\O,0.6855534733,-1.164373055,-1.378069444\C,0.7460453038,-1.2916747811,0.0373653561\C,-0.5980869644,-0.8437876473,0.6400864683\C,-1.6475450597,-1.7837384136,0.0868316617\C,-1.4329697212,-2.0401139013,-1.3699431051\C,1.9161456478,-0.4875131967,0.5113705531\C,2.0365029436,0.856505103,0.170969366\C,3.080298889,1.6143376888,0.67572203

4\C,4.012151959,1.0368035665,1.5288697772\C,3.9010625024,-0.3038455949,1.8651783311\
C,2.8583596289,-1.0634194784,1.3538180085\C,-3.5480275612,-3.2050448531,-4.269988758\C,-
2.525616086,-2.6162261621,-3.5488996162\C,-2.5248525171,-2.6583014493,-2.1523117974\C,-
3.5771236658,-3.3024871888,-1.5028140721\C,-4.5995417459,-3.8971317923,-2.2285110028\C,-
4.5900367819,-3.8501707427,-3.6133649553\C,-0.4769115377,-0.8389299852,2.1380841624\S,-
0.4975645448,-2.2377870277,2.9799613882\C,-0.239878701,0.4562777182,2.7843764319\C,-
0.8547522015,1.6207268223,2.3130062574\C,-0.633325114,2.8354709538,2.9386586891\
C,0.2337171958,2.9139968077,4.0191682731\C,0.8613005924,1.7661375832,4.4879973939\C,0.614
7647453,0.5467191026,3.8870743854\H,0.8851191966,-2.349096341,0.2966197295\H,-
0.7701982158,0.1716438372,0.2794526011\H,-2.6472361743,-1.3659661788,0.2293807545\H,-
1.6147078254,-2.7237435264,0.6478092546\H,1.3082200087,1.3107517052,-0.4912234607\
H,3.1651116611,2.6609607477,0.4073220588\H,4.8250710896,1.6321797807,1.9281407088\H,4.627
5462201,-0.7616699682,2.5264220011\H,2.7674131737,-2.1108769418,1.6197153316\H,-
3.536141664,-3.1581463677,-5.3529035065\H,-1.7152563048,-2.112075541,-4.0593277719\H,-
3.601655382,-3.3541013806,-0.4213238063\H,-5.4057671362,-4.3982247274,-1.7055014694\H,-
5.3906312725,-4.3103138357,-4.1807528841\H,-1.5391300863,1.5769580799,1.4748362781\H,-
1.1346450535,3.7251106666,2.5765987448\H,0.4232443688,3.8693305674,4.4945018674\H,1.5487
12322,1.8251093836,5.3234827381\H,1.1038488937,-0.3522504894,4.2405477767\\Version=ES64L-
G09RevD.01\HF=-1415.0940042\RMSD=8.934e-09\RMSF=4.194e-06\Dipole=-0.4794721,
1.4056272,0.9376351\Quadrupole=5.0502123,1.1770666,-6.2272788,5.7847975,11.2106117,
6.2513393\PG=C01 [X(C23H19N1O1S1)]\\@

23a-TS

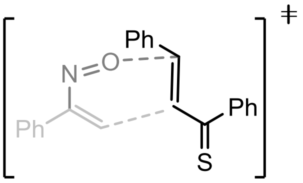

HF = -1415.0070587 (1, -339.2710 cm^{-1})

1\1\GINC-R02N13\FTS\RPBE1PBE\def2TZVP\C23H19N1O1S1\WURTHWE\09-Aug-2019 \0\\#
pbe1pbe/def2tzvp emp=gd3bj opt=(ts,noeigentest,readfc) geom=check guess=read
scrf=(solvent=dichloromethane) pop=nbo freq nosym\\ 23a-TS-Search \\0,1\C,0.0739365456,
0.190464675,0.035400761\C,0.0620058519, 0.1416750235,1.4213928054\C,1.2445965665,-
0.0024862703,2.2279199194\C,2.4833977396,-0.3641462802,1.685268838\C,3.5845686662,-
0.5380419463,2.5047519958\C,3.4718468753,-0.3430279509,3.8743256467\ C,2.2489594237,
0.0244022888,4.4274977341\C,1.1460546977,0.1859694204,3.6138109228\H,1.0358995482,0.25763
94468,-0.4563662189\H,-0.8614009676, 0.4020267205,1.9250895643\H,2.5913713134,-
0.5193257058,0.6196055244\H,4.5349686988,-0.8282493429,2.0730528129\H,4.3368165185,-
0.4765899604,4.5134718427\H,2.1603814539, 0.1763788988,5.4965334288\ H,0.1860748915,
0.454091152,4.0405092744\C,-1.069443696,0.690092181,-0.7246734669\C,-0.7808605313,
1.1858145182,-2.0817693284\S,-2.6023895092,0.6483781729,-0.1321675855\O,-0.645999684,-
2.0416673341,1.8584899302\ N,0.3006762166,-2.7752866164,1.5197433431\C,0.6637577821,-
2.6624321721,0.2014863273\C,-0.1257840929,-1.8737530174,-0.6141695462\H,-1.1633203948,-
1.7630245869,-0.3260244356\H,0.1309043255,-1.7204401716,-1.6544965676\C,1.8976314273,-
3.3408907511,-0.2123959858\C,2.1392778203,-3.6316900061,-1.5565699507\C,3.3070905154,-
4.2677568334,-1.9458896929\C,4.2501779521,-4.6385748974,-0.9977886714\C,4.0156609947,-
4.3644736509,0.3434353783\C,2.8556109624,-3.717275905,0.7330945467\H,2.6849972696,-
3.4824091642,1.775647969\H,4.748217905,-4.6464753804,1.0911459979\H,5.1619208061,-
5.1393986294,-1.3018851416\H,3.4748744253,-4.4850465417,-2.9944390349\H,1.3997611535,-
3.3775989096,-2.3068740161\C,-1.7210288711,1.034727237,-3.1046975147\C,-1.4554733296,
1.4965661719,-4.3803712077\C,-0.2542762363,2.1396174426,-4.6535664991\ C,0.6824532511,

2.3093915592,-3.6441008868\C,0.4290181284,1.8248487164,-2.3716822828\H,-2.6557854298, 0.5354144803,-2.8811499514\H,-2.1871942295,1.3547833712,-5.1669357517\H,- 0.0495061133,2.5076961517,-5.6521130147\H,1.6150567609,2.8223153571,-3.847020866\ H,1.1628136436,1.983564373,-1.5908872547\\Version=ES64L-G16RevB.01\HF=- 1415.0070587\RMSD=5.225e-09\RMSF=3.897e-06\Dipole=2.4467377,0.8547036,- 1.4358569\Quadrupole=0.4689842,-13.5866213,13.1176372,- 2.7836648,5.7927831,4.6768502\PG=C01[X(C23H19N1O1S1)]\\@

24a

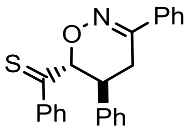

HF=-1415.0873733 a.u. (0)

1\1\GINC-LUPUS08\FOpt\RPBE1PBE\def2TZVP\C23H19N1O1S1\WURTHWE\27-Oct-2018\0\\# pbe1pbe/def2tzvp emp=gd3bj opt=readfc nosym geom=check guess=read pop=NBO Freq scrf(solvent=dichloromethane)\\4+2-Product \\0,1\C,1.2982834271,0.1081373701,0.8410001638\ C,0.0404228424,-0.5175561307,1.3595267154\C,-1.1916872539,0.0667636365,0.6919211531\C,- 0.957135724, 1.574445865,0.6175333674\O,0.206923505,1.8662270242,-0.1571580538\ N,1.3549997835, 1.1742885001,0.1365802495\C,-2.478286121,-0.2691920836,1.3927284214\C,- 2.666752193, 0.027820377,2.740333906\C,-3.8722100276,-0.2529373957,3.3644310036\C,- 4.9090412305,-0.8367593088,2.6490684728\C,-4.731568364,-1.1363349436,1.3064759557\C,- 3.5244387533,-0.8517307942,0.6846117799\C,-2.1205488826,2.3336822739,0.0141323771\S,- 2.7961155313, 3.5221515073,0.898505558\C,-2.641526514,1.9221339725,-1.2941362577\C,- 4.00403045, 2.0802029915,-1.5738936273\C,-4.5298461574,1.6737499901,-2.7844792905\C,- 3.7021142372,1.1149083748,-3.7511783729\C,-2.3477741607,0.960981365,-3.4932285945\C,- 1.8200546255,1.3495659867,-2.2733599677\C,3.7521304692,-0.177284635,0.4279414696\ C,2.5932237366,-0.552389894,1.1130552202\C,2.6805331407,-1.5652102507,2.0673435789\ C,3.8937514042,-2.1833410569,2.3355921096\C,5.0362467876,-1.8040702085,1.6497903432\ C,4.9586244977,-0.7979321865,0.6931989675\H,-0.0105433132,-0.3856780383,2.4447656428\ H,0.0835120033,-1.5945955369,1.1820900758\H,-1.2506993644,-0.3087393362,-0.3320576426\H,- 0.7965302583,1.9632216873,1.6283238519\H,-1.8707493499,0.4920551058,3.3129423562\H,- 4.0034280989,-0.0121180753,4.4130791878\H,-5.8515026157,-1.0562270713,3.1371609322\H,- 5.5351892322,-1.5915104852,0.7390365018\H,-3.3921149357,-1.0783750091,-0.3680682962\H,- 4.6444809598,2.5051327112,-0.8111642158\H,-5.5904219418,1.7869151566,-2.9754328847\H,- 4.1138845382,0.7989115134,-4.70263399\H,-1.6953390541,0.5354498674,-4.2464281809\H,- 0.7606553985,1.2416141705,-2.0888573071\H,3.6914291969,0.6050073152,-0.3175013729\ H,1.8014055654,-1.8768542848,2.6175862859\H,3.9415341833,-2.9654087767,3.0843844392\ H,5.9830872573,-2.2896196908,1.8554710579\H,5.8465024864,-0.4983595693,0.1482739103\\ Version=AS64L-G09RevD.01\HF=-1415.0873733\RMSD=6.158e-09\RMSF=1.534e-06\Dipole=- 0.4779091,-2.1364101,-0.1556426\Quadrupole=9.029754,-17.3769212,8.3471672,1.5736427, 5.8813505,-3.5732012\PG=C01 [X(C23H19N1O1S1)]\\@

24a-TS

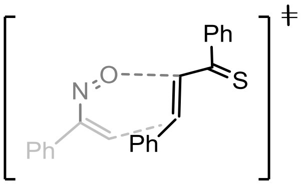

HF=-1415.0096792 a.u. (1, -321.6155 cm^{-1})

1\1\GINC-R03N14\FTS\RPBE1PBE\def2TZVP\C23H19N1O1S1\WURTHWE\12-Aug-2019 \0\\# pbe1pbe/def2tzvp emp=gd3bj opt=(ts,noeigentest,readfc) geom=chec k guess=read scrf=(solvent=dichloromethane) pop=nbo freq nosym\\Produc t from TS-Search 24a-TS\\0,1\C,-0.0046032675,-0.5423846496,0.1079444956\C,-0.0849774558,0.039600256, 1.3765575007\ C,1.0703426504,0.5990933695,1.9327309834\C,2.2704473666,0.5620500691,1.2423413732\C,2.333 5155262,-0.0083911982,-0.0209036949\C,1.1895677752,-0.5572790876,-0.5879096473\C,-1.3678252548,0.0554873333,2.1008815191\S,-2.4712452357,-1.159823713,1.9046126378\C,-1.607105935,1.172718594,2.9794527888\C,-2.8304895797, 1.4284699556,3.5792711595\C,-3.1410135405,2.695072984,4.244664912\C,-2.1474654539, 3.5786589521,4.6707012392\C,-2.4830062105,4.7489123318,5.330461974\C,-3.8131450209, 5.0543363682,5.5821733239\C,-4.809975788,4.1814334241,5.1669047175\C,-4.4755269946, 3.0126125898,4.5054058403\C,-2.8581296471,-0.0233521879,5.1501628753\C,-2.0591643972, 0.5648071735,6.1098280975\N,-0.7391999895,0.7971844247,5.8132515226\O,-0.3397424402, 0.3144110324,4.7424523585\C,-2.5686285087,1.1789378812,7.3376113993\C,-1.8957732382, 2.2442181204,7.9421994154\C,-2.3929956863,2.8253698015,9.0958945509\C,-3.5706725653, 2.3594245006,9.6649326611\C,-4.2448968485,1.2985007332,9.0755377746\C,-3.7468318624, 0.7115668944,7.9249508167\H,-3.9272586161,-0.0773653221,5.3104196329\H,-2.4300711144,-0.7325167579,4.4499223912\H,-3.6744103914,0.8642269659,3.1900819276\H,-0.8402015818, 1.9338115781,3.0112924266\H,-1.1021522815,3.3495947667,4.5022318618\H,-1.6999035577, 5.4223268364,5.6585544928\H,-4.0723709156,5.968354512,6.1033589343\H,-5.8508670027, 4.4118505258,5.361282688\H,-5.2554441347,2.3288795399,4.1869889562 \H,1.0411799834, 1.0154775018,2.9311607933\ H,3.162054299,0.9785354099,1.6961332952\H,3.2715323639,-0.0260084948,-0.5636064211\ H,1.2302303224,-0.9965080359,-1.5777328124\H,-0.9004699111,-0.9682128985,-0.3271617098\H,-0.9912147754,2.6226634808,7.4858383441\H,-4.2685306749,-0.1326321039,7.4894055409\H,-5.1590443076,0.9209733244,9.5186330349\H,-3.9607468745,2.8198499649,10.565189676\H,-1.8640083837,3.6565091071,9.5478374965\\ Version=ES64L-G16RevB.01\HF=-1415.0096792\RMSD=7.564e-09\RMSF=1.081e-06\Dipole=-0.7894625,1.5626068, 0.5481521\Quadrupole=4.9174797,-7.2874109,2.3699312,-5.8178319,-22.0365065,18.5383392\ PG=C01[X(C23H19N1O1S1)]\\@

trans-**25a**

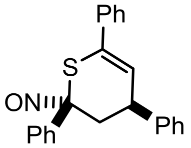

HF=-1415.0700441 a.u. (0)

1\1\GINC-LUPUS04\FOpt\RPBE1PBE\def2TZVP\C23H19N1O1S1\WURTHWE\26-Oct-2018\0\\# pbe1pbe/def2tzvp emp=gd3bj opt=readfc nosym geom=check guess=read pop=NBO Freq scrf(solvent=dichloromethane)\\4+2-Product \\0,1\C,-0.4513549189,-2.6849953363, 4.195517312\C,0.512879974,-2.3938332735,3.2293096819\C,1.8076905868,-2.8828657588,3.4053574933\C,2.123293649,-3.6609272973,4.508442494\C,1.1539883293,-3.9559866648,5.4564608191\C,-0.1345419738,-3.4641451765,5.2962004272\S,1.3811373941,-

0.4117203724,1.5963815653\C,0.7908943487,0.1091075514,-0.0230305732\C,-
0.7161884003,0.3237168919,-0.022930716\C,-1.4989550445,-0.9649961893,0.2263011169\C,-
1.007544368,-1.7433599413,1.4036949738\C,0.1521636847,-1.5860496487,2.0478541989\C,-
3.4873059691,-0.135657025,1.5385286748\C,-4.8289991492,0.1981990412,1.6407313407\C,-
5.673978691,0.0306384747,0.5514026489\C,-5.1674854783,-0.4727694915,-0.6379665547\C,-
3.823499782,-0.8059010942,-0.7363430751\C,-2.9698982615,-0.6407088302,0.3484499012\
C,1.6053399325,1.3155216631,-0.4314071804\C,2.9804943857,1.163587445,-0.60646743\
C,3.7670570235,2.244542114,-0.9701793109\C,3.1873145448,3.4899224803,-1.1664647076\
C,1.8191637985,3.6462716669,-0.9986920002\C,1.0297740755,2.5646971776,-0.6376437502\
N,1.0217029813,-0.9034642839,-1.1365587913\O,1.5752589085,-1.9061382919,-0.8354841238\H,-
1.4512559798,-2.2813670543,4.0853706055\H,2.5646959166,-2.6657564525,2.6607428752\
H,3.131425704,-4.0417085375,4.6249103258\H,1.4027142641,-4.5612269964,6.3204423504\H,-
0.8945323303,-3.678423367,6.0388445966\H,-1.019130385,0.7348462123,-0.988795503\H,-
0.9584430314,1.0613183141,0.7445139555\H,-1.3903935188,-1.6018616991,-0.6633809385\H,-
1.6589869549,-2.542855549,1.7441333334\H,-2.8308316506,-0.0080872067,2.3934849582\H,-
5.2176092215,0.5881475865,2.5747276859\H,-6.7236998924,0.2884845154,0.631235331\H,-
5.8205610511,-0.610533352,-1.4922275976\H,-3.4310614373,-1.2024174007,-1.6674325623\
H,3.4401107483,0.1937678849,-0.4506219574\H,4.8343739203,2.1123770508,-1.1022675762\
H,3.800585624,4.3368662054,-1.4510569893\H,1.3600708227,4.6162430345,-1.1495925796\H,-
0.035345127,2.7068939807,-0.510378748\\Version=AS64L-G09RevD.01\HF=-1415.0700441\
RMSD=4.461e-09\RMSF=1.498e-06\Dipole=-0.5714183,0.8673386,0.1483813
\Quadrupole=5.8696069,-2.7391967,-3.1304102,2.2424044,-1.8590086,-7.3533771\PG=C01
[X(C23H19N1O1S1)]\\@

trans-**25a**-TS

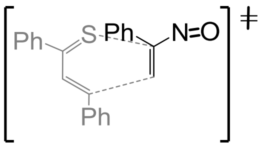

HF=-1415.0115979 a.u. (1, -353.1666 cm^{-1})

\1\GINC-R07N17\FTS\RPBE1PBE\def2TZVP\C23H19N1O1S1\WURTHWE\13-Aug-2019 \0\\#
pbe1pbe/def2tzvp emp=gd3bj opt=(ts,noeigentest,readfc) geom=chec k guess=read
scrf=(solvent=dichloromethane) pop=nbo freq nosym\\TS-Search trans-25a\\0,1\C,0.1617761449,-
0.0692523607,0.0189888477\C,0.0089151677,0.022848065,1.4054209761\C,1.1588390544,0.165509
7401,2.1946107348\C,2.4091369905,0.2025840495,1.6116342567\C,2.5488944043,0.0908491004,0.
2307061212\C,1.4215544972,-0.0433190728,-0.5607535617\C,-1.319525606,-0.0540096184,
2.0025962935\N,-1.3643401352,-0.0861231983,3.4285391739\O,-2.4652058582,-0.0259503461,
3.9321426559\C,-2.4860380324,0.06082423,1.2680516409\C,-3.109510543,-1.8471318073,
0.6796677507\C,-4.2705550196,-1.4980295071,-0.1473458795\C,-4.1711481559,-1.2418139354,-
1.5164386086\C,-5.2968875271,-0.919109479,-2.2537841438\C,-6.540174892,-0.8456962025,-
1.6375411239\C,-6.6500190795,-1.0915181758,-0.2762953419\C,-5.5223576975,-1.4080779826,
0.4631880396\C,-1.9831328827,-2.488078965,0.16716395\C,-0.9472382617,-2.966657466,
0.9836229711\C,0.2222861309,-3.6021942509,0.3434139136\C,0.0579261526,-4.3964432327,-
0.7924691065\C,1.1526071784,-4.9880769343,-1.4028014003\C,2.4278738205,-4.7832085735,-
0.8952642987\C,2.5999537307,-3.9922276931,0.2336324355\C,1.5060075987,-3.4147865259,
0.8537583338\S,-1.0054751839,-2.7156858257,2.636968716\H,1.634165937,-2.7911468257,
1.7298144604\H,3.5938411183,-3.8212600845,0.6309701\H,3.284729949,-5.2411027537,-
1.3757513095\H,1.0075819422,-5.6150823681,-2.2749037225\H,-0.936081691,-4.5758461047,-
1.1862239572\H,-1.8544683512,-2.5575425038,-0.907041823\H,-3.3133251465,-2.0601398993,
1.7282240245\H,-3.2079923979,-1.2854572192,-2.011746244\H,-5.2051796651,-0.7216078326,-
3.3153582245\H,-7.4198267686,-0.5931894313,-2.2178419936\H,-7.6158038716,-1.0338022514,

0.2116708648\H,-5.6070699049,-1.5956373712,1.5283679367\H,-3.3949162246,0.2399342964,
1.8280548873\H,-2.4514080913,0.4650149655,0.2669232934\H,1.0558760589,0.2382189068,
3.2687649235\H,3.2873550202,0.3160518298,2.2366781924\H,3.5337383077,0.1134084785,-
0.2208336781\H,1.5169498012,-0.1281578562,-1.6368021099\H,-0.7052809683,-0.162255122,-
0.6210763423\\Version=ES64L-G16RevB.01\HF=-1415.0115979\RMSD=5.225e-09\RMSF=1.476e-
06\Dipole=-0.2515632,0.106622,-3.157593\Quadrupole=21.5202101,-7.1518375,-14.3683726,-
1.5153775,11.0915914,9.7256886\PG=C01[X(C23H19N1O1S1)]\\@

trans-**26a**

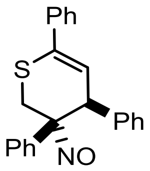

HF=-1415.0702655 a.u. (0)

1\1\GINC-LUPUS08\FOpt\RPBE1PBE\def2TZVP\C23H19N1O1S1\WURTHWE\27-Oct-2018\0\\#
pbe1pbe/def2tzvp emp=gd3bj opt=readfc nosym geom=check guess=read pop=NBO Freq
scrf(solvent=dichloromethane)\\Product trans-26a\\0,1\C,0.3908700752,-
0.9604394927,0.4547433424\C,-0.0800565337,-0.054099701,1.3986426754\C,0.7427742111,
1.009748242,1.7732214217\ C,2.0002058203,1.1637950609,1.2130158348\C,2.4548655896,
0.2628407314,0.2607705637\C,1.6450127803,-0.7971507326,-0.116402971\C,-1.4517146977,-
0.1801483157,2.0283364811\C,-2.142785671,-1.4839771341,1.6916092876\S,-3.6716603059,-
1.763480755,2.605833109\C,-4.2390192807,-0.1387530801,2.896709947\C,-3.6083400505,
0.9742554885,2.4974966824\C,-2.3384616977,1.0392013658,1.7122762716\N,-1.1841423689,-
0.0454748499,3.5240498221\O,-1.1471968824,-1.078504636,4.1105589381\H,-1.7817529971,
1.9121699347,2.0638897917\C,-5.5018529778,-0.0876409638,3.6606417814\C,-5.6718551854,
0.8614902549,4.6691568874\C,-6.8610386644,0.93373242,5.375975486\C,-7.8965157256,
0.0526860498,5.0945871313\C,-7.7327435465,-0.9025202454,4.101729615\C,-6.5441315776,-
0.974972295,3.3918176914\H,-4.8557658554,1.5329123803,4.9091845407\H,-6.4285409051,-
1.7144835873,2.6075723911\H,-8.5359495328,-1.5937023269,3.8739158586\H,-8.8248640948,
0.1071220838,5.6511273605\H,-6.9751529638,1.6745139203,6.159039496\H,-2.3541217122,-
1.5084835942,0.6228940873\H,-1.5104968345,-2.3360435011,1.9413526263\C,-2.5680951289,
1.2172034781,0.2271315789\H,-4.0910345506,1.922954351,2.7087858547\H,0.3991908182,
1.7261881957,2.5094889944\H,2.624892896,1.9947040327,1.5195700192\H,3.4349615762,0.38663
84325,-0.1846933703\H,1.9871569646,-1.5055478393,-0.8618821847\H,-0.219691643,-
1.7962173258,0.1384114862\C,-1.6973378881, 2.0020407788,-0.5250662948\C,-1.8698719902,
2.1419179741,-1.8935027496\C,-2.921349265, 1.4993096966,-2.5324749233\C,-3.8016155997,
0.7248397299,-1.7910393077\C,-3.6276043494, 0.5885501419,-0.4211240234\H,-0.8737406775,
2.5058153131,-0.0318989238\H,-1.1817641223, 2.7571501284,-2.4619874872\H,-3.0585495166,
1.6089312238,-3.6020445506\H,-4.632462441, 0.2278206818,-2.2787517276\H,-4.3307109683,-
0.0044109222,0.1533427881\\Version=AS64L-G09RevD.01\HF=-1415.0702655\RMSD=4.271e-
09\RMSF=2.511e-06\Dipole=0.2195451, 0.5307097,-1.3202483\Quadrupole=9.0621343,0.8780166,-
9.9401509,1.508315,-2.013716, 8.7695876\PG=C01 [X(C23H19N1O1S1)]\\@

trans-**26a**-TS

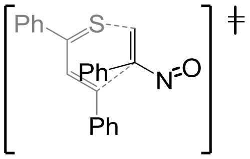

HF=-1415.0227357 a.u. (1, -77.2273 cm^{-1})

1\1\GINC-R03N23\FTS\RPBE1PBE\def2TZVP\C23H19N1O1S1\WURTHWE\09-Jun-2019\0\\#
pbe1pbe/def2tzvp emp=gd3bj Opt=(ts,noeigentest,calcfc) freq pop=nbo
scrf=(solvent=dichloromethane) nosym\\Product TS-Search for E-Thiochalcone\\0,1\
C,0.0357173895,0.0447765856,-0.0268282762\C,-0.006355547,-0.0915410614,1.3638630536\
C,1.1976558184,-0.2772303393,2.051415179\C,2.4024979541,-0.3103880181,1.3701216883\
C,2.4271621444,-0.1841050912,-0.0114611753\C,1.237534968,-0.0107724817,-0.7080388823\C,-
1.2934253126,-0.0284036898,2.0812436334\S,-2.5457352148,0.8877624125,1.4751234964\C,-
1.4058912164,-0.7499658313,3.309712185\C,-2.5525223857,-0.8587128406,4.0208876881\C,-
2.7038815324,-1.5353329763,5.2872609215\C,-1.6109570213,-1.9140052472,6.0783593479\C,-
1.8092622961,-2.5479575048,7.2894484993\C,-3.0988934833,-2.8165493268,7.7362762014\C,-
4.19205267,-2.4379966683,6.9683525694\C,-3.9958551515,-1.7968384906,5.7586734117\C,-
2.8784225695,2.6201701457,3.3985656576\C,-2.5257660468,2.0831371171,4.5953948324\C,-
1.1530694486,1.9899151275,5.1032687892\N,-3.5324699741,1.635543408,5.4867118929\O,-
4.6606466065,1.5549583916,5.046196797\H,1.2035896792,-0.3490286267,3.131048672\H,-
0.8936204811,0.1832952825,-0.565669693\H,1.2479747363,0.0803808642,-1.7877842276\
H,3.3700229437,-0.2201083082,-0.5446205939\H,3.3267255319,-0.4318377698,1.9227375415\H,-
3.9305676168,2.6976805893,3.1586865265\H,-2.1811802738,3.1887707856,2.8017378138\H,-
3.4519826797,-0.4339891543,3.5829901922\H,-0.513635789,-1.2464400848,3.6701286524\H,-
0.6029151957,-1.6882041782,5.7532769644\H,-0.9563934096,-2.8292858026,7.8959084729\H,-
3.2497510624,-3.3131702694,8.6877001017\H,-5.1979961346,-2.6374014296,7.3180164194\H,-
4.8461491259,-1.4868282183,5.161844081\C,-0.8933681179,1.578511179,6.4147243452\
C,0.4083982769,1.4561277394,6.8711666932\C,1.479011103,1.7395362016,6.0340389234\C,1.2329
607975,2.1479442297,4.730424228\C,-0.0670188897,2.2692102272,4.2695443622\H,-
1.7218932614,1.3491337671,7.0706978584\H,0.587434302,1.1339217148,7.8907247731\H,2.49663
62816,1.6423827964,6.3940121963\H,2.0583769743,2.3654675953,4.0623237307\H,-0.2323573871,
2.5653832491,3.2410334559\\Version=ES64L-G09RevD.01\HF=-1415.0227357\ RMSD=5.303e-
09\RMSF=1.866e-06\Dipole=2.1352641,-0.9222881,0.0407973\Quadrupole=-5.6130783,-
3.1912872,8.8043655,6.4613508,12.9056733,-16.1934442\PG=C01 [X(C23H19N1O1S1)]\\@

cis-**25a**

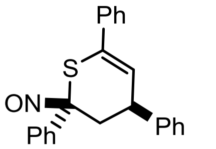

HF=-1415.0670916 a.u. (0)

1\1\GINC-LUPUS06\FOpt\RPBE1PBE\def2TZVP\C23H19N1O1S1\WURTHWE\27-Oct-2018\0\\#
pbe1pbe/def2tzvp emp=gd3bj opt=readfc nosym geom=check guess=read pop=NBO Freq
scrf(solvent=dichloromethane)\\Product: Other configuration at CHPh\\0,1\C,-0.2496920757,
0.0181460663,-0.1164100542\C,-0.0688639431,0.1505270767,1.2563177387\C,1.2155783575,
0.3749570762,1.7518209799\C,2.2945433236,0.4848717151,0.8904863119\C,2.1050034472,0.36220
37564,-0.4786674341\C,0.8321631792,0.1282003507,-0.9775618238\C,-1.2172177402,
0.0511788352,2.2427758095\S,-1.2709088418,1.6125886053,3.1392393658\C,-2.8395247507,
1.5189751018,3.9276561496\C,-3.7799642835,0.6159562319,3.638820766\C,-3.7174642364,-
0.4515826335,2.5988796286\C,-2.5603740167,-0.277343037,1.6092254743\N,-0.8322290954,-
1.159369234,3.0854020987\O,-0.4884704792,-0.9427452312,4.1977910652\H,-4.6275737206,-
0.3489576018,1.993757004\C,-3.0393729103,2.5640053735,4.9513043948\C,-4.2649120208,
3.2242222444,5.0500834736\C,-4.4724713318,4.1840501027,6.0274158212\C,-3.455630742,
4.5081015448,6.9154875685\C,-2.2292117667,3.8661968923,6.8175873027\C,-2.0200608336,
2.9048632542,5.841090423\H,-5.0513831364,2.9901093493,4.3420568775\H,-1.0649970761,
2.3966639017,5.7761319794\H,-1.4304043514,4.1117165925,7.5078678953\H,-3.616901312,

5.2626302634,7.6766252037\H,-5.4291508685,4.6900849437,6.0881627442\H,-2.4546538168,-1.1932239035,1.0232891111\H,-2.7998713575,0.5323227838,0.9176577523\C,-3.7844257784,-1.8494864374,3.191940284\H,-4.6866027775,0.6406483091,4.2366710872\H,1.372313219,0.4671199774,2.8208325877\H,3.2856280411,0.6620483553,1.2910265474\H,2.947776882,0.4453205874,-1.1548066846\H,0.6763228155,0.0297817534,-2.0454708742\H,-1.2338028452,-0.1628333218,-0.5276142165\C,-4.1132420291,-2.921503328,2.3639087287\C,-4.1754468958,-4.2126045005,2.8609404579\C,-3.9150966819,-4.4547199435,4.204149624\C,-3.5983689392,-3.3946910774,5.039122152\C,-3.5363472585,-2.1021733816,4.535588401\H,-4.3269575208,-2.7404289357,1.3148163401\H,-4.4324072596,-5.0328754419,2.2002489507\H,-3.9633126706,-5.4641660397,4.5959107925\H,-3.3945620654,-3.5714057083,6.0891535966\H,-3.2775164582,-1.2801772161,5.193305597\\Version=AS64L-G09RevD.01\HF=-1415.0670916\RMSD=8.081e-09\RMSF=2.112e-06\Dipole=-0.4690796,0.224184,-1.0010904\Quadrupole=8.1223768,-4.7666602,-3.3557165,-0.1133777,-1.3914856,7.3556798\PG=C01 [X(C23H19N1O1S1)]\\@

cis-**25a**-TS

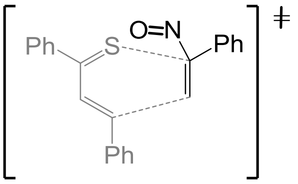

HF=-1415.011253 a.u. (1, -290.4896 cm^{-1})

1\1\GINC-R03N09\FTS\RPBE1PBE\def2TZVP\C23H19N1O1S1\WURTHWE\13-Aug-2019 \0\\# pbe1pbe/def2tzvp emp=gd3bj opt=(ts,noeigentest,readfc) geom=chec k guess=read scrf=(solvent=dichloromethane)pop=nbofreqnosym\\TS-Search for cis-25a\\0,1\C,-0.3321988179,0.610435817,0.0741279781\C,-0.0118465153,-0.0624953617,1.254114999\C,1.2662436856,-0.6051772559,1.3922106382\C,2.1989283259,-0.481714795,0.3766443799\C,1.8725789892,0.1896815716,-0.7942367551\C,0.6043358379,0.7344511305,-0.9392944666\C,-0.9958691804,-0.180227442,2.3358213857\N,-2.3514811391,0.0331039694,1.9866848128\C,-0.6728116431,-0.3166921953,3.6677620539\C,-0.9481954439,-2.3845546413,4.2128564336\C,-2.2863984858,-2.6297815435,3.9397373973\C,-2.7838180419,-2.8764562839,2.6439251861\S,-1.7982081414,-2.6460533237,1.3230819425\C,-0.4036382734,-2.3281222208,5.5716885453\C,0.9423164749,-2.6446068779,5.7630148875\C,1.5021952916,-2.6203567166,7.0296125831\C,0.7259946025,-2.2641401369,8.1232235283\C,-0.6115911968,-1.932382791,7.9424028736\C,-1.1731676383,-1.9630106169,6.6784031391\C,-4.2121752733,-3.2163671968,2.4971243978\C,-4.8543977401,-3.9869256554,3.4682338697\C,-6.1928465553,-4.3194353851,3.3328522365\C,-6.9128303827,-3.873149745,2.2339650036\C,-6.2831132785,-3.1019824318,1.2646729377\C,-4.9421226662,-2.7849948962,1.3894705456\H,-0.2532430639,-2.6660020573,3.4251771676\H,-4.2963836442,-4.3545817983,4.3218482324\H,-4.4433296315,-2.1856894064,0.6375001211\H,-6.8418910568,-2.7455367259,0.407126547\H,-7.9613438317,-4.1274274803,2.1308218653\H,-6.6732972363,-4.9320720181,4.0867805014\H,-1.4225979905,-0.025211175,4.3894578106\H,0.3611425951,-0.2817860096,3.9808965896\H,-3.0031821117,-2.5935704645,4.7518532343\H,-1.3205397419,1.035925572,-0.0418430504\H,0.3415273847,1.2637276463,-1.8480087454\H,2.6031915038,0.2873927723,-1.5886361827\H,3.1845142512,-0.9159809407,0.4981653356\H,1.5313787275,-1.1474322786,2.2926216572\H,-2.2139311739,-1.6881087327,6.5518052597\H,-1.2186669188,-1.6452636181,8.7928492841\H,1.1619375682,-2.239512668,9.1149840426\H,2.5465180691,-2.8768711985,7.1627832927\H,1.5488638898,-2.9183019649,4.905982834\O,-3.1348617778,-0.0189771007,2.9228614081\\Version=ES64L-G16RevB.01\HF=-1415.011253\RMSD=7.527e-09\RMSF=1.783e-06\Dipole=1.2176147,-0.5000942,2.0001227\Quadrupole=-5.6688985,-16.5669022,22.2358008,2.2075423,1.8481599,-14.8367614\PG=C01[X(C23H19N1O1S1)]\\@

cis-**26a**

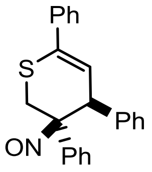

HF=-1415.0642944 a.u. (0)

1\1\GINC-LUPUS04\FOpt\RPBE1PBE\def2TZVP\C23H19N1O1S1\WURTHWE\27-Oct-2018\0\\# pbe1pbe/def2tzvp emp=gd3bj opt=readfc nosym geom=check guess=read pop=NBO Freq scrf(solvent=dichloromethane)\\Product \\0,1\C,3.6075061043,1.7124033277,0.0356166496\C, 2.76788749,2.1650565315,-0.9830483994\C,3.2938268198,3.0144111302,-1.9567365751\ C,4.6290105593,3.3861408034,-1.9223354848\C,5.4585172202,2.9201594324,-0.9126026204\ C,4.9419265627,2.0824103045, 0.0668156617\S,0.2550516359,3.0283075161,-1.4574871013\C,- 1.3357956804,2.1811135514,-1.402280295\C,-1.4212869274,1.0705871225,-0.3638477812\C,- 0.415081929,-0.0542084725,-0.7102531168\C,0.9802649596,0.4942452632,-0.7128874597\ C,1.3523666012,1.7450754941,-1.0191748663\C,0.0613092464,-1.3458856236,1.4203476326\C,- 0.1034088124,-2.4652563982, 2.2226654378\C,-0.874636089,-3.5327660614,1.7841289833\C,- 1.4740583796,-3.4752838392, 0.5341649417\C,-1.3078386962,-2.353689979,-0.2638805866\C,- 0.5464230088,-1.272894861, 0.1702286713\C,-2.8361064689,0.5392069455,-0.2269701356\C,- 3.2915719108,0.0411306542, 0.9939773027\C,-4.5681858241,-0.4847692671,1.1119266287\C,- 5.4089690431,-0.5371139247,0.0102708956\C,-4.9619323125,-0.053834298,-1.2097482126\C,- 3.6882175113,0.4837404752,-1.3264956416\H,3.2016328407,1.0800623835,0.8166505493\ H,2.655801764,3.370339711,-2.7576978022\H,5.0231466246,4.0395786608,-2.6920735905\ H,6.5015657918,3.213088918,-0.8848948801\H,5.5788705993,1.7244443201,0.8674952199\H,- 1.5416659336,1.769333695,-2.3878798494\H,-2.0686579734,2.9605678221,-1.194590811\N,- 1.0049182312,1.5633080059,1.005174874\H,-0.6768704045,-0.3758479195,-1.7256369634\ H,1.7721302108,-0.2122224955,-0.4864710771\H,0.6571278151,-0.512906024,1.7732924607\ H,0.3729364425,-2.502855758,3.1958412151\H,-1.0030795533,-4.4072852316,2.4115580034\H,- 2.0731742663,-4.3056599073,0.1782929575\H,-1.7843205503,-2.3110606423,-1.2378297767\H,- 2.6398788541,0.0474163736,1.8582128365\H,-4.9015843818,-0.8644228413,2.0707778638\H,- 6.4051400136,-0.954086633,0.1011060488\H,-5.6059666193,-0.0922127558,-2.0807117686\H,- 3.3677702213,0.8507704494,-2.2933034011\O,-1.1510106917,2.7341000419,1.1634353624\\ Version=AS64L-G09RevD.01\HF=-1415.0642944\RMSD=4.606e-09\RMSF=3.407e-06\Dipole=- 0.4930217,-1.0235116,-0.7682887\Quadrupole=10.0127204,-10.6681442,0.6554238,6.2323497, 5.6300263,-7.6122016\PG=C01 [X(C23H19N1O1S1)]\\@

cis-**26a**-TS

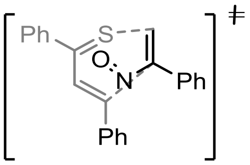

HF=-1415.0230689 a.u. (1, -73.4677 cm^{-1})

1\1\GINC-R02N29\FTS\RPBE1PBE\def2TZVP\C23H19N1O1S1\WURTHWE\09-Jun-2019\0\\# pbe1pbe/def2tzvp emp=gd3bj opt=(ts,noeigentest,calcfc) pop=nbo freq nosym scrf=(solvent=dichloromethane)\\Product: TS-Search \\0,1\C,-0.1470923858,- 0.0887251635,0.0778980928\C,-0.1457351283,-0.2435387593, 1.4663299076\C,1.0691911935,- 0.1442633825,2.1466163157\C,2.2470868042, 0.08950134,1.4546250327\C,2.2362635411, 0.2340398785,0.0749633649\C,1.0315531202,0.1458387114,-0.6103532012\C,-1.399500351,-

0.4989549837,2.1853442991\N,-1.3865909823,-0.1860542431,3.5670893214\O,-2.4368111421,-
0.3287468968,4.1620507278\C,-2.5111128719,-1.0456465225,1.6285187408\C,-
1.7916267089,2.510039229,2.0292037971\C,-0.5940141021, 3.1854612636,2.4655952795\C,-
0.4737412811,3.8027352761,3.7188715002\ C,0.7041387421, 4.4230430306,4.0831387525\
C,1.7873756148,4.440564407,3.2083785981\C,1.6861518769,3.8283381636,1.9679365302\C,0.5071
075302,3.2031522213,1.6020131733\C,-2.9793701477,2.462010496, 2.6785450051\C,-
4.1110540948,1.7297146526,2.2088976231\S,-4.081486071,0.8384653652, 0.7991965555\C,-
5.3272346568,1.7518150414,3.038877808\C,-5.2411431061,1.8016796657, 4.4338159366\C,-
6.3877867365,1.8009080004,5.2100375339\C,-7.6382981474,1.7755945174, 4.6082971027\C,-
7.7362658251,1.7340820431,3.2232286628\C,-6.5920531477,1.7104044792, 2.446591446\H,-
4.2717445816,1.7899246602,4.9166268781\H,-6.6623828613,1.6743696315, 1.3662384708\H,-
8.7096475321,1.7198381846,2.7470985968\H,-8.5348826376,1.7851343646, 5.2170941239\H,-
6.3037777994,1.8144848527,6.2903314891\H,-2.5039687937,-1.4373838819, 0.6212383251\H,-
3.3349722149,-1.3165973347,2.2743918637\H,-1.738252041,2.0144106067, 1.0644031697\H,-
3.0994270249,2.9900934761,3.6169100607\H,-1.3037507823,3.7892092634, 4.4146962394\
H,0.7864066387,4.8941664964,5.0554945989\H,2.7104016496,4.9275454623,3.5008728557\H,2.529
7501743,3.8298309736,1.2880506451\H,0.4293622489,2.7083802066,0.6406412309\H,1.084535939
2,-0.2527374987,3.2231690333\H,3.1807125222,0.1623279778,2.0005717183\ H,3.1585874518,
0.4191646181,-0.4630885039\H,1.0076464589,0.2683872766,-1.6871258572\H,-1.0832723511,-
0.1295491664,-0.4672958447\\Version=ES64L-G09RevD.01\HF=-1415.0230689\RMSD=5.012e-
09\RMSF=6.188e-06\Dipole=0.6284848,1.0528013,-0.1443495\Quadrupole=7.0344005,-4.3973244,-
2.6370761,2.0481686,-3.7376393,12.038128\PG=C01 [X(C23H19N1O1S1)]\\@

27a

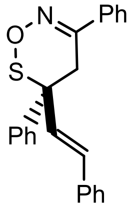

HF=-1415.0634127 a.u. (0)

1\1\GINC-LUPUS09\FOpt\RPBE1PBE\def2TZVP\C23H19N1O1S1\WURTHWE\26-Oct-2018\0\\#
pbe1pbe/def2tzvp emp=gd3bj opt=readfc nosym geom=check guess=read pop=NBO Freq
scrf(solvent=dichloromethane)\\product (not observed)\\0,1\C,-2.4098932568,-2.8926606719,
0.5310010253\C,-2.7586234955,-1.7852859148,-0.2485658401\C,-3.8524457695,-1.9028235212,-
1.1088921208\C,-4.5679938388,-3.0866891929,-1.2011235854\C,-4.2054483385,-4.1786186771,-
0.4270531301\C,-3.1240396349,-4.0738617936,0.4409101104\C,-2.0382679579,-0.5141338123,-
0.2050705259\C,-0.8924905037,-0.2787295676,0.4323597844\C,-0.153612217,1.0272253133,
0.4394945977\C,1.2258615909,0.8617772538,-0.1903944772\C,2.2091362296,0.0483643492,
0.6085205125\N,2.1523022355,-0.2615075415,1.8468282218\O,1.0637220804,0.1304141599,
2.6023305875\S,0.2137327828,1.5044787034,2.1670036578\C,-0.9466869534,2.1789061589,-
0.1353274093\C,-2.0777323486,2.6263535973,0.5489627392\C,-2.8417909376,3.6689129506,
0.0515639259\C,-2.4951227707,4.2743082757,-1.1484088041\C,-1.3803457508,3.8291377326,-
1.8417132471\C,-0.6080468779,2.7914685864,-1.3370840068\H,-2.3677043318,2.1401937953,
1.4739236204\H,-3.7136621521,4.0051111583,0.6003948996\H,-3.0930561887,5.0878417049,-
1.5421327281\H,-1.1047371693,4.2907064961,-2.782763709\H,0.254850158,2.4594904951,-
1.9002408601\H,-0.397109708,-1.0755627313,0.9793695955\H,-2.5000156692,0.2938707491,-
0.7657794651\H,-4.13927414,-1.0497477746,-1.7150777248\H,-5.412008515,-3.1549140818,-
1.8779226199\H,-4.7632407963,-5.1054449552,-0.4934663505\H,-2.8393177274,-
4.9202187552,1.0555932118\H,-1.5782851615,-2.8289897391,1.2230162832\H,1.6636201094,
1.8496490583,-0.3567876818\H,1.126438141,0.3830641848,-1.1690165085\C,3.3937203869,-

0.4794617756,-0.108120147\C,4.0997403853,-1.574041296,0.39561886\C,5.2067866208,-2.0646404836,-0.2723572408\C,5.6321880635,-1.4712146807,-1.4548771144\C,4.9371303621,-0.3857245788,-1.9642369161\C,3.822577033,0.1044919551,-1.2992288264\H,3.7636734164,-2.0387371672,1.3134511558\H,5.7389067722,-2.919841947,0.1279341051\H,6.4991034801,-1.8576924625,-1.9779280062\H,5.260608465,0.0852562764,-2.8850811688\H,3.297258898,0.9562841672,-1.7130626798\\Version=AS64L-G09RevD.01\HF=-1415.0634127\RMSD=4.615e-09\RMSF=1.750e-06\Dipole=-0.3519329,0.4008736,-2.0271238\Quadrupole=4.4698667,4.2136957,-8.6835624,-4.4614532,-5.6811487,-6.8331476\PG=C01 [X(C23H19N1O1S1)]\\@

27a-TS

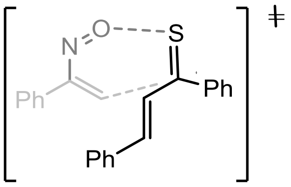

HF=-1415.0000785 a.u. (1, -344.9279cm^{-1})

1\1\GINC-PALMA337\FTS\RPBE1PBE\def2TZVP\C23H19N1O1S1\WURTHWE\03-Nov-2018\0\\# pbe1pbe/def2tzvp emp=gd3bj opt=(ts,noeigentest,readfc) geom=check guess=read pop=nbo freq nosym scrf=(solvent=dichloromethane)\\Product: other conformer: TS\\0,1\C,-0.0855308572,-0.2232817221,0.0322021175\C,-0.0826112198,-0.076422003,1.4183934145\C,1.1478752281,-0.0721874602,2.0833346708\C,2.3328097421,-0.194648271,1.382203103\C,2.3162036631,-0.3227195443,-0.0008185928\C,1.1034167269,-0.3384600102,-0.6715360557\C,-1.3523291995,0.0176441345,2.1767852989\C,-1.4609548439,0.8901972849,3.3446243096\C,-0.6506252308,1.9241917773,3.6153954296\C,-0.7942805236,2.8569995067,4.7241611271\C,-1.7764192326,2.7316280079,5.7137328509\C,-1.8618654483,3.6493375571,6.7440706927\C,-0.9699008796,4.714154529,6.8148015693\C,0.0109430613,4.8508386765,5.8436960966\C,0.09660856,3.9310208171,4.8109475178\S,-2.8131745771,-0.437161456,1.4709055118\C,-1.213499001,-1.9128594334,3.4786790377\C,-0.8121059489,-2.8203082605,2.5246914881\N,-1.6539590944,-3.1488299427,1.5028618542\O,-2.7891035333,-2.6063772675,1.5535858561\C,0.56046566,-3.3260405754,2.4141921681\C,1.3532939787,-3.4732094524,3.5534107449\C,2.6596773253,-3.9198550897,3.4492297623\C,3.1910098686,-4.2339161084,2.2053732613\C,2.4063093319,-4.1007750487,1.0680320744\C,1.1019901727,-3.6478868869,1.1689701658\H,1.1737653904,-0.0045871083,3.1638706512\H,3.2739714513,-0.2074834372,1.9191177353\H,3.245371072,-0.4228511965,-0.5494911661\H,1.0783004762,-0.4451594354,-1.7497248818\H,-1.0335119414,-0.2404101403,-0.4907092813\H,-2.3358713596,0.7095945707,3.9592621125\H,0.1820335526,2.1253128393,2.9490142614\H,0.8640992536,4.0399298524,4.05182767\H,0.7119112019,5.6762471213,5.8897051279\H,-1.0406649572,5.4308900686,7.624556151\H,-2.6292936711,3.5352801891,7.5010555751\H,-2.4801111093,1.9081862378,5.6826763263\H,-0.5055912467,-1.5326618952,4.2020352752\H,-2.266074851,-1.7904608304,3.6862389184\H,0.4987816341,-3.5109090137,0.2815063418\H,2.8185279654,-4.3362280723,0.0936801516\H,4.2143886542,-4.5814813832,2.1236304702\H,3.2631880006,-4.0311566773,4.3425226207\H,0.9389587264,-3.251824521,4.5303630838\\Version=EM64L-G09RevD.01\HF=-1415.0000785\RMSD=8.664e-09\RMSF=1.758e-06\Dipole=2.1509611,1.0450776,1.3305521\Quadrupole=-6.04431,-10.8594085,16.9037185,-8.8687329,0.5974251,5.7819985\PG=C01 [X(C23H19N1O1S1)]\\@

28a

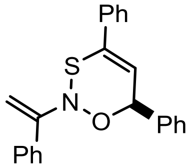

HF=-1415.0483667 a.u. (0)

1\1\GINC-LUPUS06\FOpt\RPBE1PBE\def2TZVP\C23H19N1O1S1\WURTHWE\26-Oct-2018\0\\# pbe1pbe/def2tzvp emp=gd3bj opt=readfc nosym geom=check guess=read pop=NBO Freq scrf(solvent=dichloromethane)\\Product 28a\\0,1\C,-0.0261670288,0.2878476817,0.0286009652\C,-0.1269924765,1.3481825237,0.9256850375\C,1.0128291003,1.9188508511,1.4709526844\C,2.2677064827,1.4417261646,1.1200121425\C,2.376350057,0.3880994458,0.2216628681\C,1.238161859,-0.1837175863,-0.3218862166\C,-1.2452821352,-0.3387822263,-0.5214779388\C,-2.3301774351,-0.5897220853,0.2148294081\N,-1.1166555184,-0.7398794989,-1.8642506136\S,-2.4566553668,-1.5505323735,-2.5708496813\C,-1.6503722634,-1.8910743063,-4.0889869384\C,-0.5401134063,-1.2415136892,-4.4494090596\C,0.0779332472,-0.0895084262,-3.7203898369\O,-0.7586123645,0.3742444567,-2.6652921896\C,0.2905137603,1.0950706962,-4.6264221851\C,-0.7749696001,1.6179197445,-5.3536500344\C,-0.5854206249,2.7132831708,-6.1795427052\C,0.6743758495,3.2897634227,-6.2930538219\C,1.7395816992,2.769962009,-5.5735660282\C,1.5454457932,1.6773165498,-4.7386129558\C,-2.3106792884,-2.9114945167,-4.9201062127\C,-2.3619988202,-2.7535100857,-6.3061517945\C,-2.9604693514,-3.7150450768,-7.1028002129\C,-3.5323330272,-4.8420120895,-6.5267659276\C,-3.5005126376,-5.0004274809,-5.1487435501\C,-2.8961688482,-4.042396661,-4.349656649\H,-1.9430182179,-1.8596488941,-6.7535892454\H,-2.8585886122,-4.1792432571,-3.2749559561\H,-3.943713293,-5.877626794,-4.6918601308\H,-4.0070007152,-5.5907081319,-7.1501947716\H,-2.9932575232,-3.5776215599,-8.1774195131\H,1.0478016255,-0.3892210921,-3.3028051699\H,-0.0358845262,-1.5432738726,-5.36147623\H,-1.7544424264,1.1604366795,-5.2662264322\H,-1.4200713984,3.1181801707,-6.7401712988\H,0.8234612167,4.1441982585,-6.9431432072\H,2.7234157223,3.2168843146,-5.6577740009\H,2.3755356469,1.2736160091,-4.1685401168\H,-3.2389117863,-0.9988055051,-0.2080183343\H,-2.3099696222,-0.4162050422,1.2821199602\H,-1.1062823245,1.7373781287,1.179338069\ H,0.9202931023,2.7474696002,2.1635285266\H,3.1589467787,1.8916224785,1.5418857585\H,3.353239525,0.0071179114,-0.0532706334\H,1.320678446,-1.0159707172,-1.0107671759\\Version=AS64L-G09RevD.01\HF=-1415.0483667\RMSD=3.598e-09\RMSF=2.231e-06\Dipole=0.5397706,-0.1110081,-0.6595493\Quadrupole=-4.0886389,-4.0519866,8.1406254,6.9849413,0.4700757,4.3585079\PG=C01 [X(C23H19N1O1S1)]\\@

28a-TS

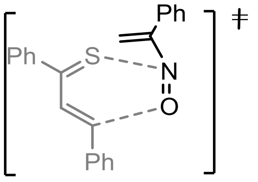

HF=-1415.0014361 a.u. (1, -314.2833 cm^{-1})

1\1\GINC-R09N48\FTS\RPBE1PBE\def2TZVP\C23H19N1O1S1\WURTHWE\16-Aug-2019\0\\#pbe1pbe/def2tzvpopt=(ts,noeigentest,readfc,maxstep=5)geom=checkguess=readscrf=(solvent=dichloromethane)pop=nbofreqnosymemp=gd3bj\\TS 28a\\0,1\C,0.0823575021,0.0667409231,0.0944801328\C,0.0550767551,0.2910866863,1.4732348132\C,1.2337582501,0.4700473875,2.1746277571\C,2.4540180602,0.4303218961,1.5104545294\C,2.4935173568,0.2026161492,0.1415819777\C,1.3150413899,0.0150339093,-0.5597011797\C,-1.1303242897,-0.1360896609,-0.6839098234\C,-2.3881865496,0.3460459392,-0.2996383051\C,-3.5219688242,0.1304383178,-1.0729961679\S,-

3.4362966822,-0.8069101502,-2.4727856748\C,-4.8305745712,0.6271690219,-0.6028748336\C,-5.7964507282,1.0391698166,-1.5219229929\C,-7.0232711389,1.5146606777,-1.0922873831\C,-7.3134522064,1.5694072645,0.2647512574\C,-6.3660777216,1.1496395521,1.1878253969\C,-5.1315732924,0.6890391287,0.7592169491\H,-2.4952560405,0.8389139528,0.6586436002\H,-0.9896753742,-0.3141591747,-1.7473245008\H,-0.8902083233,0.3111015733,2.001893238\H,1.2040792799,0.6391930898,3.2444392408\H,3.3753675506,0.5734606964,2.062939219\H,3.4438502421,0.1678470615,-0.3775417584\H,1.33964853,-0.1695638184,-1.6280505851\H,-4.4101401474,0.3415572251,1.4887849424\H,-6.5907105785, 1.1746503194,2.2477961602\H,-8.2768458759,1.9343932232,0.6013156853\H,-7.7570289307, 1.8431851528,-1.8190695029\H,-5.5687016213,0.9906546719,-2.5804154025\O,-1.5659242166,-1.9837265226,-0.3865426867\N,-2.0128558504,-2.4939403621,-1.4427376557\C,-3.0684983488,-3.4385964084,-1.2115659797\C,-3.3805970733,-3.8587468254,0.0177249722\C,-3.6489466436,-3.9847203851,-2.4491498728\C,-4.9762173342,-4.4110571534,-2.4783306804\C,-5.5267876069,-4.9254707292,-3.6403321816\C,-4.7612209307,-5.0168427531,-4.7952575539\C,-3.4415136495,-4.5886223651,-4.7774082721\C,-2.8875281753,-4.0766353164,-3.614239244\H,-2.9026793791,-3.4247848263,0.8854626443\H,-4.0977213408,-4.6546556585,0.1658354011\H,-1.856771602,-3.7457517039,-3.6005953278\H,-2.8364267912,-4.6566063516,-5.6743060558\H,-5.1935153508,-5.41533975,-5.7057887934\H,-6.562395001,-5.2455744907,-3.6476634356\H,-5.5881987266,-4.3135482298,-1.5891390677\\Version=ES64L-G16RevB.01\HF=-1415.0014361 \RMSD=3.657e-09\RMSF=1.262e-06\Dipole=0.3857003,0.7438454,1.326786\ Quadrupole=10.8625108,-11.5085059,0.6459951,-1.1723096,-6.3062033,1.4702624\PG=C01[X(C23H19N1O1S1)]\\@

29a

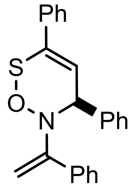

HF=-1415.0472325 a.u. (0)

1\1\GINC-R01N03\FOpt\RPBE1PBE\def2TZVP\C23H19N1O1S1\WURTHWE\08-Jun-2019\0\\# pbe1pbe/def2tzvp emp=gd3bj opt pop=nbo freq nosym int=(Acc2e=11,grid=ultrafine) scrf=(solvent=dichloromethane)\\product 29a after PCM\\0,1\C,2.2194941887,-3.1270439596, 2.5412720749\C,2.3302668802,-1.9954767889,1.7320977377\C,3.4967340213,-1.2330385524, 1.7964259131\C,4.5186521298,-1.5852458054,2.6622327026\C,4.398255261,-2.7102915057, 3.4677278847\C,3.2470508113,-3.4811826597,3.4021104271\S,0.4373084652,-2.9772696061, 0.0458820453\O,-0.3473078607,-2.1413145005,-1.1402982272\N,-1.156146593,-1.1328203236,-0.5530771527\C,-0.2902564343,0.032507743,-0.3111912501\C,0.8611060102,-0.3698282521, 0.5559577544\C,1.2416848923,-1.6229925077,0.8154626903\C,-0.5344880783, 1.8826577464,-1.9934128545\C,-0.1858133435,2.5195872643,-3.1752700705\C,0.8456113155, 2.0169330776,-3.9555615138\C,1.5296396333,0.8810179331,-3.5438455693\C,1.1834913472, 0.2493394887,-2.3587163454\C,0.1415384739,0.7403076966,-1.5772654799\C,-2.3187245256,-0.9302491578,-1.313895211\C,-2.6014201622,-1.6002279848,-2.4332582647\C,-3.9869762119, 0.9089312669,-1.5035477314\C,-3.2580704901,0.0327058077,-0.701583176\C,-3.4426109678, 0.0708827102, 0.6805602917\C,-4.3442097916,0.9593224584,1.2448024512\C,-5.0716304984, 1.8235991081, 0.4379146028\C,-4.8905462167,1.7944154564,-0.9381988117\H,1.3124434111,-3.7223948187, 2.5178228755\H,3.6063930537,-0.3702736813,1.149809094\H,5.4197683459,-0.9841014238, 2.7000819043\H,5.2013369025,-2.9865618398,4.1408112231\H,3.1431026504,-4.3595242414, 4.0283554086\H,-0.9104172638,0.7249289438,0.264700803\ H,1.4045025562,0.4457717355, 1.020449635\H,-1.3441208437,2.2745795127,-1.3869878669\H,-0.7200387346,3.4101181495,-3.4862636874\H,1.1203383051,2.5124420248,-4.8796713466\H,2.3409715746,0.4880776119,-4.1459494357\H,1.7272604195,-0.6294545534,-2.0337749268\H,-1.9037927468,-2.2977474032,-

2.8722746503\H,-3.5699808421,-1.4765750002,-2.8974133317\H,-3.8239703297,0.9057714252,-2.5748147636\H,-2.8804542917,-0.6102790508,1.3086742408\H,-4.4824230437,0.9736594204,2.3197801309\H,-5.7738214303,2.5205933831,0.880351673\H,-5.4466639485,2.4729486526,-1.5746668968\\ Version=ES64L-G09RevD.01\HF=-1415.0472325\RMSD=5.909e-09\RMSF=8.573e-06\Dipole=0.2993366,1.0301886,0.5536834\Quadrupole=3.5062455,-4.5852739,1.0790284,-8.0808271,3.0557724,-7.1802025\PG=C01 [X(C23H19N1O1S1)]\\@

29a-TS

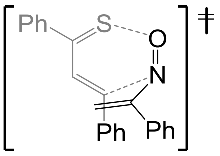

HF=- 1415.0163308 a.u. (1, -239.2487cm^{-1})

1\1\GINC-PALMA312\FTS\RPBE1PBE\def2TZVP\C23H19N1O1S1\WURTHWE\28-Oct-2018\0\\# pbe1pbe/def2tzvp opt=(ts,noeigentest,readfc) geom=check guess=read emp=gd3bj pop=nbo freq nosym scrf=(solvent=dichloromethane)\\Product: TS-Search\\0,1\C,0.2850885604,-0.3343466986,-0.0424511465\C,0.1440587607,-0.3052999006,1.3353600825\C,1.2572623656,-0.1406970624,2.1583769553\C,2.5146270745,0.0001626762,1.5719190187\C,2.6539469848,-0.023913902,0.1942728579\C,1.5414402932,-0.1949117699,-0.617129169\C,1.1114109941,-0.0890707754,3.6194760256\C,0.1129566066,0.4955064651,4.2805857292\N,2.2189687964,-0.6769734794,4.3350273955\O,2.241962636,-0.5105580468,5.5440335935\C,2.4382639455,-2.6512452457,4.0236422458\C,3.3031016761,-2.898026605,2.8711677807\C,2.817638718,-3.2990512423,1.6256801545\C,3.684695442,-3.5107881484,0.568773886\C,5.0498331575,-3.3183511693,0.7344907611\C,5.5462345944,-2.9203802275,1.9689611492\C,4.6789859315,-2.7177316236,3.0280680596\C,1.0707293212,-2.9343806358,4.0392422303\C,0.2528037591,-2.7696192705,5.1609248817\S,0.8667523242,-2.212779755,6.6175923292\C,-1.2046342182,-2.9736924254,5.0111851494\C,-1.85846062,-2.6851136881,3.811529677\C,-3.2272343454,-2.8618620866,3.6906044091\C,-3.9652931374,-3.3453591836,4.7616987915\C,-3.3251968718,-3.645433363,5.9569911848\C,-1.9595183279,-3.4557195181,6.0814589009\H,-1.2987139015,-2.2900747679,2.9734771005\H,-1.4574516581,-3.6890749354,7.0130676875\H,-3.8927037764,-4.0320662135,6.7954835385\H,-5.0352182081,-3.4887896865,4.6653225552\H,-3.719556405,-2.6147208854,2.7571324834\H,2.937368811,-2.6491961144,4.9888950963\H,0.5842225852,-3.186926872,3.1053606496\H,1.7553369663,-3.4371533786,1.4695525653\H,3.292636308,-3.8191767052,-0.3931672498\H,5.7261998997,-3.4805478379,-0.0967053081\H,6.6108951531,-2.7718459608,2.1057989134\H,5.0625987845,-2.4053556118,3.9933696552\H,0.0936416603,0.4777736151,5.3625728101\H,-0.6817682363,1.0045621512,3.7521866805\H,-0.8379995552,-0.4165474656,1.7804850024\H,3.3859892323,0.1275887279,2.2026196843\H,3.6377194502,0.0850961065,-0.2469078187\H,1.6522331014,-0.2208297557,-1.6949379213\H,-0.5882377382,-0.4707917276,-0.6696295768\\Version=EM64L-G09RevD.01\HF=-1415.0163308\RMSD=9.205e-09\RMSF=2.554e-06\Dipole=-0.2643968,-0.2685759,-2.5243574\Quadrupole=23.1881024,2.0459476,-25.23405,0.4732496,-11.489539,6.1362521\PG=C01 [X(C23H19N1O1S1)]\\@

5. References theoretical part

[1] Gaussian 16, Revision B.01, M. J. Frisch, G. W. Trucks, H. B. Schlegel, G. E. Scuseria, M. A. Robb, J. R. Cheeseman, G. Scalmani, V. Barone, G. A. Petersson, H. Nakatsuji, X. Li, M. Caricato, A. V. Marenich, J. Bloino, B. G. Janesko, R. Gomperts, B. Mennucci, H. P. Hratchian, J. V. Ortiz, A. F. Izmaylov, J. L. Sonnenberg, D. Williams-Young, F. Ding, F. Lipparini, F. Egidi, J. Goings, B. Peng, A. Petrone, T. Henderson, D. Ranasinghe, V. G. Zakrzewski, J. Gao, N. Rega, G. Zheng, W. Liang, M. Hada, M. Ehara, K. Toyota, R. Fukuda, J. Hasegawa, M. Ishida, T. Nakajima, Y. Honda, O. Kitao,

H. Nakai, T. Vreven, K. Throssell, J. A. Montgomery, Jr., J. E. Peralta, F. Ogliaro, M. J. Bearpark, J. J. Heyd, E. N. Brothers, K. N. Kudin, V. N. Staroverov, T. A. Keith, R. Kobayashi, J. Normand, K. Raghavachari, A. P. Rendell, J. C. Burant, S. S. Iyengar, J. Tomasi, M. Cossi, J. M. Millam, M. Klene, C. Adamo, R. Cammi, J. W. Ochterski, R. L. Martin, K. Morokuma, O. Farkas, J. B. Foresman, D. J. Fox, Gaussian, Inc., Wallingford CT, 2016.

[2] Gaussian 09, Revision D.01, M. J. Frisch, G. W. Trucks, H. B. Schlegel, G. E. Scuseria, M. A. Robb, J. R. Cheeseman, G. Scalmani, V. Barone, B. Mennucci, G. A. Petersson, H. Nakatsuji, M. Caricato, X. Li, H. P. Hratchian, A. F. Izmaylov, J. Bloino, G. Zheng, J. L. Sonnenberg, M. Hada, M. Ehara, K. Toyota, R. Fukuda, J. Hasegawa, M. Ishida, T. Nakajima, Y. Honda, O. Kitao, H. Nakai, T. Vreven, J. A. Montgomery, Jr., J. E. Peralta, F. Ogliaro, M. Bearpark, J. J. Heyd, E. Brothers, K. N. Kudin, V. N. Staroverov, R. Kobayashi, J. Normand, K. Raghavachari, A. Rendell, J. C. Burant, S. S. Iyengar, J. Tomasi, M. Cossi, N. Rega, N. J. Millam, M. Klene, J. E. Knox, J. B. Cross, V. Bakken, C. Adamo, J. Jaramillo, R. Gomperts, R. E. Stratmann, O. Yazyev, A. J. Austin, R. Cammi, C. Pomelli, J. W. Ochterski, R. L. Martin, K. Morokuma, V. G. Zakrzewski, G. A. Voth, P. Salvador, J. J. Dannenberg, S. Dapprich, A. D. Daniels, Ö. Farkas, J. B. Foresman, J. V. Ortiz, J. Cioslowski, D. J. Fox, Gaussian, Inc., Wallingford CT, 2009.

[3] A. D. Becke, *J. Chem. Phys.* 1**993,** *98,* 5648–5652; C. Lee, W. Yang, R. G. Parr, *Phys. Rev. B* **1988**, *37*, 785–789.

[4] S. Grimme, S. Ehrlich, L. Goerigk, *J. Comput. Chem.* **2011**, *32*, 1456–1465; for a review, see: S. Grimme, A. Hansen, J. G. Brandenburg, C. Bannwarth, *Chem. Rev.* **2016**, *116*, 5105–5154.

[5] J. P. Perdew, K. Burke, M. Ernzerhof, *Phys. Rev. Lett.*, **77** (1996) 3865–3868; J. P. Perdew, K. Burke, M. Ernzerhof, *Phys. Rev. Lett.*, **78** (1997) 1396; C. Adamo, V. Barone, *J. Chem. Phys.*, **110** (1999) 6158–6169; M. Ernzerhof, G. E. Scuseria, *J. Chem. Phys.*, **1999**, *110*, 5029–5036,

[6] J.Tomasi, B. Mennucci, R. Cammi, *Chem. Rev.* **2005**, *105*, 2999–3093.

[7] Details of the quantum chemical calculations may be obtained from E.-U. W. upon request.
